# Teaching troubleshooting skills to graduate students

**DOI:** 10.7554/eLife.100761

**Published:** 2024-09-17

**Authors:** Gina Partipilo, Yang Gao, Alexis J Holwerda, Yassir Lekbach, Ismar E Miniel Mahfoud, Trevor R Simmons, Bailey M Tibbett, Rebecca E Wilen, Marcus S Benyamin, Benjamin K Keitz

**Affiliations:** 1 McKetta Department of Chemical Engineering, University of Texas at Austin Austin United States; 2 Interdisciplinary Life Sciences Graduate Program, University of Texas at Austin Austin United States

**Keywords:** point of view, experimental techniques, troubleshooting, problem solving, graduate school, training, None

## Abstract

Troubleshooting is an important part of experimental research, but graduate students rarely receive formal training in this skill. In this article, we describe an initiative called Pipettes and Problem Solving that we developed to teach troubleshooting skills to graduate students at the University of Texas at Austin. An experienced researcher presents details of a hypothetical experiment that has produced unexpected results, and students have to propose new experiments that will help identify the source of the problem. We also provide slides and other resources that can be used to facilitate problem solving and teach troubleshooting skills at other institutions.

## Introduction

Researchers are routinely confronted with experiments and equipment that do not work as expected. A negative control, for example, might yield an appreciable signal, or a piece of equipment might fail to produce any signal at all. Diagnosing and fixing such problems – a process known as troubleshooting – is, therefore, an essential skill for any researcher (Jonassen, 2000; Verderame et al., 2018). However, PhD students rarely receive formal training in troubleshooting, and are expected to acquire this skill ‘on the fly’ as they progress through graduate school (Barnard and Shultz, 2020). In this article, we describe an approach called Pipettes and Problem Solving that has been used to teach troubleshooting skills to graduate students at the University of Texas at Austin.

Our approach is designed to be similar in nature to a journal club, with more experienced researchers passing on their knowledge to those with less experience at a meeting that lasts between 30minutes and an hour. Each meeting is led by a different member of the research group who, prior to the meeting, draws on their own experiences to create a scenario involving an unexpected experimental outcome. The overall goal of the meeting is for the group to work together to identify the source of the problem.

**Figure 1.**
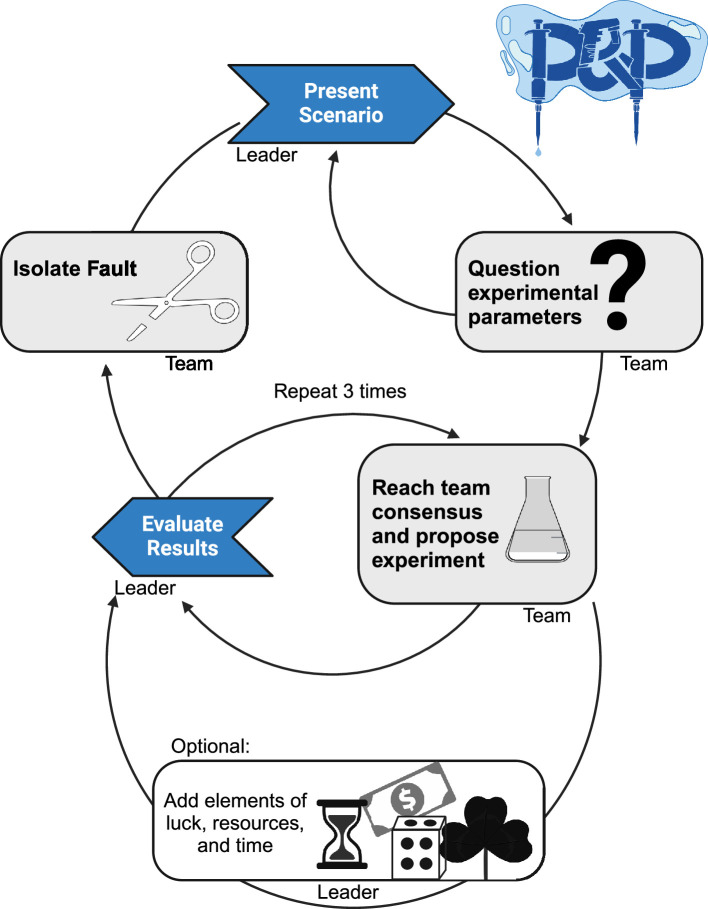
How Pipettes and Problem Solving works. The leader presents a scenario in which a hypothetical experiment has produced unexpected results. Graduate students ask questions about the experimental setup and the results. The students then discuss the scenario and potential experiments that might help to isolate the problem that has caused the unexpected results. Once they have reached a consensus on a suitable experiment, the leader reveals the results of their proposed experiment. Based on these results, the students try to figure out the source of the problem that is leading to the unexpected results. If they are correct, the exercise is over. If they are wrong, the cycle can be repeated. Elements of luck or chance can be introduced by rolling dice to determine, for example, any time or budget constraints on the proposed experiments. The Pipettes and Problem Solving logo (top right) was designed by students.

In this article, we describe how we have implemented Pipettes and Problem Solving at our own institution (Figure 1) and provide resources that will help other researchers experiment with this approach in their own labs and departments (see Table 1). These resources span a range of topics in the biological and chemical sciences. Moreover, we are confident that this approach could be extended to other fields of research.

**Table 1.** Resources for the eight scenarios listed in this table are provided in Supplementary file 1. The following is provided for each scenario: background information; a description of the scenario; the protocol for the experiment that produced the unexpected result; the results of the experiment; information on the source of the error; background information that can be used to answer questions; references; and example slides that can be used in real meetings.

Scenario	Key words	1/2/mundane
MTT assay	Mammalian cell biology; cytotoxicity; analytical chemistry	1
Membrane surface charge	Synthetic biology; bioelectronics; imaging electrochemical devices	2
Streptavidin-conjugation assay	Antibody; molecular biology; biochemistry; immunology	1
Golden gate cloning	Cloning; Golden Gate assembly; PCR; primers; synthetic biology	2
Recombinant quorum sensing expression and activity verification	quorum sensing; synthetic biology; plasmid cloning structural biology	2
Gibson cloning troubles	Synthetic biology; Gibson assembly; genetic engineering	Mundane
T cell cytokine release ELISA	Mammalian cell biology; T cell activation; cytokine release	1
Metabolite tracking by aqueous FTIR spectroscopy	Fourier transform infrared (FTIR) spectroscopy; analytical chemistry; synthetic biology	1

## Pipettes and Problem Solving: The practicalities

Two types of scenarios can be proposed by the meeting leader: the first involves an experiment with a known outcome that is returning atypical results (such as a negative control giving a positive result, and vice versa). This type of scenario is useful for teaching the use of appropriate controls, troubleshooting fundamentals, and instrument/laboratory/analytical techniques. Such scenarios are also helpful in illustrating how researcher-driven shortcuts, material aging, and general randomness can all contribute to an unexpected result.

The second type of scenario is more complicated in that the ‘right’ outcome is not known in advance. Most of the problems that require troubleshooting in research labs are of this second type. A typical example might be developing a new assay and demonstrating that it works. The assay is unlikely to work as intended the first time around, so it will be necessary to perform additional experiments with different controls, and/or characterize various compounds, strains or samples, before attempting the original experiment again. This type of scenario is useful for teaching hypothesis development, proper controls, new analytical techniques, and more advanced troubleshooting.

In preparation for either type of scenario, the leader must create 1–2 slides describing the hypothetical experimental setup and mock results from this experiment. The leader must also prepare hypothetical background information that could be useful for troubleshooting. For example, when were the relevant instruments last calibrated or serviced?; what are the temperature and humidity levels in the lab?; and what are other researchers in the hypothetical lab working on?

**Figure 2.**
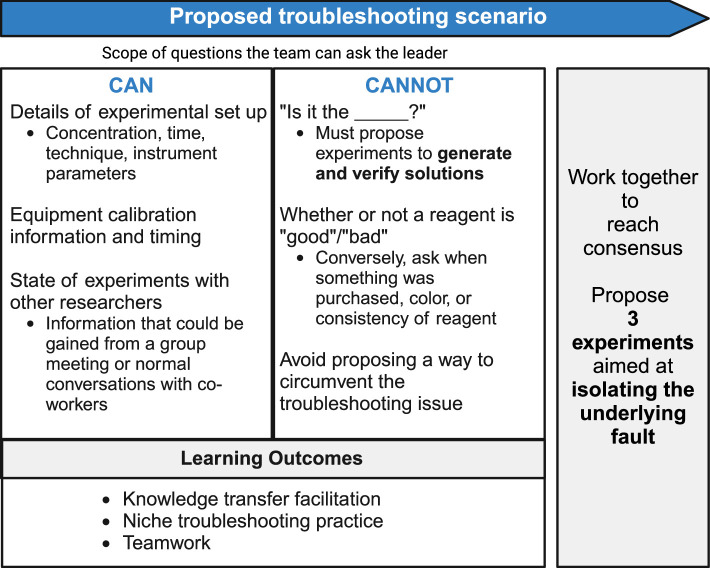
Asking the right questions. The aim of Pipettes and Problem Solving is to teach graduate students how to troubleshoot experiments that are giving unexpected results. To achieve this goal students are encouraged to ask questions that will help them to design experiments that will help them to identify the source of the problem (first column). They are not allowed to guess the problem, or to suggest ways of working around the problem (second column).

The leader presents the slides containing the experimental setup and the mock results to start the meeting, and then answers any questions the students have about the setup (such as timings, concentrations, temperatures, solvents and so so). Students can also ask questions throughout the meeting – ideally these will be fairly specific questions that the leader (as the person who ‘performed’ the hypothetical experiment) will be able to answer, possibly using the hypothetical background information they have prepared. The leader is encouraged to not answer questions that require an amount of subjectivity (Figure 2).

A major challenge for the students is to understand the science behind the hypothetical experiment: with only 1–2 slides at their disposal, the leader is not able to go into detail about the scientific background underlying the experiment, so the students are encouraged to research and discuss the topic during the meeting.

The students must then propose a limited number of experiments to help them troubleshoot the hypothetical problem. To foster conversation and collaboration, the students in the group must reach a full consensus on the proposed experiments. The students are also encouraged to discuss all possibilities and to settle on a single experiment to do first. The leader, who knows the source of the problem, then provides mock results for the proposed experiment. Based on this information the group can then either guess what is causing the problem, or propose another experiment. After a set number of experiments (typically three), the group must reach a consensus on the source of the problem, after which the leader will reveal the results.

The number of experiments can be increased or decreased at the discretion of the leader. The leader can also reject experiments that they deem to be too expensive, dangerous, or time-consuming, as well as experiments that require equipment that is not readily available at the host institution.

The students are encouraged to ask questions and work together; however, they should avoid asking questions like “is it the xxxx?” or “did the yyyyy go ‘bad’?”, as the only way to answer such questions is to run additional experiments! Conversely, they could ask the color of a solution or the time something was purchased. When proposing experiments the students are not allowed to iterate on the experiment in a way to circumvent the failure. Although proposing a new experiment to circumvent the problem could be an interesting exercise, it would not help the group to identify the source of problem, which is the purpose of Pipettes and Problem Solving.

There is often an element of chance or luck in research, and bad luck is sometimes the reason why an experiment does not work. For instance, mechanical noise might create a strange signal in one experiment, but not another, so we encourage leaders to include such seemingly mundane sources of error (such as contamination, miscalibration, instrument malfunctions and software bugs). In such cases we suggest that the leader gives the group a hint about the problem being mundane. The leader should keep in mind that the goal of the exercise is to foster troubleshooting instincts, not to ‘stump’ the group.

Another twist that can be added is to use dice to reflect various aspects of the research process. For example, the leader could roll dice to determine how long the proposed experiment could take: a low number could mean that the group has to propose an experiment that can be done in a day, whereas a high number could mean that the experiment is allowed to take a week or longer. A similar approach could be used to determine the budget for the proposed experiment. Adding game-related elements like dice can improve participation, particularly among less experienced group members.

An effective round of Pipettes and Problem Solving should take between 30 and 60minutes. The students should be challenged, but not so much as to decrease their confidence. If the meeting appears to be taking longer than the allotted time, the leader can intervene to provide additional time, information, or support in the form of hints – however, it should remain a balanced and student-led discussion.

## Pipettes and Problem Solving: An example

To give readers a better idea of how Pipettes and Problem Solving works in practice, we describe an actual meeting at the University of Texas Austin in which the leader describes a hypothetical cell viability assay (MTT Assay; see example 1 in Supplementary file 1). In the hypothetical experiment, a researcher is interested in the cytotoxic effect of a protein aggregate on human neuroblastoma cells; however, unexpectedly, there are very high error bars, and much higher than expected values. The leader described the workflow and presented simulated data with a high amount of sample variance. The rest of the group started by returning to the plotted data and discussing two major points: Were the appropriate controls run? What are the culturing conditions of this particular mammalian cell line? The group began researching both questions, and discussing what elements should be included in the first experiment.

The first discussion was around finding an appropriate negative control in the form of a cytotoxic compound. They discussed that an ideal compound would exhibit a range of behavior from low- to high- cytotoxicity. Next, the discussion turned to the culturing of the particular cell line, which was revealed to be dual adherent and non-adherent. The group discussed considerations of culturing dual adherent cell lines and whether this property was contributing to the high degree of variability. It was at this point that someone who had experience with mammalian cell viability assays mentioned the source of the error: aspiration of cells during the washes. This was taken up by the group dialog which led to a conversation surrounding the wash steps.

The group proposed only two experiments, despite discussing the scenario for nearly an hour: the first was to remove wash steps and to look only at a negative control instead of the protein aggregate. However, this introduced a new source of error (background noise from fetal bovine serum (FBS) and phenol red). The group quickly realized their error after looking at the detailed protocol and decided that the washes were strictly required. They examined the possibility that insufficient washing was the initial source of noise the entire time.

The second experiment they proposed was to add an additional wash step and to specifically use a pipette to aspirate the supernatant. They emphasized taking care to examine the cell density after each step, and several people mimed appropriately slow aspiration mentioning things like “placing the pipette on the well wall” and “slightly tilting the plate”. In addition to the wash step, the group proposed this experiment be done with both a negative control and the protein aggregate in order to directly compare the two.

Ultimately, the source of error was user generated, and by focusing on the aspiration technique the group was able to uncover the source of error and achieve appropriate results. The inclusion of a negative control in the final experiment allowed them to see that the assay was functioning correctly and that the protein aggregate cytotoxicity study yielded useful data. Importantly, we note that there was also a discussion about particular lab techniques including specific motion and speeds, which is a conversation that might not have been facilitated otherwise.

## Conclusions

Pipettes and Problem Solving was developed in October 2022 largely because of challenges with training new graduate students during and after the COVID-19 pandemic. This student-led meeting takes the form of sharing interesting methods, journal articles, and discussing particular instrumentation. Since the advent of Pipettes and Problem Solving, we have added it as a regular component to group meetings and have performed a session roughly once a month for almost two years. Between 3 and 10 graduate students, undergraduate students, and postdoctoral researchers attend per session. Participation is optional, but most of the group chooses to attend. It has also been piloted at another two research groups at our university.

We have found that the challenge of a troubleshooting scenario, with the near instant gratification of hypothetical experimental results, is a very engaging environment for graduate students, postdocs, and undergraduate students alike. A senior graduate student in a lab that had recently tried Pipettes and Problem Solving for the first time said: “It stimulated a lot of great discussion both from newer lab members thinking about problems they haven’t experienced yet and from experienced lab members who were able to easily share their thought processes behind troubleshooting”. In our lab, it has provided the opportunity to talk about specific instrument quirks, common lab errors, and potential experimental pitfalls, preserving this information as students graduate and transition into new roles (Jonassen and Hung, 2006).

## Additional files

Supplementary file 1. Resources for the eight scenarios listed in Table 1 (zip file). For each scenario there is a Word file that contains the following: background information; a description of the scenario; the protocol for the experiment that produced the unexpected result; the results of the experiment; information on the source of the error; background information that can be used to answer questions; and references. There is also a PowerPoint file for each scenario that contains example slides that can be used in real meetings. There are also templates for the Word and PowerPoint files.
