# Supplementary material for: Teaching troubleshooting skills to graduate students
Source: eLife. 2024 Sep 17;13:e100761. doi: 10.7554/eLife.100761 (PMC11407763; doi:10.7554/eLife.100761)
Supplement: Supplementary file 1. — For each scenario there is a Word file that contains the following: background information; a description of the scenario; the protocol for the experiment that produced the unexpected result; the results of the experiment; information on the source of the error; background information that can be used to answer questions; and references. There is also a PowerPoint file for each scenario that contains example slides that can be used in real meetings. There are also templates for the Word and PowerPoint files. [file elife-100761-supp1.zip › Final Scenarios/Example6.pptx]

## Slide 1
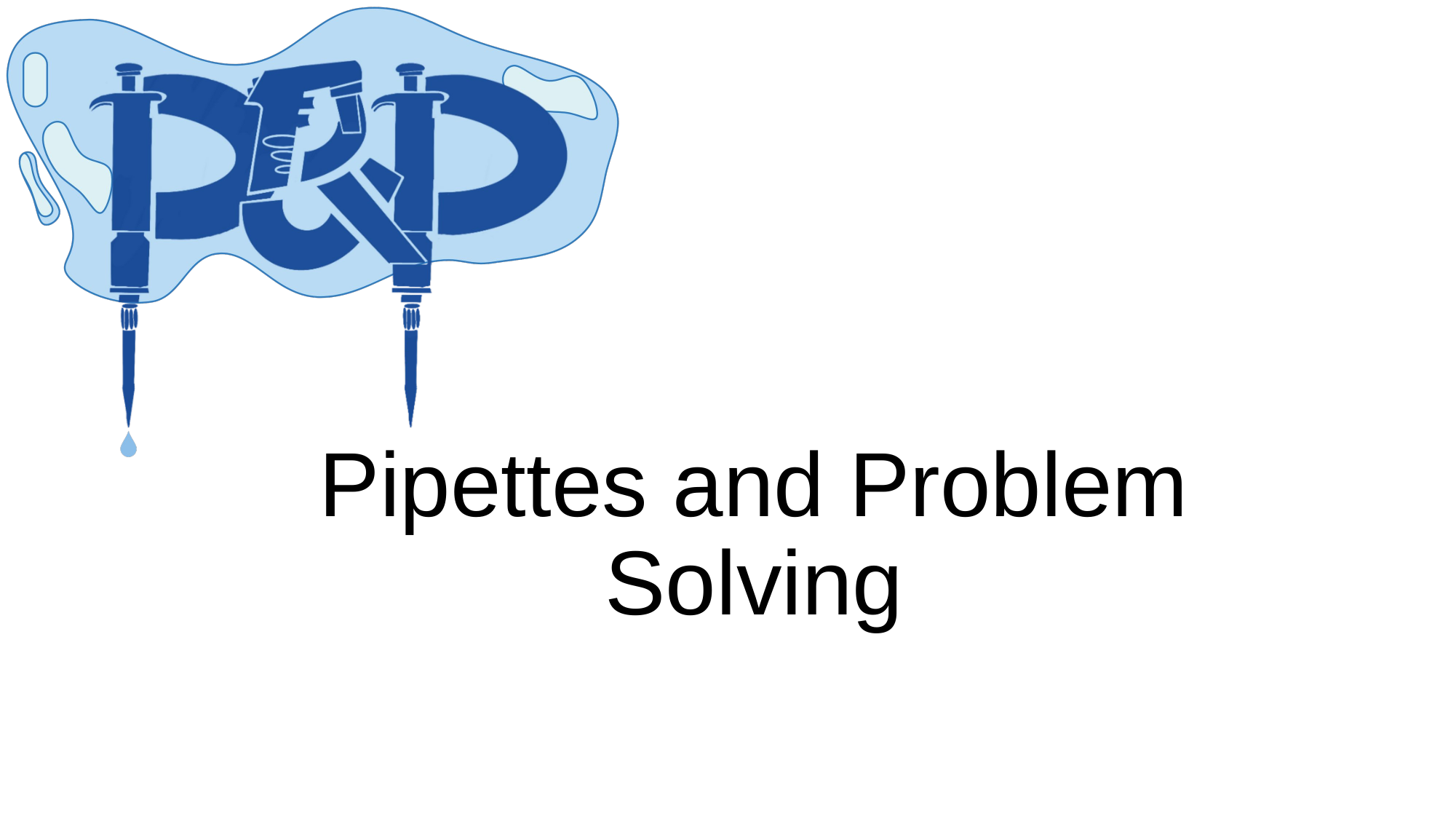

# Pipettes and Problem Solving

## Slide 2
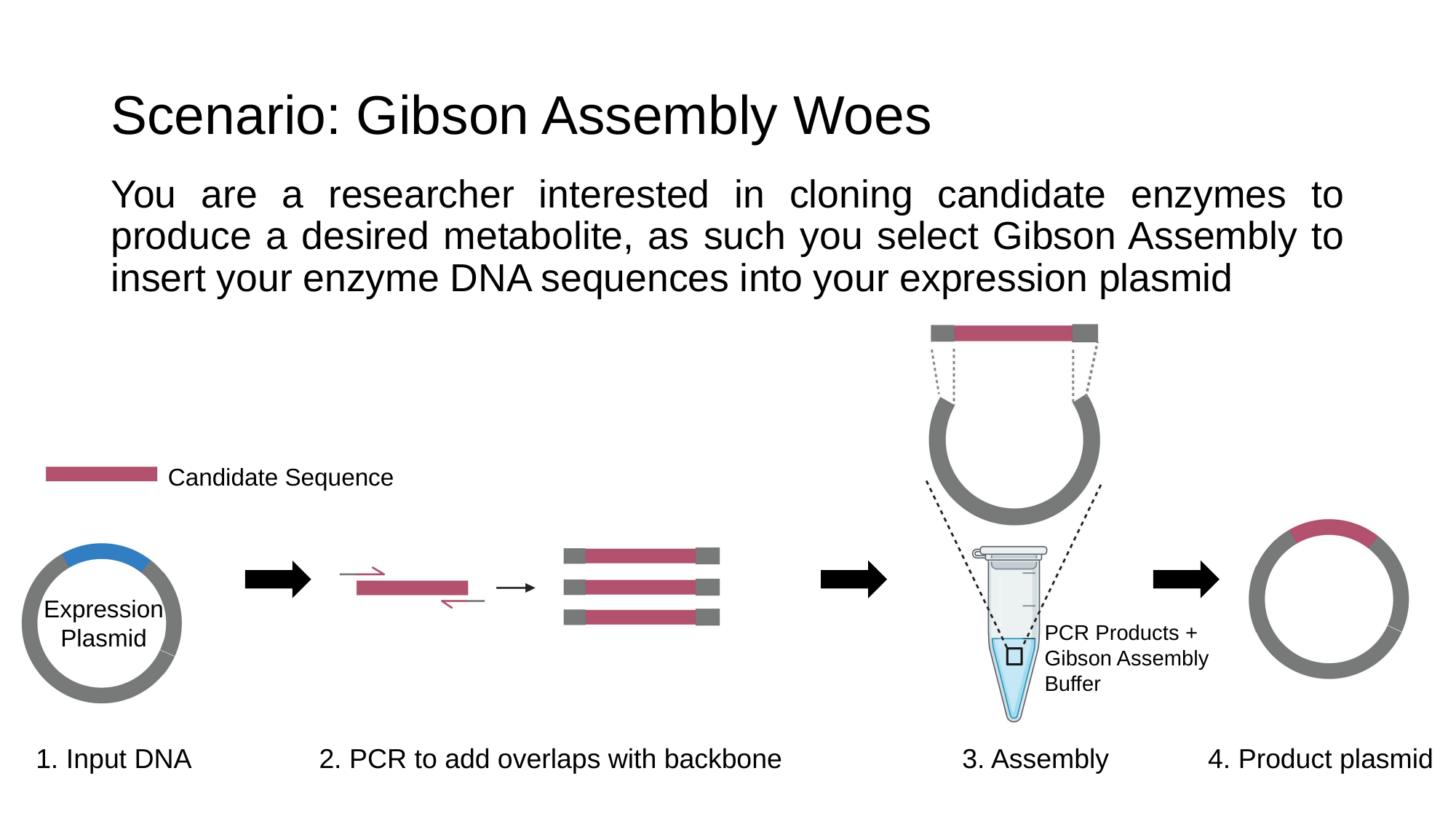

# Scenario: Gibson Assembly Woes
You are a researcher interested in cloning candidate enzymes to produce a desired metabolite, as such you select Gibson Assembly to insert your enzyme DNA sequences into your expression plasmid
Candidate Sequence
Expression
Plasmid
PCR Products +
Gibson Assembly Buffer
1. Input DNA
2. PCR to add overlaps with backbone
3. Assembly
4. Product plasmid

## Slide 3
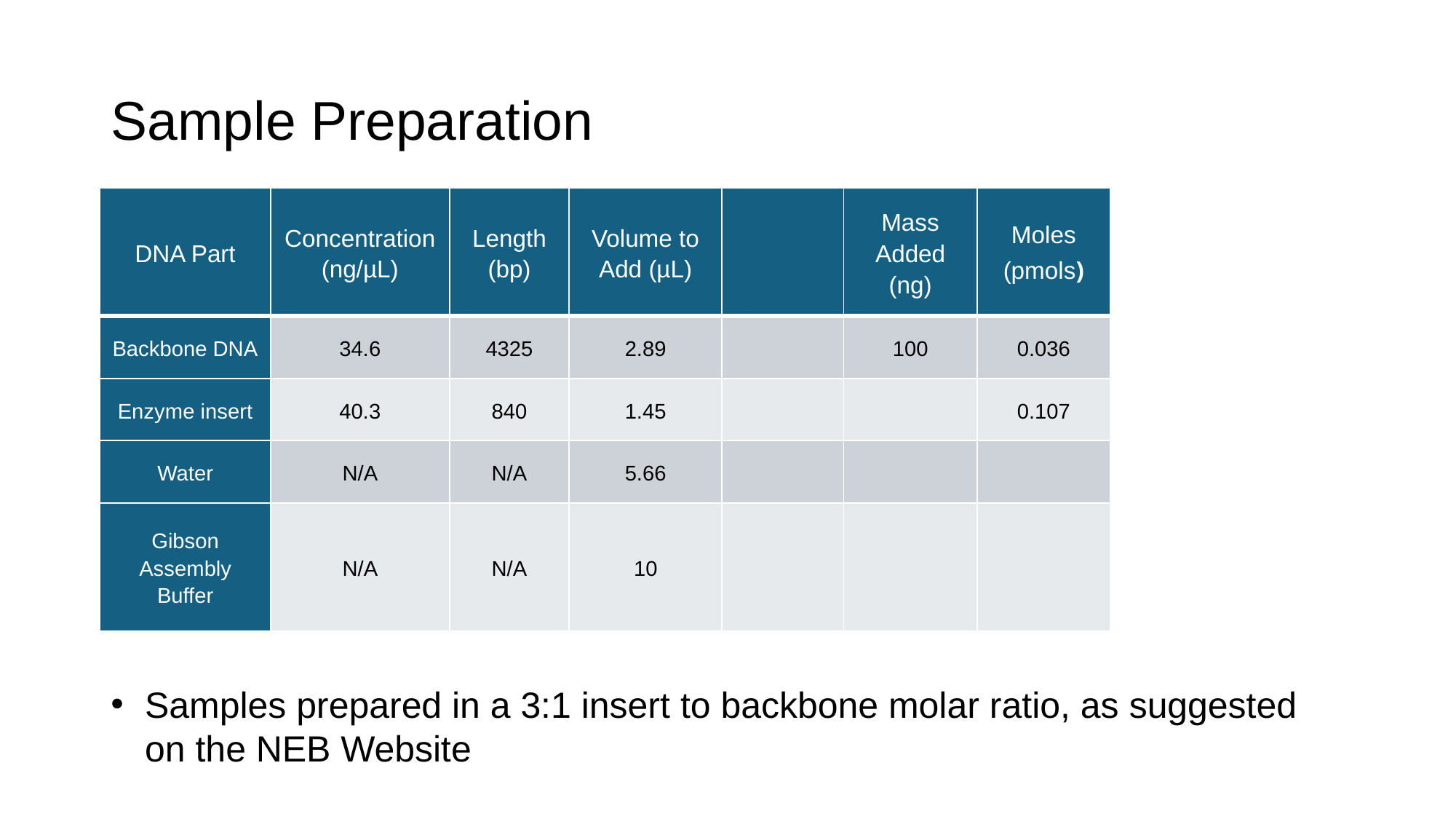

# Sample Preparation
| DNA Part | Concentration (ng/µL) | Length (bp) | Volume to Add (µL) | | Mass Added (ng) | Moles (pmols) |
| --- | --- | --- | --- | --- | --- | --- |
| Backbone DNA | 34.6 | 4325 | 2.89 | | 100 | 0.036 |
| Enzyme insert | 40.3 | 840 | 1.45 | | | 0.107 |
| Water | N/A | N/A | 5.66 | | | |
| Gibson Assembly Buffer | N/A | N/A | 10 | | | |
Samples prepared in a 3:1 insert to backbone molar ratio, as suggested on the NEB Website

## Slide 4
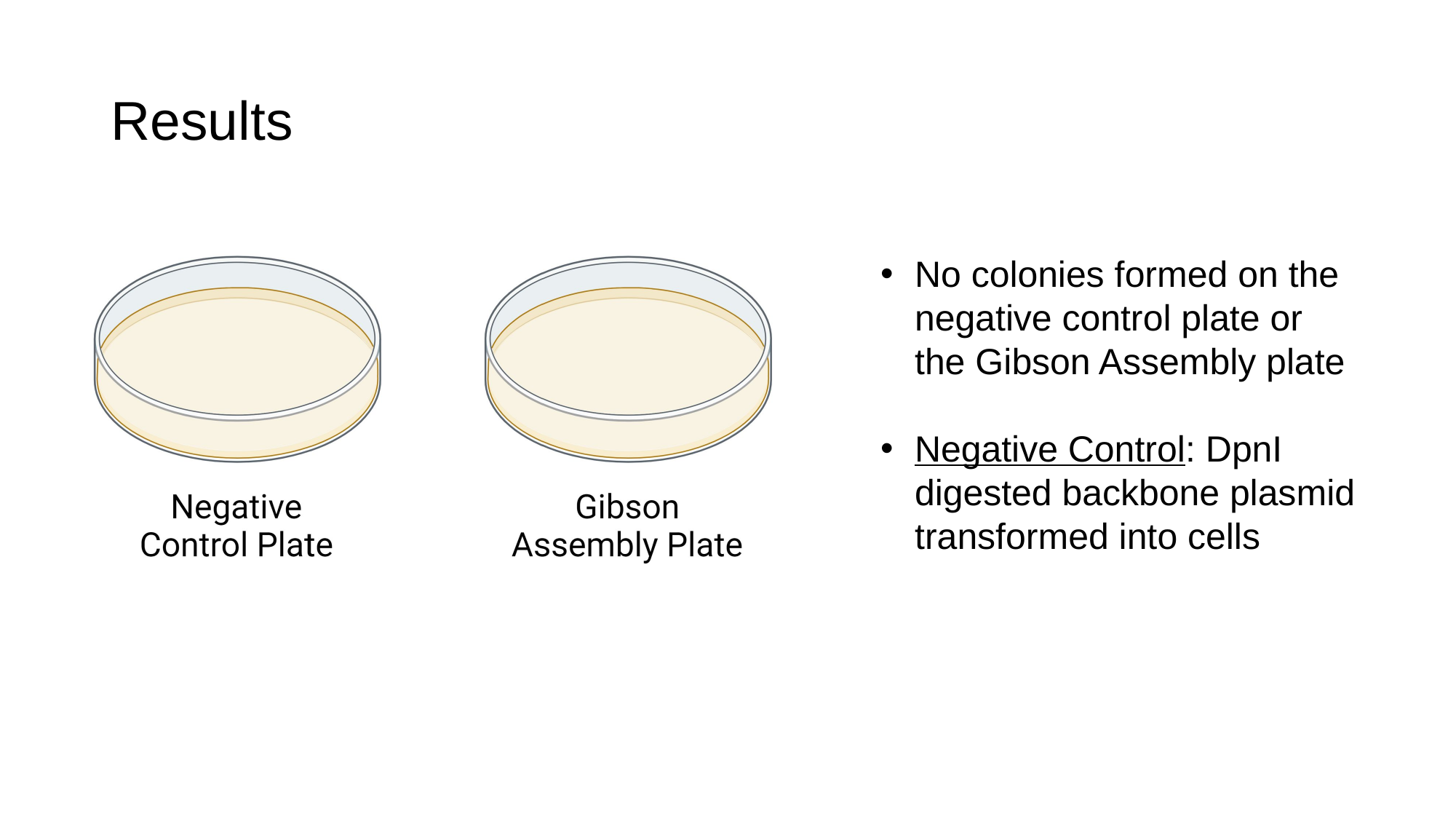

# Results
No colonies formed on the negative control plate or the Gibson Assembly plate
Negative Control: DpnI digested backbone plasmid transformed into cells

## Slide 5
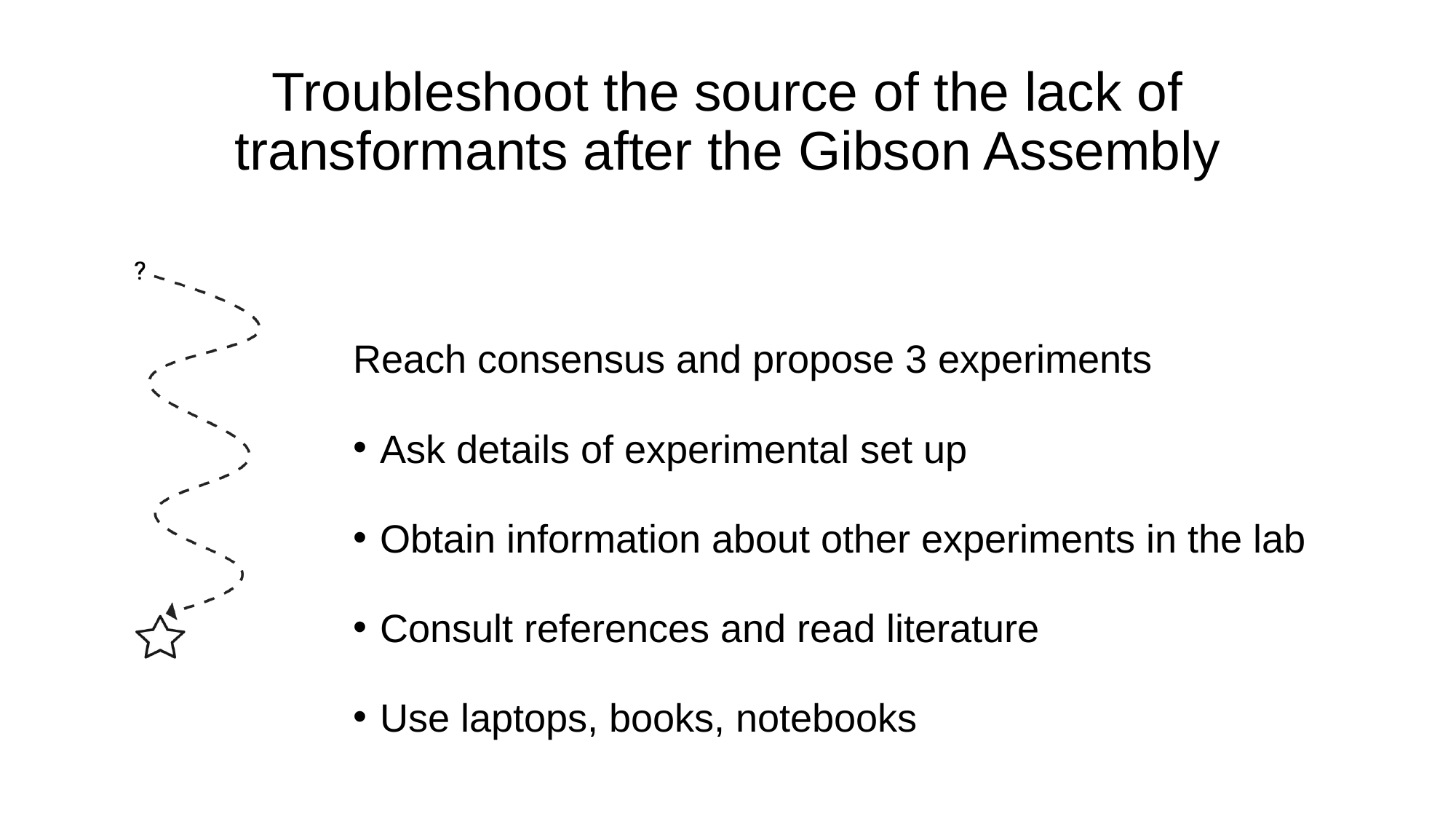

# Troubleshoot the source of the lack of transformants after the Gibson Assembly
Reach consensus and propose 3 experiments
Ask details of experimental set up
Obtain information about other experiments in the lab
Consult references and read literature
Use laptops, books, notebooks

## Slide 6
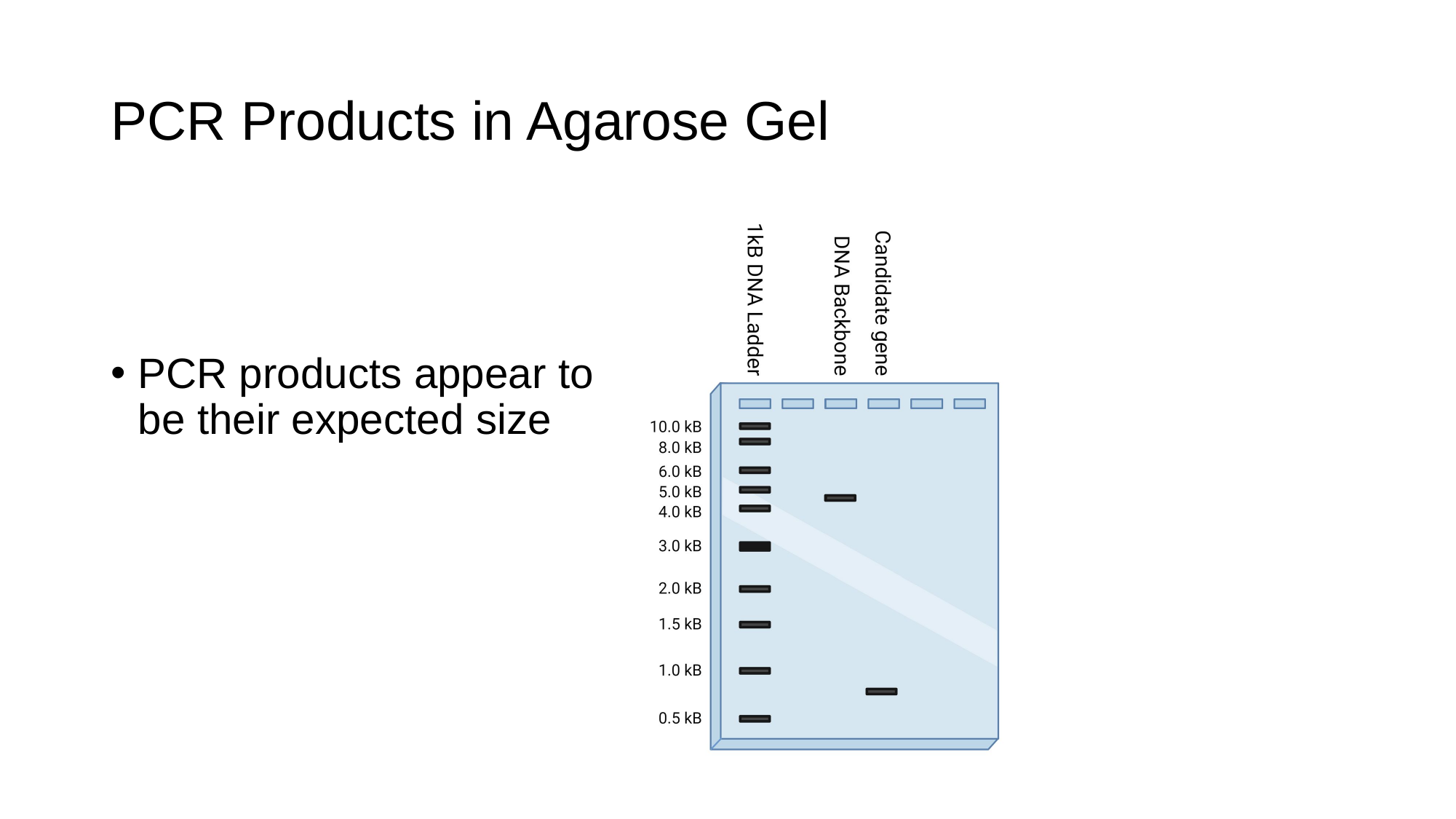

# PCR Products in Agarose Gel
PCR products appear to be their expected size

## Slide 7
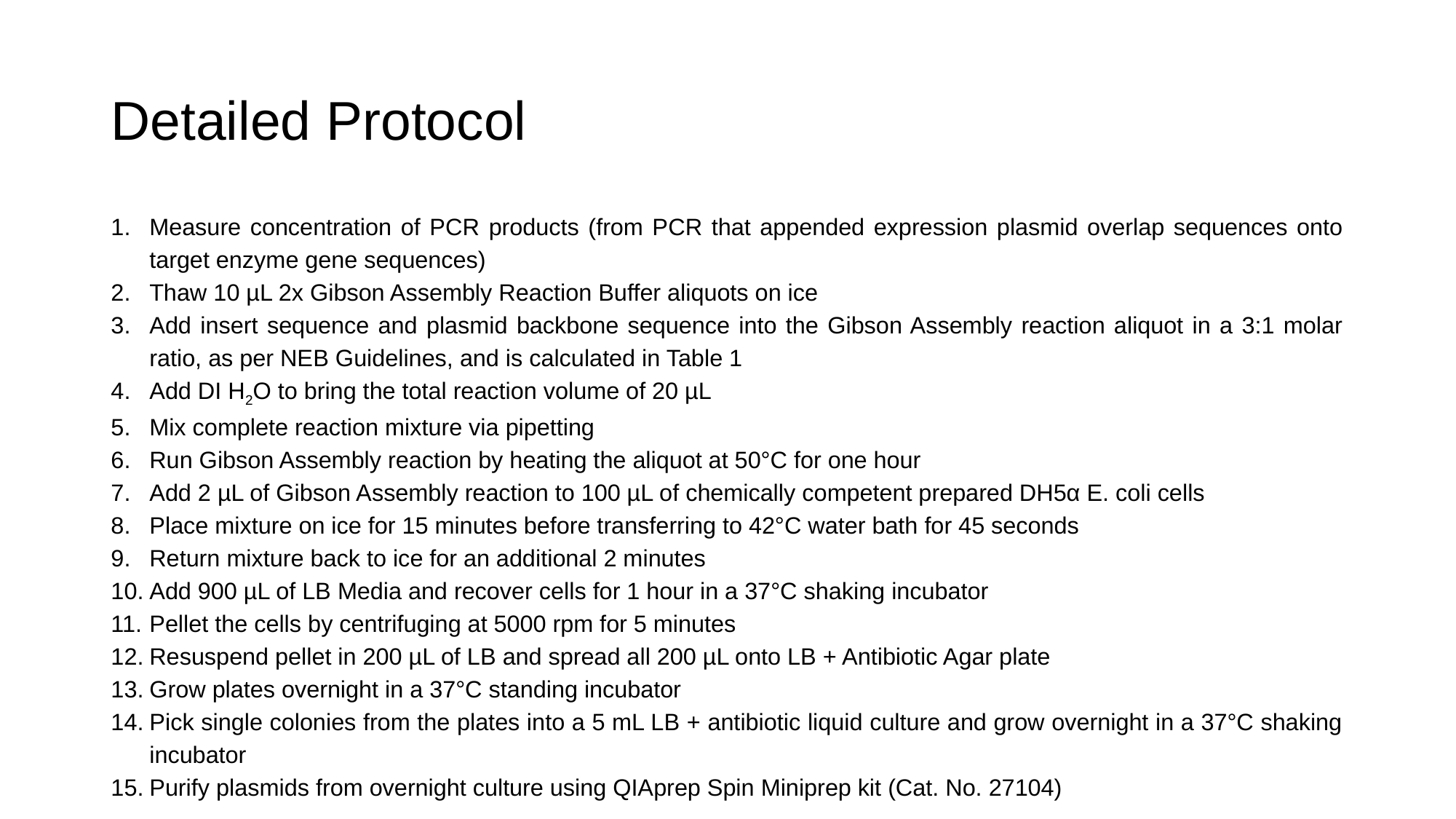

# Detailed Protocol
Measure concentration of PCR products (from PCR that appended expression plasmid overlap sequences onto target enzyme gene sequences)
Thaw 10 µL 2x Gibson Assembly Reaction Buffer aliquots on ice
Add insert sequence and plasmid backbone sequence into the Gibson Assembly reaction aliquot in a 3:1 molar ratio, as per NEB Guidelines, and is calculated in Table 1
Add DI H2O to bring the total reaction volume of 20 µL
Mix complete reaction mixture via pipetting
Run Gibson Assembly reaction by heating the aliquot at 50°C for one hour
Add 2 µL of Gibson Assembly reaction to 100 µL of chemically competent prepared DH5α E. coli cells
Place mixture on ice for 15 minutes before transferring to 42°C water bath for 45 seconds
Return mixture back to ice for an additional 2 minutes
Add 900 µL of LB Media and recover cells for 1 hour in a 37°C shaking incubator
Pellet the cells by centrifuging at 5000 rpm for 5 minutes
Resuspend pellet in 200 µL of LB and spread all 200 µL onto LB + Antibiotic Agar plate
Grow plates overnight in a 37°C standing incubator
Pick single colonies from the plates into a 5 mL LB + antibiotic liquid culture and grow overnight in a 37°C shaking incubator
Purify plasmids from overnight culture using QIAprep Spin Miniprep kit (Cat. No. 27104)

## Slide 8
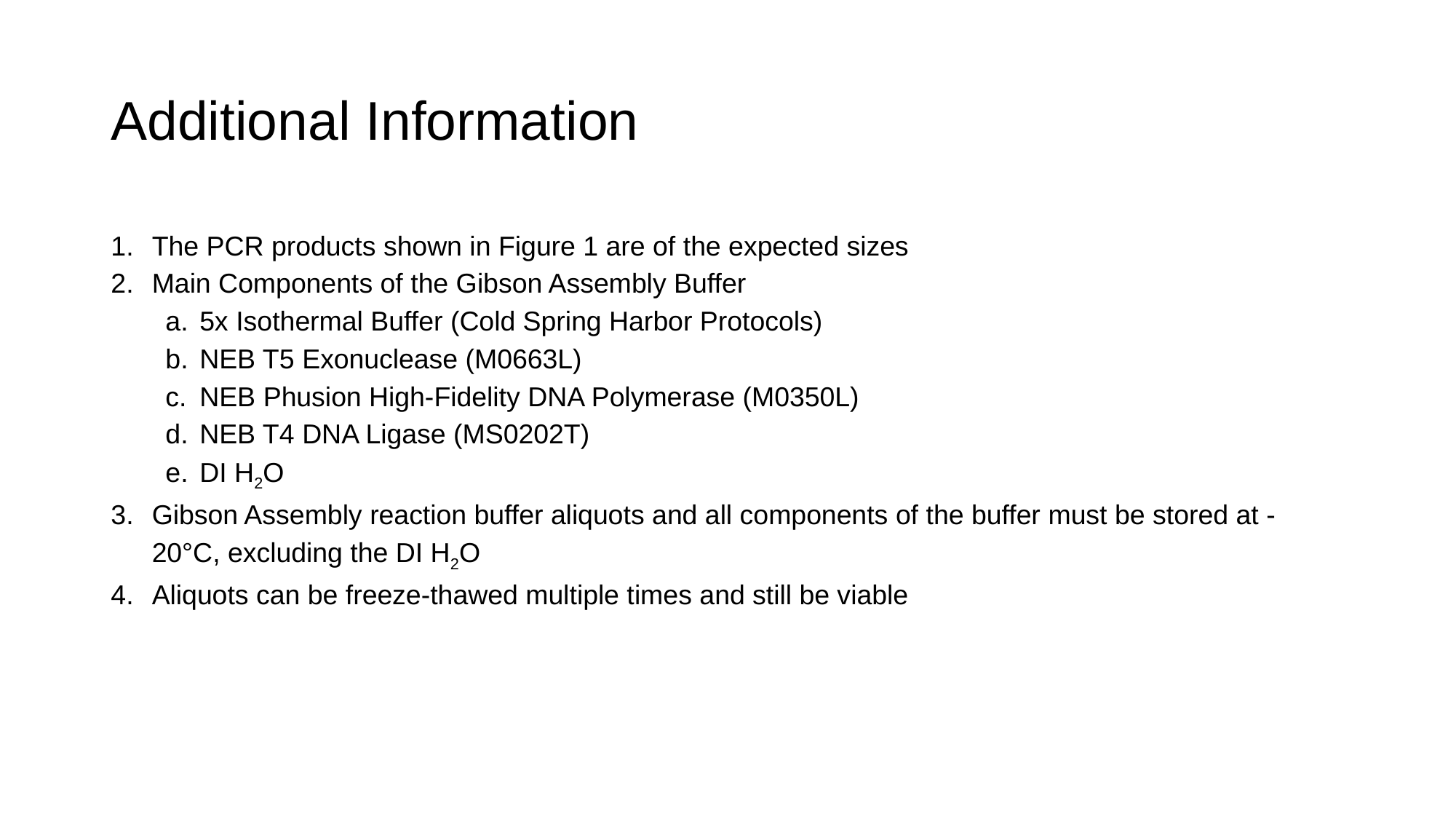

# Additional Information
The PCR products shown in Figure 1 are of the expected sizes
Main Components of the Gibson Assembly Buffer
5x Isothermal Buffer (Cold Spring Harbor Protocols)
NEB T5 Exonuclease (M0663L)
NEB Phusion High-Fidelity DNA Polymerase (M0350L)
NEB T4 DNA Ligase (MS0202T)
DI H2O
Gibson Assembly reaction buffer aliquots and all components of the buffer must be stored at -20°C, excluding the DI H2O
Aliquots can be freeze-thawed multiple times and still be viable

## Slide 9
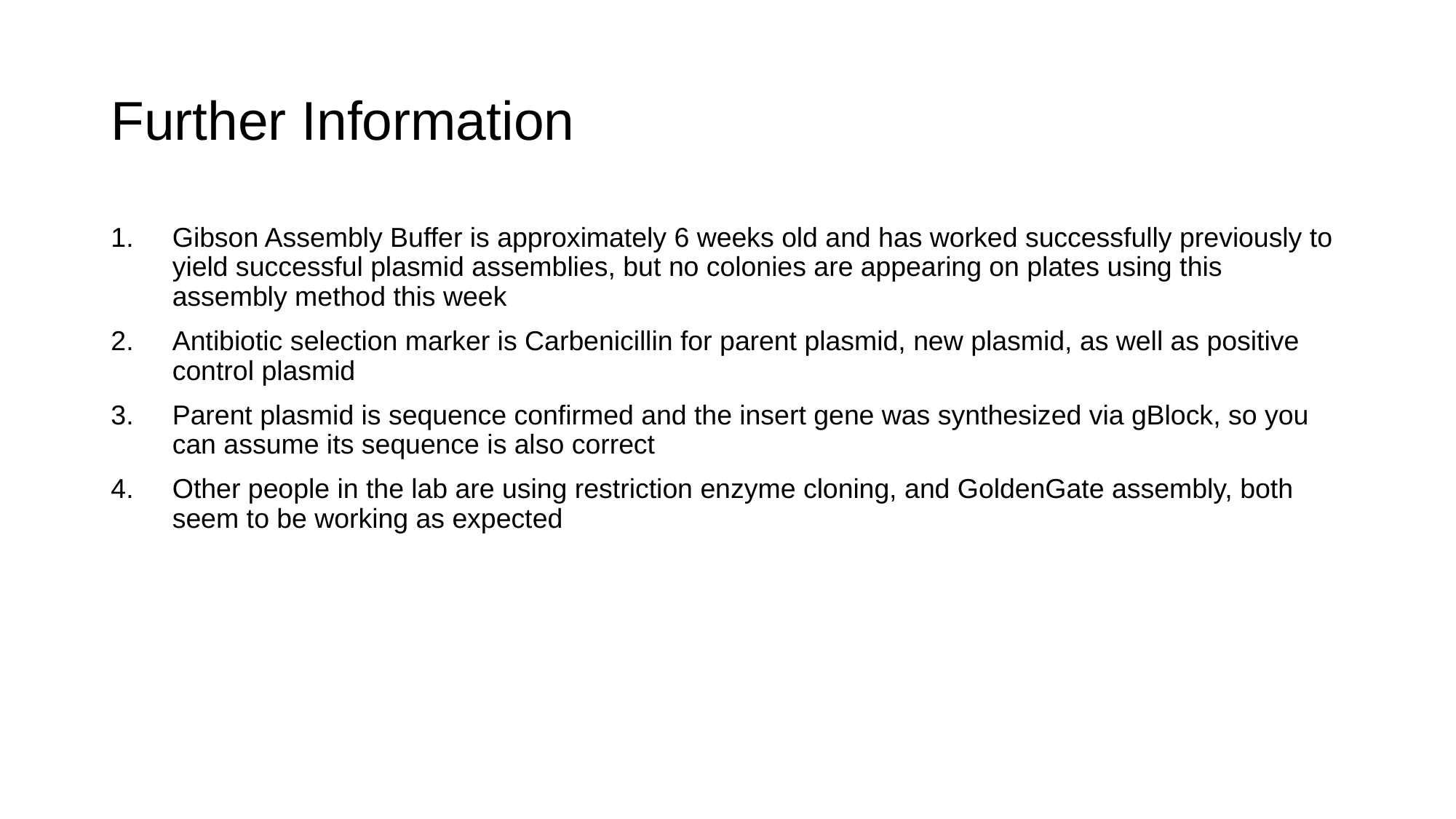

# Further Information
Gibson Assembly Buffer is approximately 6 weeks old and has worked successfully previously to yield successful plasmid assemblies, but no colonies are appearing on plates using this assembly method this week
Antibiotic selection marker is Carbenicillin for parent plasmid, new plasmid, as well as positive control plasmid
Parent plasmid is sequence confirmed and the insert gene was synthesized via gBlock, so you can assume its sequence is also correct
Other people in the lab are using restriction enzyme cloning, and GoldenGate assembly, both seem to be working as expected

## Slide 10
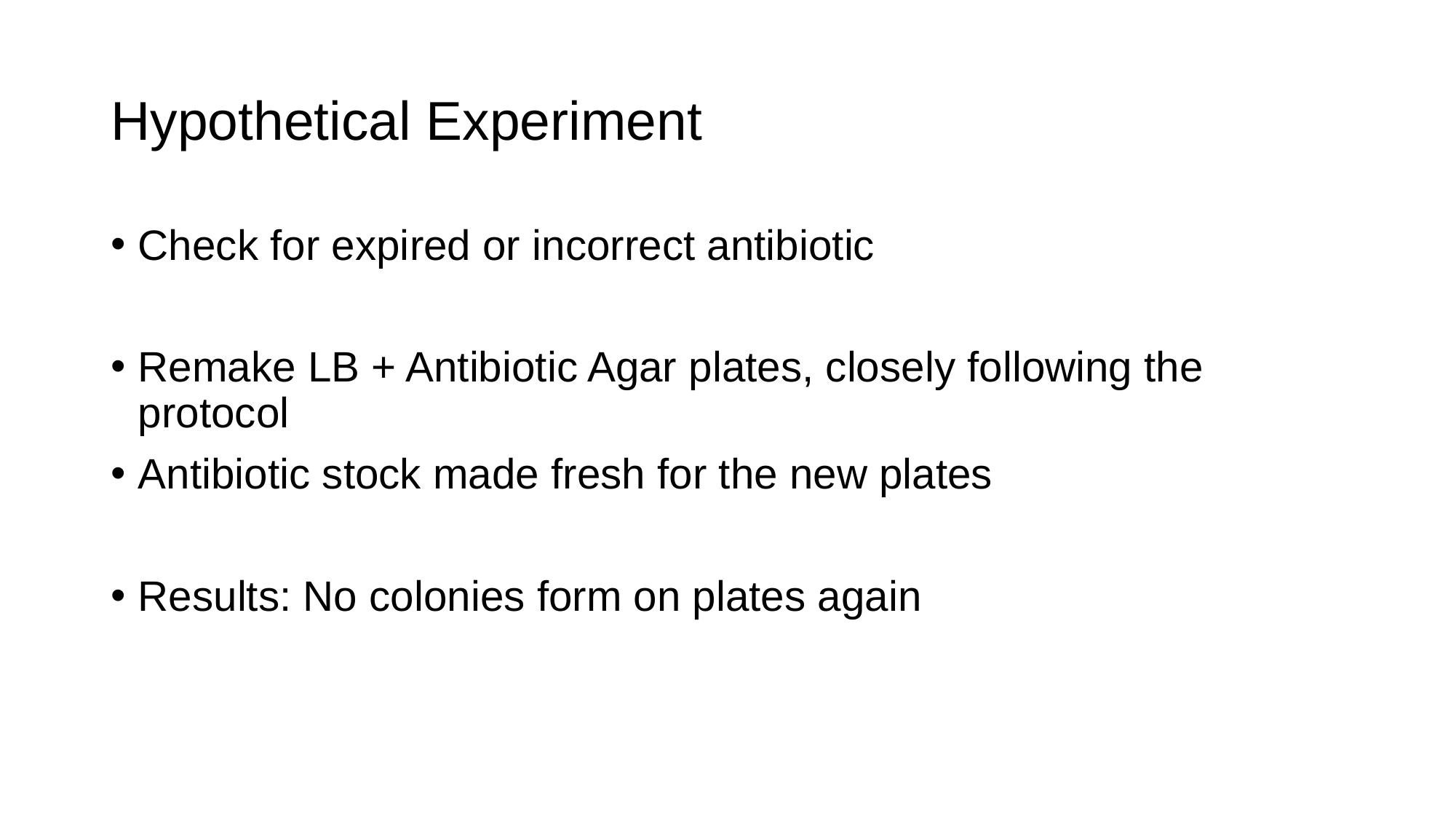

# Hypothetical Experiment
Check for expired or incorrect antibiotic
Remake LB + Antibiotic Agar plates, closely following the protocol
Antibiotic stock made fresh for the new plates
Results: No colonies form on plates again

## Slide 11
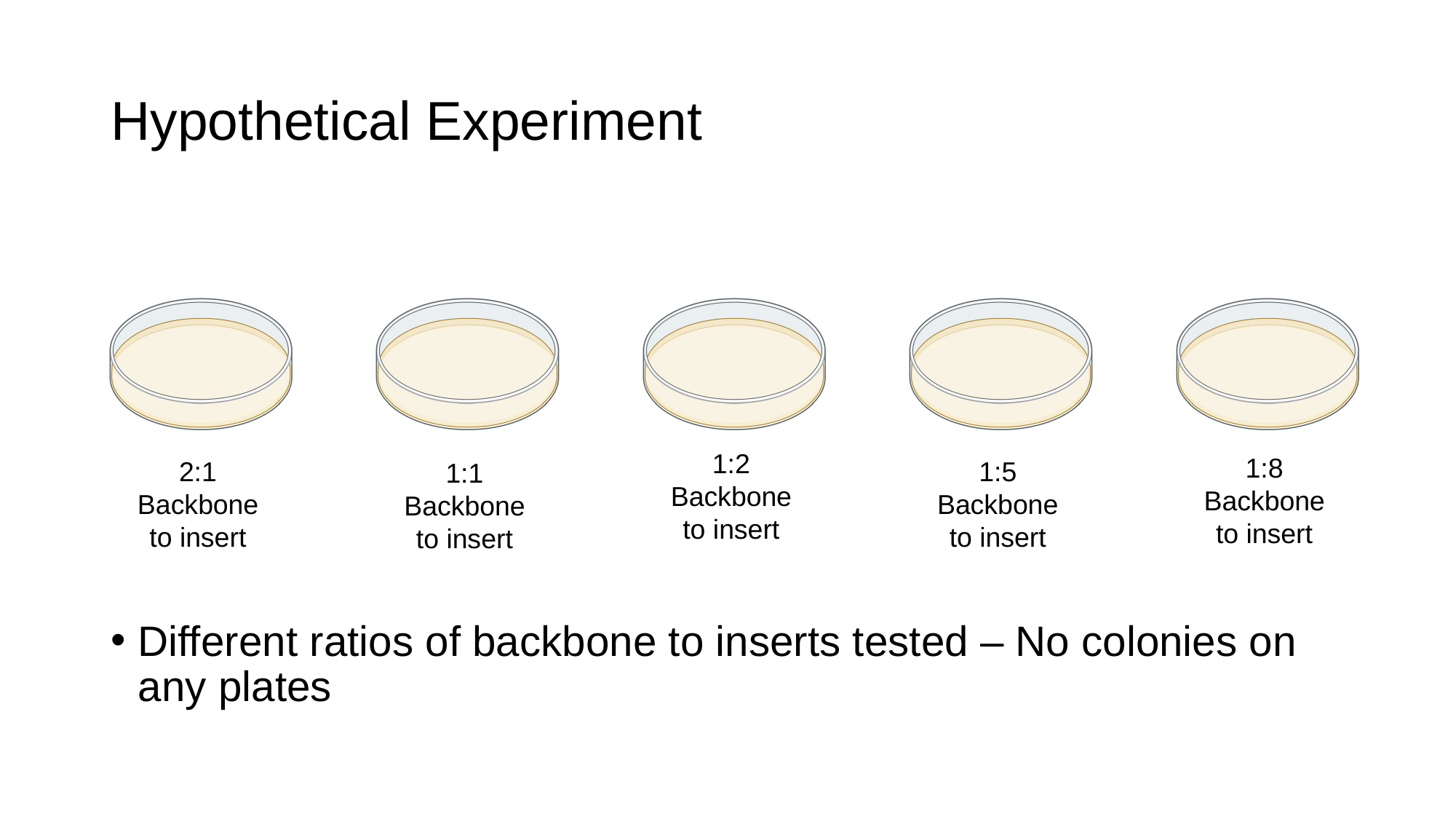

# Hypothetical Experiment
1:2
Backbone to insert
1:8
Backbone to insert
2:1
Backbone to insert
1:5
Backbone to insert
1:1
Backbone to insert
Different ratios of backbone to inserts tested – No colonies on any plates

## Slide 12
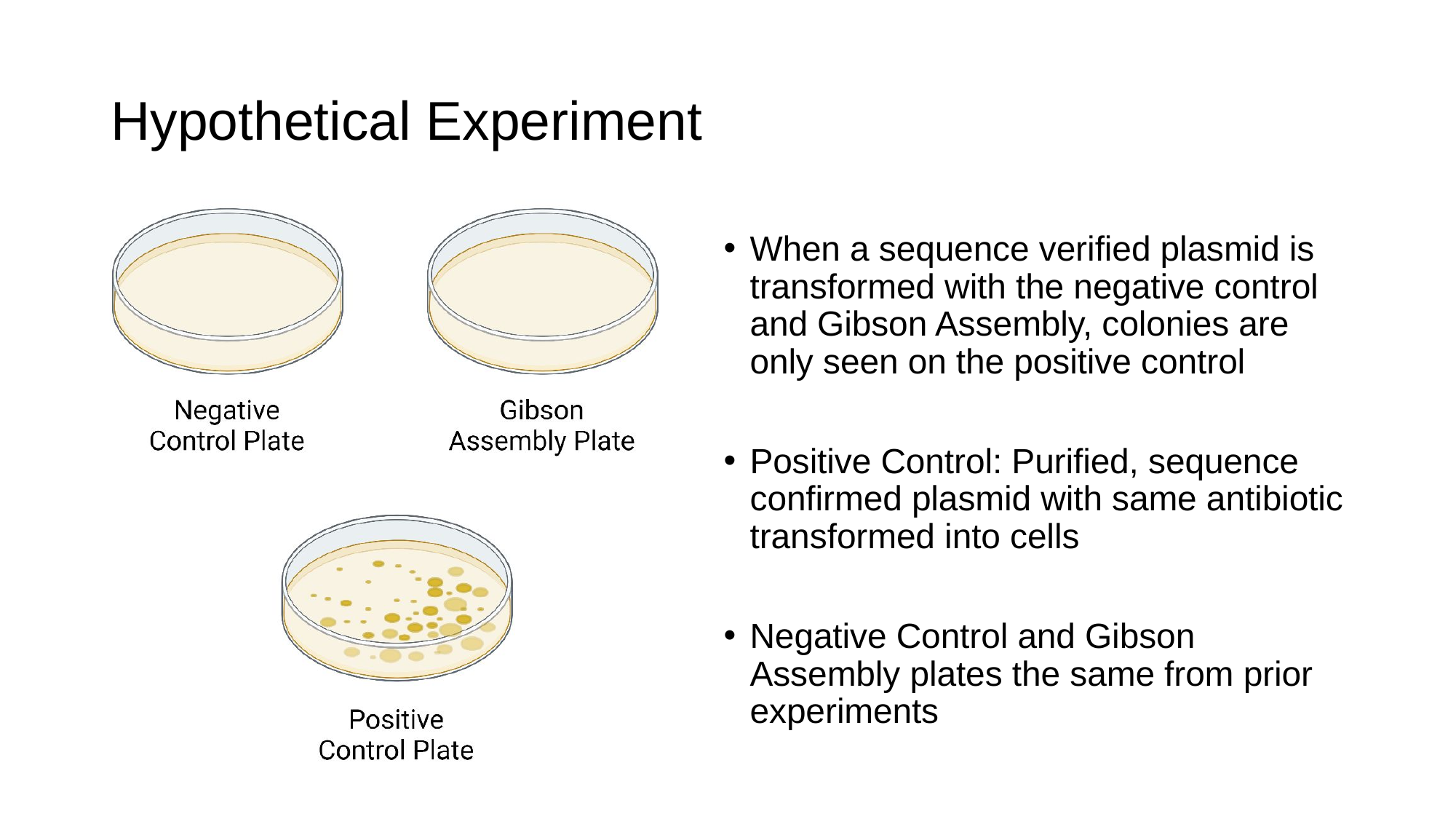

# Hypothetical Experiment
When a sequence verified plasmid is transformed with the negative control and Gibson Assembly, colonies are only seen on the positive control
Positive Control: Purified, sequence confirmed plasmid with same antibiotic transformed into cells
Negative Control and Gibson Assembly plates the same from prior experiments

## Slide 13
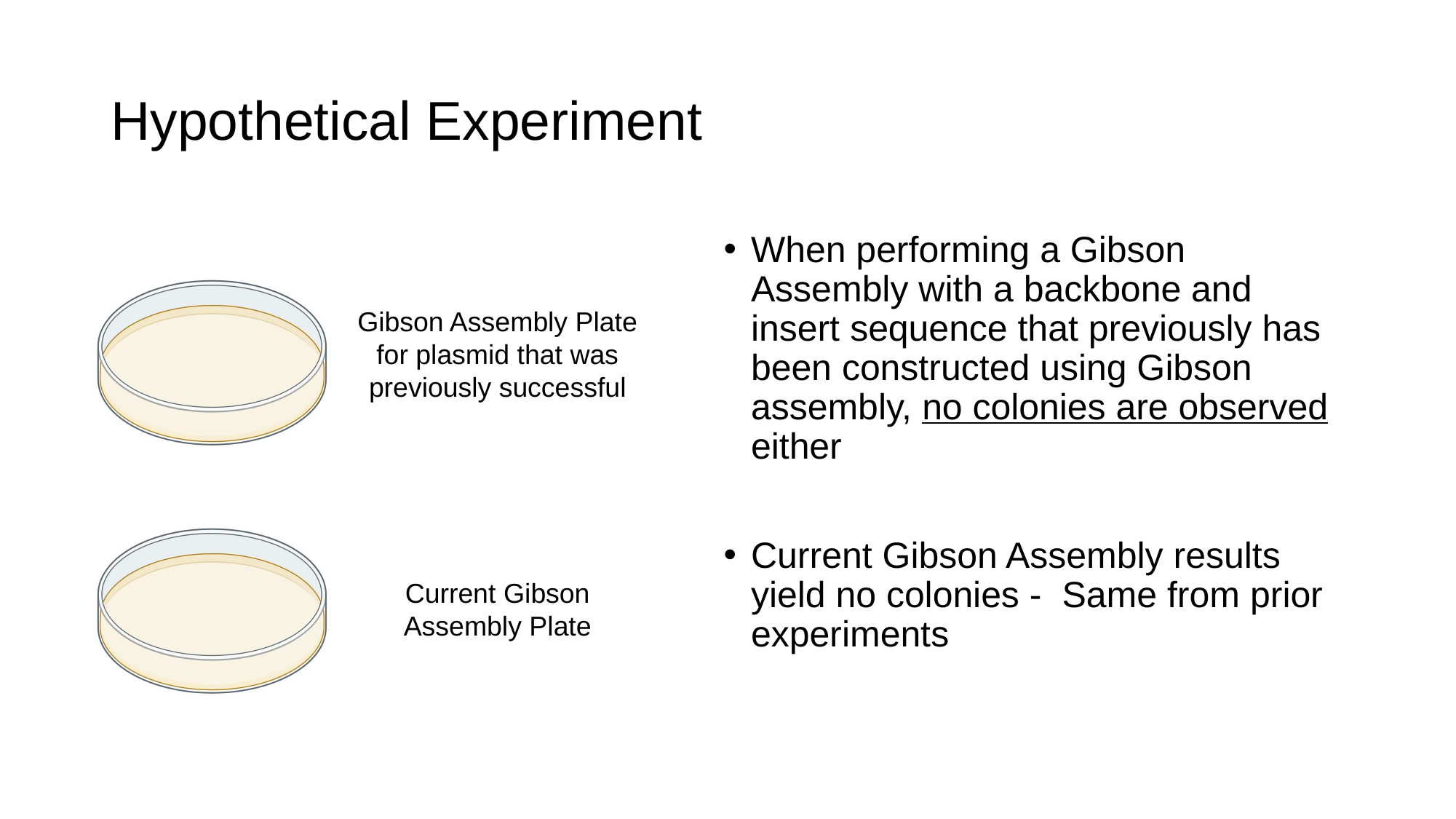

# Hypothetical Experiment
When performing a Gibson Assembly with a backbone and insert sequence that previously has been constructed using Gibson assembly, no colonies are observed either
Current Gibson Assembly results yield no colonies - Same from prior experiments
Gibson Assembly Plate for plasmid that was previously successful
Current Gibson Assembly Plate

## Slide 14
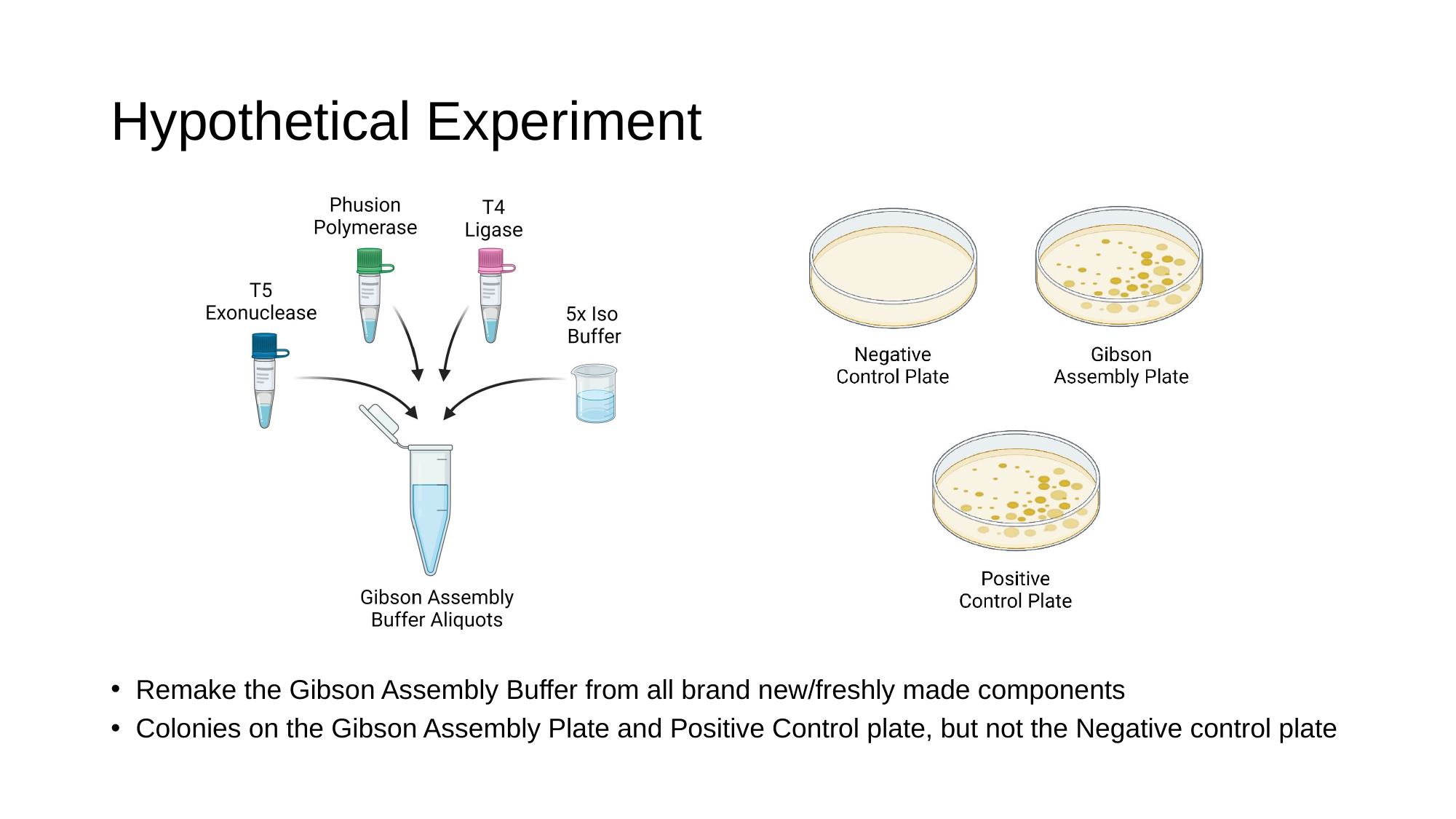

# Hypothetical Experiment
Remake the Gibson Assembly Buffer from all brand new/freshly made components
Colonies on the Gibson Assembly Plate and Positive Control plate, but not the Negative control plate

## Slide 15
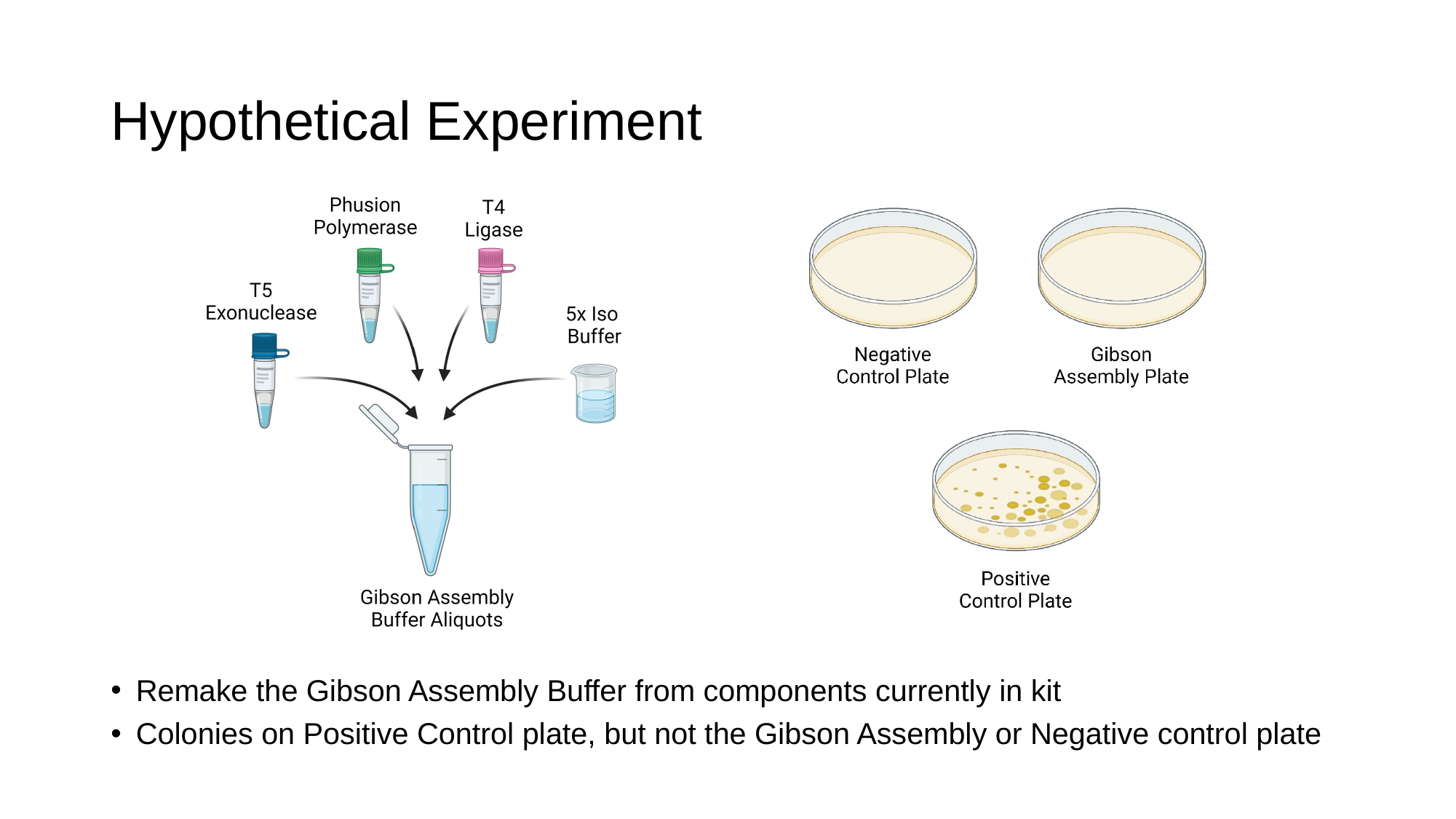

# Hypothetical Experiment
Remake the Gibson Assembly Buffer from components currently in kit
Colonies on Positive Control plate, but not the Gibson Assembly or Negative control plate

## Slide 16
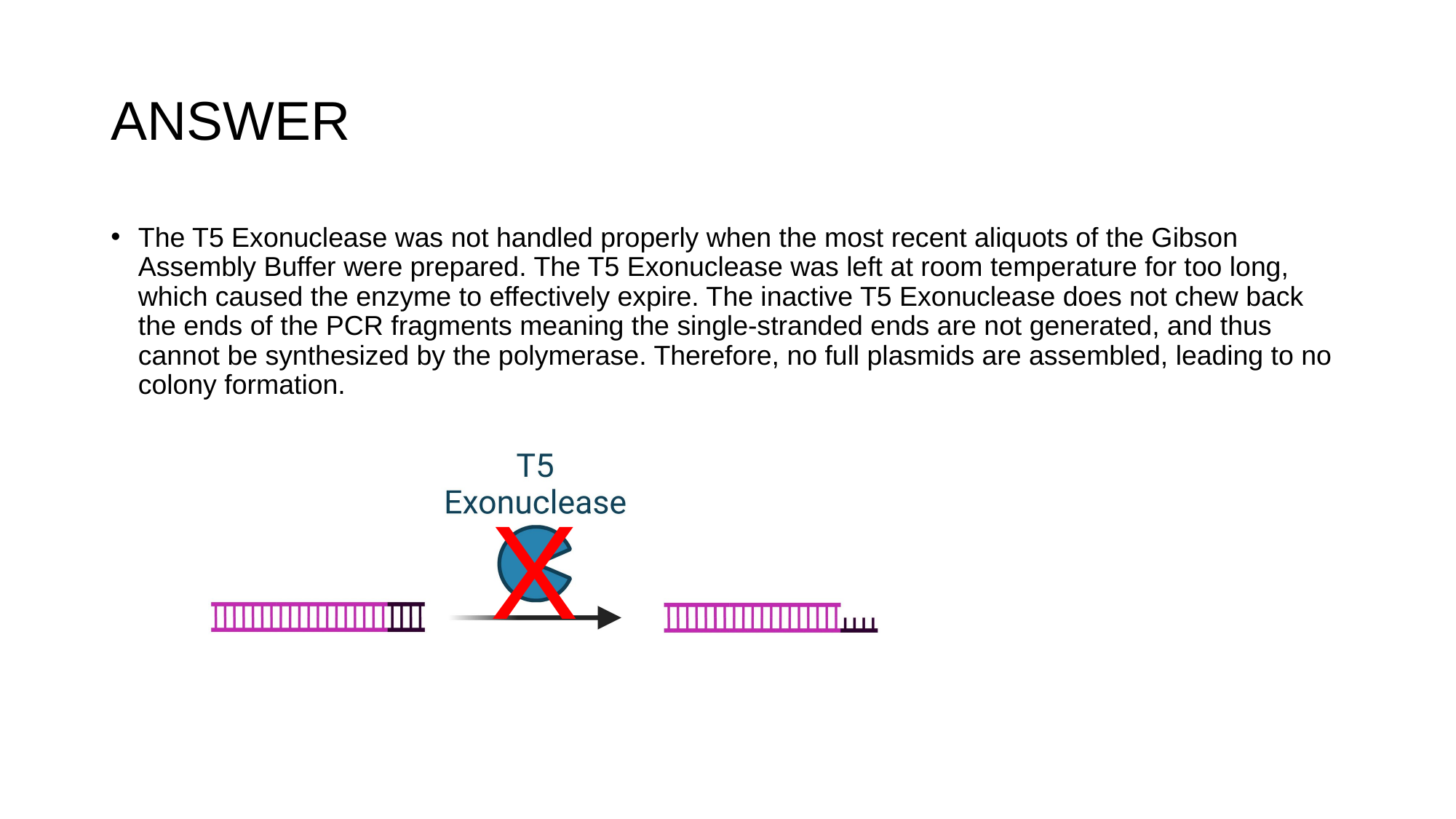

# ANSWER
The T5 Exonuclease was not handled properly when the most recent aliquots of the Gibson Assembly Buffer were prepared. The T5 Exonuclease was left at room temperature for too long, which caused the enzyme to effectively expire. The inactive T5 Exonuclease does not chew back the ends of the PCR fragments meaning the single-stranded ends are not generated, and thus cannot be synthesized by the polymerase. Therefore, no full plasmids are assembled, leading to no colony formation.
X
