# Supplementary material for: Teaching troubleshooting skills to graduate students
Source: eLife. 2024 Sep 17;13:e100761. doi: 10.7554/eLife.100761 (PMC11407763; doi:10.7554/eLife.100761)
Supplement: Supplementary file 1. — For each scenario there is a Word file that contains the following: background information; a description of the scenario; the protocol for the experiment that produced the unexpected result; the results of the experiment; information on the source of the error; background information that can be used to answer questions; and references. There is also a PowerPoint file for each scenario that contains example slides that can be used in real meetings. There are also templates for the Word and PowerPoint files. [file elife-100761-supp1.zip › Final Scenarios/Example.template.docx]

**Background:**

Chose a protocol that is run semi-often in your own group or that has similar guiding principles and analytical techniques used by your group.

**Scenario:**

You are a researcher interested in________________. To do this you have decided to ____________. Unfortunately, _________________.

**Protocol:**

Write out a protocol to be provided to the group and to answer questions regarding the set up.

**Sample list:**

**Example graphs and experimental outcomes:**

**Details of specific set up:**

1. Detail 1
2. Detail 2

**Source of error:**

Detail the source of error for the group as well as for yourself. The group will propose experiments and based on the source of the error, you will infer simulated results. It is helpful to prepare 2-3 plausible experiments a head of time and sketch out results. Other experiments proposed by the group during the meeting will require you to sketch out the results on a white board or paper in real time. Having a thorough understanding of the error will help you to produce these simulated results quickly.

**Table 1.** Additional information known by the leader that can be provided upon request

| **Meeting Notes for the Leader**  Not to be shared with the group | |
| --- | --- |
| Other researcher’s experiments | - Include information about the other experiments being run in the same lab space or by the same researchers - Experiments do not necessarily have to deal with similar topics as the proposed scenario - Experiments may or may not include relevant information - The provided “Other researchers” experiments are meant to be shared upon request and simulate a healthy-lab communication network. - Keep this information, but do not provide it until asked. |
| Storage information | - Simulate information about the storing of potentially volatile or degradable materials. - Keep this information, but do not provide it until asked |
| Sample information | - Simulate information regarding the specifics of the sample. |
| Source of error | - Keep this information close to reference in case it is required to answer new proposed experiments |
| Hints for group | - Keep one to two hints that will help the group in case they get stuck. - An ideal scenario should take 45-60 minutes - Encourage the group to look up information, read papers, read protocols |

Tips and tricks for leading the meeting:

- Encourage the group to talk, and foster an inclusive environment
- Solicit group opinions
- Ask members of the group to identify what an ideal outcome would look like
  - Encourage the group to identify the similarities and differences are between the proposed scenario
- Give preference to the least experienced trainees and allow them to speak first, and encourage more senior scientists hold their thoughts until the group reaches a lull
- Take care to remember what types of information a researcher knows when they set up an experiment and redirect group members to propose experiments to test probing
