# Supplementary material for: Teaching troubleshooting skills to graduate students
Source: eLife. 2024 Sep 17;13:e100761. doi: 10.7554/eLife.100761 (PMC11407763; doi:10.7554/eLife.100761)
Supplement: Supplementary file 1. — For each scenario there is a Word file that contains the following: background information; a description of the scenario; the protocol for the experiment that produced the unexpected result; the results of the experiment; information on the source of the error; background information that can be used to answer questions; and references. There is also a PowerPoint file for each scenario that contains example slides that can be used in real meetings. There are also templates for the Word and PowerPoint files. [file elife-100761-supp1.zip › Final Scenarios/Example1.docx]

**MTT-assay**

Keywords: Mammalian cell biology, cytotoxicity, analytical chemistry

**Background:**

An MTT assay utilizes the reduction of a tetrazolium salt to purple formazan crystals to measure cell metabolic activity^1^. It is an indirect measure of cell viability but is commonly used to determine cell viability. The MTT Assay is commercially available from Sigma Aldrich (Product number 1146500701). Broadly the assay involves incubating the cells with the tetrazolium salt for some length of time, and then adding in a solvent such as DMSO or sodium dodecyl sulfate/HCl^2^ to solubilize the formazan crystals before taking absorbance readings. The MTT cleavage follows first order kinetics and can be fitted with Michaelis kinetics^3^ although oftentimes end point readings are taken.

The protein concentration in serum can interfere with MTT by creating formazan crystal aggregates. For this reason, serum is often omitted from the reactions^2^.

**Scenario:**

You are a researcher interested in examining the cytotoxicity of a purified protein aggregate on a human neuroblastoma cell line SH-SY5Y. To do this you have decided to run an MTT assay as described below. Unfortunately, the error bars of your experiment are incredibly large, and you are seeing viability in your control sample as low as 45% and as high as 186%.

**Protocol:**

1. Passage SH-SY5Y until passage 6-10
2. In a 96-well plate, plate 200µL for a total of 40,000 cells/well in a 1:1 mixture of F12:EMEM in 10% FBS and let adhere overnight.
3. Next morning prep your protein aggregate in a concentrated solution.
4. Check the SH-SY5Y cells on microscope for adherence and then spin down 96 well plate at 500 xg for 5 minutes.
5. Aspirate supernatant using autoclaved Pasteur pipette connected to a vacuum inside a BSC.
6. Wash with 200µL 1X PBS and spin down 96 well plate at 500 xg for 5 min.
7. Resuspend the protein aggregate in DMEM without phenol red indicator and without FBS.
8. Add 190µL of aggregate suspension to sample wells, in triplicate. Additionally, add 190µL of DMEM without phenol red and without FBS to control wells.
9. Let sit for 24 hours.
10. After 24 hour incubation, spin down 96 well plate cells at 500 xg for 5 min.
11. Aspirate the supernatant using Pasteur pipette and then add 100µL of DMEM without phenol red without FBS along with 10µL of MTT solution (prepared at a stock of 5mg/mL in PBS).
12. Let MTT incubate with cells for 4 hours.
13. After 4 hour incubation, spin cells down at 500 xg for 5 min, and aspirate supernatant.
14. Then, add 100µL of DMSO to each well, pipetting up and down vigorously to solubilize formazan.
15. Place in 37^o^C incubator for 5 minutes
16. Analyze the absorbance at 570 nm.

**Table 1.** Sample layout

| A1 | 2 | 3 | 4 | 5 | 6 | 7 | 8 | 9 | 10 | 11 | 12 |
| --- | --- | --- | --- | --- | --- | --- | --- | --- | --- | --- | --- |
| B | Sample 1 | Sample 2 | Sample 3 | Sample 4 | Sample 5 | Sample 6 |  |  |  |  |  |
| C | Sample 1 | Sample 2 | Sample 3 | Sample 4 | Sample 5 | Sample 6 |  |  |  |  |  |
| D | Sample 1 | Sample 2 | Sample 3 | Sample 4 | Sample 5 | Sample 6 |  |  |  |  |  |
| E | Sample 1 | Sample 2 | Sample 3 | Sample 4 | Sample 5 | Sample 6 |  |  |  |  |  |
| F | Sample 1 | Sample 2 | Sample 3 | Sample 4 | Sample 5 | Sample 6 |  |  |  |  |  |
| G | Sample 1 | Sample 2 | Sample 3 | Sample 4 | Sample 5 | Sample 6 |  |  |  |  |  |
| H |  |  |  |  |  |  |  |  |  |  |  |

**Sample list**

1. DMEM blank without cells or protein aggregate
2. SH-SY5Y control cells without protein aggregate
3. SH-SY5Y cells with high concentration of aggregate
4. SH-SY5Y cells with medium concentration of aggregate
5. SH-SY5Y cells with low concentration of aggregate
6. Cell-free control with high concentration of aggregate

**Example graphs and experimental outcomes**

**Table 2.** Absorbance at 570 nm

| A1 | 2 | 3 | 4 | 5 | 6 | 7 | 8 | 9 | 10 | 11 | 12 |
| --- | --- | --- | --- | --- | --- | --- | --- | --- | --- | --- | --- |
| B | 0.009 | 0.089 | 0.023 | 0.147 | 0.402 | 0.020 |  |  |  |  |  |
| C | 0.010 | 0.248 | 0.093 | 0.245 | 0.310 | 0.019 |  |  |  |  |  |
| D | 0.014 | 0.157 | 0.145 | 0.233 | 0.182 | 0.012 |  |  |  |  |  |
| E | 0.089 | 0.149 | 0.124 | 0.293 | 0.123 | 0.010 |  |  |  |  |  |
| F | 0.009 | 0.300 | 0.240 | 0.251 | 0.255 | 0.008 |  |  |  |  |  |
| G | 0.010 | 0.088 | 0.197 | 0.433 | 0.243 | 0.024 |  |  |  |  |  |
| H |  |  |  |  |  |  |  |  |  |  |  |

This hypothetical data was generated and is not a reflection of a real experiment. It should not be cited, used, or interpreted in anyway. It is solely for a training exercise.

**Figure 1.** Example viability data from hypothetical experiment. Data represents

Abs_sample_ /Average(Abs_Sample2_) * 100%

**Details:**

1. MTT is stable in PBS in fridge for 1 month. This particular aliquot appeared to be new; however, it was undated.
2. The cells used were passaged to passage 7 before use

**Source of error:**

When aspirating off the supernatant in step 11, if the tip touches the bottom of the well plate. In doing so, the delicate cells are aspirated with the supernatant creating inconsistent cell densities throughout the experiment. This creates inconsistencies with the entirety of the experiment because essentially each well was run at a different density.

**Table 1.** Additional information known by the leader that can be provided upon request

| **Meeting Notes for the Leader**  Not to be shared with the group | |
| --- | --- |
| Other researcher’s experiments | - Other researchers are sharing the biosafety cabinets - The SH-SY5Y cells are not being cultured by anyone else - There was a bacterial infection in an incubator one lab space over - Another researcher ran an MTT assay 2 months ago, and had smaller error bars, and more reasonable results |
| Storage information | - The media F12 and EMEM are 2 weeks old, and freshly aliquoted - The MTT appears new but was unlabelled - The PBS was opened fresh before the experiment |
| Protein information | - The concentration of the protein aggregate was determined via Bradford assay - The protein is expressed and purified from a recombinant strain of *E. coli* |
| Source of error | - When aspirating off the supernatant in step 11, if the tip touches the bottom of the well plate. The delicate cells are aspirated with the supernatant creating inconsistent cell densities throughout the experiment. |
| Hints for group | - Other students in the lab have performed MTT experiments previously - The student performing the experiment has limited experiment culturing dual adherent/non-adherent cell lines, and minimal experience with cell culture in general - There are plate readers, microscopes, NMRs, and multiple HPLCs within the lab |

**References**

(1) Mosmann, T. Rapid Colorimetric Assay for Cellular Growth and Survival: Application to Proliferation and Cytotoxicity Assays. *J. Immunol. Methods* **1983**, *65* (1–2), 55–63. https://doi.org/10.1016/0022-1759(83)90303-4.

(2) Tada, H.; Shiho, O.; Kuroshima, K.; Koyama, M.; Tsukamoto, K. An Improved Colorimetric Assay for Interleukin 2. *J. Immunol. Methods* **1986**, *93* (2), 157–165. https://doi.org/10.1016/0022-1759(86)90183-3.

(3) Gerlier, D.; Thomasset, N. Use of MTT Colorimetric Assay to Measure Cell Activation. *J. Immunol. Methods* **1986**, *94* (1–2), 57–63. https://doi.org/10.1016/0022-1759(86)90215-2.
