# Supplementary material for: Teaching troubleshooting skills to graduate students
Source: eLife. 2024 Sep 17;13:e100761. doi: 10.7554/eLife.100761 (PMC11407763; doi:10.7554/eLife.100761)
Supplement: Supplementary file 1. — For each scenario there is a Word file that contains the following: background information; a description of the scenario; the protocol for the experiment that produced the unexpected result; the results of the experiment; information on the source of the error; background information that can be used to answer questions; and references. There is also a PowerPoint file for each scenario that contains example slides that can be used in real meetings. There are also templates for the Word and PowerPoint files. [file elife-100761-supp1.zip › Final Scenarios/Example7.pptx]

## Slide 1
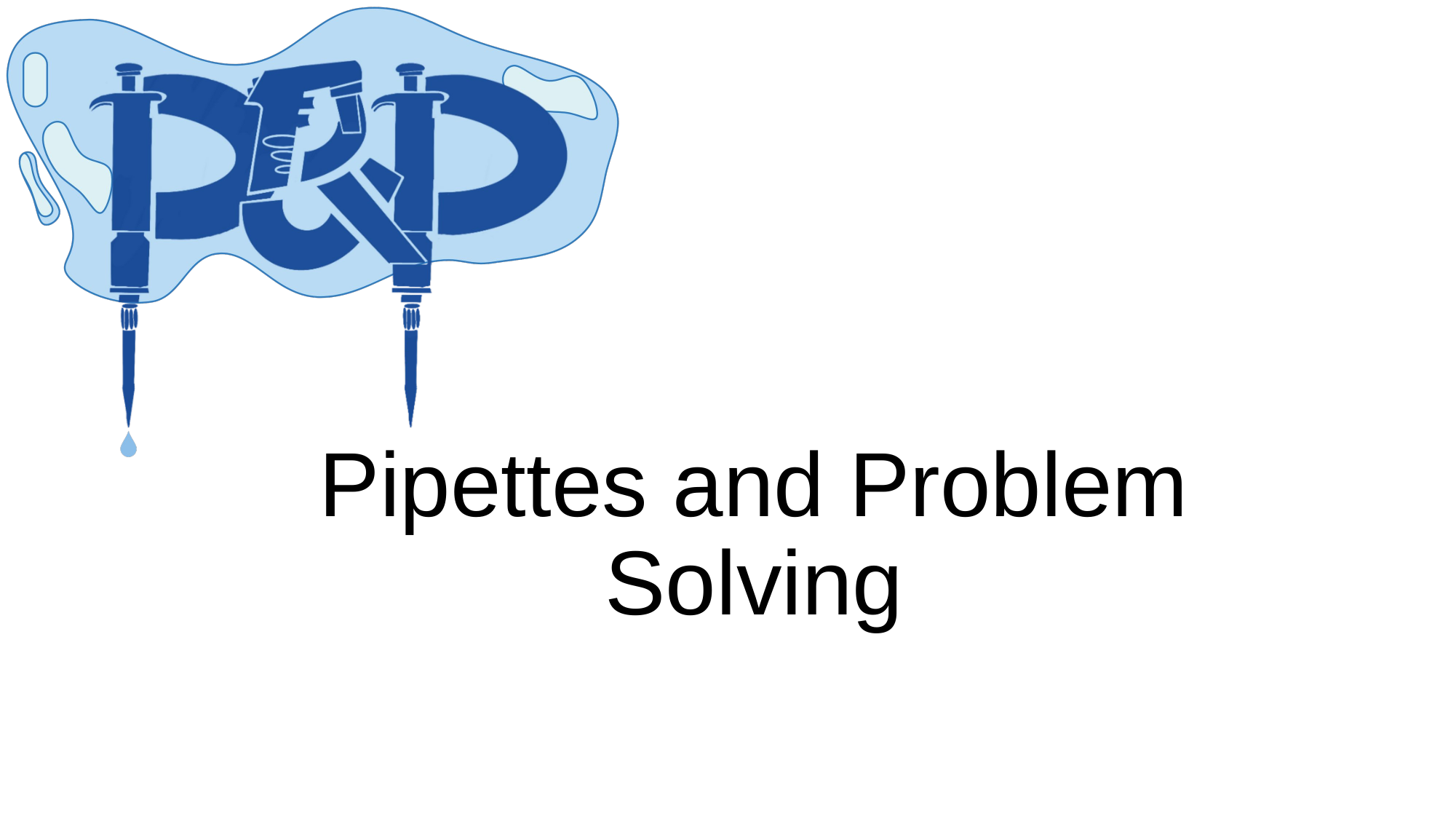

# Pipettes and Problem Solving

## Slide 2
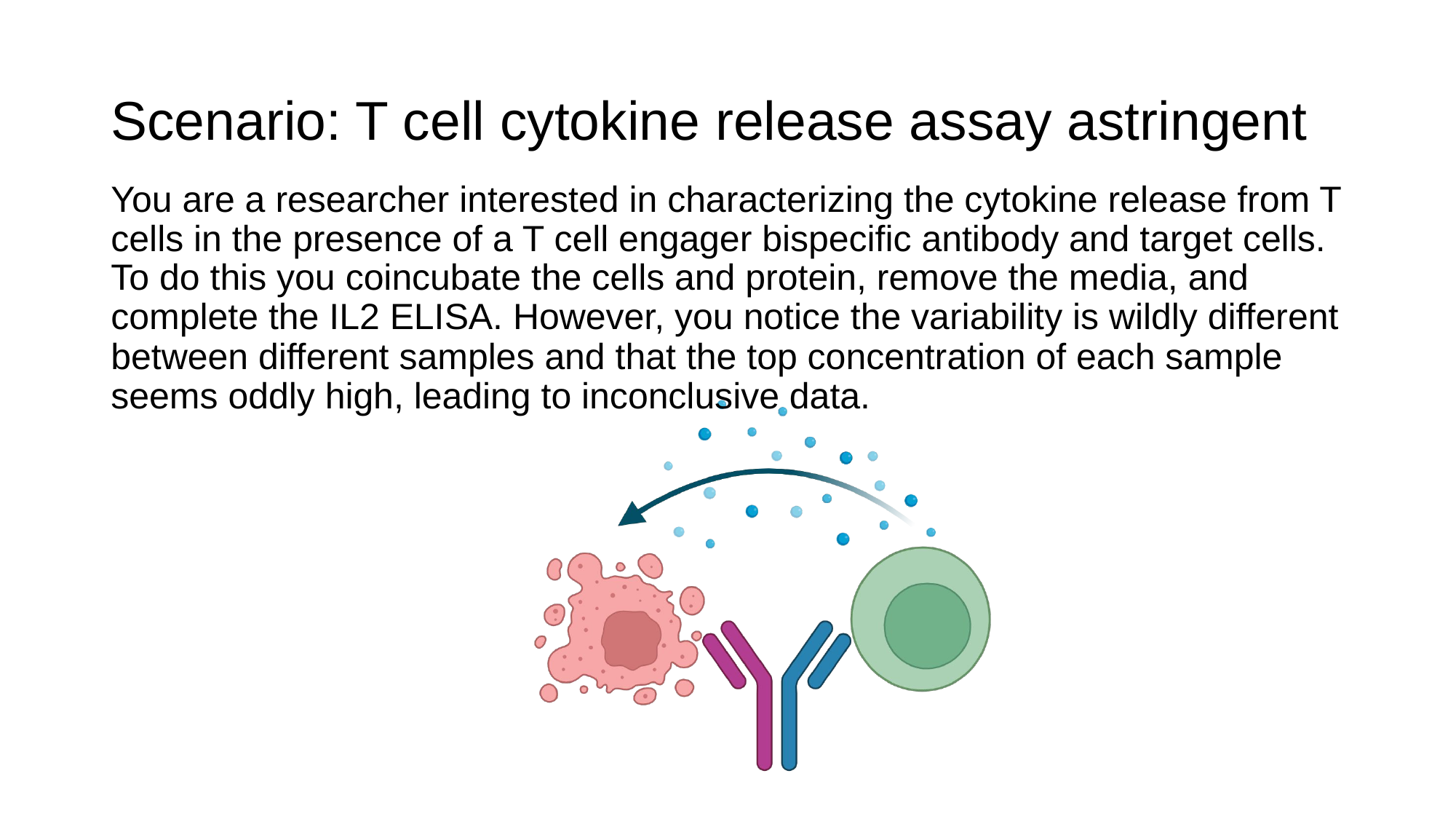

# Scenario: T cell cytokine release assay astringent
You are a researcher interested in characterizing the cytokine release from T cells in the presence of a T cell engager bispecific antibody and target cells. To do this you coincubate the cells and protein, remove the media, and complete the IL2 ELISA. However, you notice the variability is wildly different between different samples and that the top concentration of each sample seems oddly high, leading to inconclusive data.

## Slide 3
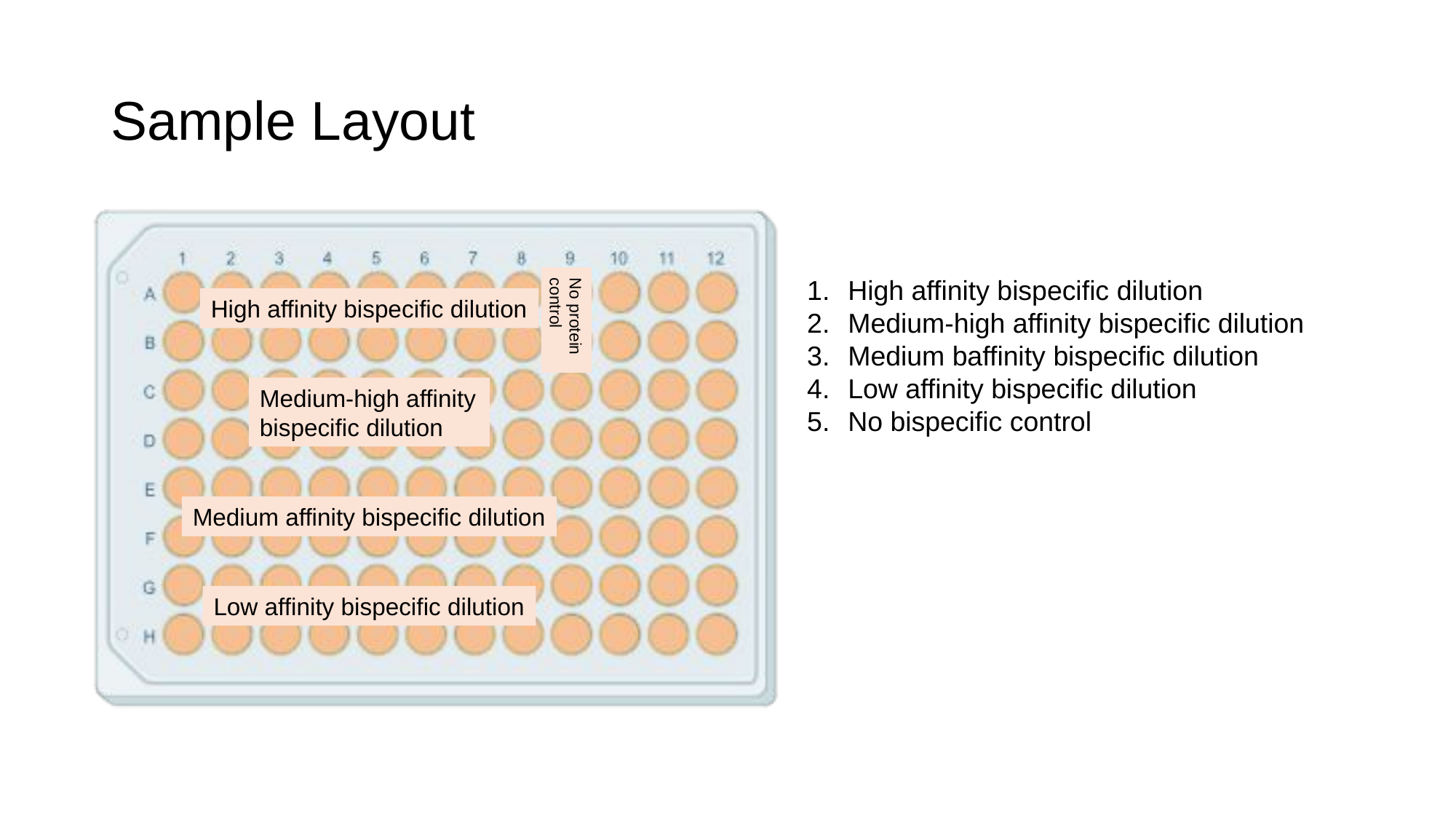

# Sample Layout
High affinity bispecific dilution
Medium-high affinity bispecific dilution
Medium baffinity bispecific dilution
Low affinity bispecific dilution
No bispecific control
High affinity bispecific dilution
No protein control
Medium-high affinity bispecific dilution
Medium affinity bispecific dilution
Low affinity bispecific dilution

## Slide 4
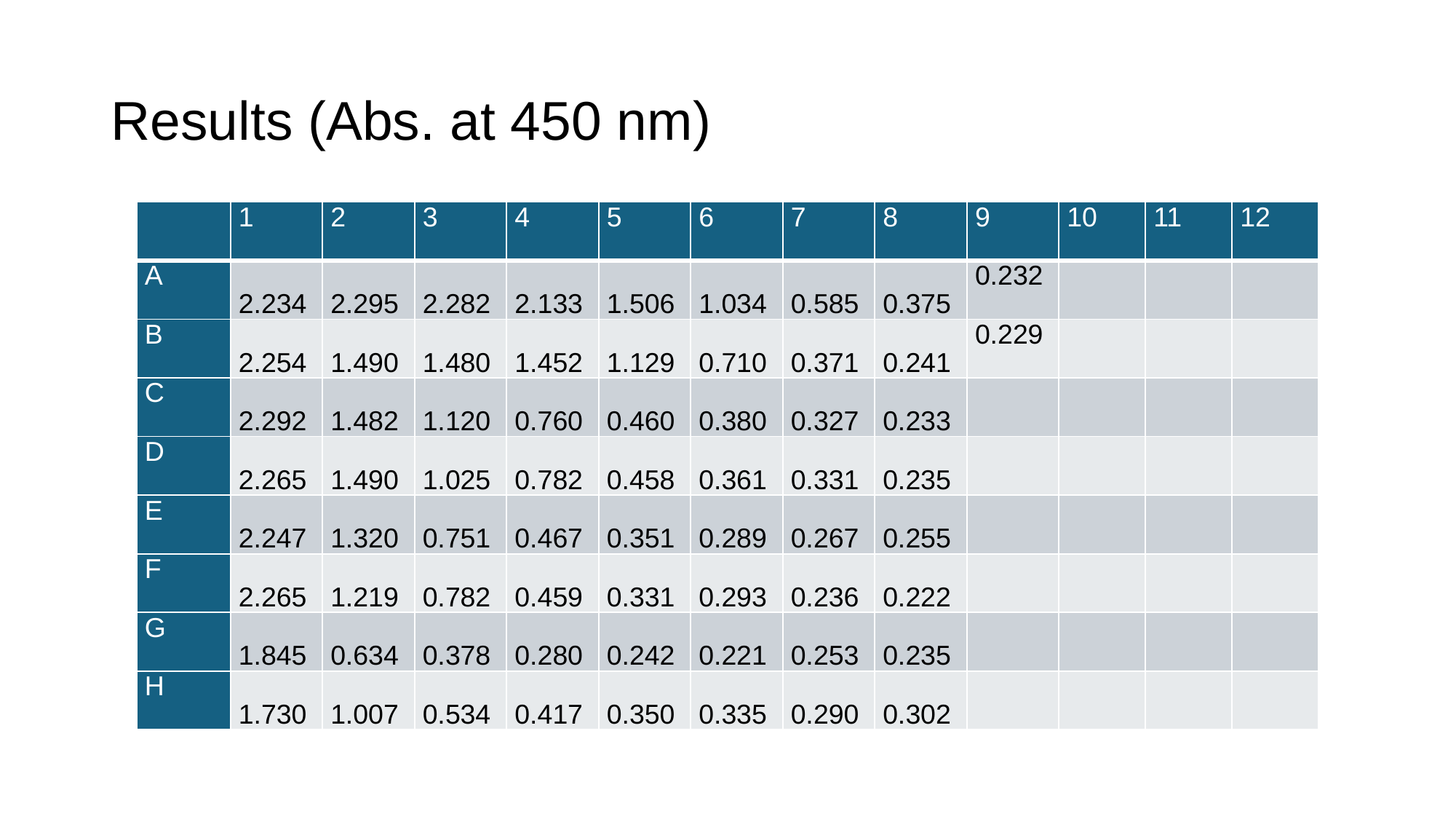

# Results (Abs. at 450 nm)
| | 1 | 2 | 3 | 4 | 5 | 6 | 7 | 8 | 9 | 10 | 11 | 12 |
| --- | --- | --- | --- | --- | --- | --- | --- | --- | --- | --- | --- | --- |
| A | 2.234 | 2.295 | 2.282 | 2.133 | 1.506 | 1.034 | 0.585 | 0.375 | 0.232 | | | |
| B | 2.254 | 1.490 | 1.480 | 1.452 | 1.129 | 0.710 | 0.371 | 0.241 | 0.229 | | | |
| C | 2.292 | 1.482 | 1.120 | 0.760 | 0.460 | 0.380 | 0.327 | 0.233 | | | | |
| D | 2.265 | 1.490 | 1.025 | 0.782 | 0.458 | 0.361 | 0.331 | 0.235 | | | | |
| E | 2.247 | 1.320 | 0.751 | 0.467 | 0.351 | 0.289 | 0.267 | 0.255 | | | | |
| F | 2.265 | 1.219 | 0.782 | 0.459 | 0.331 | 0.293 | 0.236 | 0.222 | | | | |
| G | 1.845 | 0.634 | 0.378 | 0.280 | 0.242 | 0.221 | 0.253 | 0.235 | | | | |
| H | 1.730 | 1.007 | 0.534 | 0.417 | 0.350 | 0.335 | 0.290 | 0.302 | | | | |

## Slide 5
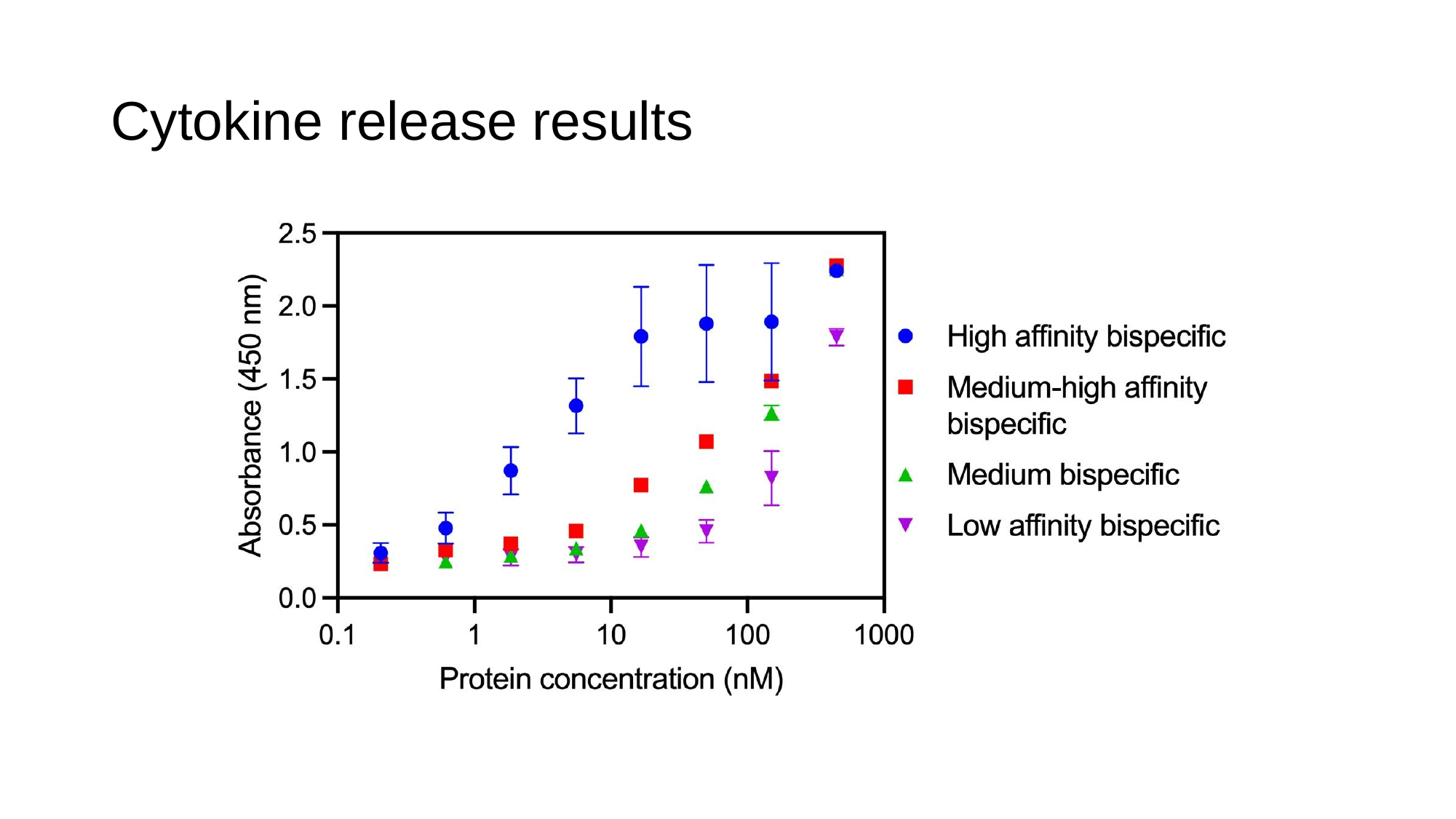

# Cytokine release results

## Slide 6
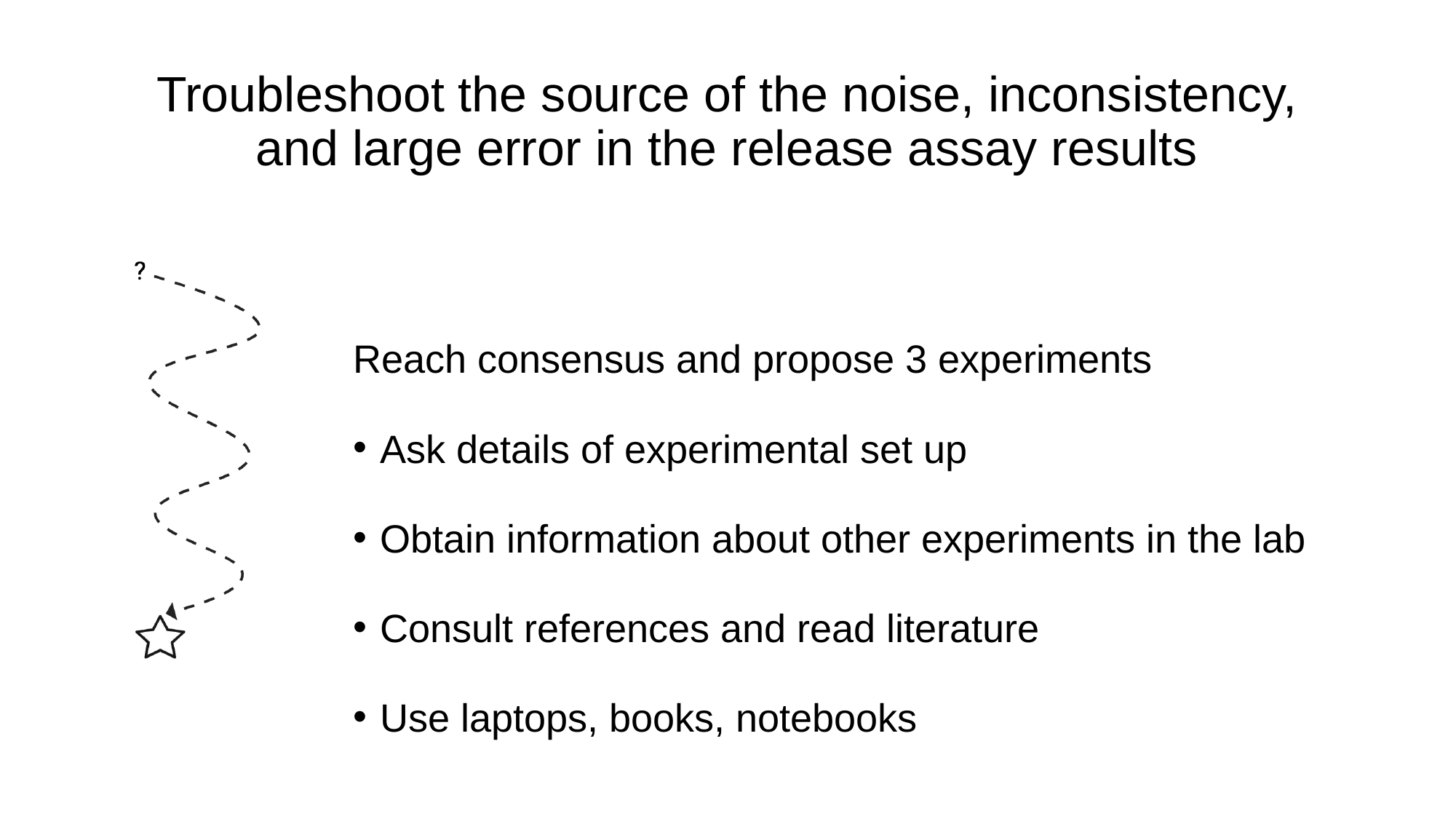

# Troubleshoot the source of the noise, inconsistency, and large error in the release assay results
Reach consensus and propose 3 experiments
Ask details of experimental set up
Obtain information about other experiments in the lab
Consult references and read literature
Use laptops, books, notebooks

## Slide 7
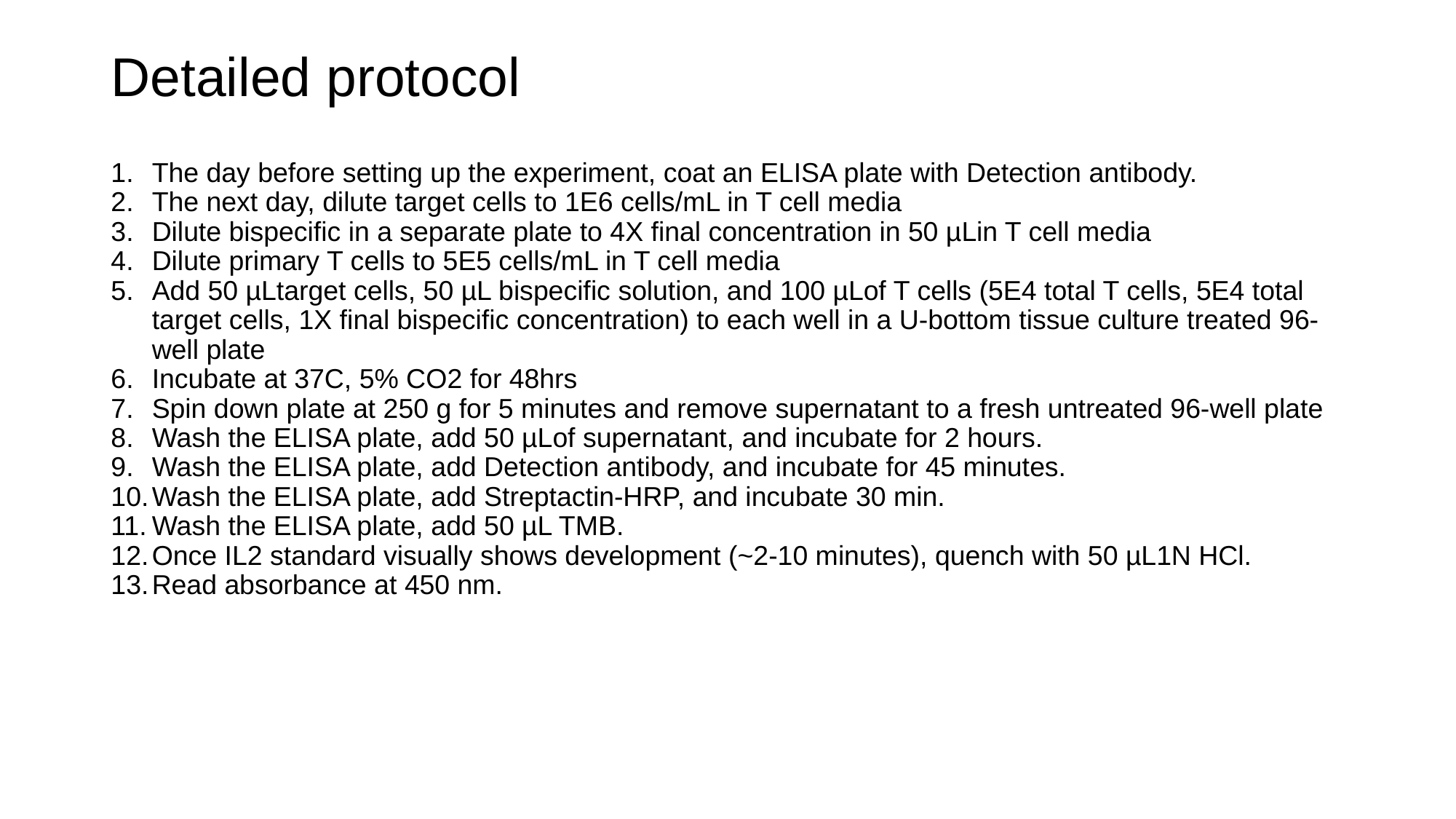

# Detailed protocol
The day before setting up the experiment, coat an ELISA plate with Detection antibody.
The next day, dilute target cells to 1E6 cells/mL in T cell media
Dilute bispecific in a separate plate to 4X final concentration in 50 µLin T cell media
Dilute primary T cells to 5E5 cells/mL in T cell media
Add 50 µLtarget cells, 50 µL bispecific solution, and 100 µLof T cells (5E4 total T cells, 5E4 total target cells, 1X final bispecific concentration) to each well in a U-bottom tissue culture treated 96-well plate
Incubate at 37C, 5% CO2 for 48hrs
Spin down plate at 250 g for 5 minutes and remove supernatant to a fresh untreated 96-well plate
Wash the ELISA plate, add 50 µLof supernatant, and incubate for 2 hours.
Wash the ELISA plate, add Detection antibody, and incubate for 45 minutes.
Wash the ELISA plate, add Streptactin-HRP, and incubate 30 min.
Wash the ELISA plate, add 50 µL TMB.
Once IL2 standard visually shows development (~2-10 minutes), quench with 50 µL1N HCl.
Read absorbance at 450 nm.

## Slide 8
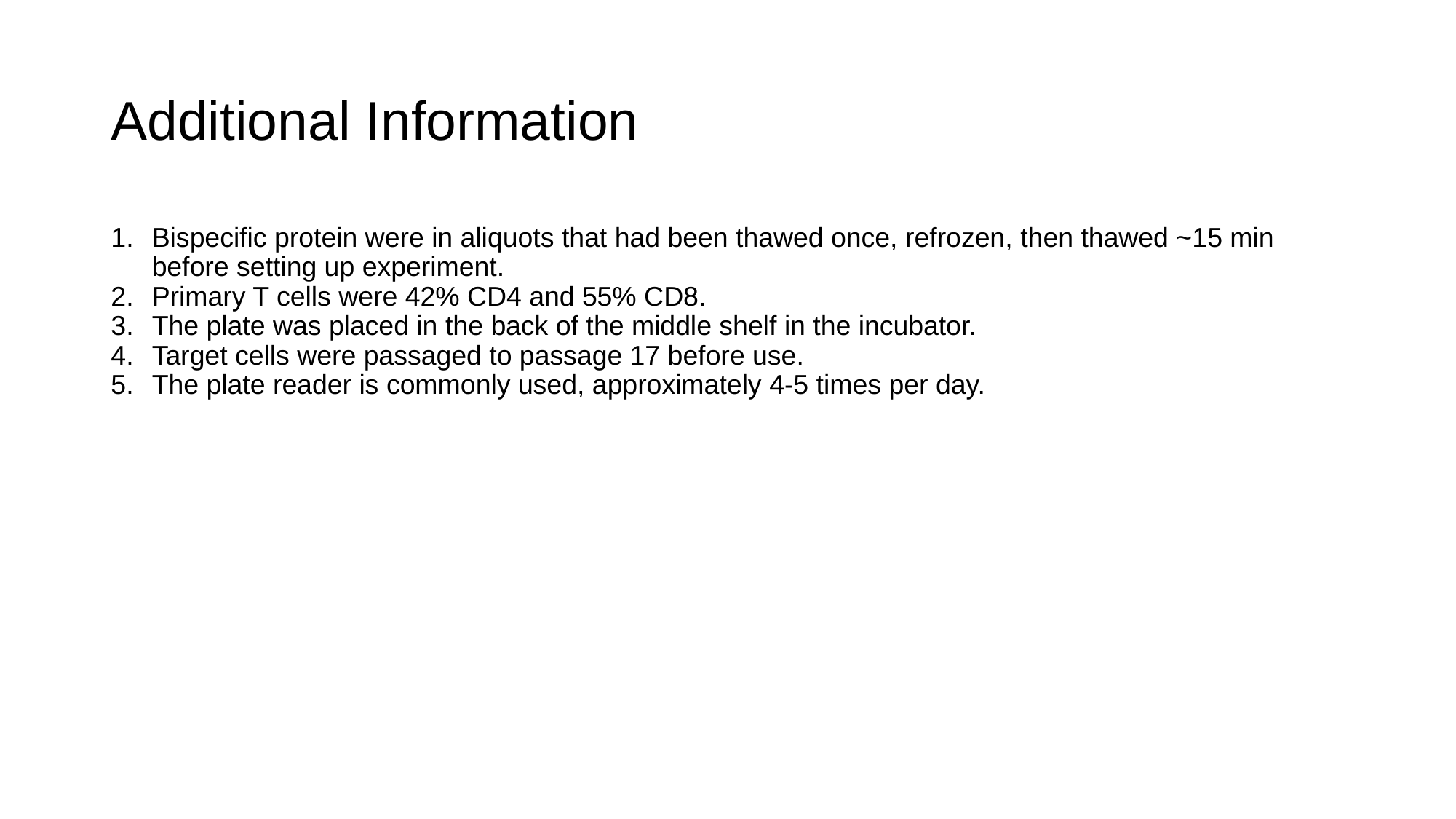

# Additional Information
Bispecific protein were in aliquots that had been thawed once, refrozen, then thawed ~15 min before setting up experiment.
Primary T cells were 42% CD4 and 55% CD8.
The plate was placed in the back of the middle shelf in the incubator.
Target cells were passaged to passage 17 before use.
The plate reader is commonly used, approximately 4-5 times per day.

## Slide 9
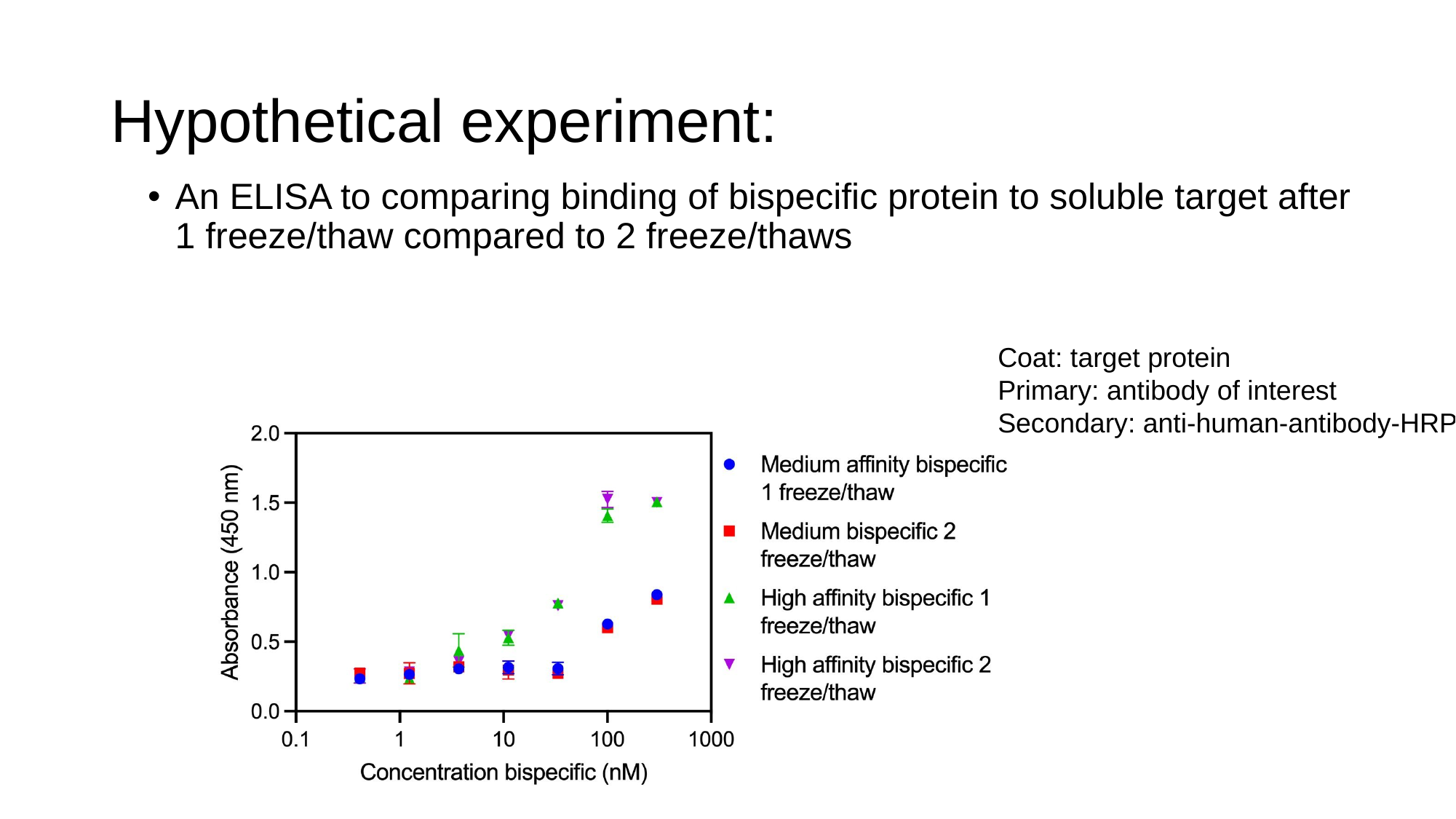

# Hypothetical experiment:
An ELISA to comparing binding of bispecific protein to soluble target after 1 freeze/thaw compared to 2 freeze/thaws
Coat: target protein
Primary: antibody of interest
Secondary: anti-human-antibody-HRP

## Slide 10
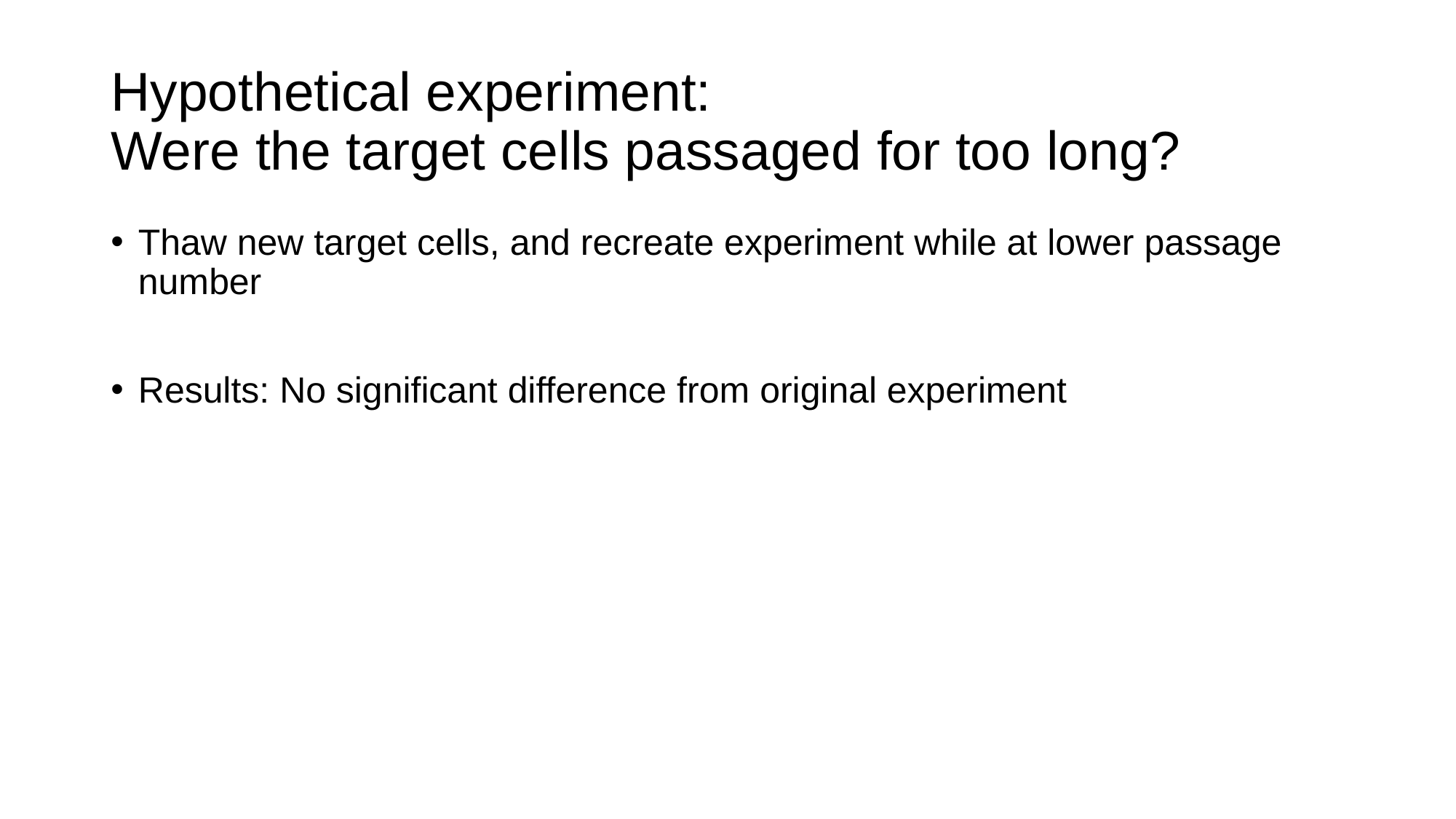

# Hypothetical experiment: Were the target cells passaged for too long?
Thaw new target cells, and recreate experiment while at lower passage number
Results: No significant difference from original experiment

## Slide 11
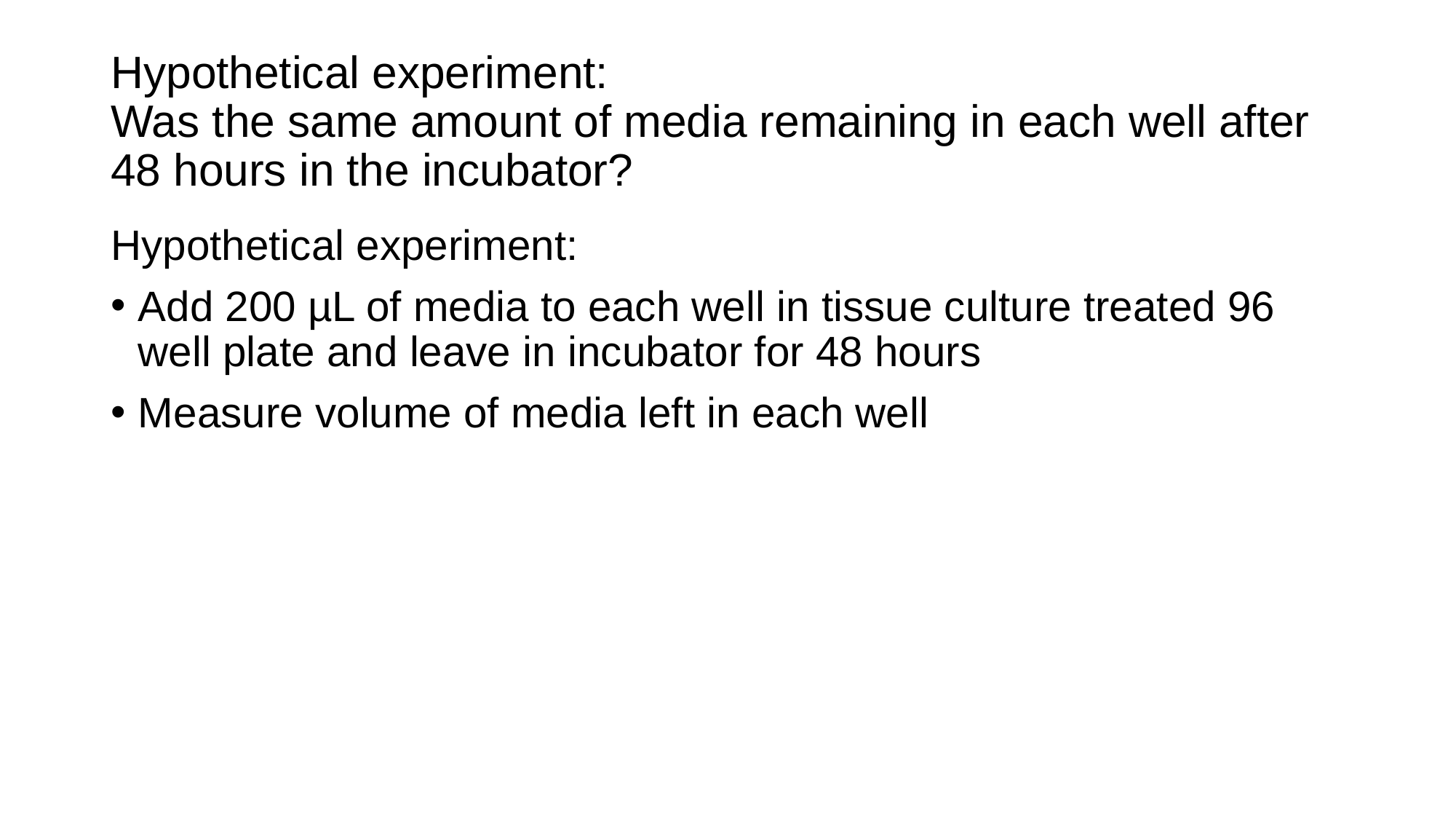

# Hypothetical experiment: Was the same amount of media remaining in each well after 48 hours in the incubator?
Hypothetical experiment:
Add 200 µL of media to each well in tissue culture treated 96 well plate and leave in incubator for 48 hours
Measure volume of media left in each well

## Slide 12
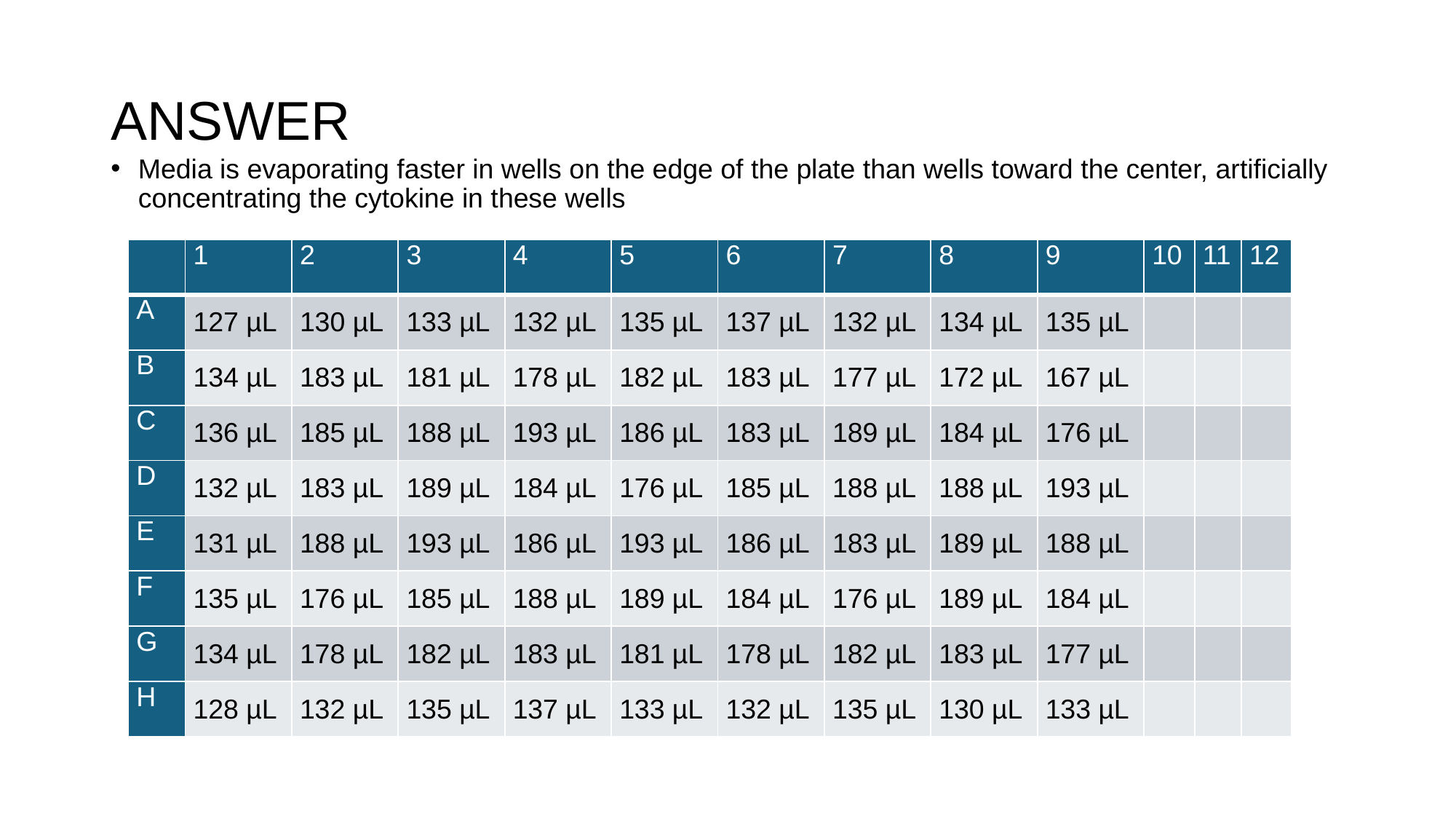

# ANSWER
Media is evaporating faster in wells on the edge of the plate than wells toward the center, artificially concentrating the cytokine in these wells
| | 1 | 2 | 3 | 4 | 5 | 6 | 7 | 8 | 9 | 10 | 11 | 12 |
| --- | --- | --- | --- | --- | --- | --- | --- | --- | --- | --- | --- | --- |
| A | 127 µL | 130 µL | 133 µL | 132 µL | 135 µL | 137 µL | 132 µL | 134 µL | 135 µL | | | |
| B | 134 µL | 183 µL | 181 µL | 178 µL | 182 µL | 183 µL | 177 µL | 172 µL | 167 µL | | | |
| C | 136 µL | 185 µL | 188 µL | 193 µL | 186 µL | 183 µL | 189 µL | 184 µL | 176 µL | | | |
| D | 132 µL | 183 µL | 189 µL | 184 µL | 176 µL | 185 µL | 188 µL | 188 µL | 193 µL | | | |
| E | 131 µL | 188 µL | 193 µL | 186 µL | 193 µL | 186 µL | 183 µL | 189 µL | 188 µL | | | |
| F | 135 µL | 176 µL | 185 µL | 188 µL | 189 µL | 184 µL | 176 µL | 189 µL | 184 µL | | | |
| G | 134 µL | 178 µL | 182 µL | 183 µL | 181 µL | 178 µL | 182 µL | 183 µL | 177 µL | | | |
| H | 128 µL | 132 µL | 135 µL | 137 µL | 133 µL | 132 µL | 135 µL | 130 µL | 133 µL | | | |

## Slide 13
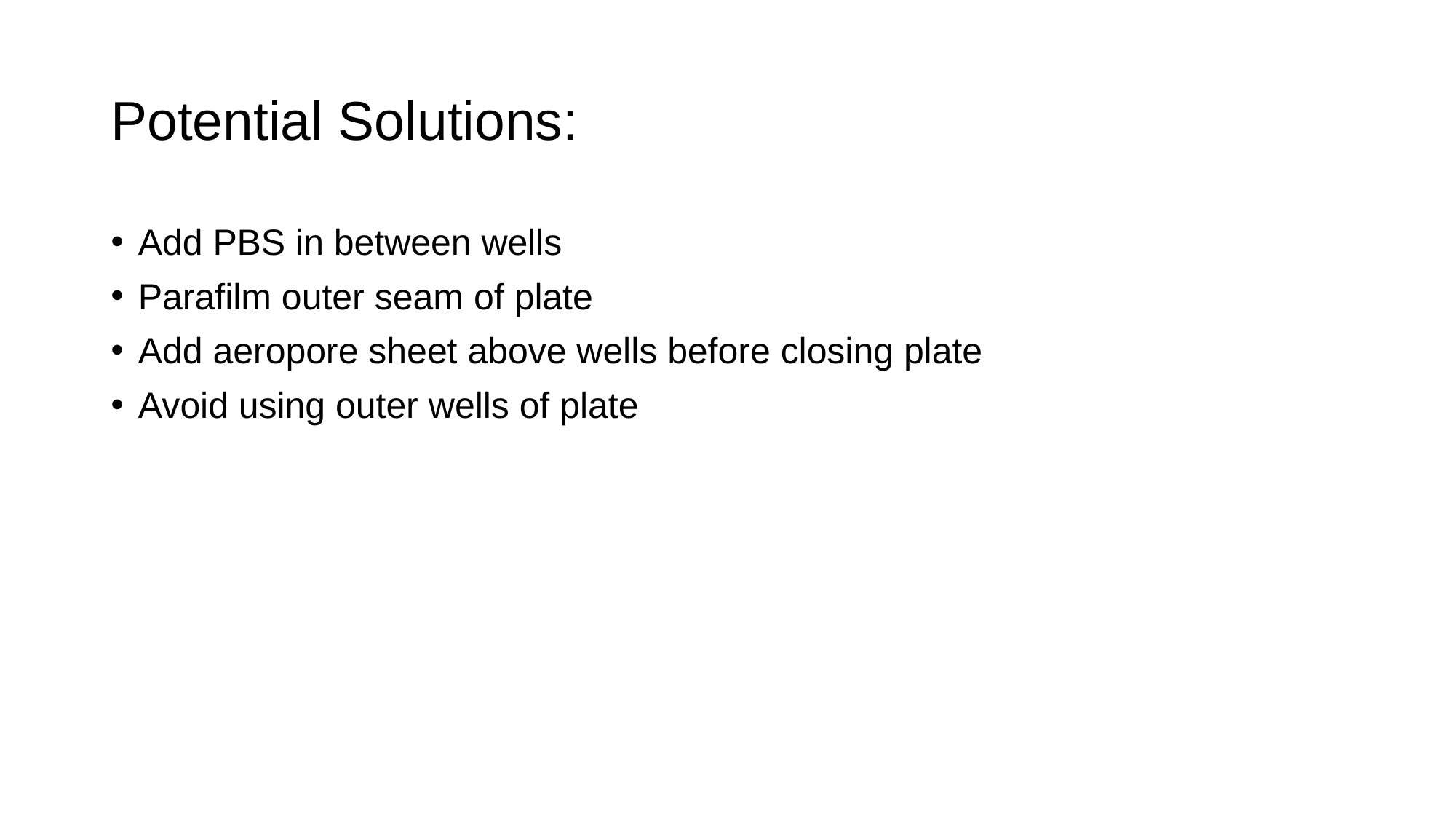

# Potential Solutions:
Add PBS in between wells
Parafilm outer seam of plate
Add aeropore sheet above wells before closing plate
Avoid using outer wells of plate
