# Supplementary material for: Teaching troubleshooting skills to graduate students
Source: eLife. 2024 Sep 17;13:e100761. doi: 10.7554/eLife.100761 (PMC11407763; doi:10.7554/eLife.100761)
Supplement: Supplementary file 1. — For each scenario there is a Word file that contains the following: background information; a description of the scenario; the protocol for the experiment that produced the unexpected result; the results of the experiment; information on the source of the error; background information that can be used to answer questions; and references. There is also a PowerPoint file for each scenario that contains example slides that can be used in real meetings. There are also templates for the Word and PowerPoint files. [file elife-100761-supp1.zip › Final Scenarios/Example6.docx]

**Gibson Cloning Troubles**

Keywords: Synthetic biology, Gibson Assembly, Genetic Engineering

**Background:**

Gibson Assembly was developed by Daniel Gibson and colleagues in 2009, in which the authors established a protocol to assemble plasmids from multi-kilobase pair linear fragments in a one-pot, isothermal reaction^1^. The linear fragments in the Gibson Assembly must share the same terminal sequences, which can be appended via PCR by designing primers to add the desired sequence. Once the sequences are generated, they are assembled using a three-step process: (i) T5 Exonuclease chews back the ends of the fragments exposing single-stranded ends, (ii) Phusion polymerase synthesizes the two overlapping single stands, and (iii) Taq Ligase seals remaining nicks in the new double-stranded product. This entire process occurs in a single reaction buffer at 50°C for approximately an hour. This buffer can then be transformed directly into bacteria to sequence confirm the plasmids contained in the resulting colonies. This process can allow for one-day assemblies of plasmids and has greatly accelerated the speed at which synthetic biology can advance.

**Scenario:**

You are a researcher who is interested in evaluating new enzymes to produce a desired metabolite, as such you utilize Gibson Assembly to insert the DNA sequences of your candidate enzymes into your expression plasmid. Your lab has used Gibson Assembly previously with success. However, when you try to clone your new plasmids via Gibson Assembly, you do not observe any colonies the next day. Your backbone of your plasmid is ~4300 base pairs, while the insert is 840 base pairs.

**Protocol:**

1. Measure concentration of PCR products (from PCR that appended expression plasmid overlap sequences onto target enzyme gene sequences)
2. Thaw 10 µL 2x Gibson Assembly Reaction Buffer aliquots on ice
3. Add insert sequence and plasmid backbone sequence into the Gibson Assembly reaction aliquot in a 3:1 molar ratio, as per NEB Guidelines, and is calculated in Table 1
4. Add DI H_2_O to bring the total reaction volume of 20 µL
5. Mix complete reaction mixture via pipetting
6. Run Gibson Assembly reaction by heating the aliquot at 50°C for one hour
7. Add 2 µL of Gibson Assembly reaction to 100 µL of chemically competent prepared DH5α *E. coli* cells
8. Place mixture on ice for 15 minutes before transferring to 42°C water bath for 45 seconds
9. Return mixture back to ice for an additional 2 minutes
10. Add 900 µL of LB Media and recover cells for 1 hour in a 37°C shaking incubator
11. Pellet the cells by centrifuging at 5000 rpm for 5 minutes
12. Resuspend pellet in 200 µL of LB and spread all 200 µL onto LB + Antibiotic Agar plate
13. Grow plates overnight in a 37°C standing incubator
14. Pick single colonies from the plates into a 5 mL LB + antibiotic liquid culture and grow overnight in a 37°C shaking incubator
15. Purify plasmids from overnight culture using QIAprep Spin Miniprep kit (Cat. No. 27104)

**Table 1.** Gibson Assembly Volumes Sample Spreadsheet. Moles and volumes are calculated based on the backbone mass set at 100 ng, as well as concentrations and lengths of each PCR product.

| DNA Part | Concentration (ng/µL) | Length (bp) | Volume to Add (µL) |  | Mass Added (ng) | Moles (pmols) |
| --- | --- | --- | --- | --- | --- | --- |
| Backbone DNA | 34.6 | 4325 | 2.89 |  | 100 | 0.036 |
| Enzyme insert | 40.3 | 840 | 1.45 |  |  | 0.107 |
| Water | N/A | N/A | 5.66 |  |  |  |
| Gibson Assembly Buffer | N/A | N/A | 10 |  |  |  |

**Experimental Outcomes and Example Images**

These experimental outcomes are purely hypothetical and do not reflect a real experiment. It should not be cited, used, or interpreted in any way. It is solely for training exercise.


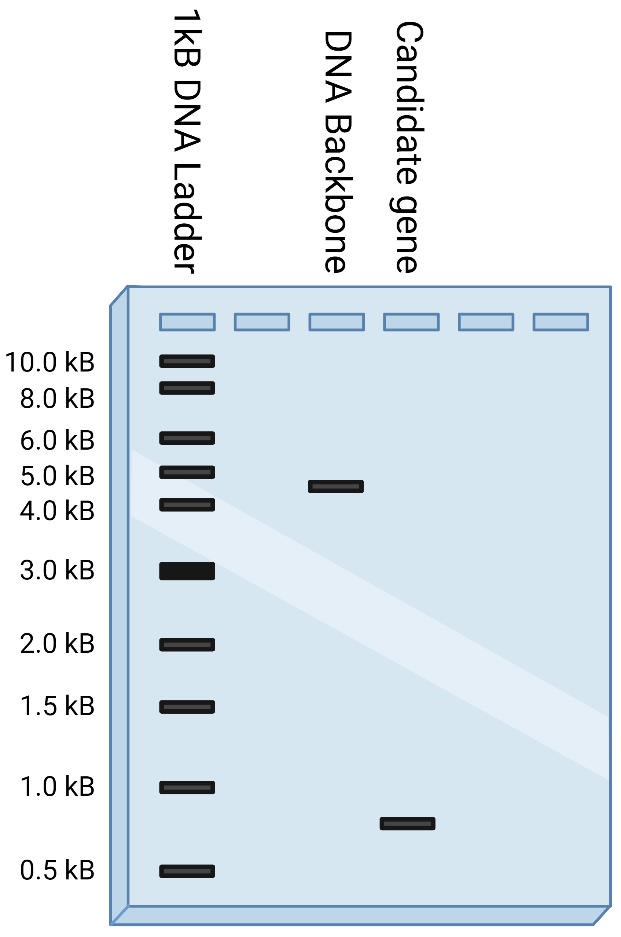


**Figure 1.** Simulated Agarose Gel of PCR Products used in the Gibson Assembly

**
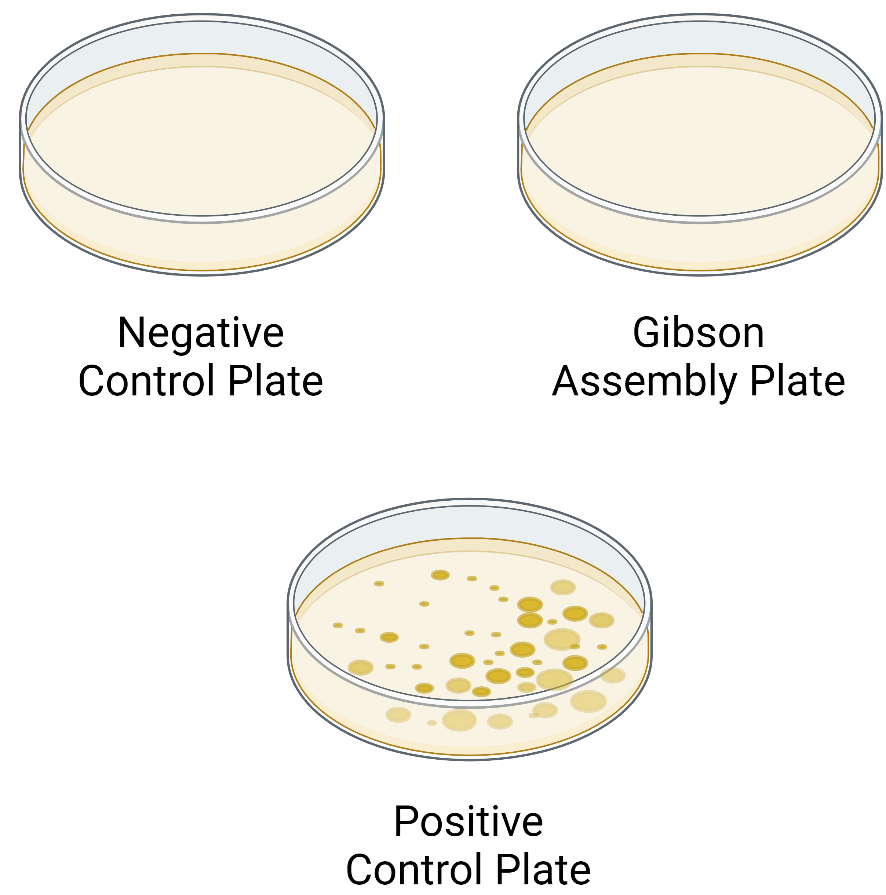
**

**Figure 2.** Images of plates containing Gibson Assembly products transformed into DH5α cells

**Transformation Sample List**

1. Negative Control Plate – Backbone plasmid that has been enzymatically degraded by DnpI
2. Gibson Assembly Reaction – Prepared as outlined in Table 1

**Details:**

1. The PCR products shown in Figure 1 are of the expected sizes
2. Main Components of the Gibson Assembly Buffer
   1. 5x Isothermal Buffer (Cold Spring Harbor Protocols)
   2. NEB T5 Exonuclease (M0663L)
   3. NEB Phusion High-Fidelity DNA Polymerase (M0350L)
   4. NEB T4 DNA Ligase (MS0202T)
   5. DI H_2_O
3. Gibson Assembly reaction buffer aliquots and all components of the buffer must be stored at -20°C, excluding the DI H_2_O
4. Aliquots can be freeze-thawed multiple times and still be viable

**Source of Error:**

The T5 Exonuclease was inactivated due to the Buffer being left out for about an hour when being used for a Gibson Assembly over the weekend. The T5 Exonuclease was left at room temperature for too long, which caused the enzyme to effectively expire. Since the Gibson Assembly Buffer utilizes commercial enzymes, these can be extremely temperature sensitive and can become inactive if not handled properly. The inactive T5 Exonuclease does not chew back the ends of the PCR fragments meaning the single-stranded ends are not generated, and thus cannot be synthesized by the polymerase. Therefore, no full plasmids are assembled, leading to no colony formation when grown on LB + Antibiotic Agar plates.

**Table 1.** Additional information known by the leader that can be provided upon request

| **Meeting Notes for the Leader**  Not to be shared with the group | |
| --- | --- |
| Other researcher’s experiments | - Those also using Gibson assembly are having the same issue and not seeing colonies on their transformations - Share if asked - Restriction Enzyme cloning is working - Share if asked - GoldenGate cloning working |
| Storage information | - Gibson Assembly Buffer is stored in the -20 °C Freezer and can be quickly thawed as needed without loss of fidelity - This buffer stock is approximately 6 weeks old |
| Construct information | - Plasmid is using a standard ColE1 origin of replication - Antibiotic selection marker is Carbenicillin - Assume gene of interest is controlled via LacI induction system contained within the plasmid |
| Source of error | - T5 Exonuclease has expired, due to the Buffer being left out for several hours last week |
| Hints for group | - Direct group to evaluate if transformation or assembly is the issue if stuck on where to begin - Direct them away from redoing PCRs/primer design - If stuck on troubleshooting assembly issues, highlight that enzymes in Buffer are temperature sensitive |

**References**

(1) Gibson, D. G.; Young, L.; Chuang, R.-Y.; Venter, J. C.; Hutchison, C. A.; Smith, H. O. Enzymatic Assembly of DNA Molecules up to Several Hundred Kilobases. *Nat. Methods* **2009**, *6* (5), 343–345. https://doi.org/10.1038/nmeth.1318.
