# Supplementary material for: Teaching troubleshooting skills to graduate students
Source: eLife. 2024 Sep 17;13:e100761. doi: 10.7554/eLife.100761 (PMC11407763; doi:10.7554/eLife.100761)
Supplement: Supplementary file 1. — For each scenario there is a Word file that contains the following: background information; a description of the scenario; the protocol for the experiment that produced the unexpected result; the results of the experiment; information on the source of the error; background information that can be used to answer questions; and references. There is also a PowerPoint file for each scenario that contains example slides that can be used in real meetings. There are also templates for the Word and PowerPoint files. [file elife-100761-supp1.zip › Final Scenarios/Example.template.pptx]

## Slide 1
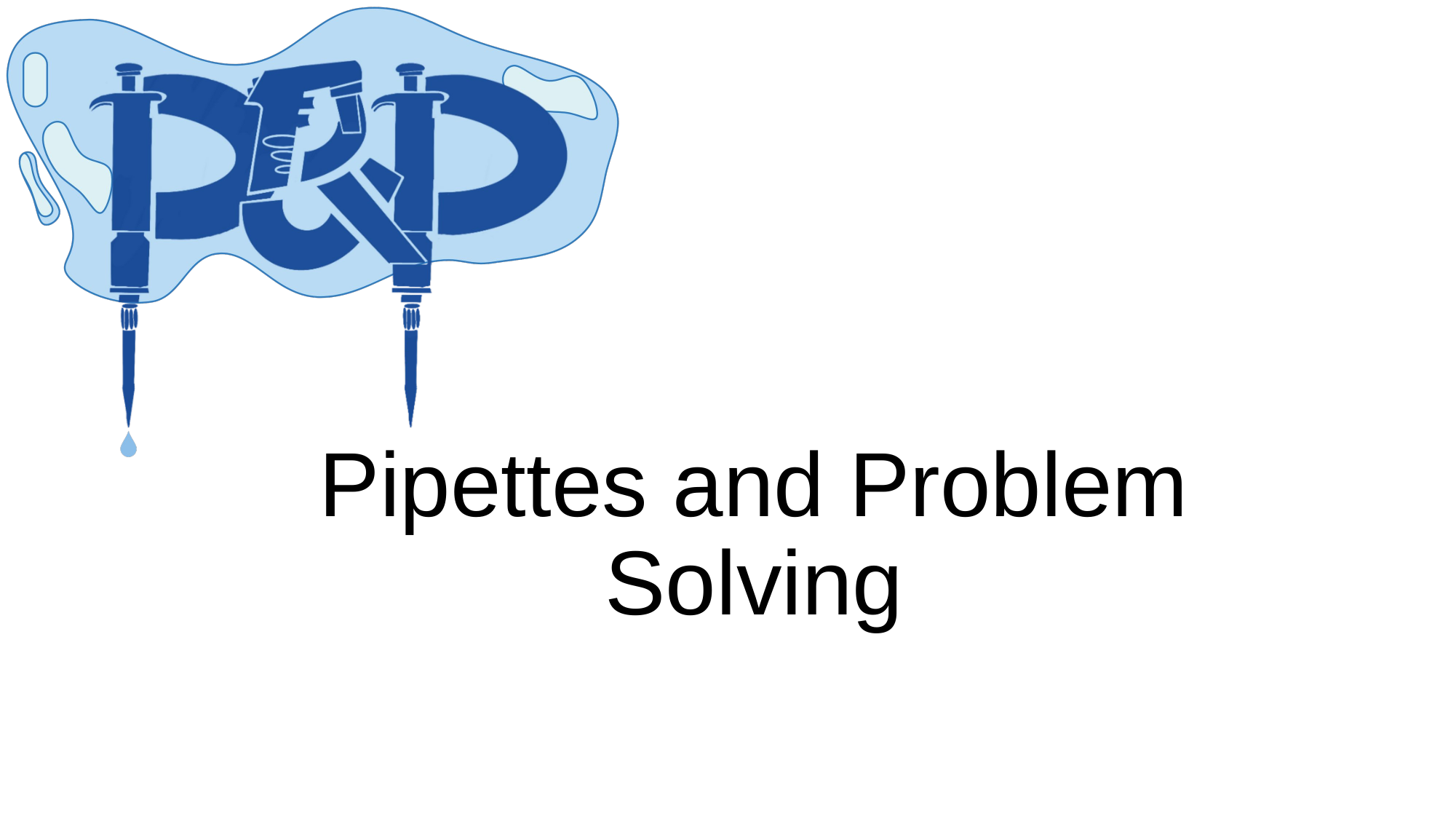

# Pipettes and Problem Solving

## Slide 2
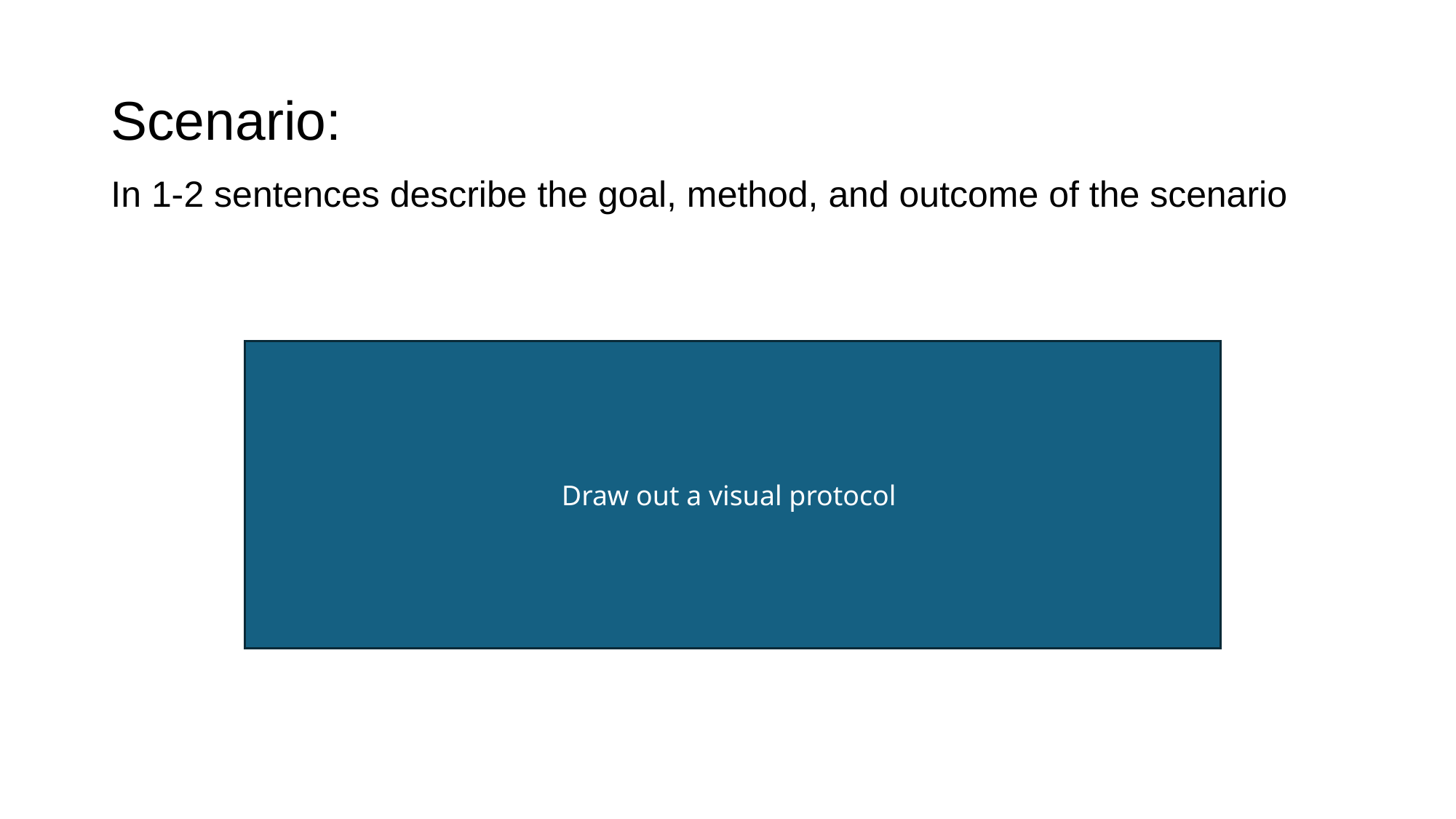

# Scenario:
In 1-2 sentences describe the goal, method, and outcome of the scenario
Draw out a visual protocol

## Slide 3
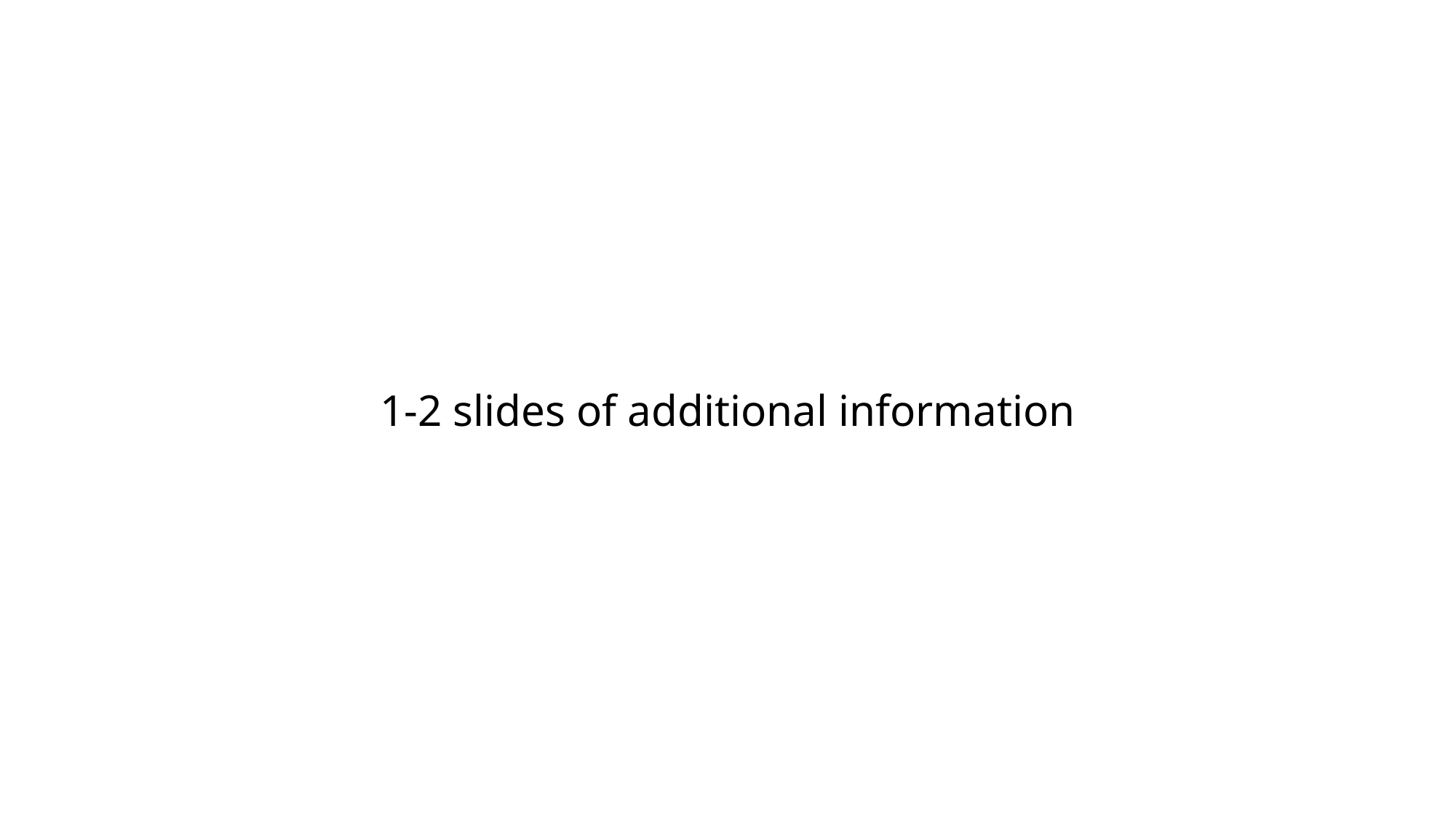

1-2 slides of additional information

## Slide 4
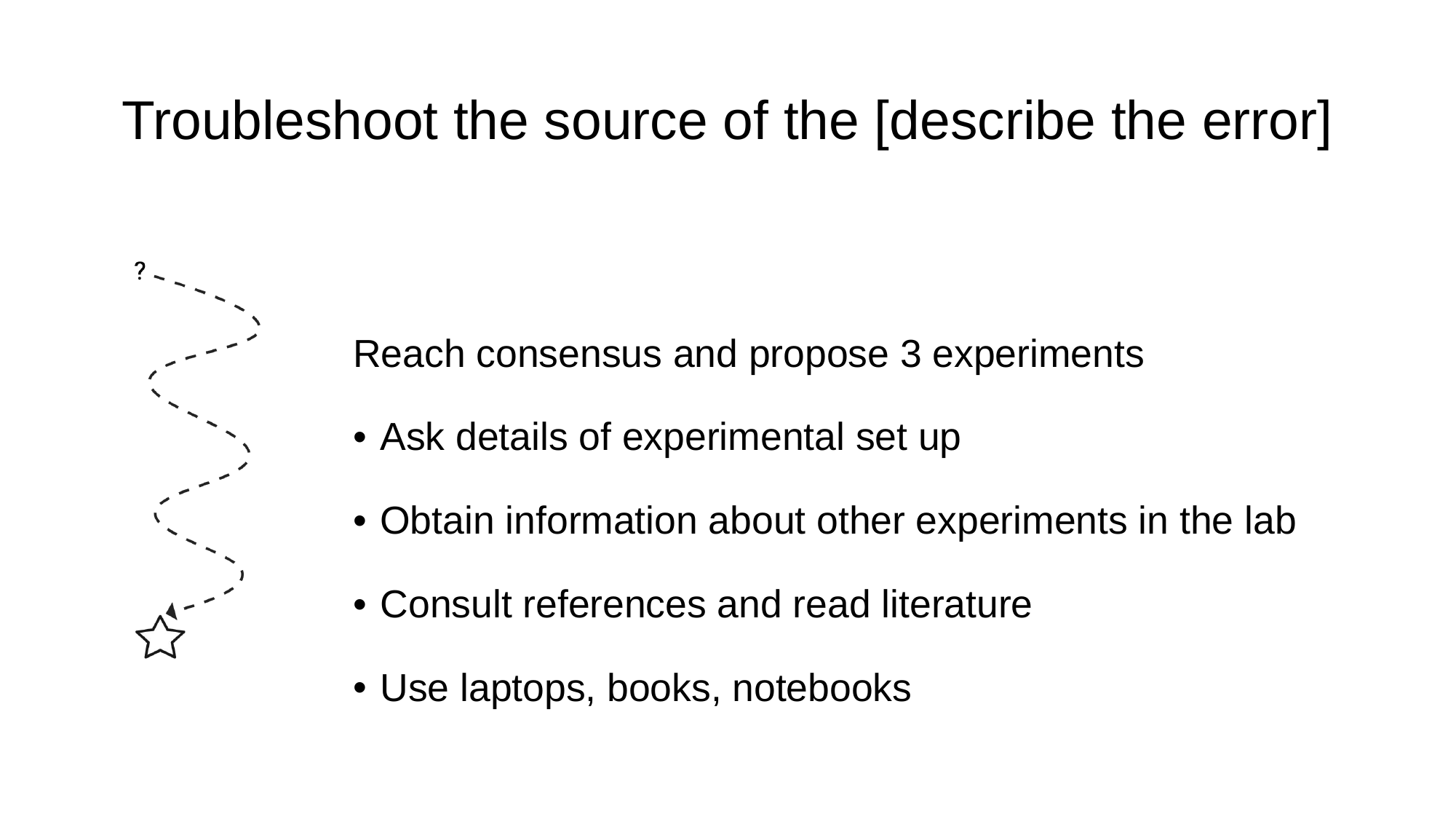

# Troubleshoot the source of the [describe the error]
Reach consensus and propose 3 experiments
Ask details of experimental set up
Obtain information about other experiments in the lab
Consult references and read literature
Use laptops, books, notebooks

## Slide 5
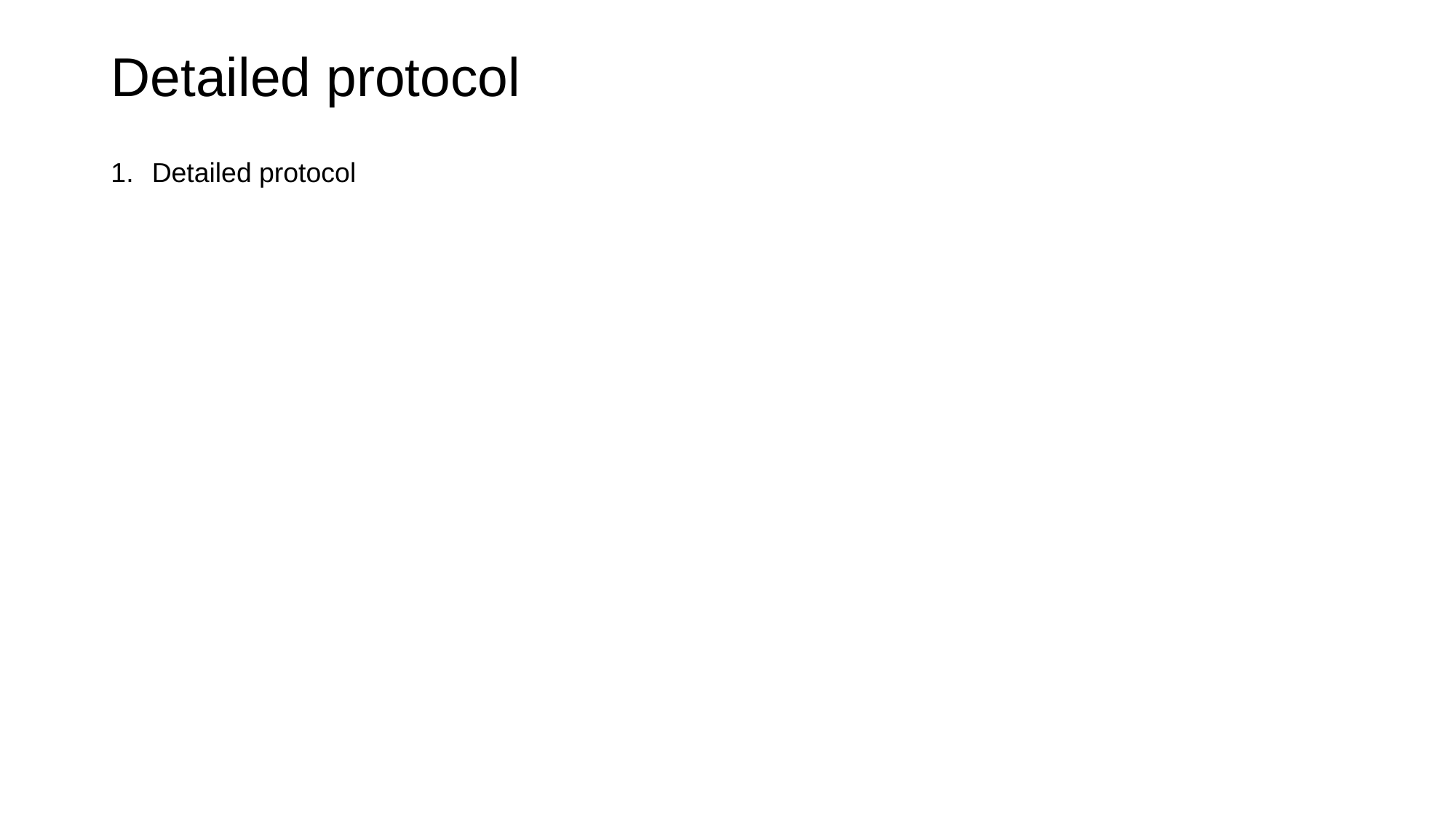

# Detailed protocol
Detailed protocol

## Slide 6
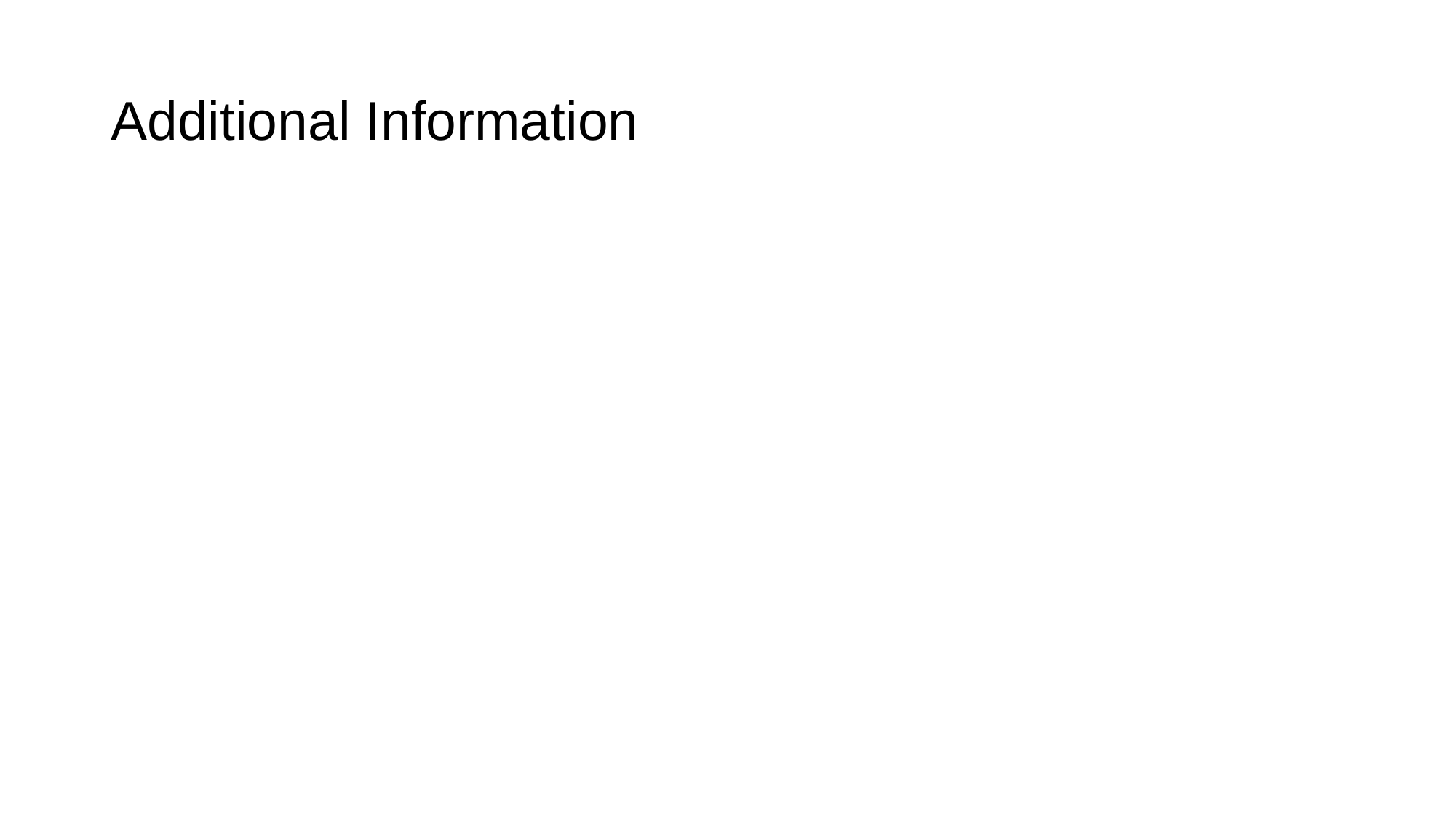

# Additional Information

## Slide 7
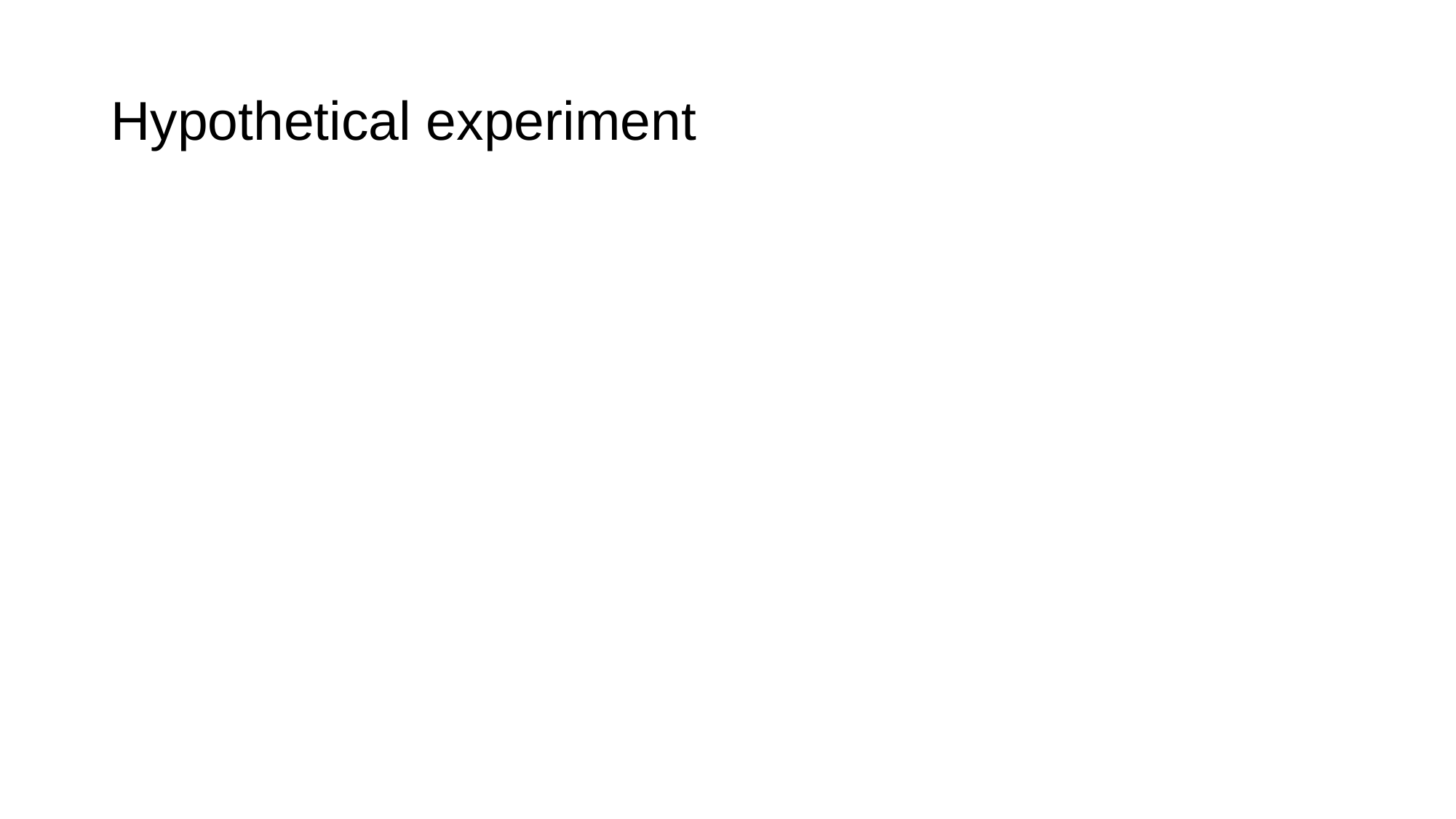

# Hypothetical experiment

## Slide 8
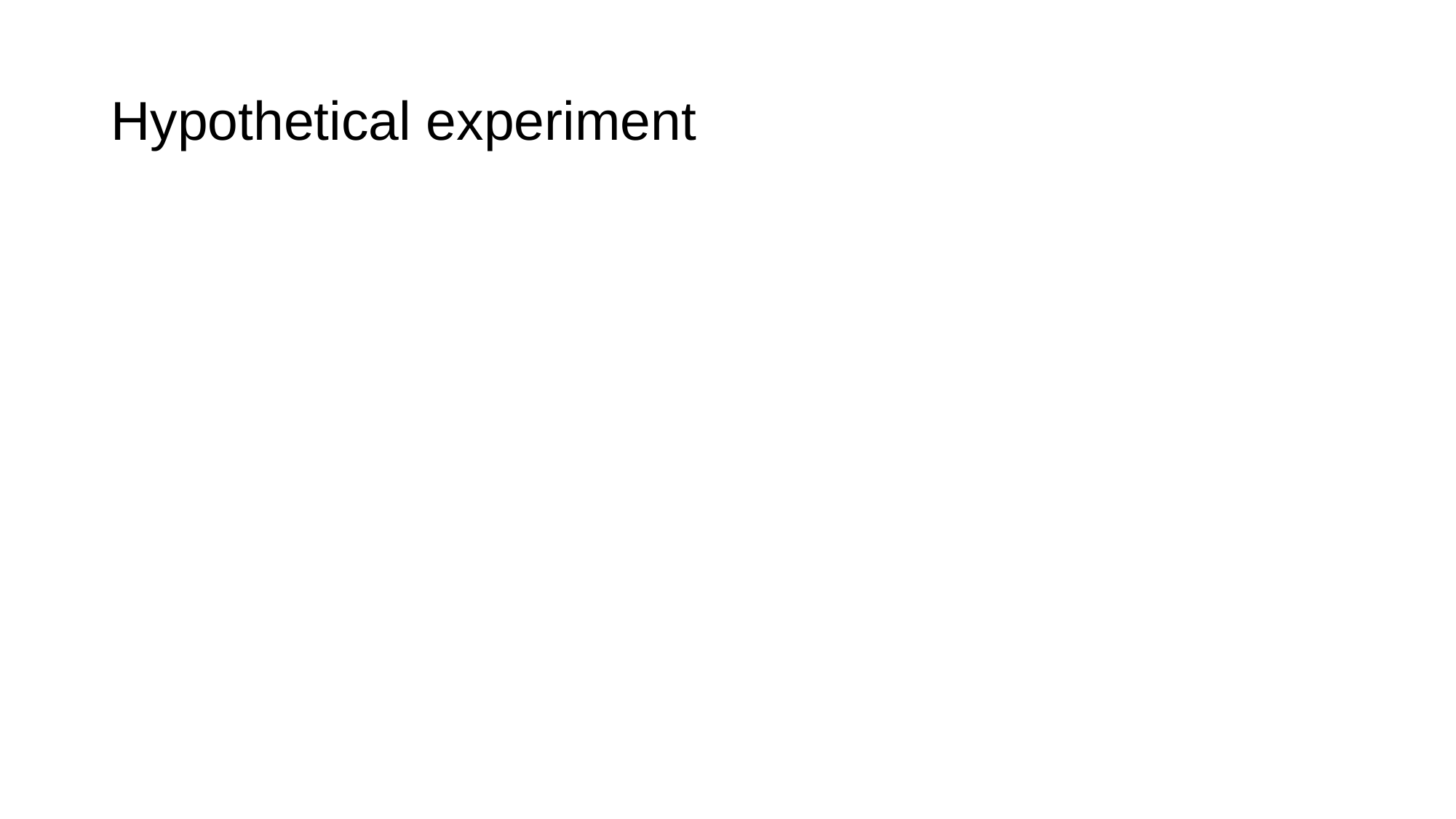

# Hypothetical experiment

## Slide 9
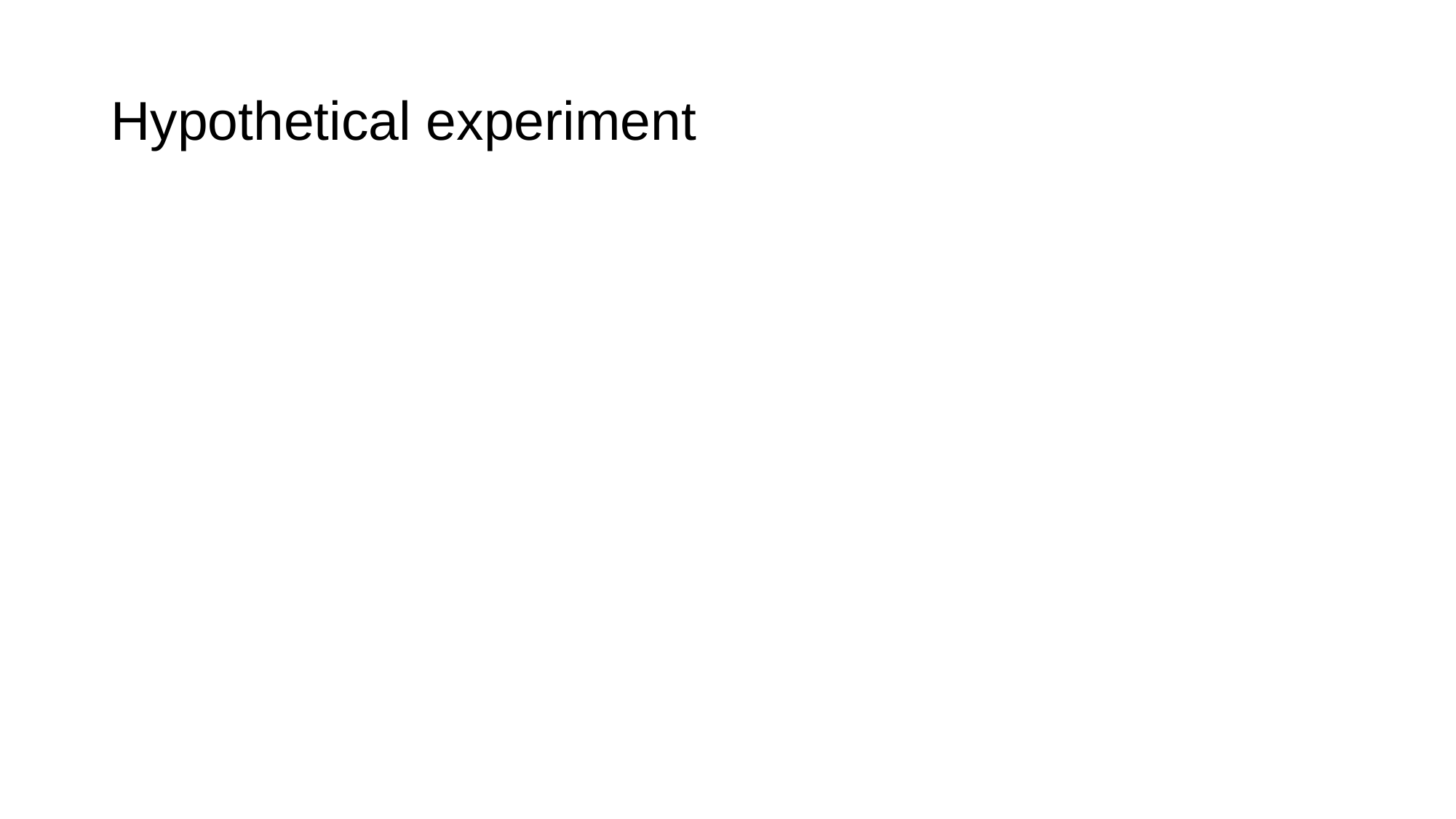

# Hypothetical experiment

## Slide 10
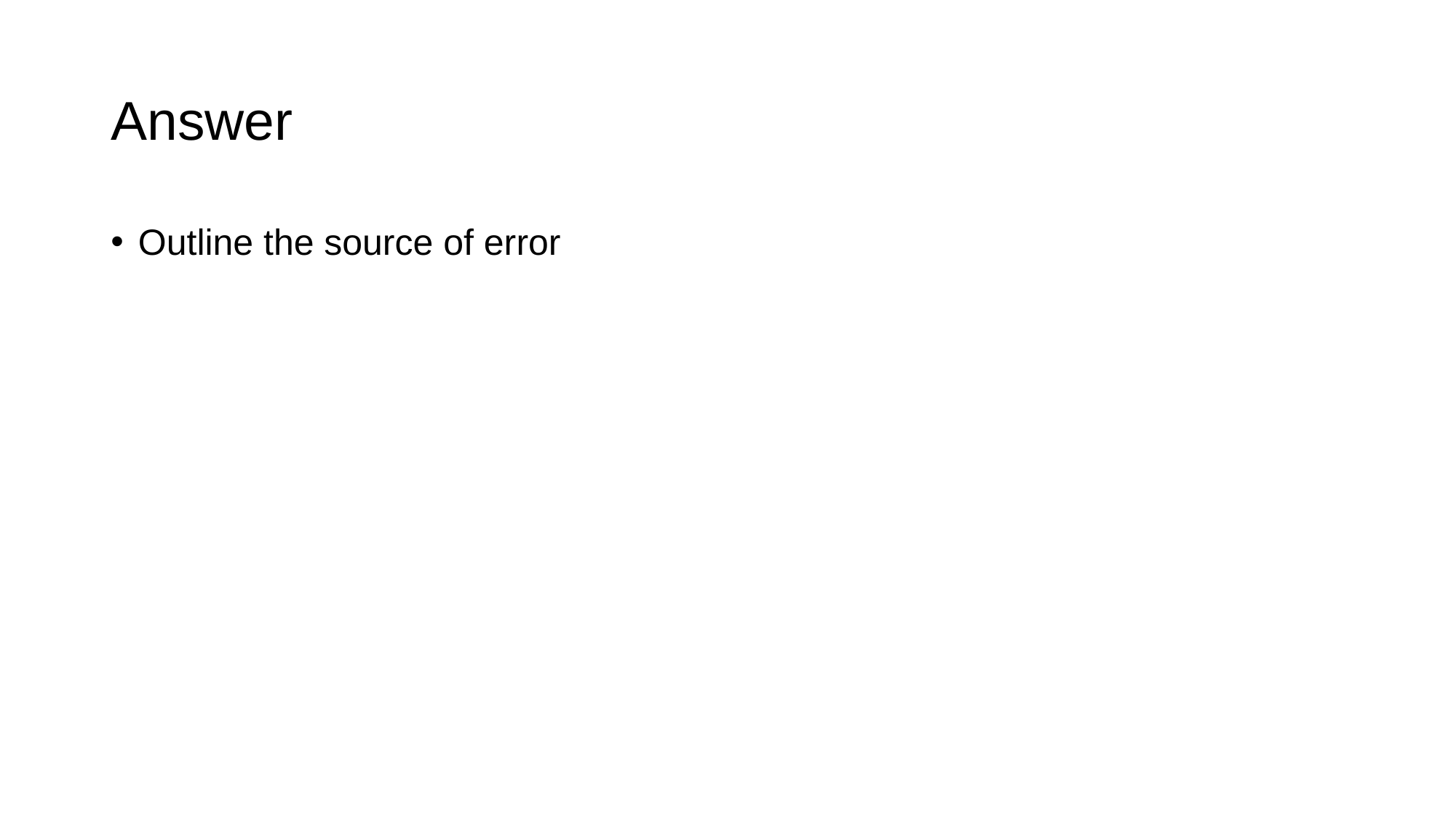

# Answer
Outline the source of error
