# Supplementary material for: Teaching troubleshooting skills to graduate students
Source: eLife. 2024 Sep 17;13:e100761. doi: 10.7554/eLife.100761 (PMC11407763; doi:10.7554/eLife.100761)
Supplement: Supplementary file 1. — For each scenario there is a Word file that contains the following: background information; a description of the scenario; the protocol for the experiment that produced the unexpected result; the results of the experiment; information on the source of the error; background information that can be used to answer questions; and references. There is also a PowerPoint file for each scenario that contains example slides that can be used in real meetings. There are also templates for the Word and PowerPoint files. [file elife-100761-supp1.zip › Final Scenarios/Example1.pptx]

## Slide 1
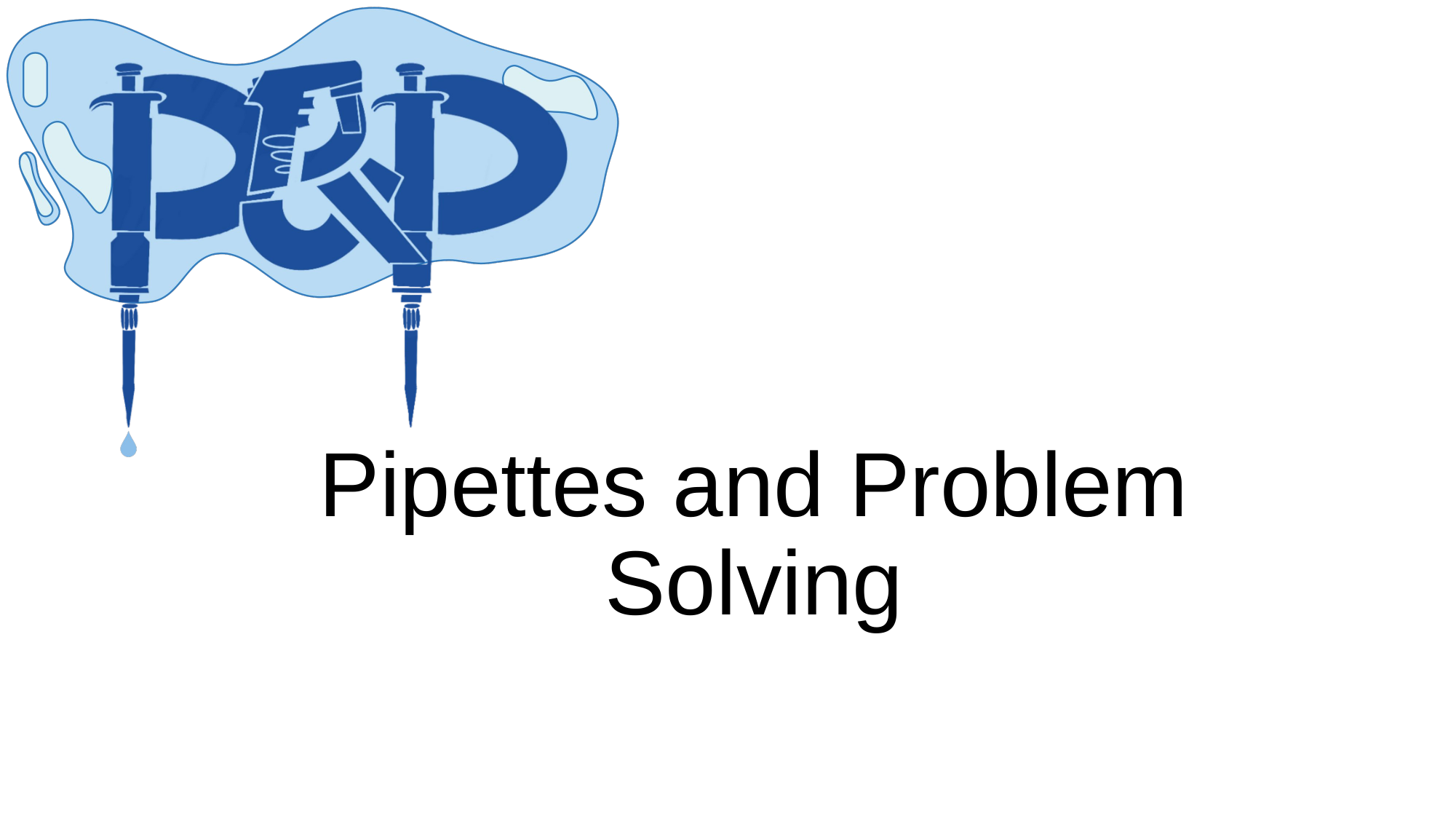

# Pipettes and Problem Solving

## Slide 2
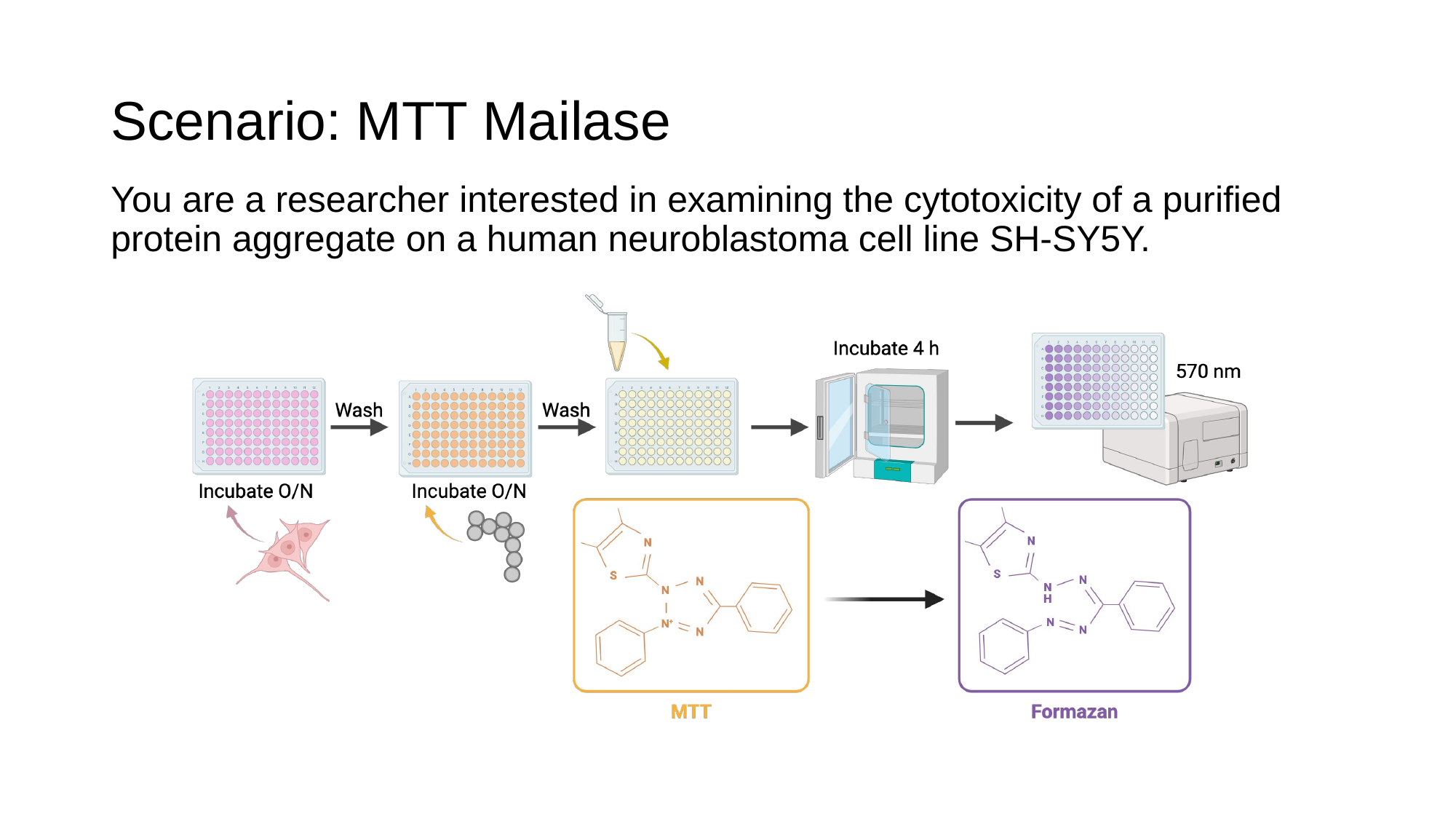

# Scenario: MTT Mailase
You are a researcher interested in examining the cytotoxicity of a purified protein aggregate on a human neuroblastoma cell line SH-SY5Y.

## Slide 3
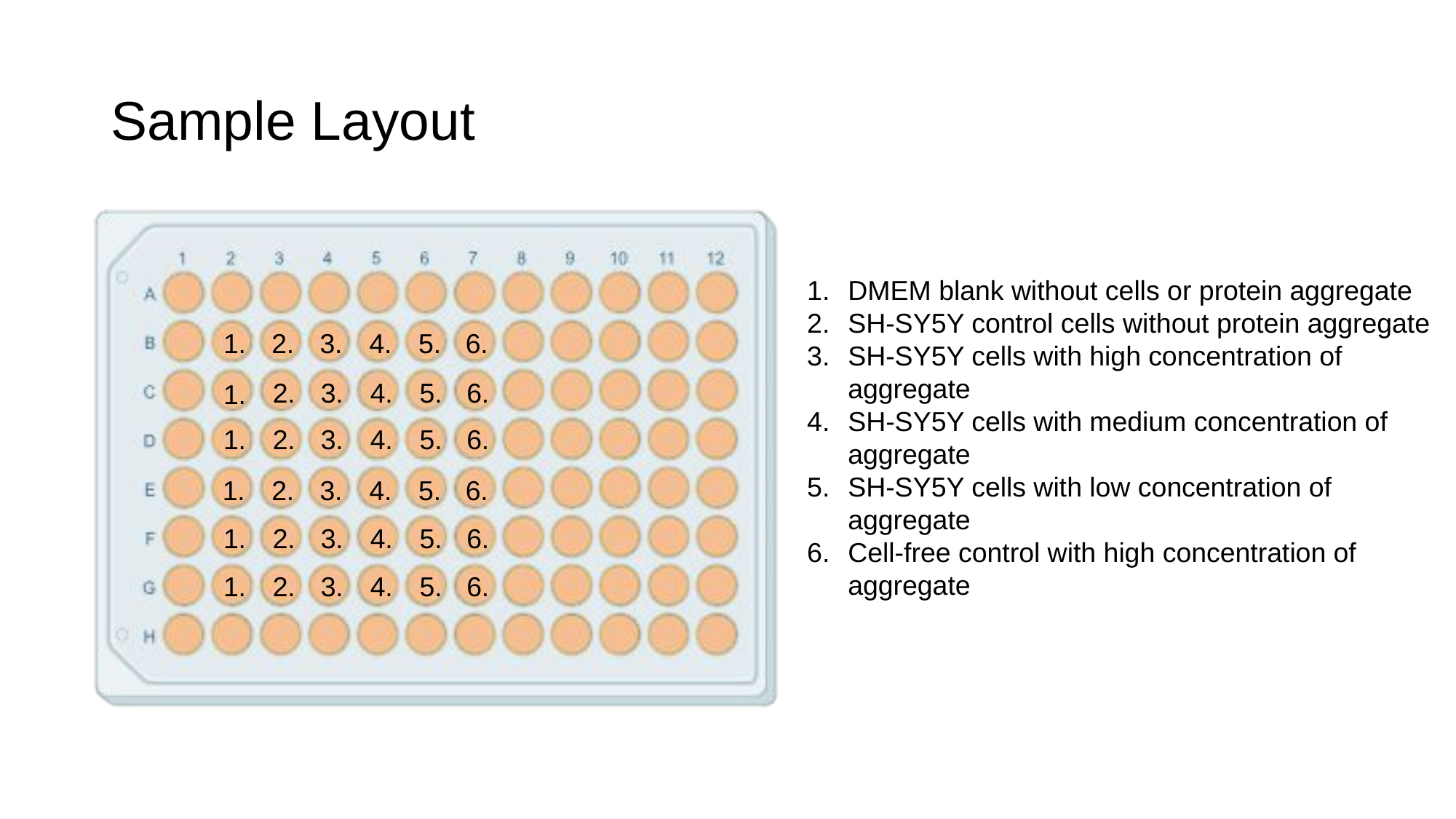

# Sample Layout
DMEM blank without cells or protein aggregate
SH-SY5Y control cells without protein aggregate
SH-SY5Y cells with high concentration of aggregate
SH-SY5Y cells with medium concentration of aggregate
SH-SY5Y cells with low concentration of aggregate
Cell-free control with high concentration of aggregate
1.
2.
3.
4.
5.
6.
2.
3.
4.
5.
6.
1.
1.
2.
3.
4.
5.
6.
1.
2.
3.
4.
5.
6.
1.
2.
3.
4.
5.
6.
1.
2.
3.
4.
5.
6.

## Slide 4
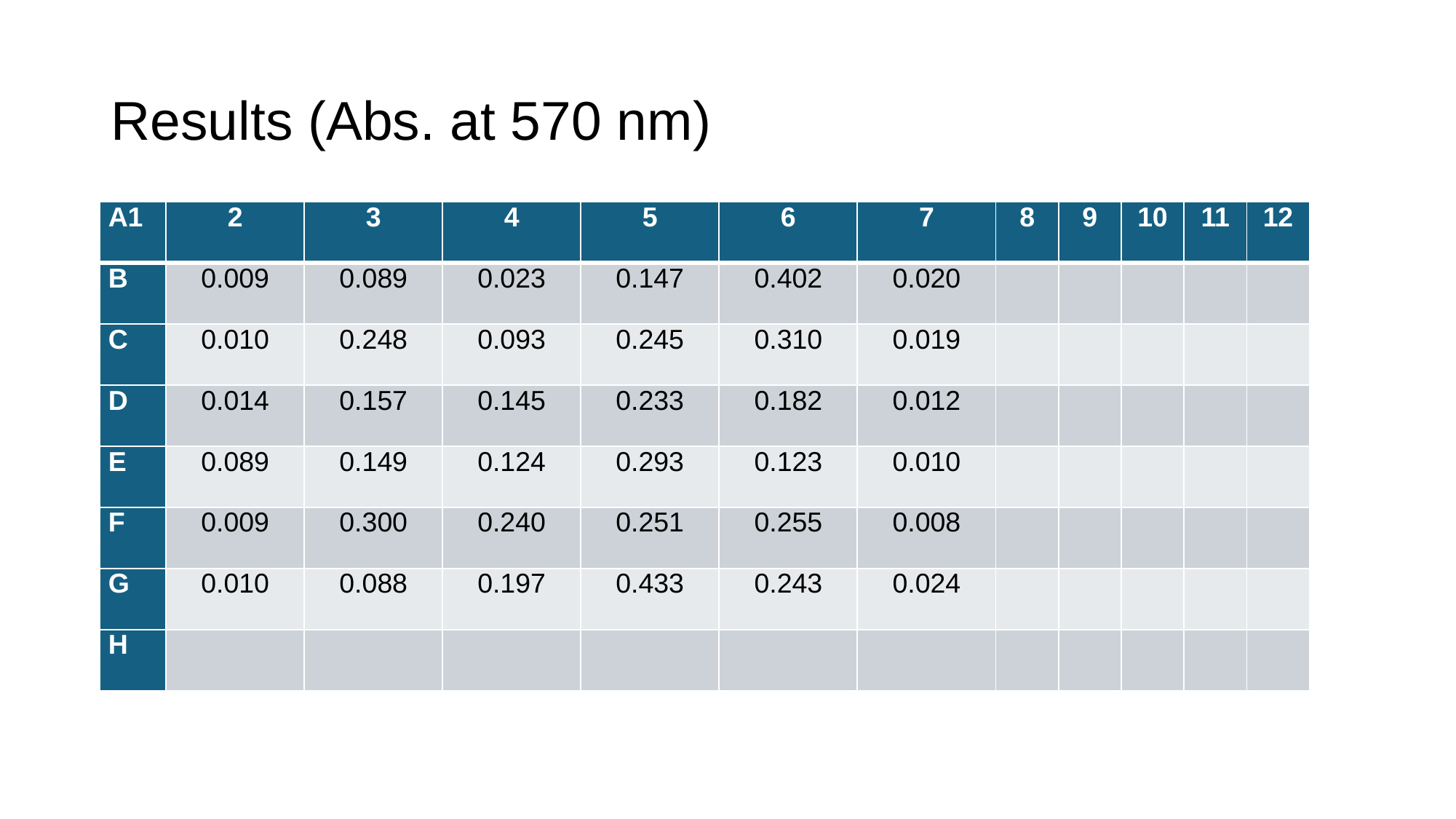

# Results (Abs. at 570 nm)
| A1 | 2 | 3 | 4 | 5 | 6 | 7 | 8 | 9 | 10 | 11 | 12 |
| --- | --- | --- | --- | --- | --- | --- | --- | --- | --- | --- | --- |
| B | 0.009 | 0.089 | 0.023 | 0.147 | 0.402 | 0.020 | | | | | |
| C | 0.010 | 0.248 | 0.093 | 0.245 | 0.310 | 0.019 | | | | | |
| D | 0.014 | 0.157 | 0.145 | 0.233 | 0.182 | 0.012 | | | | | |
| E | 0.089 | 0.149 | 0.124 | 0.293 | 0.123 | 0.010 | | | | | |
| F | 0.009 | 0.300 | 0.240 | 0.251 | 0.255 | 0.008 | | | | | |
| G | 0.010 | 0.088 | 0.197 | 0.433 | 0.243 | 0.024 | | | | | |
| H | | | | | | | | | | | |

## Slide 5
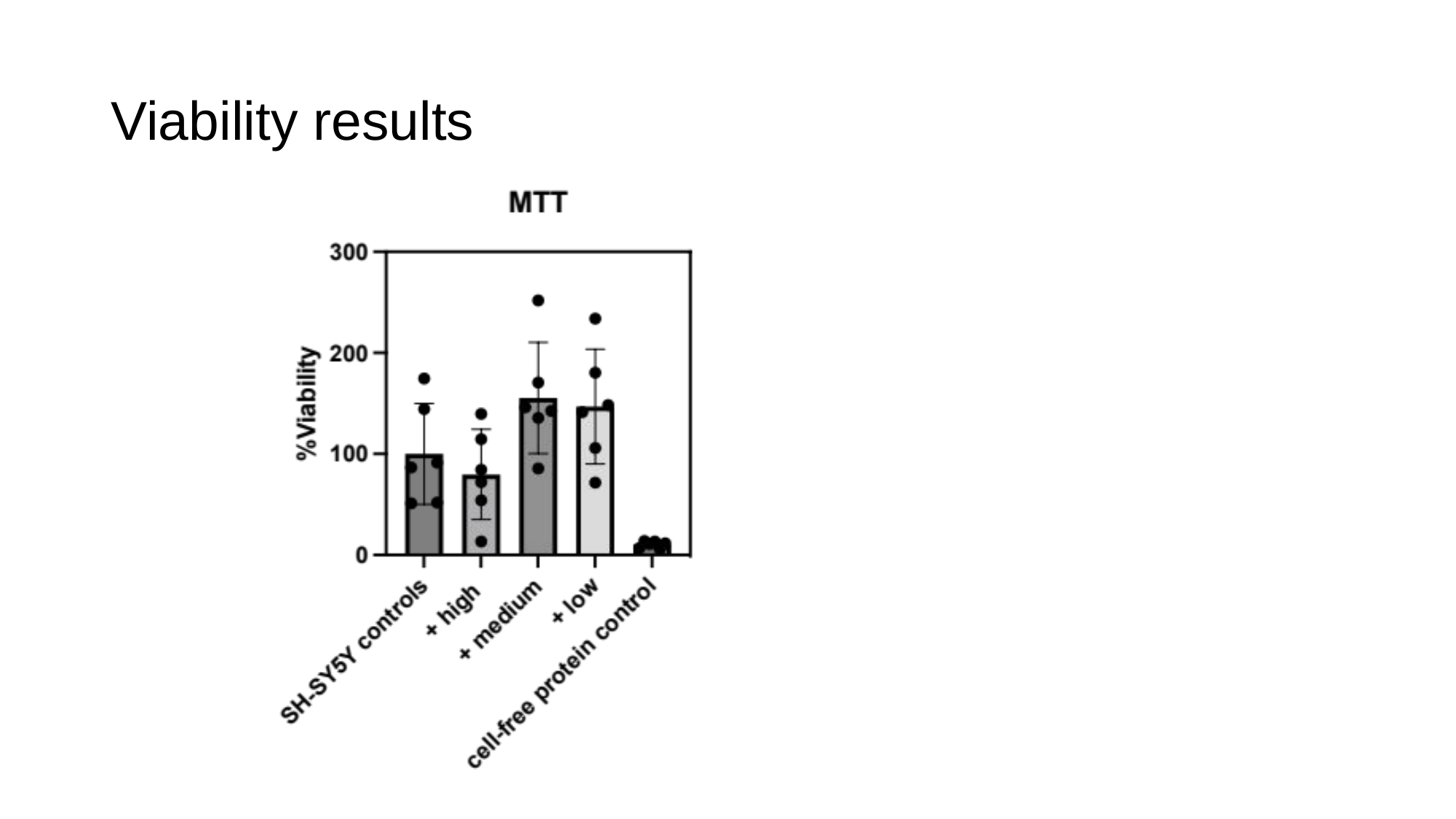

# Viability results

## Slide 6
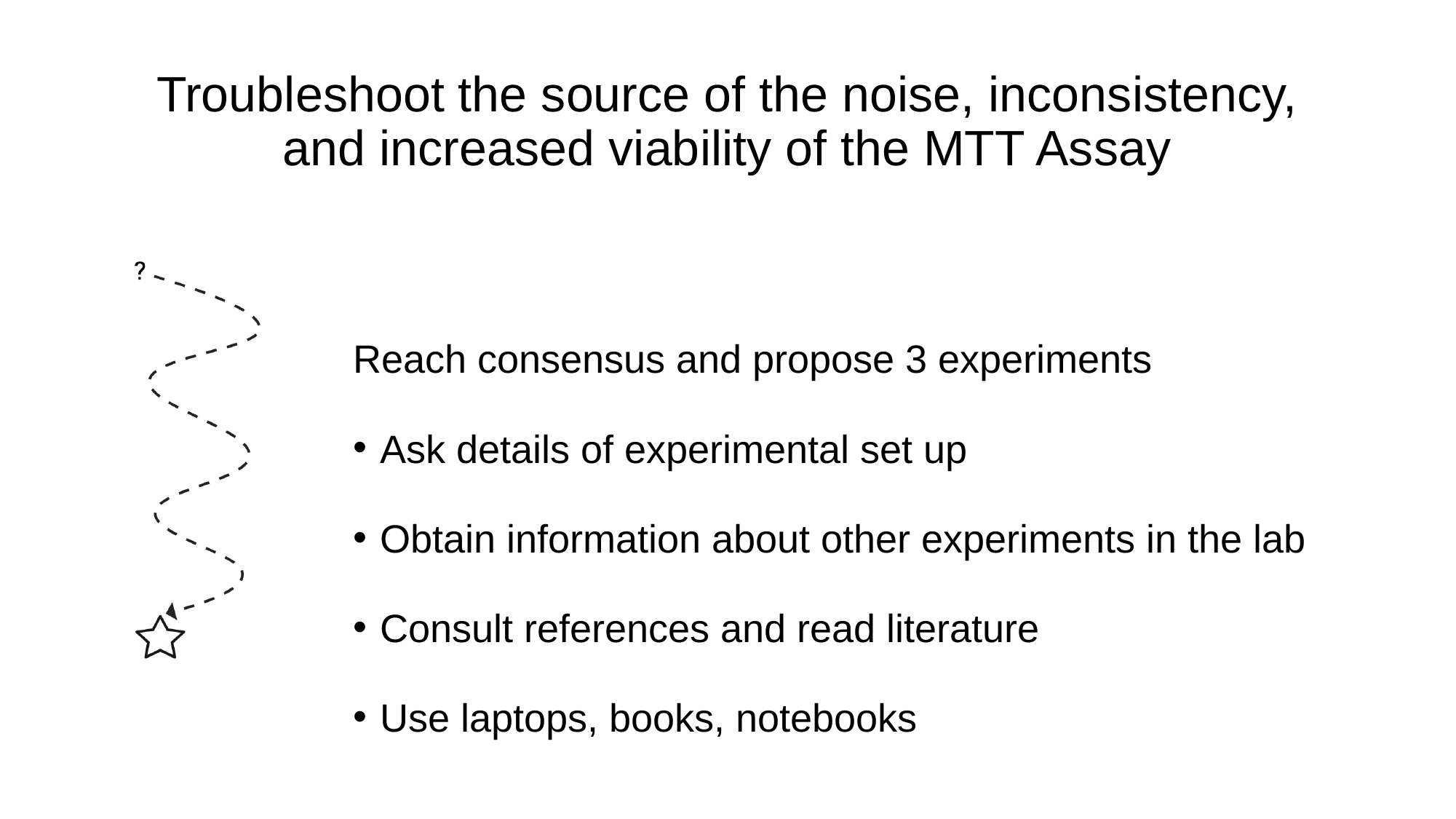

# Troubleshoot the source of the noise, inconsistency, and increased viability of the MTT Assay
Reach consensus and propose 3 experiments
Ask details of experimental set up
Obtain information about other experiments in the lab
Consult references and read literature
Use laptops, books, notebooks

## Slide 7
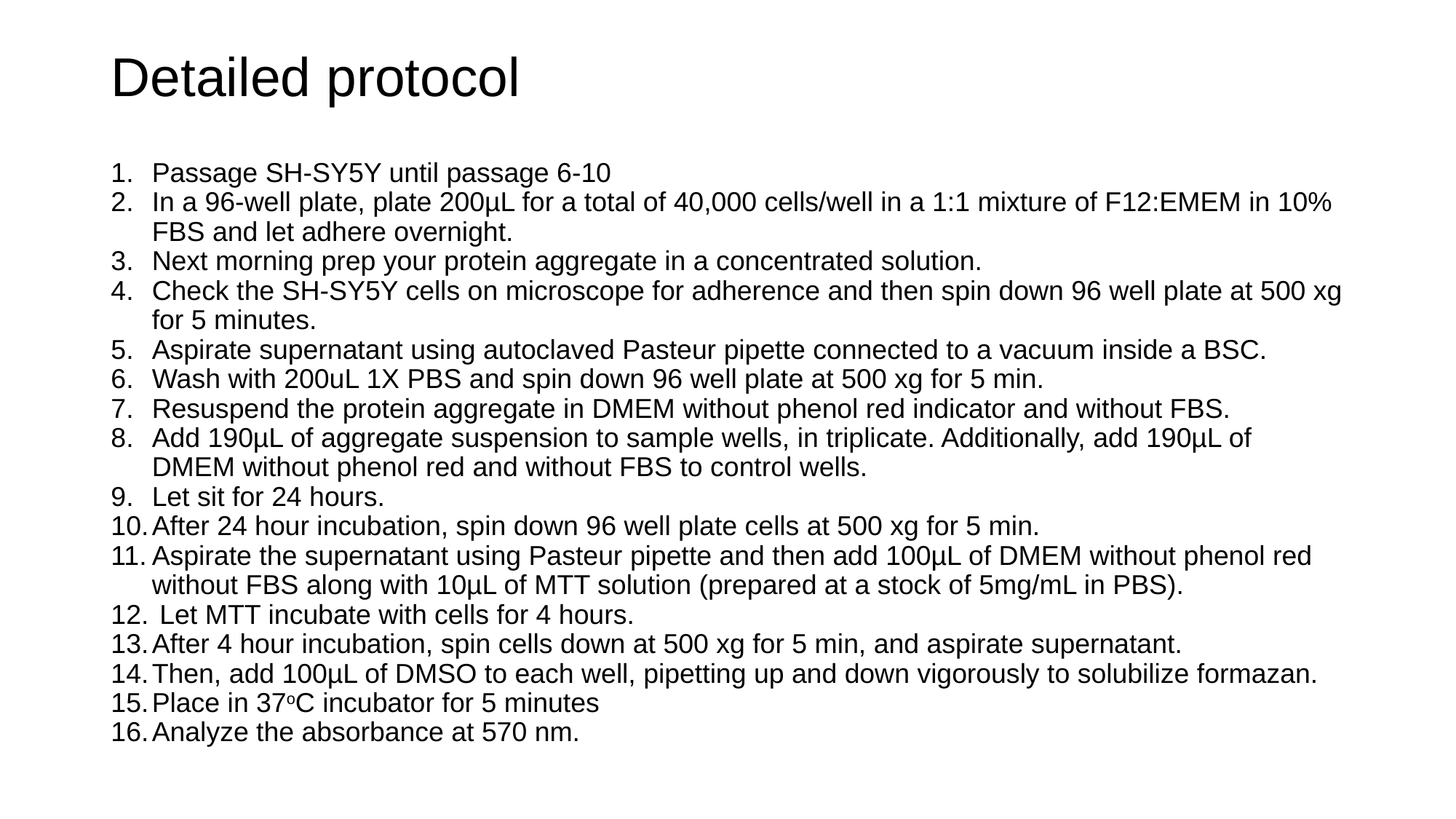

# Detailed protocol
Passage SH-SY5Y until passage 6-10
In a 96-well plate, plate 200µL for a total of 40,000 cells/well in a 1:1 mixture of F12:EMEM in 10% FBS and let adhere overnight.
Next morning prep your protein aggregate in a concentrated solution.
Check the SH-SY5Y cells on microscope for adherence and then spin down 96 well plate at 500 xg for 5 minutes.
Aspirate supernatant using autoclaved Pasteur pipette connected to a vacuum inside a BSC.
Wash with 200uL 1X PBS and spin down 96 well plate at 500 xg for 5 min.
Resuspend the protein aggregate in DMEM without phenol red indicator and without FBS.
Add 190µL of aggregate suspension to sample wells, in triplicate. Additionally, add 190µL of DMEM without phenol red and without FBS to control wells.
Let sit for 24 hours.
After 24 hour incubation, spin down 96 well plate cells at 500 xg for 5 min.
Aspirate the supernatant using Pasteur pipette and then add 100µL of DMEM without phenol red without FBS along with 10µL of MTT solution (prepared at a stock of 5mg/mL in PBS).
 Let MTT incubate with cells for 4 hours.
After 4 hour incubation, spin cells down at 500 xg for 5 min, and aspirate supernatant.
Then, add 100µL of DMSO to each well, pipetting up and down vigorously to solubilize formazan.
Place in 37oC incubator for 5 minutes
Analyze the absorbance at 570 nm.

## Slide 8
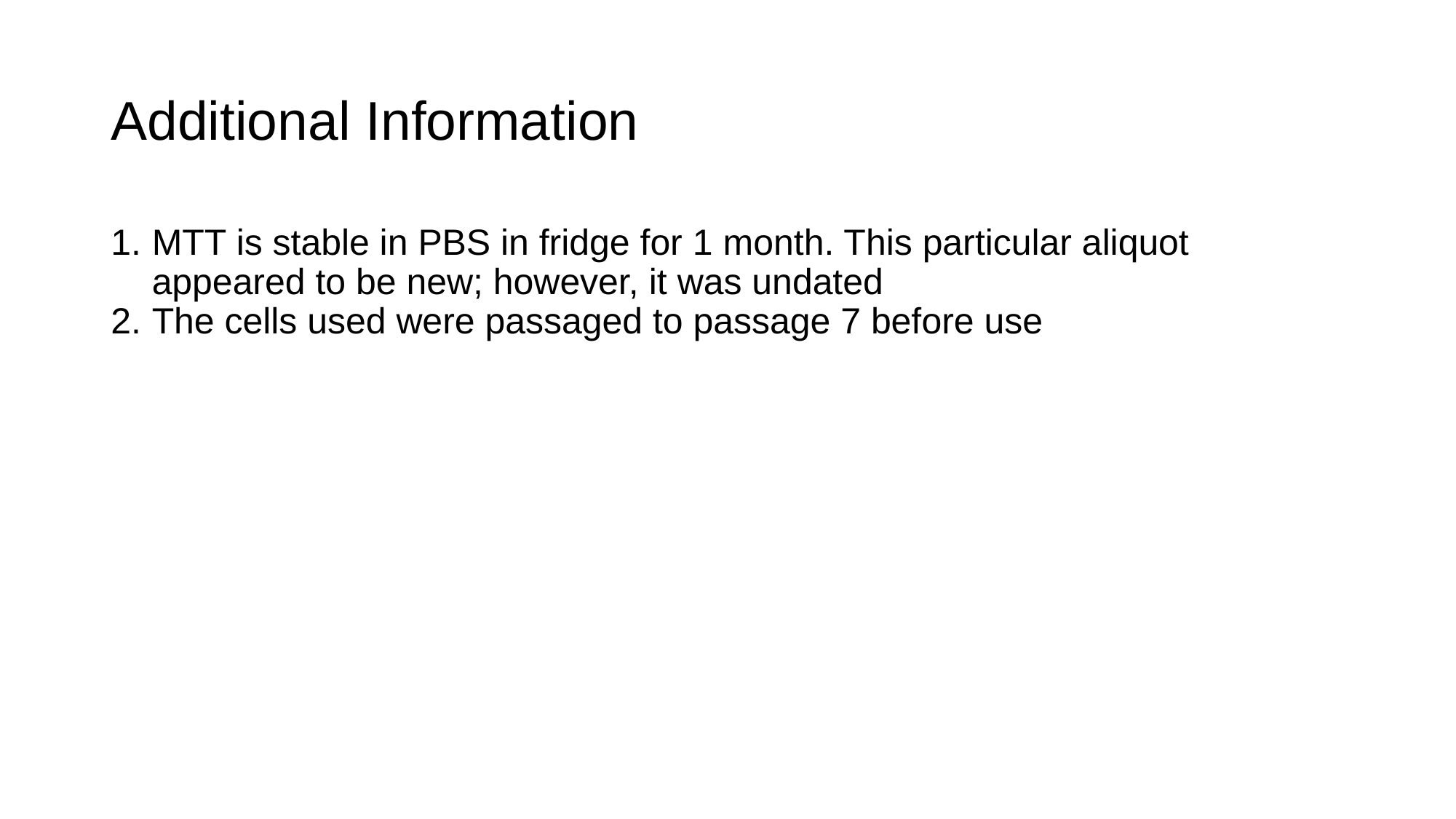

# Additional Information
MTT is stable in PBS in fridge for 1 month. This particular aliquot appeared to be new; however, it was undated
The cells used were passaged to passage 7 before use

## Slide 9
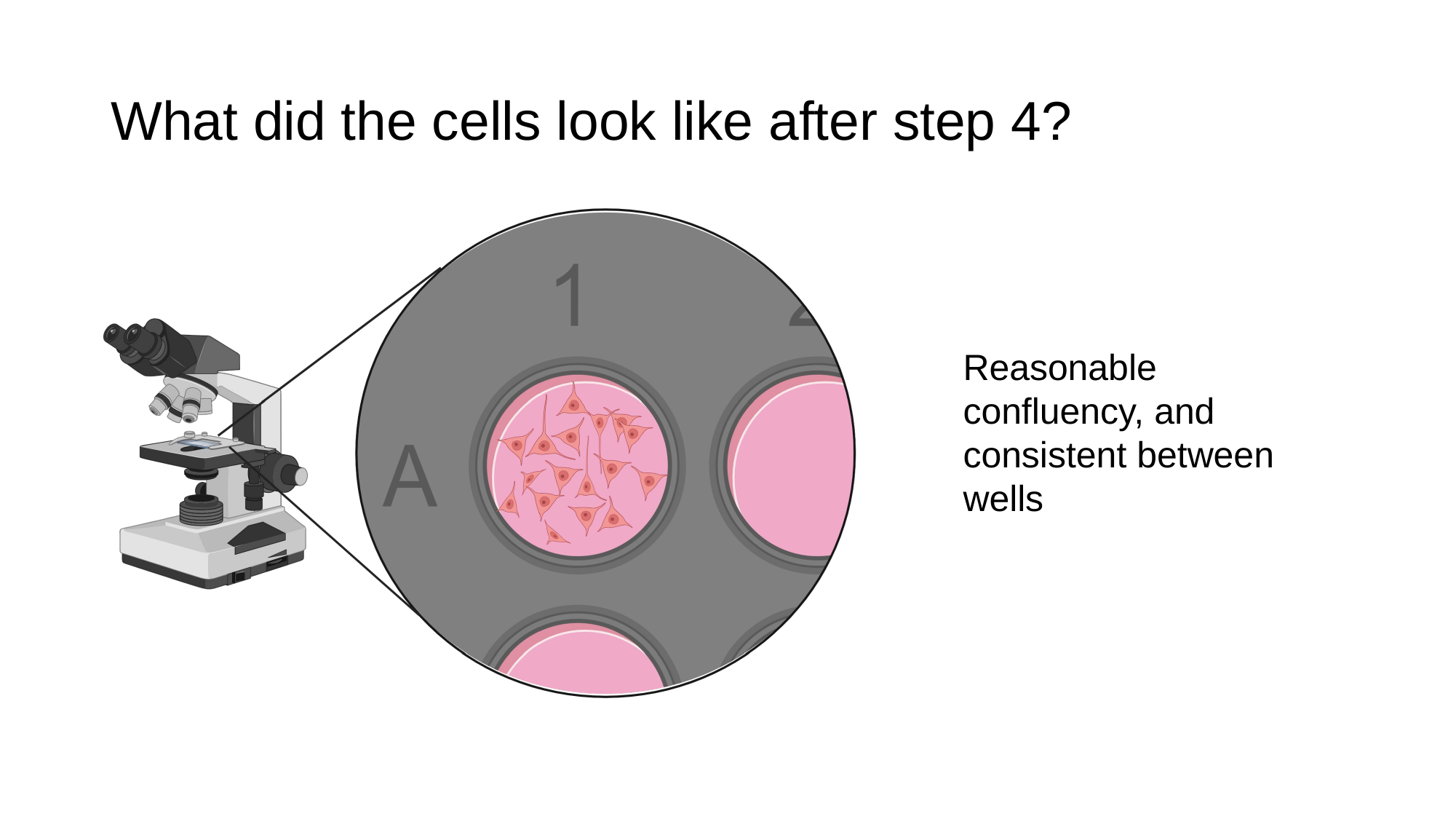

# What did the cells look like after step 4?
Reasonable confluency, and consistent between wells

## Slide 10
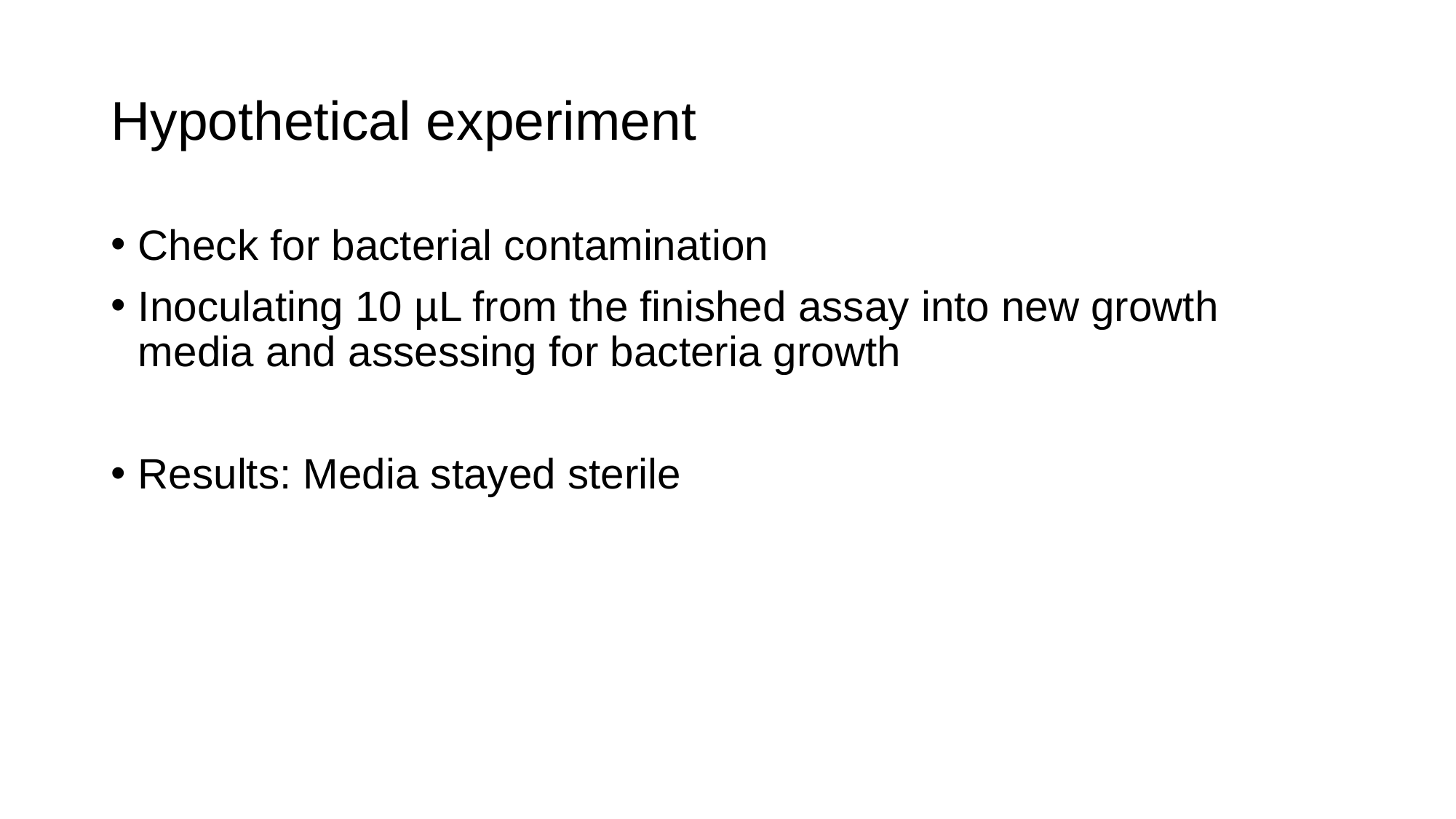

# Hypothetical experiment
Check for bacterial contamination
Inoculating 10 µL from the finished assay into new growth media and assessing for bacteria growth
Results: Media stayed sterile

## Slide 11
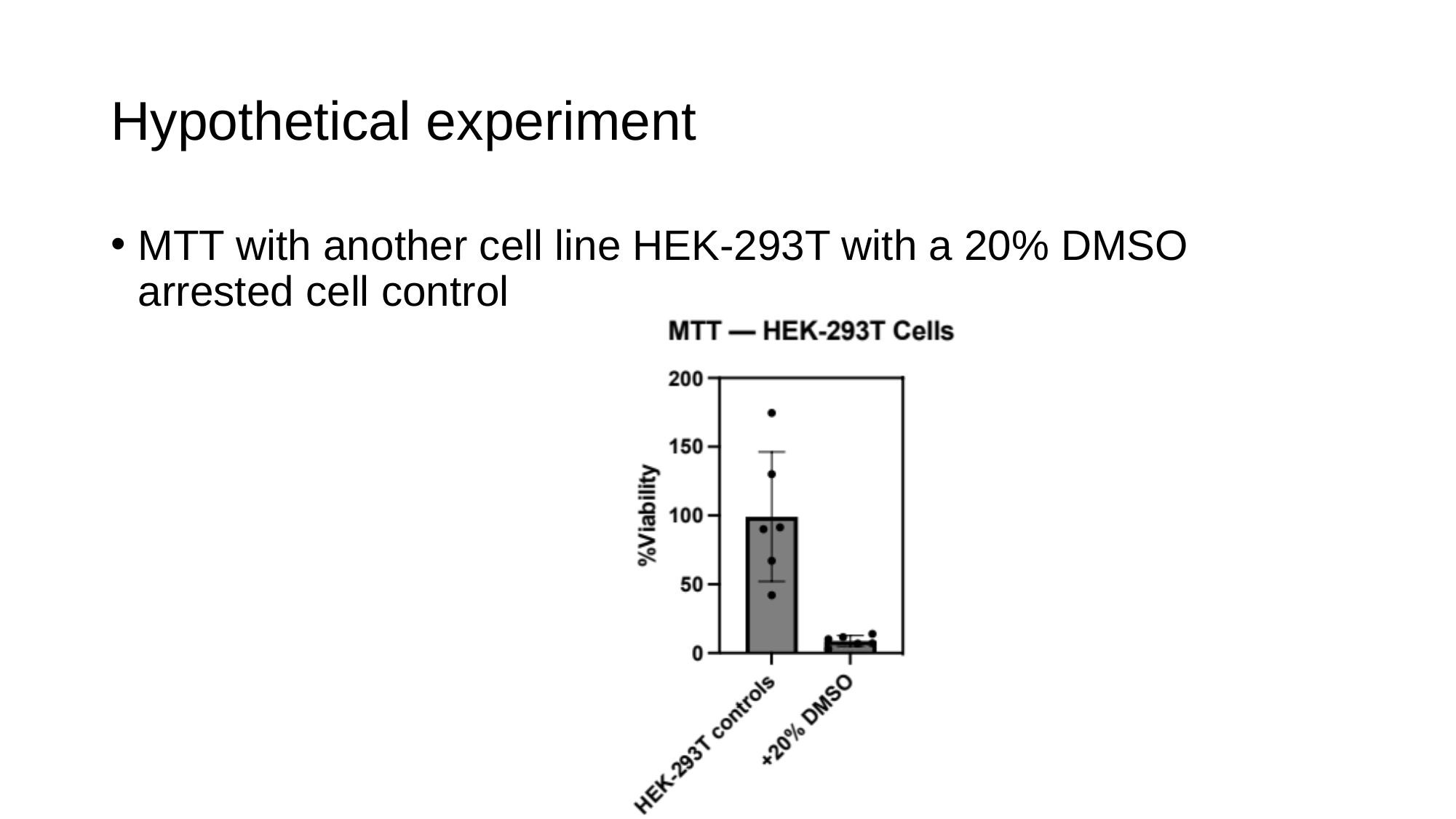

# Hypothetical experiment
MTT with another cell line HEK-293T with a 20% DMSO arrested cell control

## Slide 12
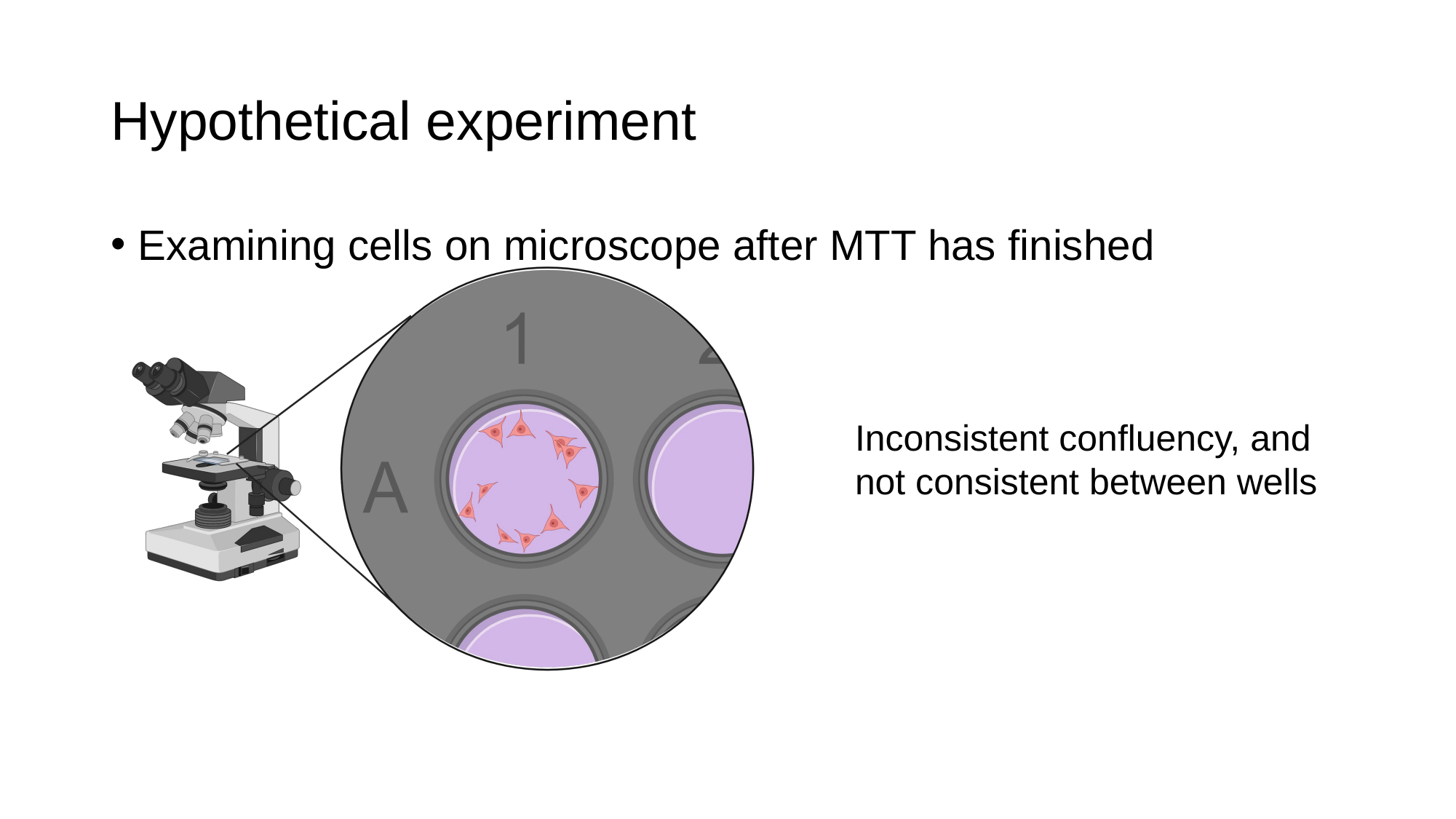

# Hypothetical experiment
Examining cells on microscope after MTT has finished
Inconsistent confluency, and not consistent between wells

## Slide 13
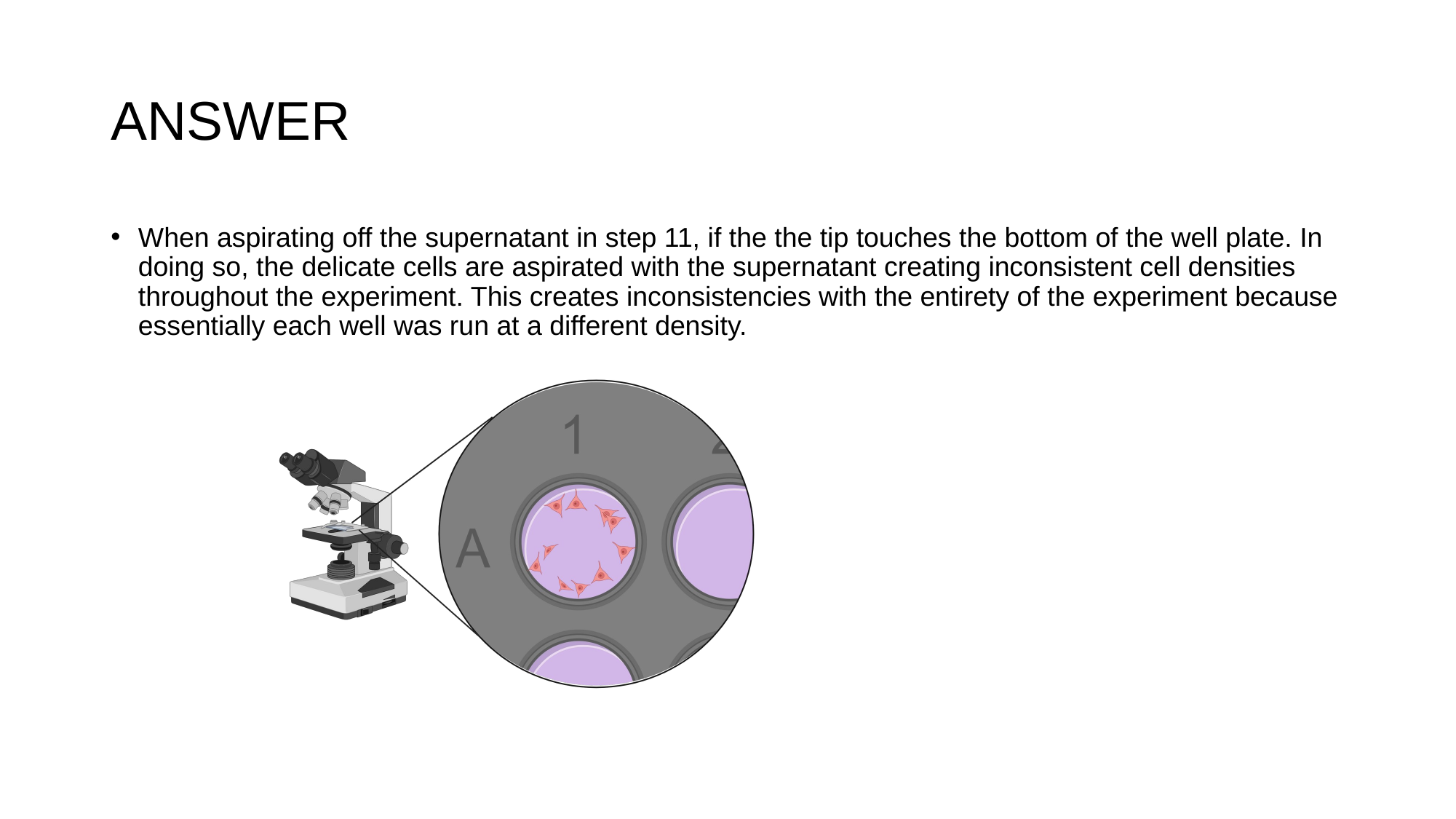

# ANSWER
When aspirating off the supernatant in step 11, if the the tip touches the bottom of the well plate. In doing so, the delicate cells are aspirated with the supernatant creating inconsistent cell densities throughout the experiment. This creates inconsistencies with the entirety of the experiment because essentially each well was run at a different density.
