# Supplementary material for: Teaching troubleshooting skills to graduate students
Source: eLife. 2024 Sep 17;13:e100761. doi: 10.7554/eLife.100761 (PMC11407763; doi:10.7554/eLife.100761)
Supplement: Supplementary file 1. — For each scenario there is a Word file that contains the following: background information; a description of the scenario; the protocol for the experiment that produced the unexpected result; the results of the experiment; information on the source of the error; background information that can be used to answer questions; and references. There is also a PowerPoint file for each scenario that contains example slides that can be used in real meetings. There are also templates for the Word and PowerPoint files. [file elife-100761-supp1.zip › Final Scenarios/Example7.docx]

**T cell cytokine release ELISA**

Keywords: Mammalian cell biology, T cell activation, Cytokine release

**Background:**

There are various methods to monitor T cell activation: upregulation of activation markers by flow cytometry (CD69, CD25, etc. ), cytotoxicity (LDH , calcein release, etc.), proliferation (CFSE or other cell staining dyes), cytokine release (IL-2, IL-6, TNF-α) , INF-γ, etc.), and others ^1–3^. Cytokine release in particular is important to study to characterize early drug candidates due to the high occurrence of cytokine release syndrome (CRS) in the clinic ^2,4,5^. While there are various ways to characterize cytokines release, the simplest are ELISAs designed to capture and detect levels of specific cytokine targets. Here we discuss the use of a commercially available human IL-2 ELISA kit (BioLegend 431801)^3,4^ . The assays kit includes an initial capture antibody against IL2, a secondary detection antibody against IL-2 with a biotin tag, and then a tertiary avidin (binds to biotin) tag bound to horseradish peroxidase (HRP) enzyme which can be quantified through the addition of TMB and read on a common plate reader.

**Scenario:**

You are a researcher interested in characterizing the cytokine release from T cells in the presence of a T cell engager bispecific antibody and target cells. To do this you co-incubate the cells and protein, remove the media, and complete the IL-2 ELISA. However, you notice the variability is wildly different between different samples and that the top concentration of each sample seems oddly high, leading to inconclusive data.

Note: Typically, this protocol involves a step to convert the raw plate reader absorbance to a pg/mL concentration using a standard. For simplicity, data will remain in raw absorbance form in this example.

**Protocol:**

1. The day before setting up the experiment, coat an ELISA plate with Detection antibody.
2. The next day, dilute target cells to 1x 10^6^ cells/mL in T cell media
3. Dilute bispecific in a separate plate to 4X final concentration in 50 µL in T cell media
4. Dilute primary T cells to 5 x 10^5^ cells/mL in T cell media
5. Add 50 µL target cells, 50 µL bispecific solution, and 100 µL of T cells (5 x 10^4^ total T cells, 5 x 10^4^ total target cells, 1 X final bispecific concentration) to each well in a U-bottom tissue culture treated 96-well plate
6. Incubate at 37°C, 5% CO_2_ for 48 hrs
7. Spin down plate at 250 g for 5 minutes and remove supernatant to a fresh untreated 96-well plate
8. Wash the ELISA plate, add 50 µL of supernatant, and incubate for 2 hours.
9. Wash the ELISA plate, add Detection antibody, and incubate for 45 minutes.
10. Wash the ELISA plate, add Avidin-HRP, and incubate 30 min.
11. Wash the ELISA plate, add 50 µL TMB.
12. Quench with 50 µL 1N HCl.
13. Read absorbance at 450 nm.

**Table 1.** Sample layout

|  | **1** | **2** | **3** | **4** | **5** | **6** | **7** | **8** | **9** | **10** | **11** | **12** |
| --- | --- | --- | --- | --- | --- | --- | --- | --- | --- | --- | --- | --- |
| **A** | S1-1 | S1-2 | S1-3 | S1-4 | S1-5 | S1-6 | S1-7 | S1-8 | S5 |  |  |  |
| **B** | S1-1 | S1-2 | S1-3 | S1-4 | S1-5 | S1-6 | S1-7 | S1-8 | S5 |  |  |  |
| **C** | S2-1 | S2-2 | S2-3 | S2-4 | S2-5 | S2-6 | S2-7 | S2-8 |  |  |  |  |
| **D** | S2-1 | S2-2 | S2-3 | S2-4 | S2-5 | S2-6 | S2-7 | S2-8 |  |  |  |  |
| **E** | S3-1 | S3-2 | S3-3 | S3-4 | S3-5 | S3-6 | S3-7 | S3-8 |  |  |  |  |
| **F** | S3-1 | S3-2 | S3-3 | S3-4 | S3-5 | S3-6 | S3-7 | S3-8 |  |  |  |  |
| **G** | S4-1 | S4-2 | S4-3 | S4-4 | S4-5 | S4-6 | S4-7 | S3-8 |  |  |  |  |
| **H** | S4-1 | S4-2 | S4-3 | S4-4 | S4-5 | S4-6 | S4-7 | S3-8 |  |  |  |  |

S1-1, Sample 1 Concentration 1

ST-1, IL-2 Standard Concentration 1

**Sample list:**

1. High affinity bispecific
2. Medium-high affinity bispecific
3. Medium affinity bispecific
4. Low affinity bispecific
5. No bispecific control

Concentrations start at 300 nM and were diluted 3-fold.

**Example graphs and experimental outcomes**

**Table 2.** Absorbance at 450 nm.

|  | **1** | **2** | **3** | **4** | **5** | **6** | **7** | **8** | **9** | **10** | **11** | **12** |
| --- | --- | --- | --- | --- | --- | --- | --- | --- | --- | --- | --- | --- |
| **A** | 2.234 | 2.295 | 2.282 | 2.133 | 1.506 | 1.034 | 0.585 | 0.375 | 0.232 |  |  |  |
| **B** | 2.254 | 1.490 | 1.480 | 1.452 | 1.129 | 0.710 | 0.371 | 0.241 | 0.229 |  |  |  |
| **C** | 2.292 | 1.482 | 1.120 | 0.760 | 0.460 | 0.380 | 0.327 | 0.233 |  |  |  |  |
| **D** | 2.265 | 1.490 | 1.025 | 0.782 | 0.458 | 0.361 | 0.331 | 0.235 |  |  |  |  |
| **E** | 2.247 | 1.320 | 0.751 | 0.467 | 0.351 | 0.289 | 0.267 | 0.255 |  |  |  |  |
| **F** | 2.265 | 1.219 | 0.782 | 0.459 | 0.331 | 0.293 | 0.236 | 0.222 |  |  |  |  |
| **G** | 1.845 | 0.634 | 0.378 | 0.280 | 0.242 | 0.221 | 0.253 | 0.235 |  |  |  |  |
| **H** | 1.730 | 1.007 | 0.534 | 0.417 | 0.350 | 0.335 | 0.290 | 0.302 |  |  |  |  |

This hypothetical data was generated and is not a reflection of a real experiment. It should not be cited, used, or interpreted in anyway. It is solely for a training exercise.


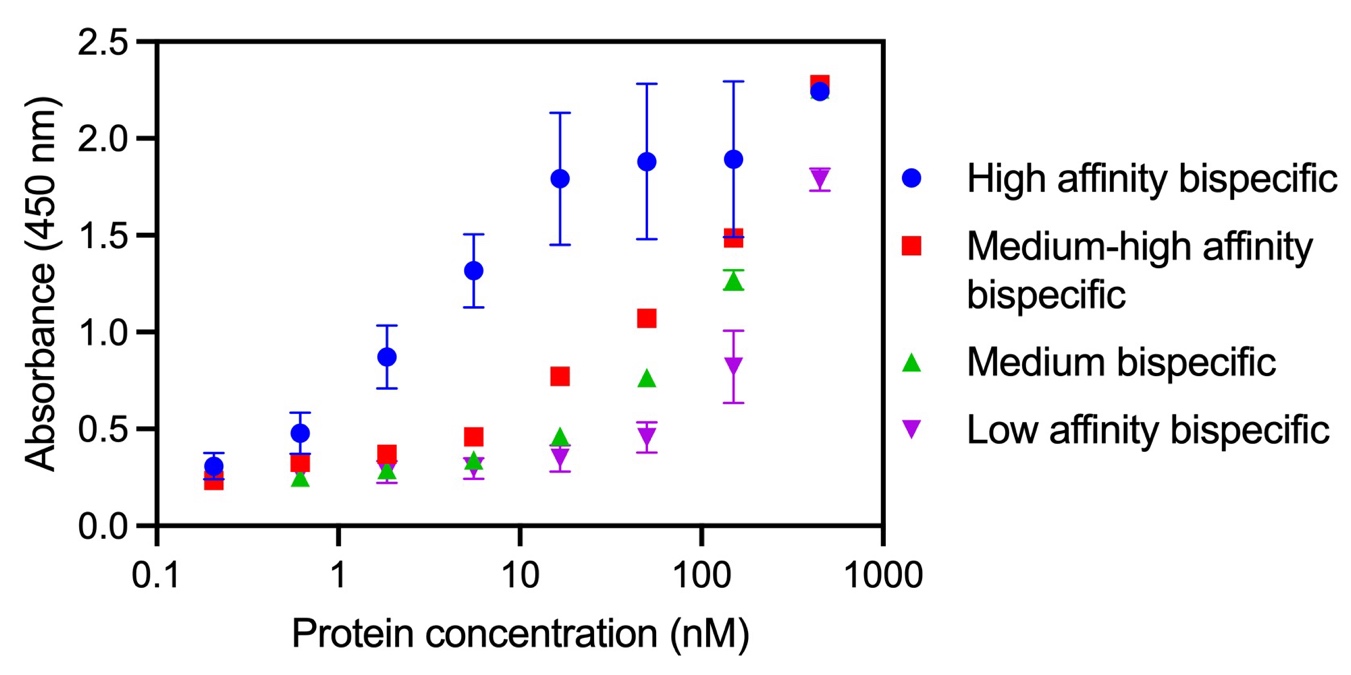


**Figure 1.** Example plotted data from hypothetical experiment.

**Details:**

1. Bispecific protein were new single use aliquots thawed ~15 min before setting up experiment.
2. Primary T cells were 42% CD4 and 55% CD8.
3. The plate was placed in the back of the middle shelf in the incubator.
4. Target cells were passaged to passage 11 before use.
5. The plate reader is commonly used, approximately 4-5 times per day.

**Source of error:**

During the 48-hour incubation in at 37°C, wells toward the edge of the plate experience more evaporation that wells toward the middle of the plate, effectively concentrating the supernatant and resulting cytokine concentration in wells on the edge of the plate. Possible solutions include adding PBS between wells on the plate, using parafilm to cover the border between plate and lid, using aeropore sheets to reduce evaporation, or excluding use of the outer wells of the plate. Example data after using these strategies is shown below.

**Table 1.** Additional information known by the leader that can be provided upon request

| **Meeting Notes for the Leader**  Not to be shared with the group | |
| --- | --- |
| Other researcher’s experiments | - Other cell lines growing: SK-BR-3 cancer cells, MRC-5 fibroblasts, Raji cells, Jurkat cells, THP-1 cells, NK-92 - Flow cytometry staining of cancer cells - ELISAs with various protein-protein binding interactions. |
| Storage information | - Bispecific proteins were stored in PBS at -80C, detection antibodies and cell culture media were stored at 4C, and ELISA plates and other buffers were stored at room temperature. |
| Sample information | - This is the first characterization of these particular bispecific antibodies |
| Source of error | - Edge effects: when the plate incubates at 37C, liquid evaporates faster on the outer edges than the inside wells leading to high error and artificially high concentrations of molecules in outer wells |
| Hints for group | - Consider the physical forces on the samples throughout the procedure. |

**References:**

1. Liddy, N. *et al.* Monoclonal TCR-redirected tumor cell killing. *Nat Med* **18**, 980–987 (2012).

2. Santich, B. H. *et al.* Interdomain spacing and spatial configuration drive the potency of IgG-[L]-scFv T cell bispecific antibodies. *Science Translational Medicine* **12**, eaax1315 (2020).

3. Warmuth, S. *et al.* Engineering of a trispecific tumor-targeted immunotherapy incorporating 4-1BB co-stimulation and PD-L1 blockade. *Oncoimmunology* **10**, 2004661 (2021).

4. Fierle, J. K. *et al.* Soluble trivalent engagers redirect cytolytic T cell activity toward tumor endothelial marker 1. *Cell Reports Medicine* **2**, 100362 (2021).

5. Li, J. *et al.* CD3 bispecific antibody–induced cytokine release is dispensable for cytotoxic T cell activity. *Science Translational Medicine* **11**, eaax8861 (2019).
