# Supplementary material for: Teaching troubleshooting skills to graduate students
Source: eLife. 2024 Sep 17;13:e100761. doi: 10.7554/eLife.100761 (PMC11407763; doi:10.7554/eLife.100761)
Supplement: Supplementary file 1. — For each scenario there is a Word file that contains the following: background information; a description of the scenario; the protocol for the experiment that produced the unexpected result; the results of the experiment; information on the source of the error; background information that can be used to answer questions; and references. There is also a PowerPoint file for each scenario that contains example slides that can be used in real meetings. There are also templates for the Word and PowerPoint files. [file elife-100761-supp1.zip › Final Scenarios/Example4.docx]

**Golden Gate Cloning**

**Keywords:** Cloning; Golden Gate Assembly; PCR; primers; synthetic biology.

**Background:**

Golden Gate Assembly is a molecular cloning method that utilizes Type IIS restriction enzymes and T4 DNA ligase to assemble multiple DNA fragments into a plasmid in a single reaction^1^. This method is especially effective for quickly and accurately assembling genetic constructs. The process entails cutting DNA fragments (insert) and a vector (backbone) with a Type IIS restriction enzyme to create predefined overhangs for seamless assembly^2^. These fragments are then ligated by T4 DNA ligase in a one-pot reaction that allows for the simultaneous digestion and ligation of multiple components^3^. Polymerase chain reaction (PCR) is used to amplify vector backbone and insert DNA fragments, ensuring sufficient quantities and compatibility with the enzymes^4^.

Appropriate primer design is crucial for the success of PCR amplification, as it ensures that the generated fragments precisely match the requirements of the assembly system, further enhancing the overall efficiency and fidelity of the cloning process.

**Scenario:**

As a researcher, your goal was to replace the gene ABC (Figure 1) with the *sfgfp* gene (Figure 2) in a plasmid via Golden Gate assembly. The assembly was designed using the Benchling software (Figure 3). To this end, specific primers were designed and utilized to amplify both the insert and vector backbone sequences through polymerase chain reaction (PCR). After the PCR, the products were subjected to DpnI enzymatic digestion followed by a PCR clean-up procedure. A DPNI enzymatic reaction is designed to digest the methylated DNA backbone, and a PCR clean-up removes buffers and unreacted nucleotides. The prepared fragments were then assembled using the Golden Gate method. The resultant construct was transformed into *Pseudomonas aeruginosa* PAO1 and plated on LB agar plates supplemented with the appropriate antibiotic. Unfortunately, no colonies were observed after an incubation period of 24 hours at 37 °C.

**Protocol:**

1. **Assembly Design**: Utilize Benchling design software for creating your assembly designs. Access the tool at www.benchling.com.
2. **Amplification of fragments**: In a sterile PCR tube, add the following reagents.

| **Reagent (stock conc.)** | **Volume (µL)** |
| --- | --- |
| Q5 High-Fidelity 2X Master Mix | 25 |
| Forward Primer (10 µM) | 2.5 |
| Reverse Primer (10 µM) | 2.5 |
| Template DNA | 1 |
| Nuclease-free Water | 19 |

PCR Cycling Conditions:

| **Step (# of cycles)** | **Temperature (°C)** | | **Timing (s)** |
| --- | --- | --- | --- |
| Initial denaturation (1x) | 98 | | 30 |
| Denaturation Annealing/Extension (25x) | 98 | | 5 |
|  | Backbone | Tm = 66.7 | 15 |
|  | Insert | Tm = 63.2 |  |
|  | 72 | | 68 s for Backbone |
|  |  |  | 30 s for insert |
| Final Amplification/Cooling (1x) | 72 | | 120 |
|  | 4 | | Infinity |

1. **Digest template using DpnI**: Add 1 µL of DpnI enzyme (New England Biolabs) to 50 µL of your PCR product directly in the PCR tube.
2. **Cleanup PCR reaction:** Perform a PCR cleanup using QIAquick PCR purification kit (Qiagen). After elution, Nanodrop your sample to make sure of the purity of your sample and to know the concentration.
3. **Golden Gate Assembly:** Calculate the required volumes using the Barrick Lab’s BroadHostRange_Reaction_Calculator, available [here](https://barricklab.org/twiki/bin/view/Lab/GoldenGateAssemblyProtocolsMainPage).

Reaction Setup:

| **Reagent** | **Volume (µL)** |
| --- | --- |
| dH_2_O | 15.27 |
| Backbone | 0.75 |
| Insert | 0.98 |
| T4 DNA Ligase Buffer 10x | 2.00 |
| T4 DNA Ligase | 0.50 |
| BsmBI | 0.50 |
| Total Reaction Volume | 20.00 |

Assembly Cycling Conditions:

| **Cycling Conditions** | |  |
| --- | --- | --- |
| 42 °C | 1.5 min | 25x |
| 16 °C | 3 min |  |
| 55 °C | 5 min | 1x |
| 80 °C | 10 min | 1x |
| 4 °C | Infinite | 1x |

1. **Transformation**: Mix 1 μL of the Golden Gate reaction with 30 μL of electrocompetent *P. aeruginosa* PAO1, transfer to a 1 mm electroporation cuvette, and electroporate at 1600 V. Immediately add 1 mL of LB to the electroporated cells, incubate and shake at 37°C and 250 rpm for 2 hours; then plate 100 μL of the cell suspension onto LB agar plates containing 34 μg/mL chloramphenicol and incubate overnight at 37°C.
2. **Verification:** Look for single colonies.

**Source of error:**

The vector backbone contained two similar terminators T0 at different locations (Figure 2). During the design of the forward primer intended to attach to the terminator following the ABC gene, it was found that this primer also shared a sequence with the Terminator T0 positioned after the chloramphenicol resistance gene (Figure 3). Consequently, the primer bound to both terminator sites, resulting in PCR products of incorrect size (1285 bp instead of the expected 2262 bp) (Figure 4).

**Figures:**


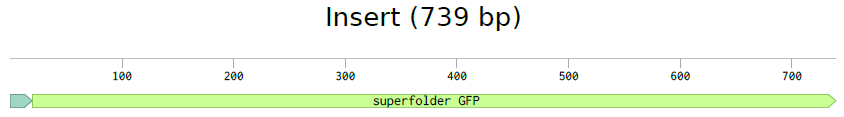


**Figure 1**: Representation of insert fragment (Read-Only Benchling Link: [Insert](https://benchling.com/s/seq-OpcfzITTASGYsT4HxSFc?m=slm-rdRMrIglbaEQHsGsKVZ1)).


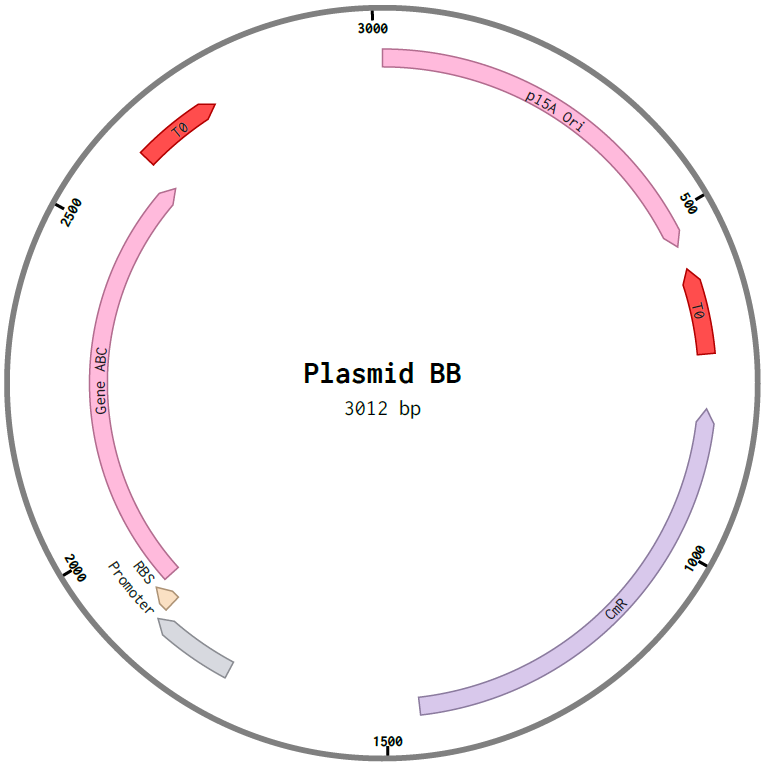


**Figure 2**: Representation of backbone plasmid (Read-Only Benchling Link: [BB_Plasmid](https://benchling.com/s/seq-b5lR24LwTwxZrS3QpPje?m=slm-MyRdvcYtu6FXmF6ZPLcG))


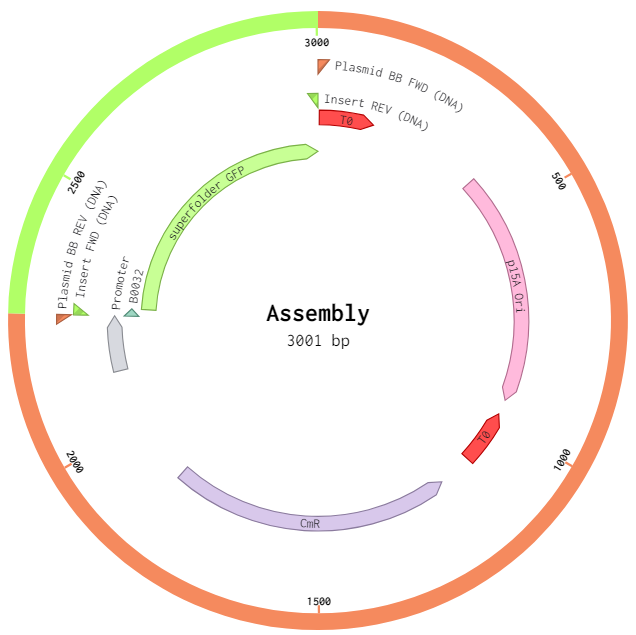


**Figure 3**: Representation of Golden Gate Assembly (Read-Only Benchling Link: [GG_Assembly](https://benchling.com/s/seq-4DqqJM6217mDHbBpkwZv?m=slm-LL0pq05nG05eH4Svk1uw)).

**Example Figures and experimental outcomes**


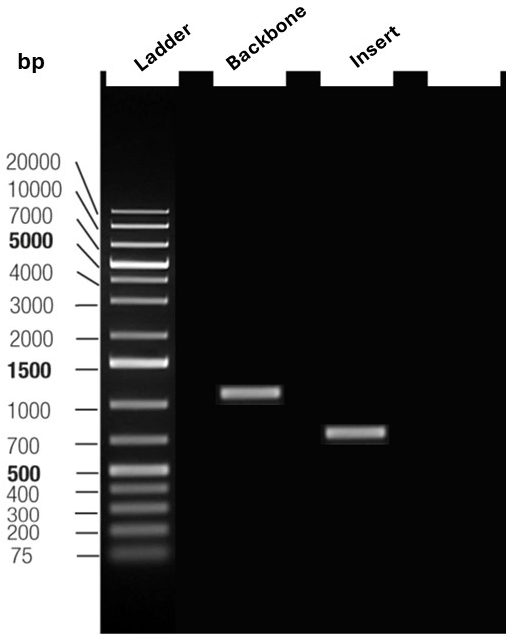


**Figure 4**: Example of Agarose gel electrophoresis results show PCR product sizes for backbone (1285 bp) and insert (739 bp).

**Table 1.** Additional information known by the leader that can be provided upon request

| **Meeting Notes for the Leader**  Not to be shared with the group | |
| --- | --- |
| Other researcher’s experiments | - Researcher 1 was conducting multiple gel electrophoresis runs for colony PCR samples prepared the previous day. - Researcher 2 was using the thermocycler with you to perform DpnI digestion and Golden Gate assembly, which later resulted in successful colony formation upon transformation. |
| Storage information | - All PCR reagents and Golden Gate enzymes were new and have been stored in the -20 °C freezer. |
| Plasmid information | - The plasmid used as the backbone contains the ABC gene, which is intended to be replaced by the GFP gene. It also includes a chloramphenicol resistance gene and a p15A origin of replication. |
| Source of error | - The vector backbone contained two similar terminators T0 at different locations. During the design of the forward primer intended to attach to the terminator following the ABC gene, it was found that this primer also shared a sequence with the Terminator T0 positioned after the chloramphenicol resistance gene. Consequently, the primer bound to both terminator sites, resulting in PCR products of incorrect size (1285 bp instead of the expected 2262 bp). |
| Hints for group | - Checking for primer complementarity to the template DNA region is important before proceeding. |

**Reference :**

(1) Engler, C.; Gruetzner, R.; Kandzia, R.; Marillonnet, S. Golden Gate Shuffling: A One-Pot DNA Shuffling Method Based on Type Ils Restriction Enzymes. *PLoS ONE* **2009**, *4* (5). https://doi.org/10.1371/journal.pone.0005553.

(2) Casini, A.; Storch, M.; Baldwin, G. S.; Ellis, T. Bricks and Blueprints: Methods and Standards for DNA Assembly. *Nat. Rev. Mol. Cell Biol.* **2015**, *16* (9), 568–576. https://doi.org/10.1038/nrm4014.

(3) Weber, E.; Engler, C.; Gruetzner, R.; Werner, S.; Marillonnet, S. A Modular Cloning System for Standardized Assembly of Multigene Constructs. *PLoS ONE* **2011**, *6* (2). https://doi.org/10.1371/journal.pone.0016765.

(4) Bird, J. E.; Marles-Wright, J.; Giachino, A. A User’s Guide to Golden Gate Cloning Methods and Standards. *ACS Synth. Biol.* **2022**, *11* (11), 3551–3563. https://doi.org/10.1021/acssynbio.2c00355.
