# Supplementary material for: Teaching troubleshooting skills to graduate students
Source: eLife. 2024 Sep 17;13:e100761. doi: 10.7554/eLife.100761 (PMC11407763; doi:10.7554/eLife.100761)
Supplement: Supplementary file 1. — For each scenario there is a Word file that contains the following: background information; a description of the scenario; the protocol for the experiment that produced the unexpected result; the results of the experiment; information on the source of the error; background information that can be used to answer questions; and references. There is also a PowerPoint file for each scenario that contains example slides that can be used in real meetings. There are also templates for the Word and PowerPoint files. [file elife-100761-supp1.zip › Final Scenarios/Example8.docx]

**Metabolite tracking by aqueous Fourier-transform infrared spectroscopy**

Keywords: spectroscopy, analytical chemistry, FTIR, synthetic biology

**Background:**

Fourier-transform infrared spectroscopy (FTIR) can rapidly quantify the concentration of organic solutes in liquid samples^1^. In biological systems, FTIR can track growth by measurement of substrate and metabolite concentrations in the aqueous medium^2^, providing an alternative to methods such as optical density (OD) in systems where OD measurement is infeasible. Additionally, only small liquid aliquots (~20 µL) are required when FTIR is configured for use with attenuated total reflectance (ATR), such that cultures are minimally disturbed by sample collection. While cultures contain a complex mix of metabolites and media components, the concentrations of individual metabolites can be obtained by linear combination of the absorbance spectra for each component^3^. In regions of the spectrum where peaks are present for only one metabolite, an approximate fit can be made simply by fitting the peak height to a standard curve.

**Scenario:**

You are using Fourier-transform infrared spectroscopy (FTIR) to measure metabolite concentrations in real time (in this case, acetic acid) in order to track growth. To do so, you place droplets of your solution on a diamond crystal and measure absorbance using attenuated total reflectance (ATR), and use deionized water to measure a background absorbance spectrum.


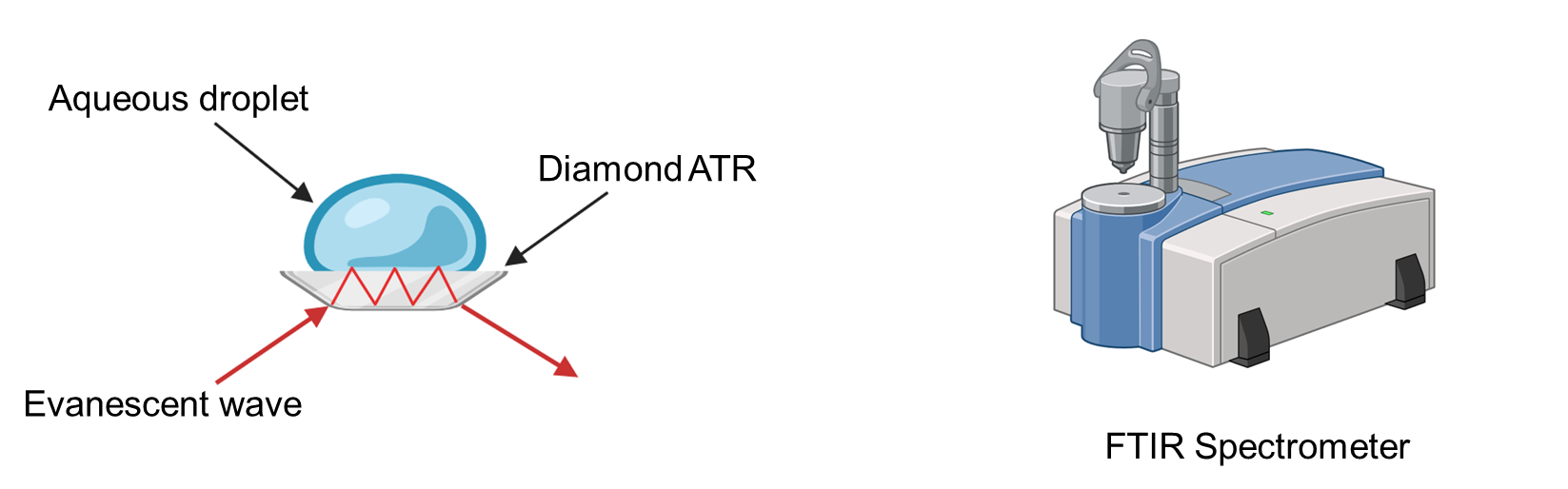


**Figure 1.** ATR schematic (left) and FTIR Spectrometer (right)

From prior experiments, you know that acetic acid has a large peak at 1710 cm^-1^ and is the major metabolic product. You expect the concentration of acetic acid in water to linearly correlate to the height of the peak at 1710 cm^-1^, based on literature detailing aqueous IR spectra for different carboxylic acids^1^. Before setting up a growth study, you make a standard curve for acetic acid by preparing a dilution of acetic acid and measuring the peak heights. You then repeat the same procedure for acetic acid in your growth medium.


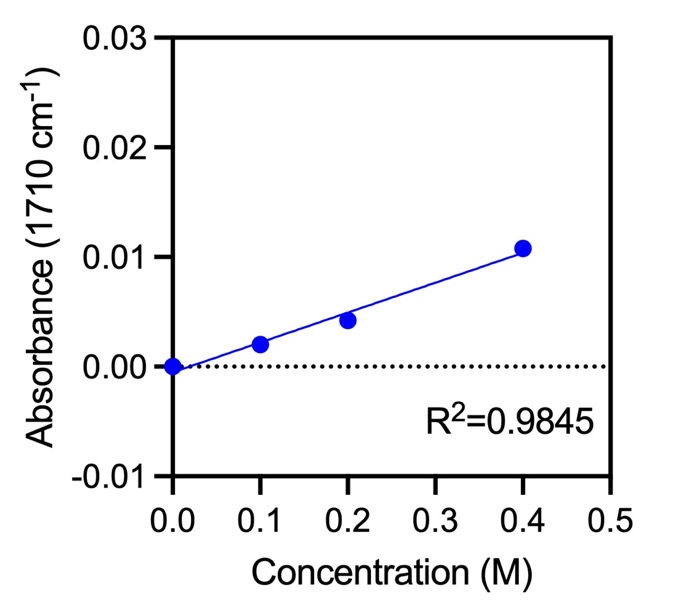

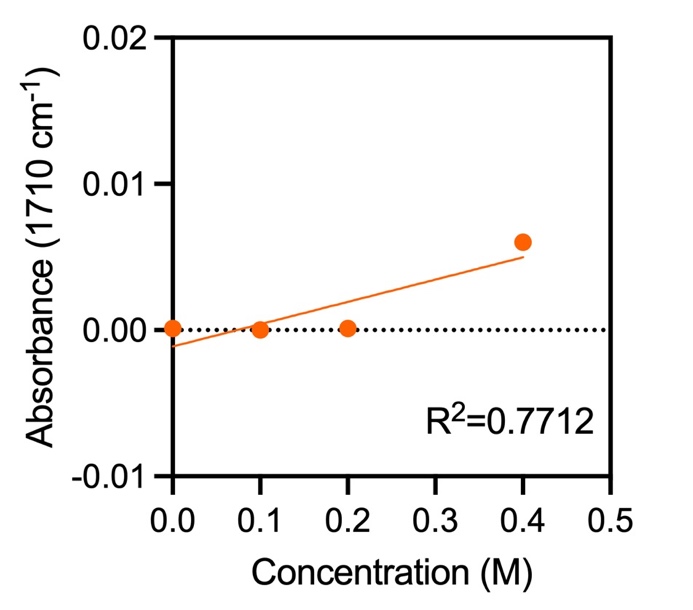


**Figure 2.** Peak height for different concentrations of aqueous acetic acid (left) and acetic acid in growth medium (right).

You see that at low concentrations, there is poor agreement between the linear fit in water versus your growth media. In the growth media, near the lower end of concentrations, there is little-to-no measurable absorbance. However, this was not the case when measured in water.

**Detailed procedure and workflow:**

**FTIR:**

1. The FTIR-ATR crystal is cleaned with DI water and wiped dry
2. The collection range is set to 1800-1000 cm^-1^, with 2 cm^-1^ resolution and 48 scans
3. 20 µL of DI water is loaded onto the ATR crystal, which is then covered with a plastic cap
4. The absorbance is measured
5. The ATR crystal is wiped dry, and 20 µL of sample is added to the crystal
6. The background spectrum is collected under the same conditions
7. The peak height is measured at 1710 cm^-1^

**Sample preparation:**

1. Acetic acid is prepared at the following concentrations in DI water: 0, 0.1, 0.2, 0.4, 0.8, and 1.6 M
2. 10X Growth medium is prepared by mixing 10.7 g Na_2_HPO_4_ and 2.95 g NaH_2_PO_4_, which is then diluted
3. Acetic acid is prepared at the same concentrations as prior, in 1X growth medium


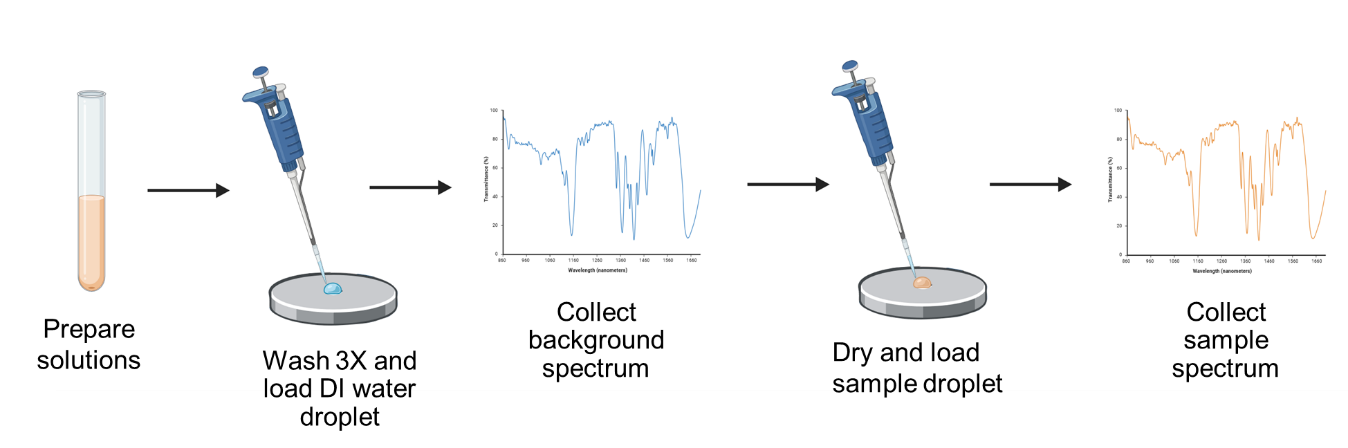


**Figure 3.** Experimental workflow.

**Source of error:**

The acetic acid peak at 1710 cm^-1^ was extinguished when acetic acid was deprotonated to acetate. Because IR spectroscopy depends on the vibration of molecular bonds, losing or gaining bonds will significantly impact the spectra of compounds in solution.

**Table 1.** Additional information known by the leader that can be provided upon request

| **Meeting Notes for the Leader**  Not to be shared with the group | |
| --- | --- |
| Other researcher’s experiments | - The FTIR instrument (Nicolet iS10) is rarely used by others. Others do regularly use the pH meter, likely to check the pH of culture media. - The acids and salts are shared with others in the lab. |
| Storage information | - The acetic acid solutions are prepared by diluting glacial acetic acid. - Sodium phosphate and sodium acetate salts are stored in a chemical cabinet, in open air. - Once the solutions are prepared, they are stored in closed polypropylene tubes on the bench. |
| Device information | - The pH meter has been calibrated within the last month, and the FTIR spectrometer seems to be functioning without error. |
| Source of error | - The acetic acid peak at 1710 cm^-1^ was extinguished when acetic acid was deprotonated to acetate. |
| Hints for group | - Replicates of these experiments did not significantly affect the result. - The fit seems to fail for concentrations up to the concentration of buffer in the growth medium, but is ok at higher concentrations. |

**References:**

1. Max, Jean-Joseph, and Camille Chapados. "Infrared spectroscopy of aqueous carboxylic acids: comparison between different acids and their salts." The Journal of Physical Chemistry A 108.16 (2004): 3324-3337.
2. Semeraro, Paola, et al. "A simple strategy based on ATR-FTIR difference spectroscopy to monitor substrate intake and metabolite release by growing bacteria." *Spectrochimica Acta Part A: Molecular and Biomolecular Spectroscopy* 302 (2023): 123031.
3. Mackie, David M., et al. "Simple, fast, and accurate methodology for quantitative analysis using Fourier transform infrared spectroscopy, with bio-hybrid fuel cell examples." *MethodsX* 3 (2016): 128-138.
