# Supplementary material for: Teaching troubleshooting skills to graduate students
Source: eLife. 2024 Sep 17;13:e100761. doi: 10.7554/eLife.100761 (PMC11407763; doi:10.7554/eLife.100761)
Supplement: Supplementary file 1. — For each scenario there is a Word file that contains the following: background information; a description of the scenario; the protocol for the experiment that produced the unexpected result; the results of the experiment; information on the source of the error; background information that can be used to answer questions; and references. There is also a PowerPoint file for each scenario that contains example slides that can be used in real meetings. There are also templates for the Word and PowerPoint files. [file elife-100761-supp1.zip › Final Scenarios/Example2.pptx]

## Slide 1
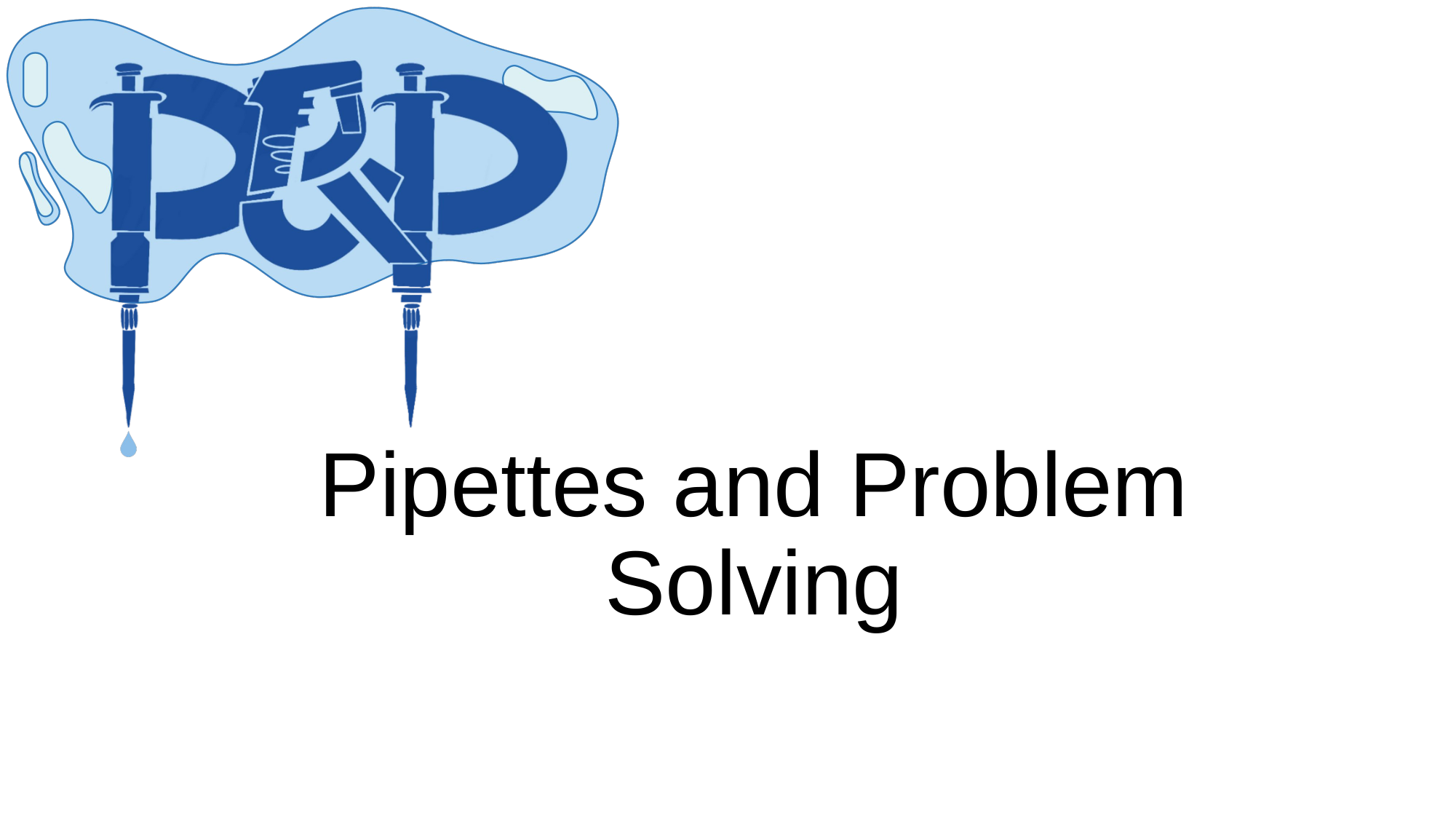

# Pipettes and Problem Solving

## Slide 2
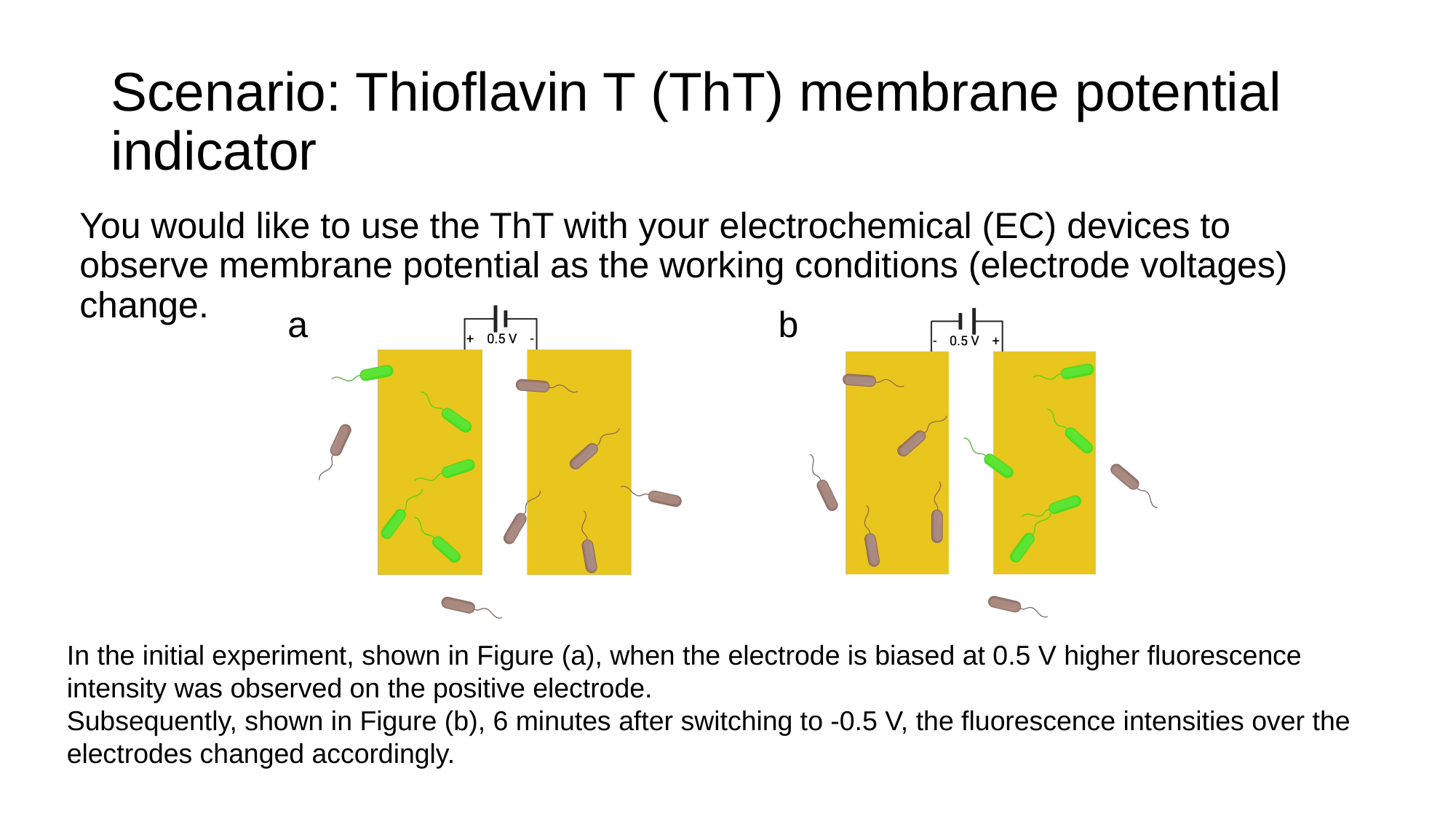

# Scenario: Thioflavin T (ThT) membrane potential indicator
You would like to use the ThT with your electrochemical (EC) devices to observe membrane potential as the working conditions (electrode voltages) change.
a
b
In the initial experiment, shown in Figure (a), when the electrode is biased at 0.5 V higher fluorescence intensity was observed on the positive electrode.
Subsequently, shown in Figure (b), 6 minutes after switching to -0.5 V, the fluorescence intensities over the electrodes changed accordingly.

## Slide 3
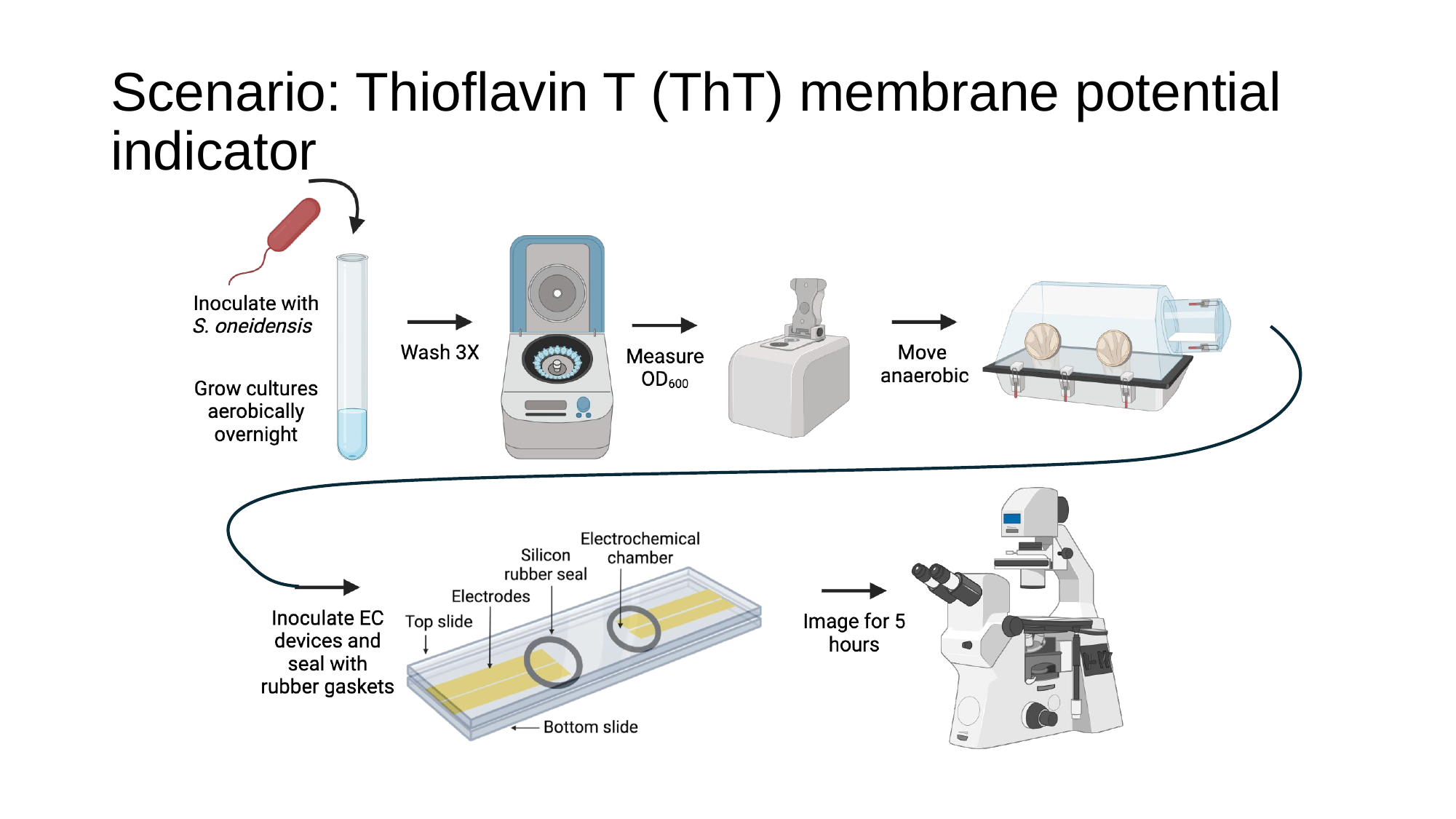

# Scenario: Thioflavin T (ThT) membrane potential indicator

## Slide 4
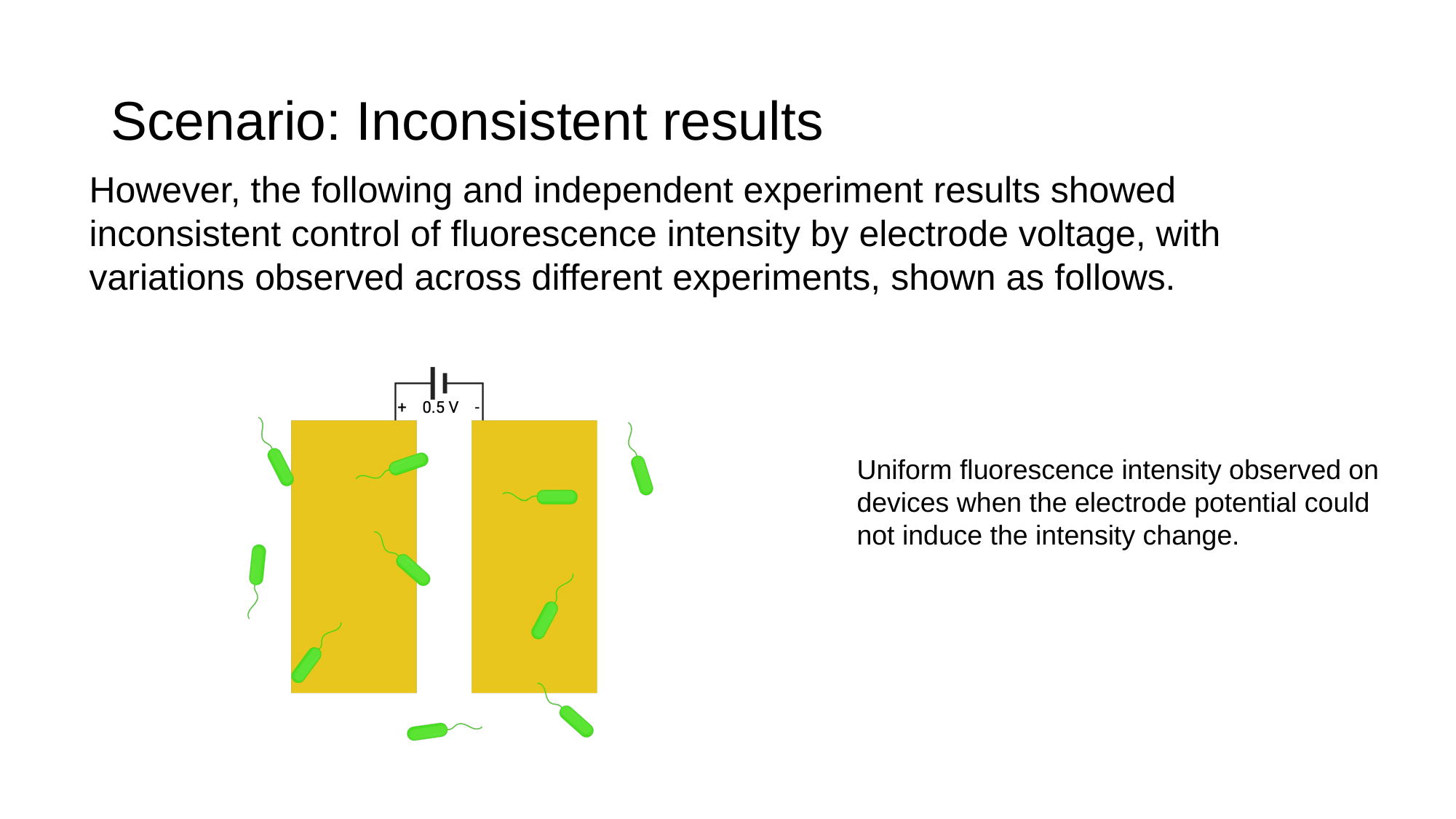

# Scenario: Inconsistent results
However, the following and independent experiment results showed inconsistent control of fluorescence intensity by electrode voltage, with variations observed across different experiments, shown as follows.
Uniform fluorescence intensity observed on devices when the electrode potential could not induce the intensity change.

## Slide 5
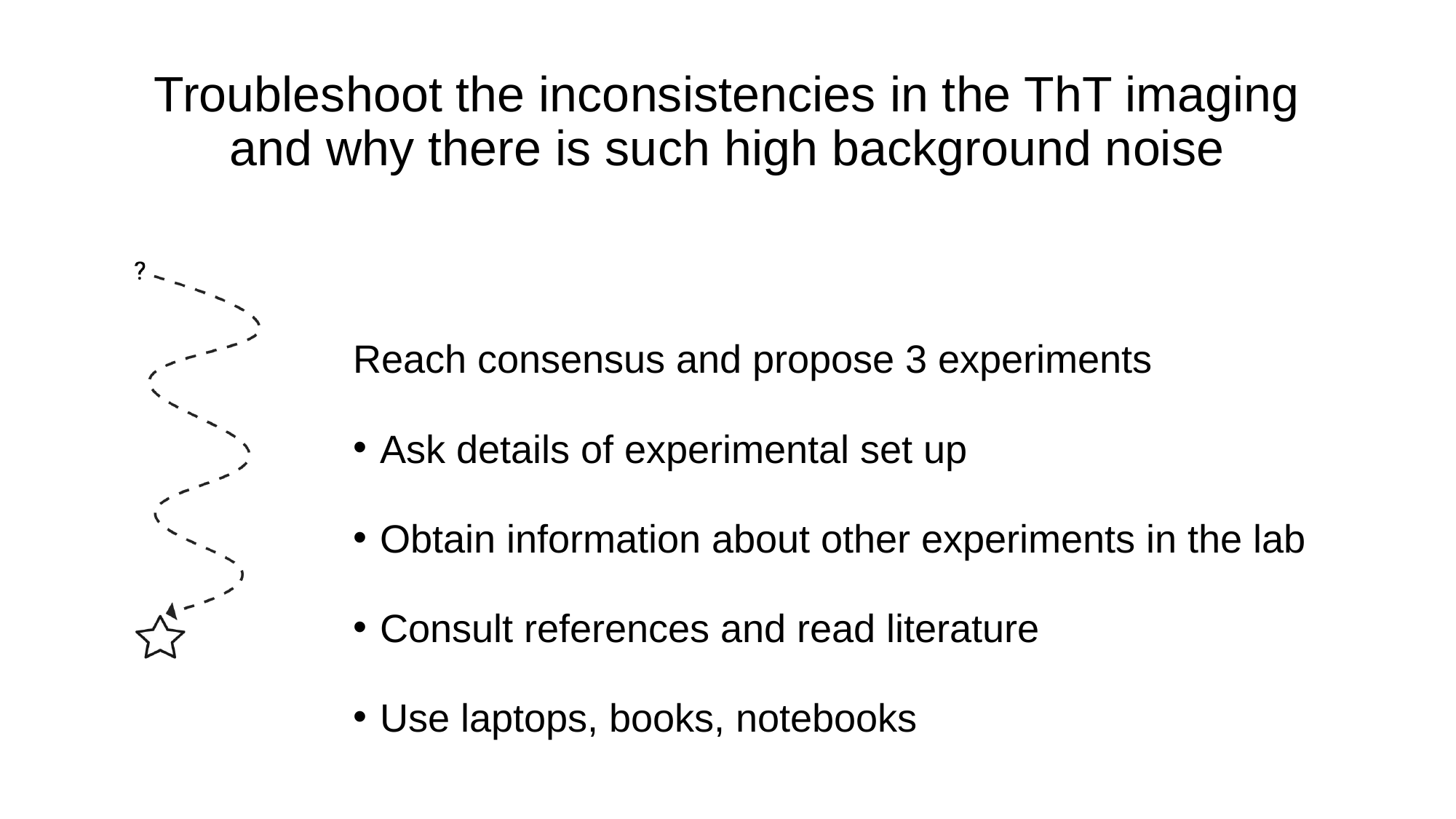

# Troubleshoot the inconsistencies in the ThT imaging and why there is such high background noise
Reach consensus and propose 3 experiments
Ask details of experimental set up
Obtain information about other experiments in the lab
Consult references and read literature
Use laptops, books, notebooks

## Slide 6
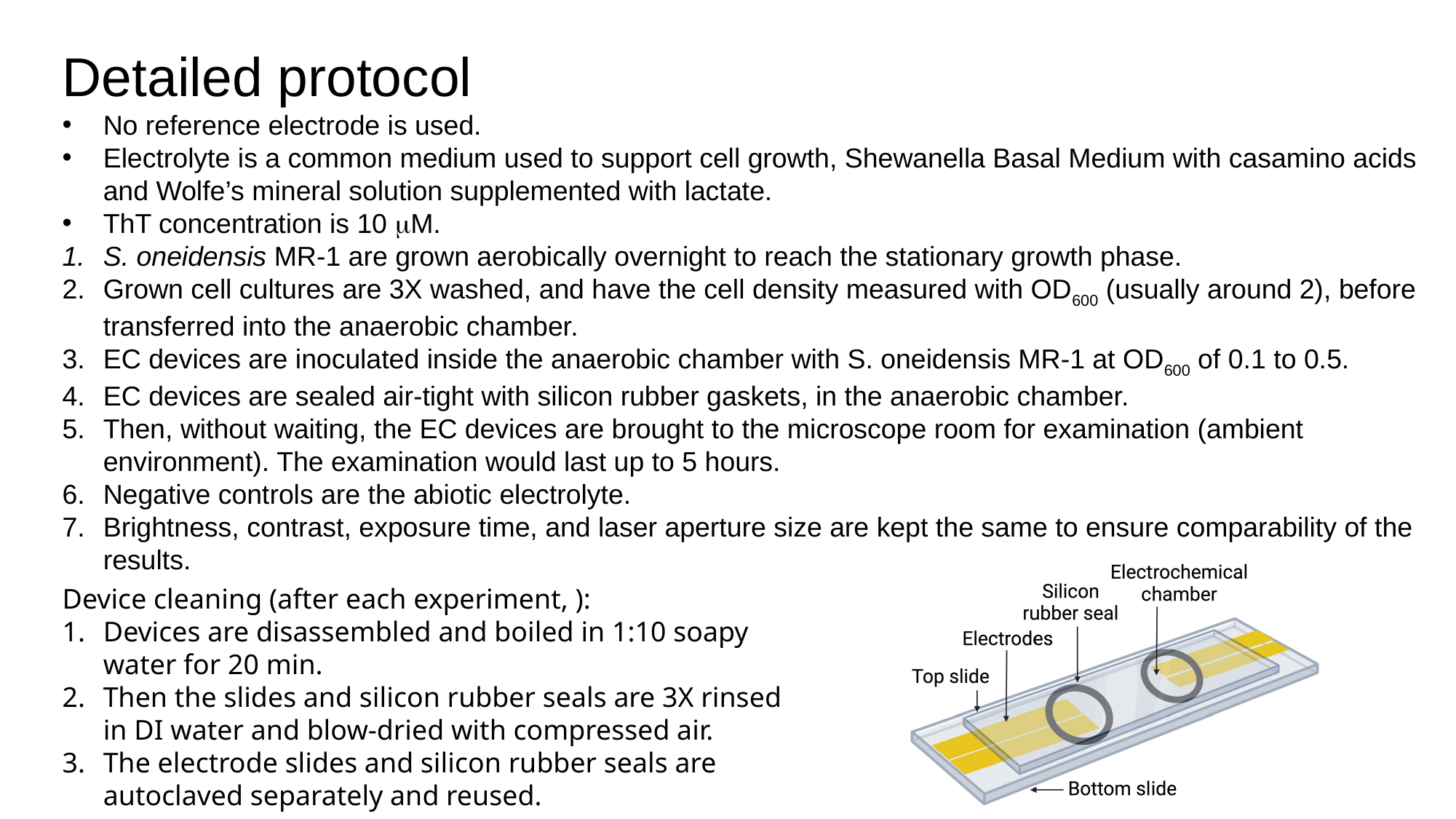

Detailed protocol
No reference electrode is used.
Electrolyte is a common medium used to support cell growth, Shewanella Basal Medium with casamino acids and Wolfe’s mineral solution supplemented with lactate.
ThT concentration is 10 μM.
S. oneidensis MR-1 are grown aerobically overnight to reach the stationary growth phase.
Grown cell cultures are 3X washed, and have the cell density measured with OD600 (usually around 2), before transferred into the anaerobic chamber.
EC devices are inoculated inside the anaerobic chamber with S. oneidensis MR-1 at OD600 of 0.1 to 0.5.
EC devices are sealed air-tight with silicon rubber gaskets, in the anaerobic chamber.
Then, without waiting, the EC devices are brought to the microscope room for examination (ambient environment). The examination would last up to 5 hours.
Negative controls are the abiotic electrolyte.
Brightness, contrast, exposure time, and laser aperture size are kept the same to ensure comparability of the results.
Device cleaning (after each experiment, ):
Devices are disassembled and boiled in 1:10 soapy water for 20 min.
Then the slides and silicon rubber seals are 3X rinsed in DI water and blow-dried with compressed air.
The electrode slides and silicon rubber seals are autoclaved separately and reused.

## Slide 7
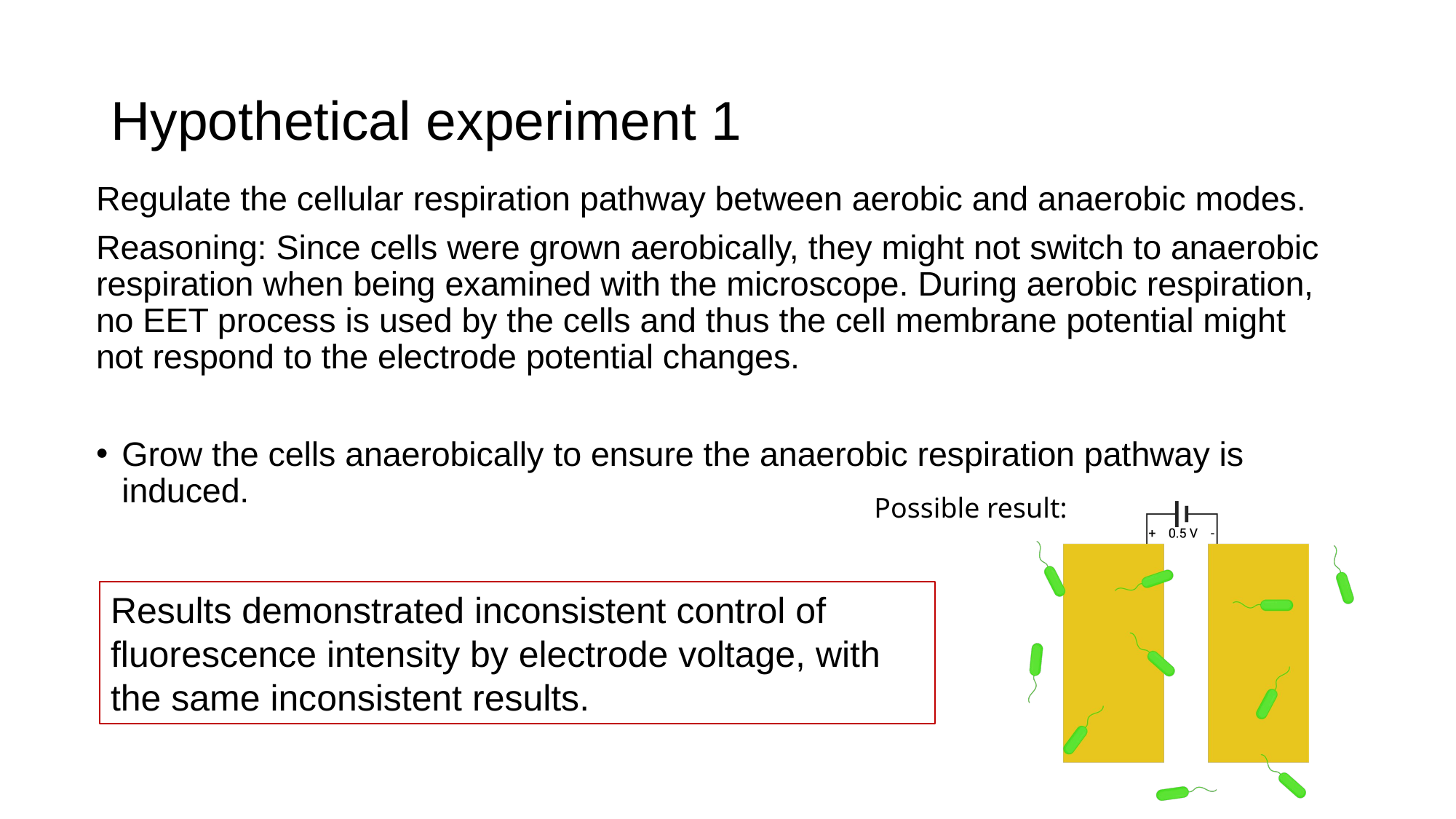

# Hypothetical experiment 1
Regulate the cellular respiration pathway between aerobic and anaerobic modes.
Reasoning: Since cells were grown aerobically, they might not switch to anaerobic respiration when being examined with the microscope. During aerobic respiration, no EET process is used by the cells and thus the cell membrane potential might not respond to the electrode potential changes.
Grow the cells anaerobically to ensure the anaerobic respiration pathway is induced.
Possible result:
Results demonstrated inconsistent control of fluorescence intensity by electrode voltage, with the same inconsistent results.

## Slide 8
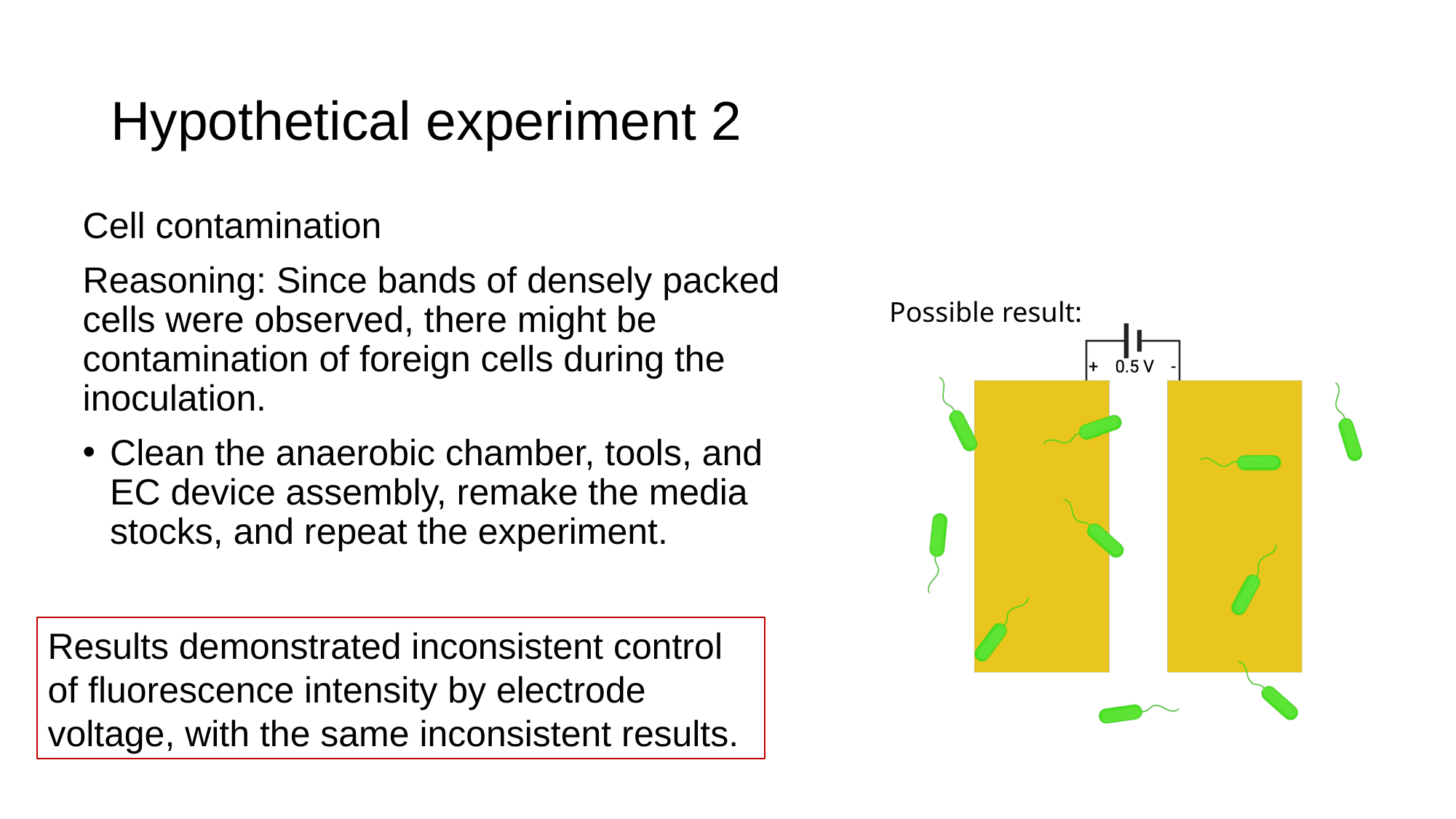

# Hypothetical experiment 2
Cell contamination
Reasoning: Since bands of densely packed cells were observed, there might be contamination of foreign cells during the inoculation.
Clean the anaerobic chamber, tools, and EC device assembly, remake the media stocks, and repeat the experiment.
Possible result:
Results demonstrated inconsistent control of fluorescence intensity by electrode voltage, with the same inconsistent results.

## Slide 9
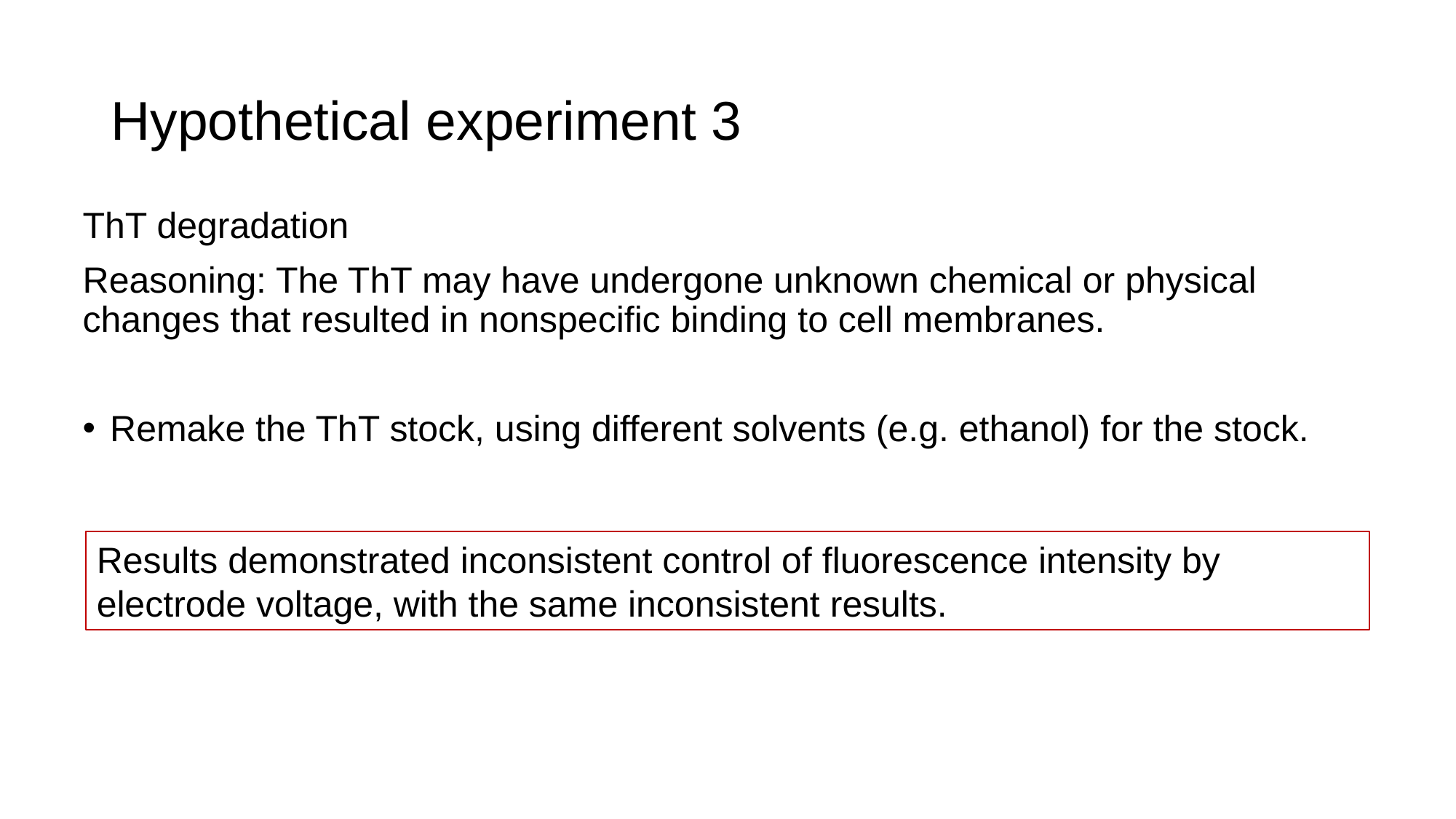

# Hypothetical experiment 3
ThT degradation
Reasoning: The ThT may have undergone unknown chemical or physical changes that resulted in nonspecific binding to cell membranes.
Remake the ThT stock, using different solvents (e.g. ethanol) for the stock.
Results demonstrated inconsistent control of fluorescence intensity by electrode voltage, with the same inconsistent results.

## Slide 10
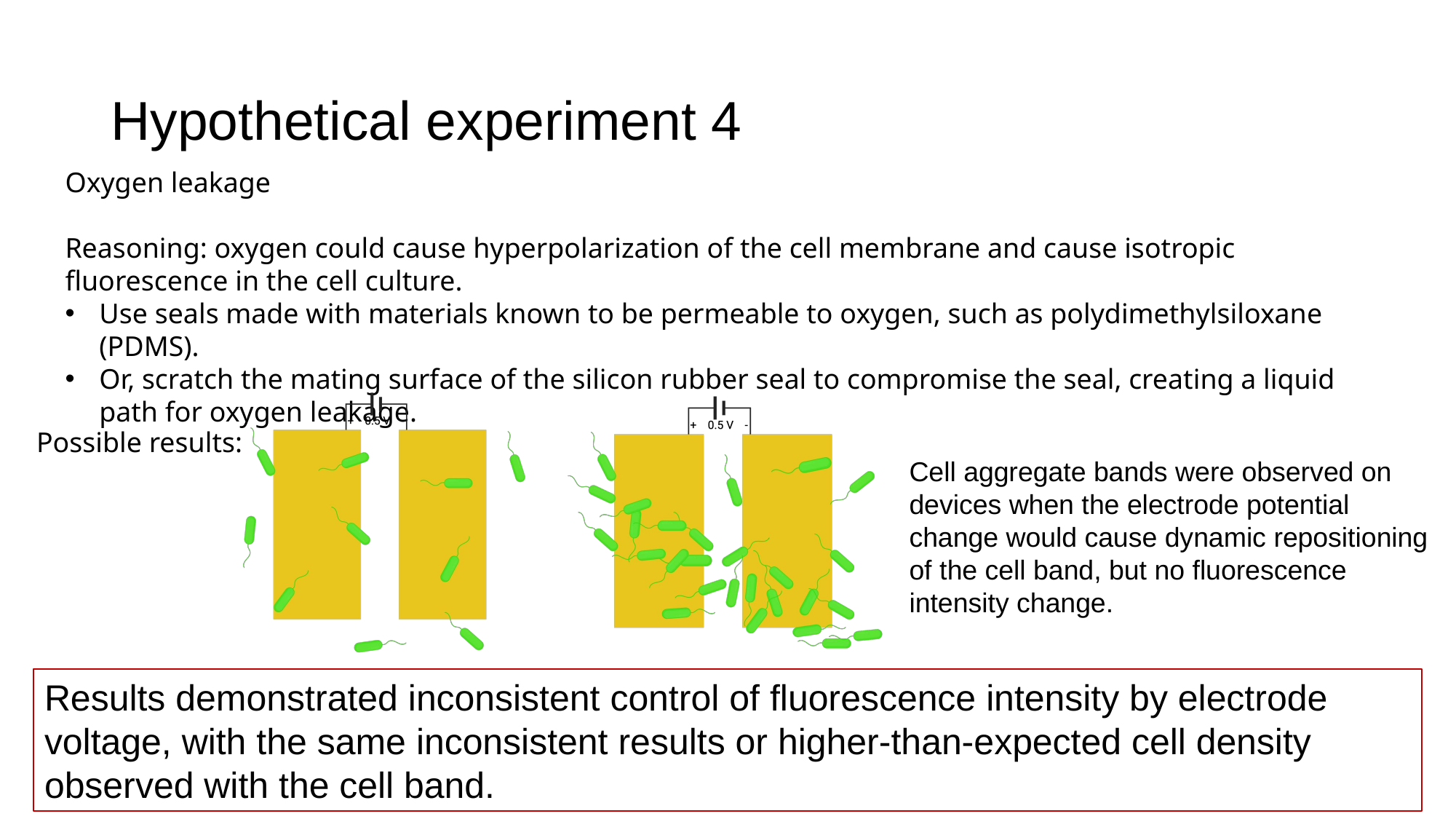

# Hypothetical experiment 4
Oxygen leakage
Reasoning: oxygen could cause hyperpolarization of the cell membrane and cause isotropic fluorescence in the cell culture.
Use seals made with materials known to be permeable to oxygen, such as polydimethylsiloxane (PDMS).
Or, scratch the mating surface of the silicon rubber seal to compromise the seal, creating a liquid path for oxygen leakage.
Possible results:
Cell aggregate bands were observed on devices when the electrode potential change would cause dynamic repositioning of the cell band, but no fluorescence intensity change.
Results demonstrated inconsistent control of fluorescence intensity by electrode voltage, with the same inconsistent results or higher-than-expected cell density observed with the cell band.

## Slide 11
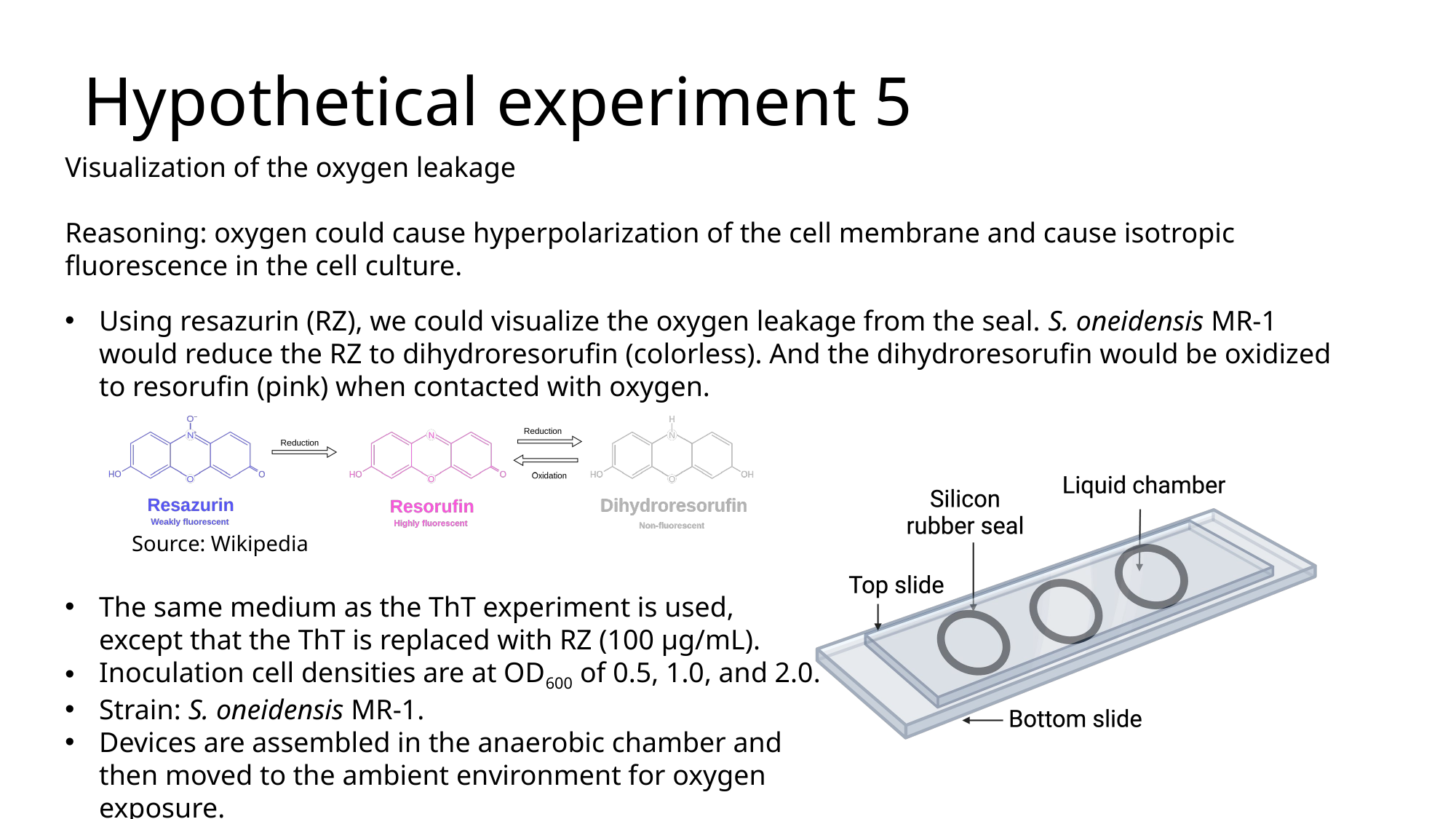

Hypothetical experiment 5
Visualization of the oxygen leakage
Reasoning: oxygen could cause hyperpolarization of the cell membrane and cause isotropic fluorescence in the cell culture.
Using resazurin (RZ), we could visualize the oxygen leakage from the seal. S. oneidensis MR-1 would reduce the RZ to dihydroresorufin (colorless). And the dihydroresorufin would be oxidized to resorufin (pink) when contacted with oxygen.
Source: Wikipedia
The same medium as the ThT experiment is used, except that the ThT is replaced with RZ (100 μg/mL).
Inoculation cell densities are at OD600 of 0.5, 1.0, and 2.0.
Strain: S. oneidensis MR-1.
Devices are assembled in the anaerobic chamber and then moved to the ambient environment for oxygen exposure.

## Slide 12
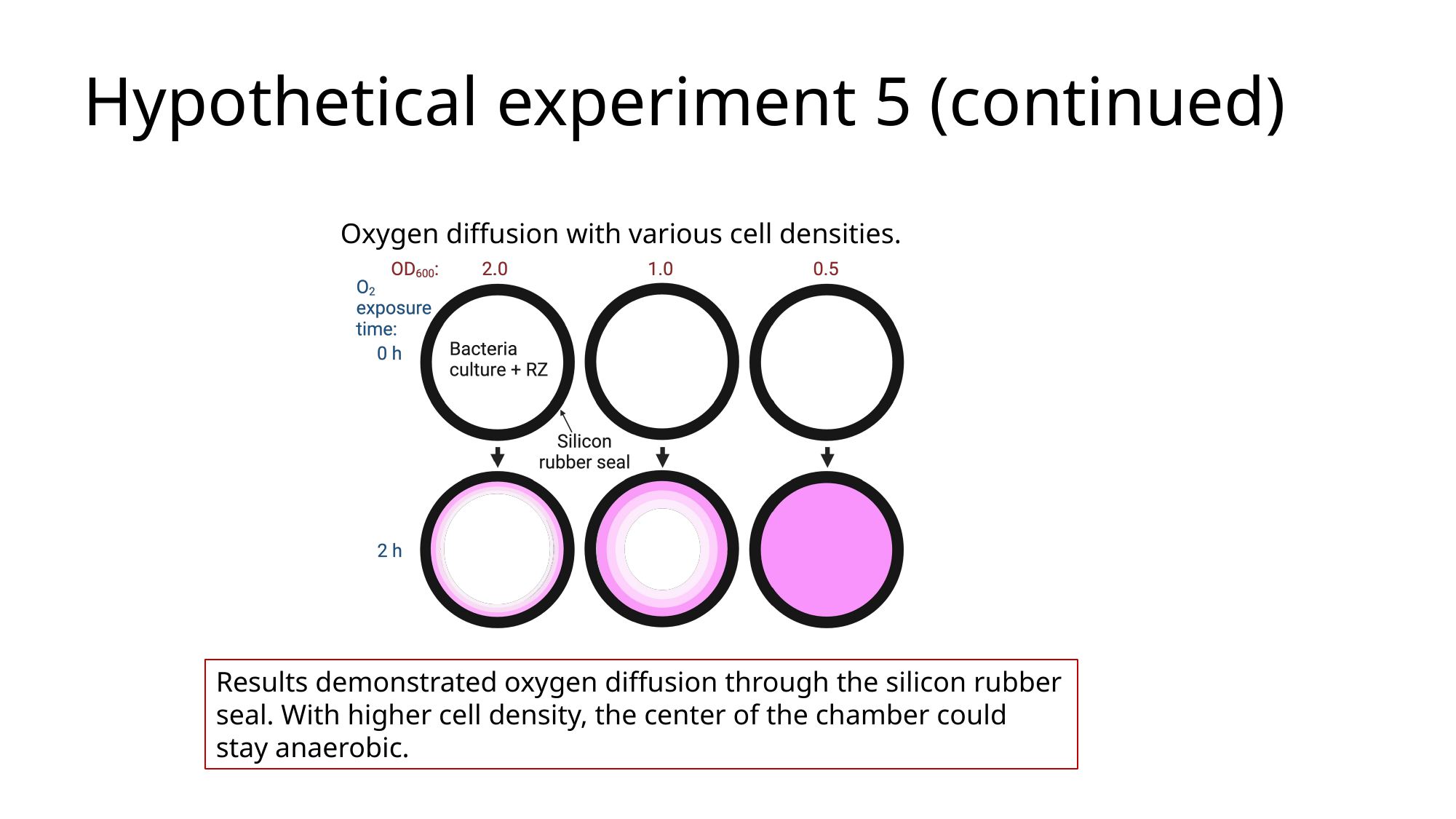

Hypothetical experiment 5 (continued)
Oxygen diffusion with various cell densities.
Results demonstrated oxygen diffusion through the silicon rubber seal. With higher cell density, the center of the chamber could stay anaerobic.

## Slide 13
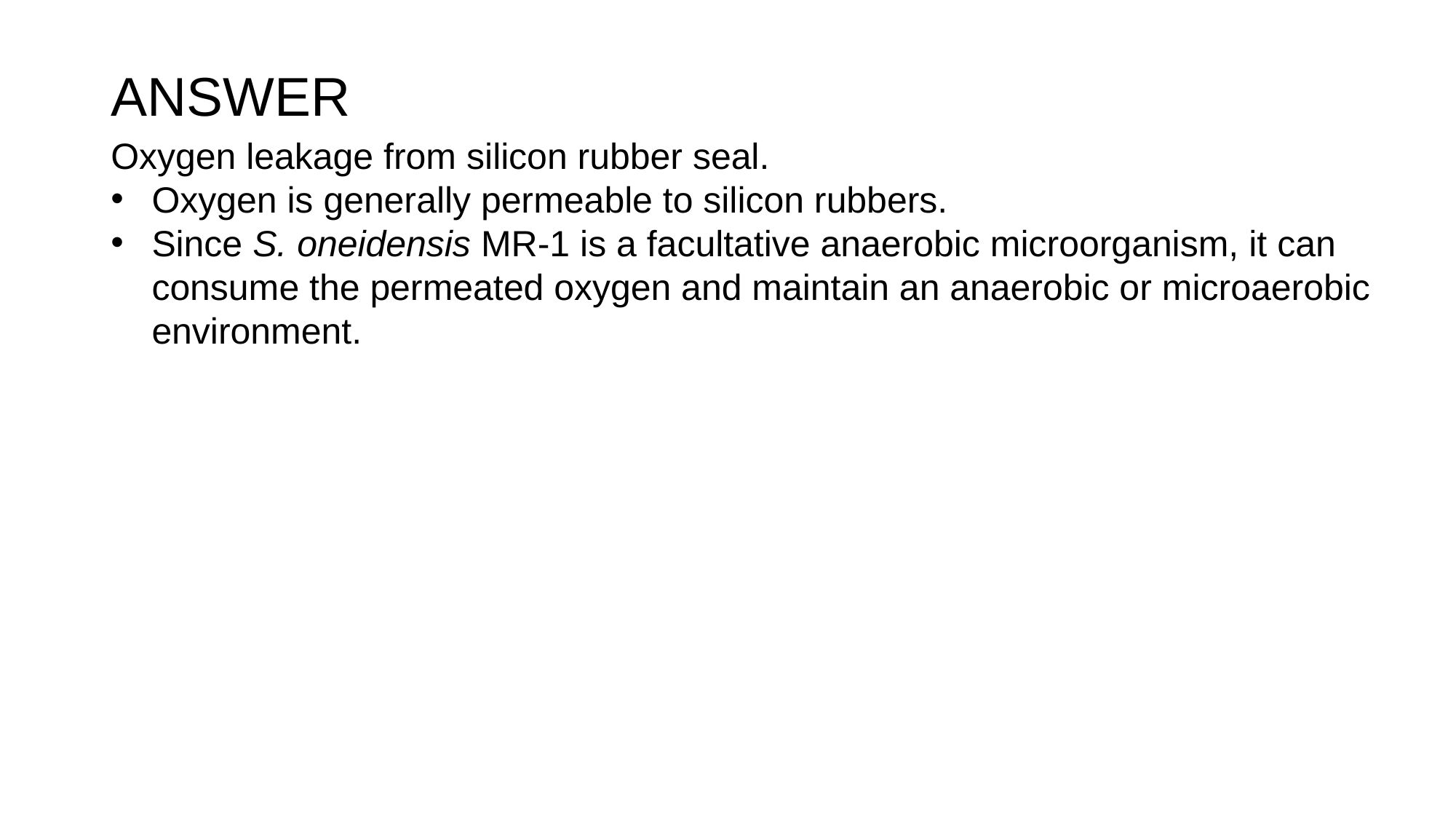

# ANSWER
Oxygen leakage from silicon rubber seal.
Oxygen is generally permeable to silicon rubbers.
Since S. oneidensis MR-1 is a facultative anaerobic microorganism, it can consume the permeated oxygen and maintain an anaerobic or microaerobic environment.
