# Supplementary material for: Teaching troubleshooting skills to graduate students
Source: eLife. 2024 Sep 17;13:e100761. doi: 10.7554/eLife.100761 (PMC11407763; doi:10.7554/eLife.100761)
Supplement: Supplementary file 1. — For each scenario there is a Word file that contains the following: background information; a description of the scenario; the protocol for the experiment that produced the unexpected result; the results of the experiment; information on the source of the error; background information that can be used to answer questions; and references. There is also a PowerPoint file for each scenario that contains example slides that can be used in real meetings. There are also templates for the Word and PowerPoint files. [file elife-100761-supp1.zip › Final Scenarios/Example3.pptx]

## Slide 1
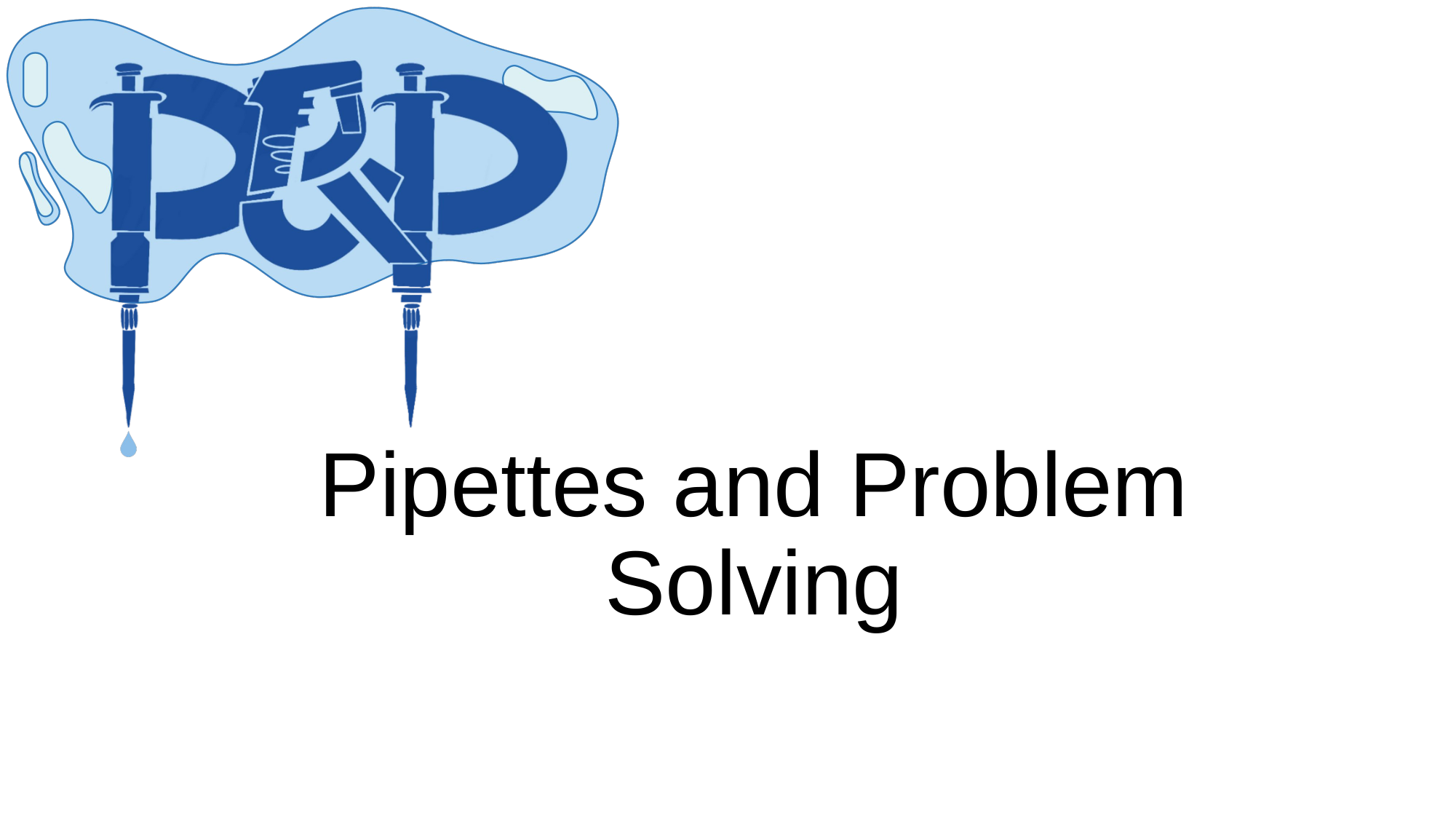

# Pipettes and Problem Solving

## Slide 2
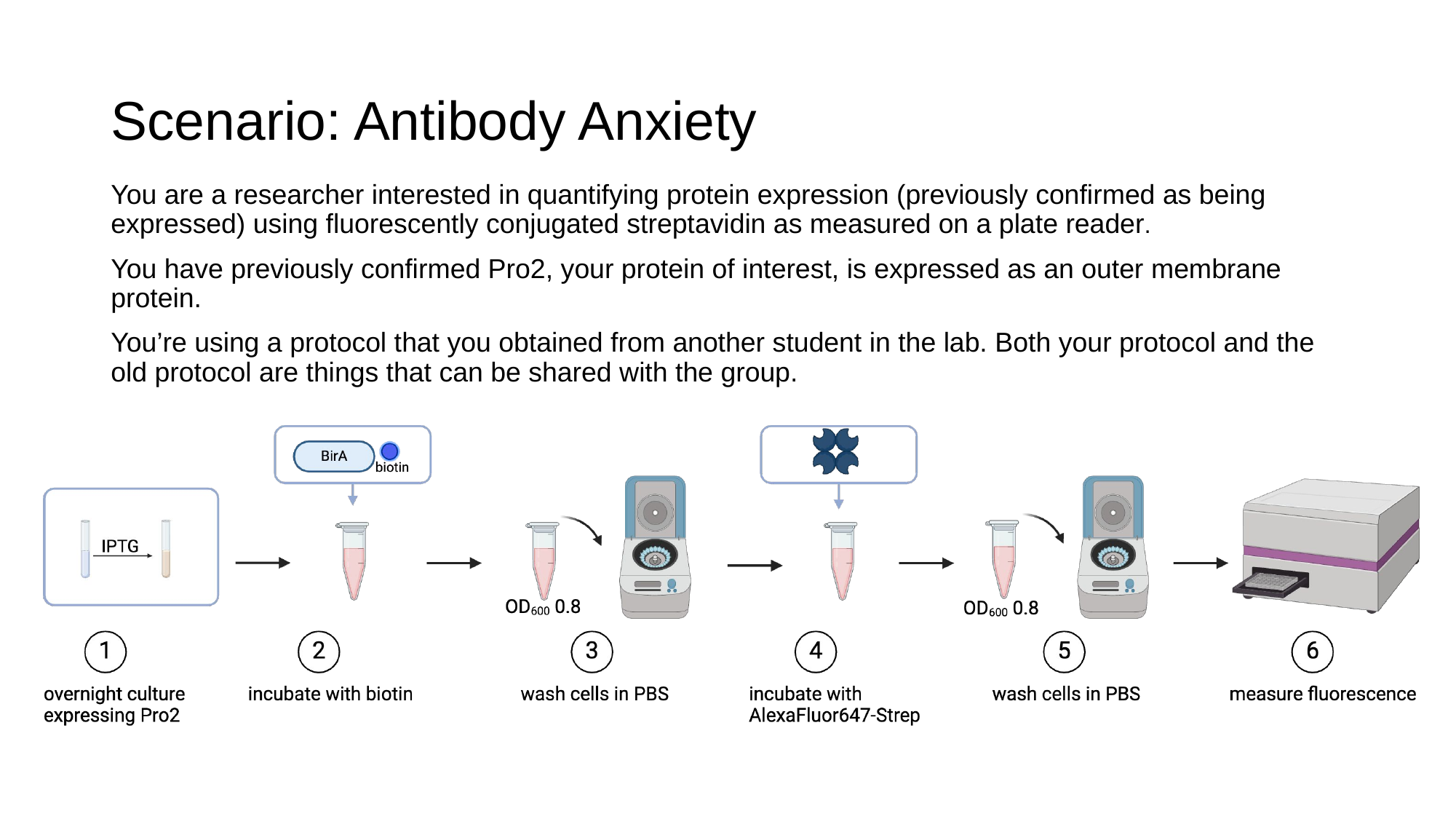

# Scenario: Antibody Anxiety
You are a researcher interested in quantifying protein expression (previously confirmed as being expressed) using fluorescently conjugated streptavidin as measured on a plate reader.
You have previously confirmed Pro2, your protein of interest, is expressed as an outer membrane protein.
You’re using a protocol that you obtained from another student in the lab. Both your protocol and the old protocol are things that can be shared with the group.

## Slide 3
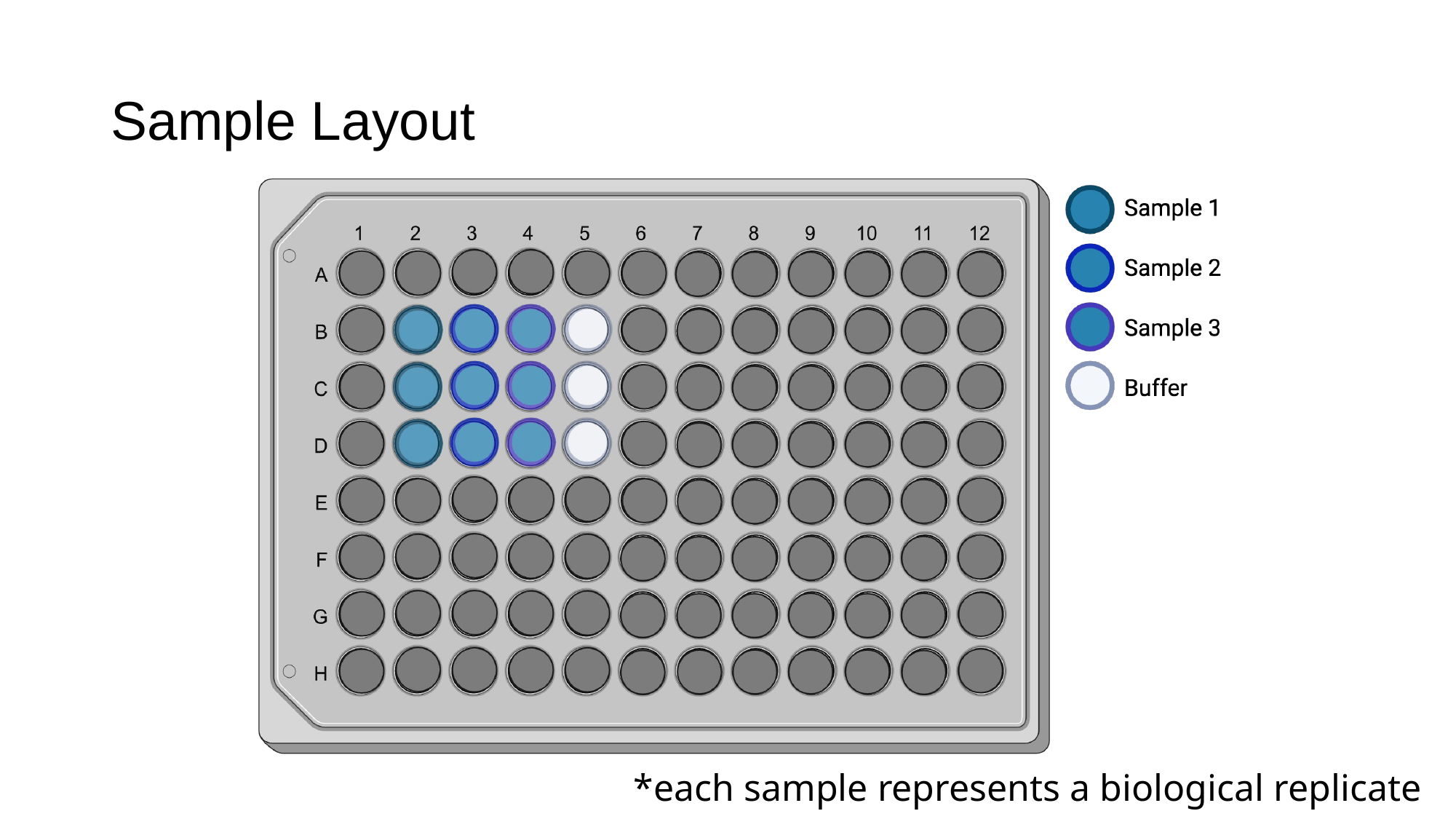

# Sample Layout
1.
2.
3.
4.
4.
3.
1.
2.
1.
2.
3.
4.
*each sample represents a biological replicate

## Slide 4
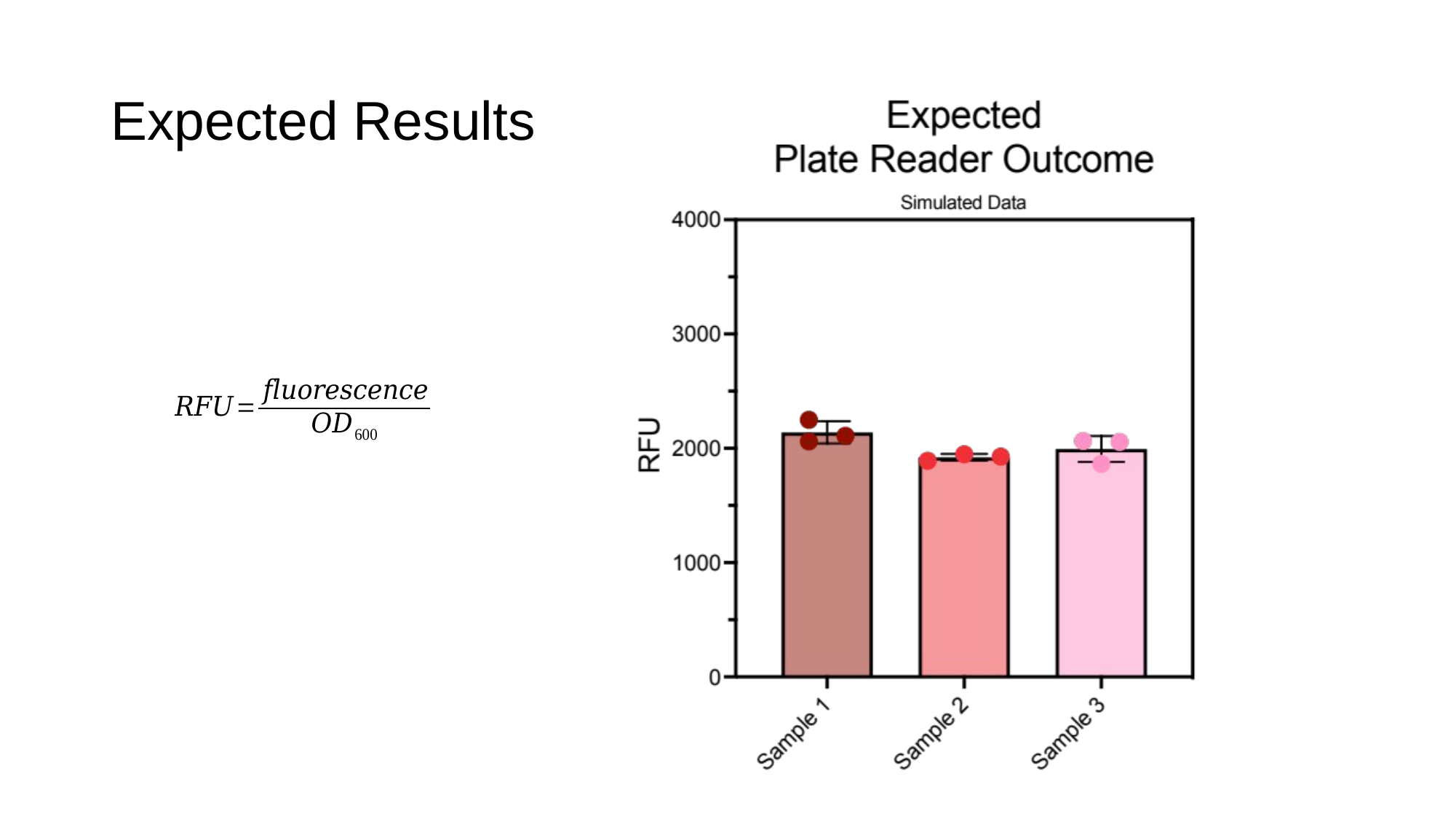

# Expected Results

## Slide 5
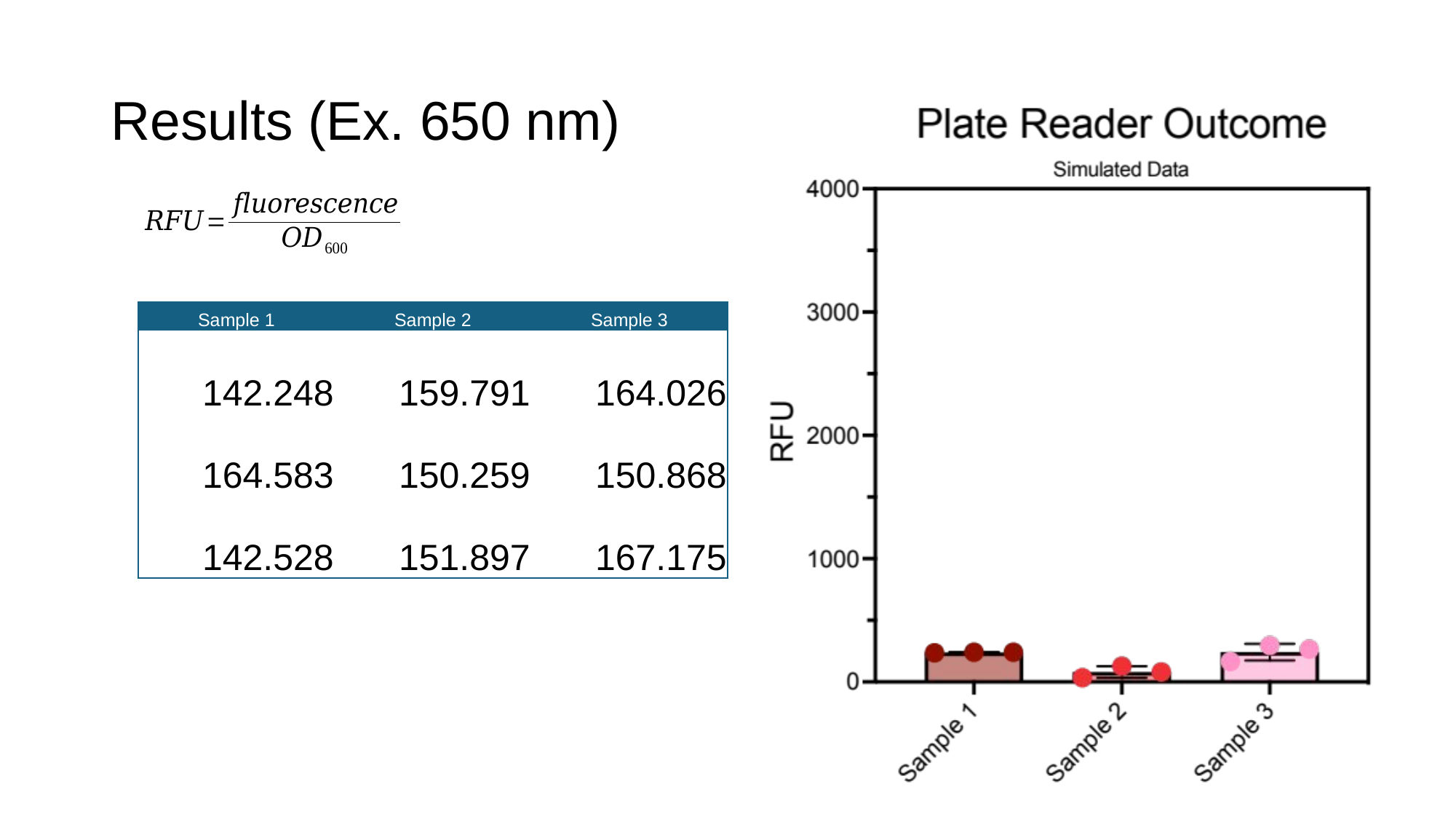

# Results (Ex. 650 nm)
| Sample 1 | Sample 2 | Sample 3 |
| --- | --- | --- |
| 142.248 | 159.791 | 164.026 |
| 164.583 | 150.259 | 150.868 |
| 142.528 | 151.897 | 167.175 |

## Slide 6
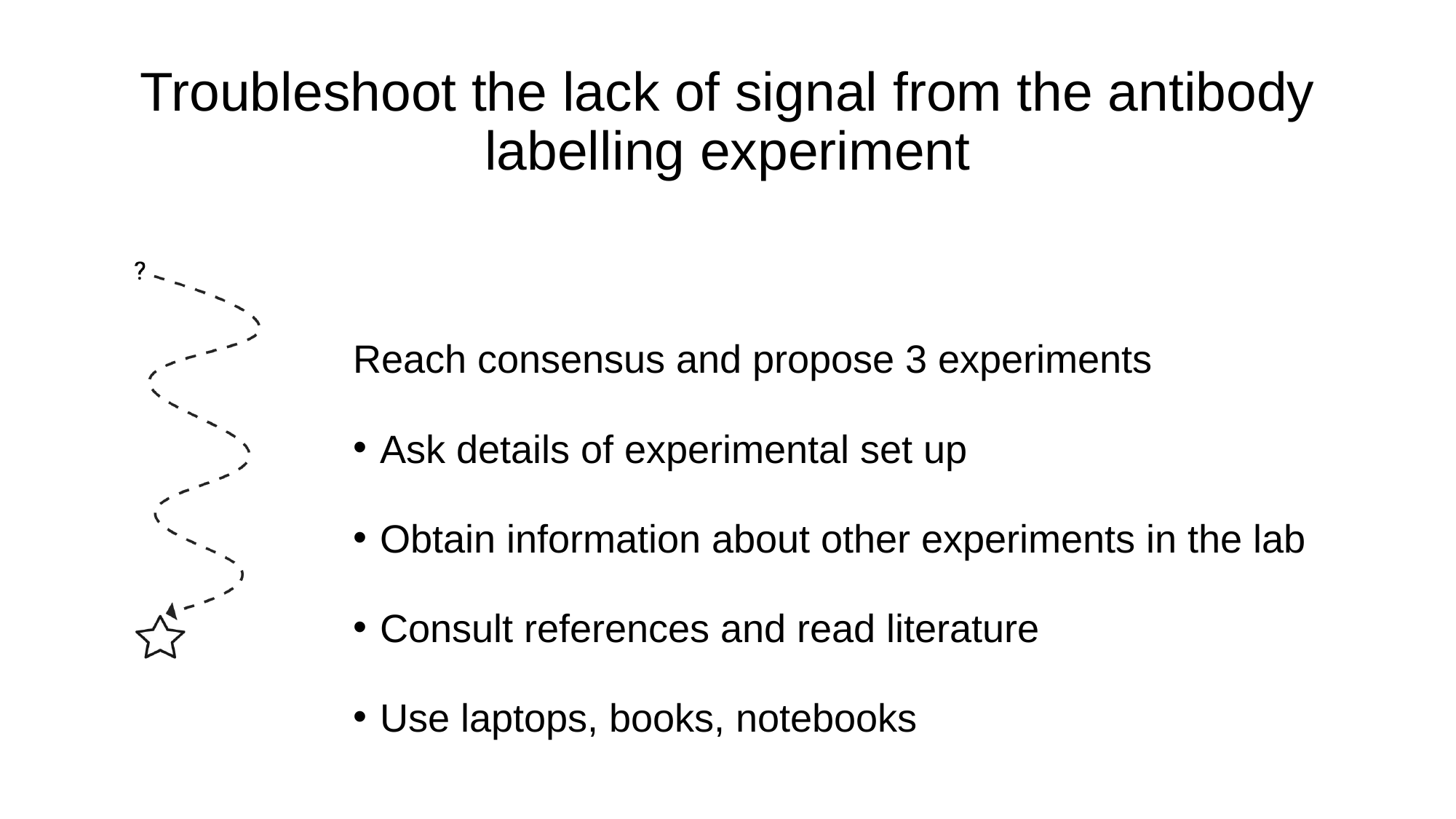

# Troubleshoot the lack of signal from the antibody labelling experiment
Reach consensus and propose 3 experiments
Ask details of experimental set up
Obtain information about other experiments in the lab
Consult references and read literature
Use laptops, books, notebooks

## Slide 7
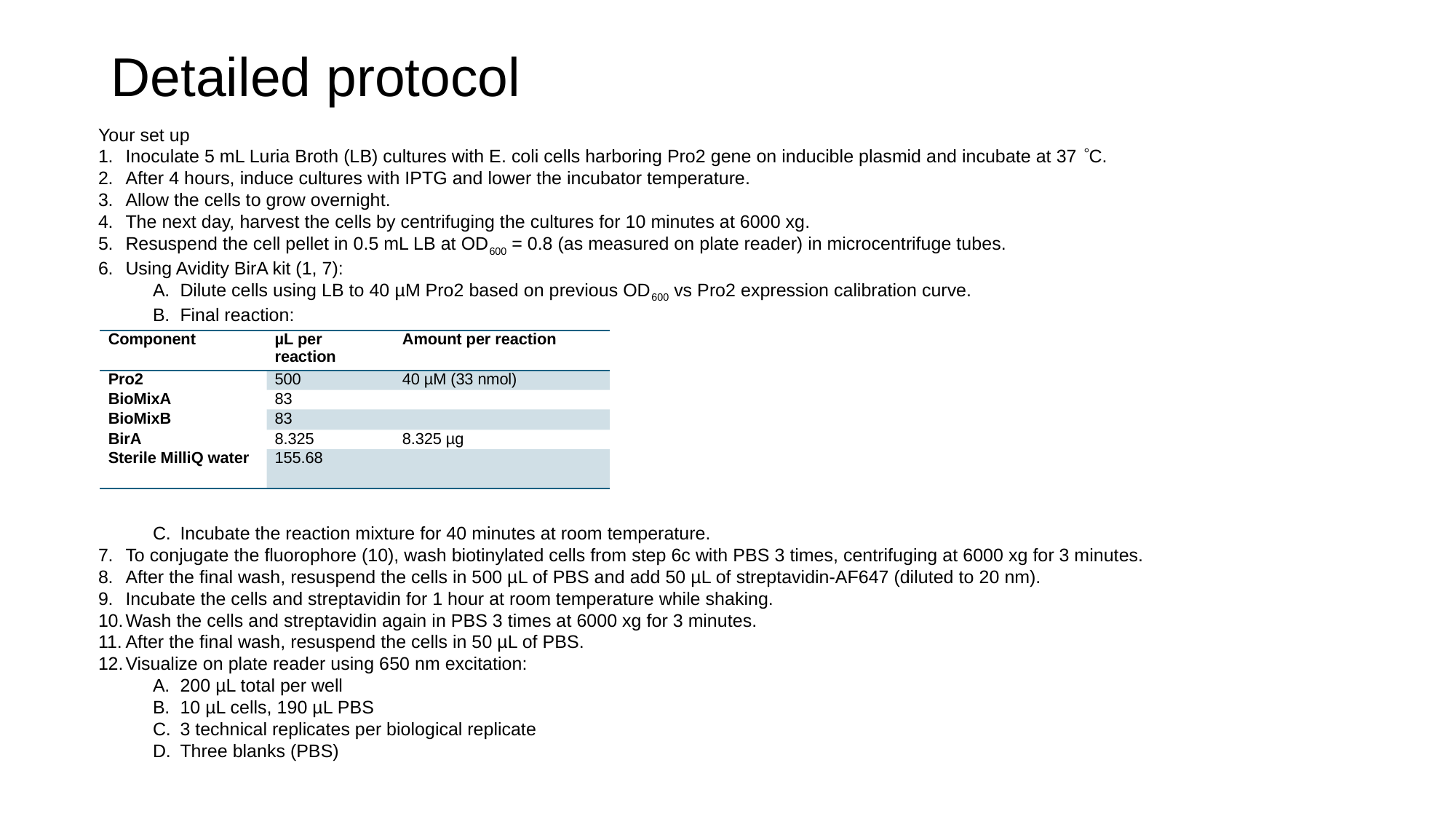

# Detailed protocol
Your set up
Inoculate 5 mL Luria Broth (LB) cultures with E. coli cells harboring Pro2 gene on inducible plasmid and incubate at 37 °C.
After 4 hours, induce cultures with IPTG and lower the incubator temperature.
Allow the cells to grow overnight.
The next day, harvest the cells by centrifuging the cultures for 10 minutes at 6000 xg.
Resuspend the cell pellet in 0.5 mL LB at OD600 = 0.8 (as measured on plate reader) in microcentrifuge tubes.
Using Avidity BirA kit (1, 7):
Dilute cells using LB to 40 µM Pro2 based on previous OD600 vs Pro2 expression calibration curve.
Final reaction:
Incubate the reaction mixture for 40 minutes at room temperature.
To conjugate the fluorophore (10), wash biotinylated cells from step 6c with PBS 3 times, centrifuging at 6000 xg for 3 minutes.
After the final wash, resuspend the cells in 500 µL of PBS and add 50 µL of streptavidin-AF647 (diluted to 20 nm).
Incubate the cells and streptavidin for 1 hour at room temperature while shaking.
Wash the cells and streptavidin again in PBS 3 times at 6000 xg for 3 minutes.
After the final wash, resuspend the cells in 50 µL of PBS.
Visualize on plate reader using 650 nm excitation:
200 µL total per well
10 µL cells, 190 µL PBS
3 technical replicates per biological replicate
Three blanks (PBS)
| Component | µL per reaction | Amount per reaction |
| --- | --- | --- |
| Pro2 | 500 | 40 µM (33 nmol) |
| BioMixA | 83 | |
| BioMixB | 83 | |
| BirA | 8.325 | 8.325 µg |
| Sterile MilliQ water | 155.68 | |

## Slide 8
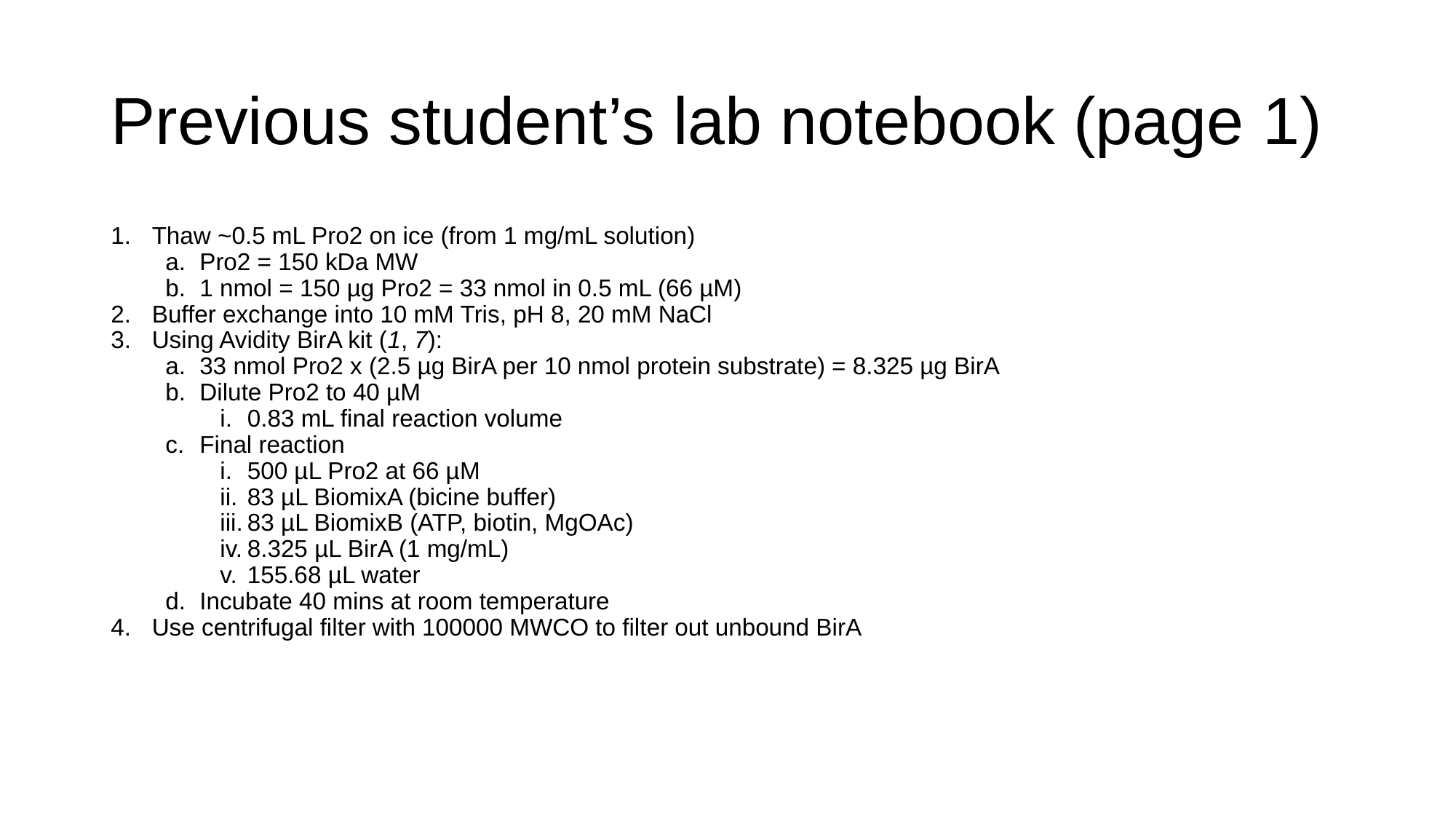

# Previous student’s lab notebook (page 1)
Thaw ~0.5 mL Pro2 on ice (from 1 mg/mL solution)
Pro2 = 150 kDa MW
1 nmol = 150 µg Pro2 = 33 nmol in 0.5 mL (66 µM)
Buffer exchange into 10 mM Tris, pH 8, 20 mM NaCl
Using Avidity BirA kit (1, 7):
33 nmol Pro2 x (2.5 µg BirA per 10 nmol protein substrate) = 8.325 µg BirA
Dilute Pro2 to 40 µM
0.83 mL final reaction volume
Final reaction
500 µL Pro2 at 66 µM
83 µL BiomixA (bicine buffer)
83 µL BiomixB (ATP, biotin, MgOAc)
8.325 µL BirA (1 mg/mL)
155.68 µL water
Incubate 40 mins at room temperature
Use centrifugal filter with 100000 MWCO to filter out unbound BirA

## Slide 9
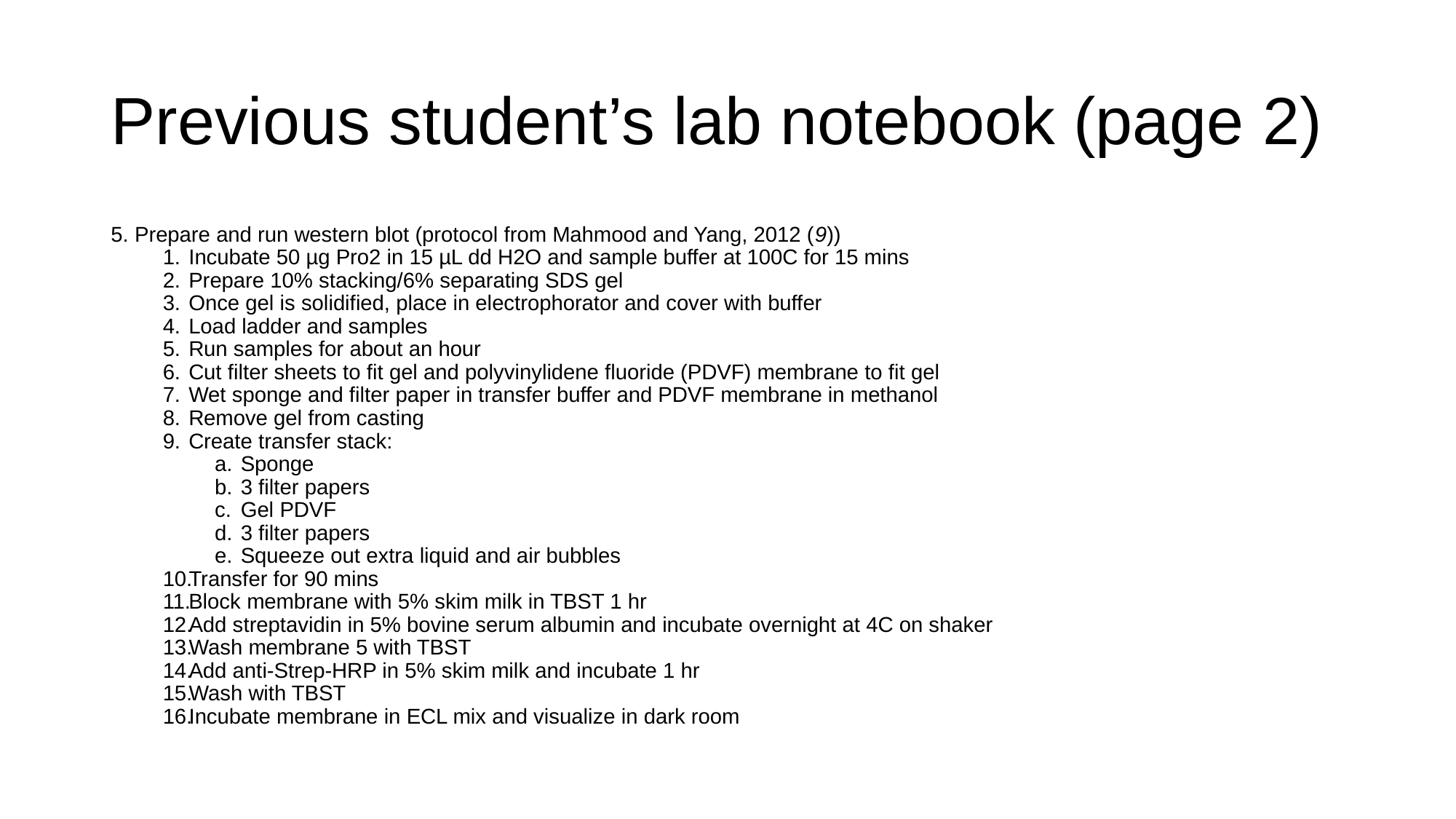

# Previous student’s lab notebook (page 2)
5. Prepare and run western blot (protocol from Mahmood and Yang, 2012 (9))
Incubate 50 µg Pro2 in 15 µL dd H2O and sample buffer at 100C for 15 mins
Prepare 10% stacking/6% separating SDS gel
Once gel is solidified, place in electrophorator and cover with buffer
Load ladder and samples
Run samples for about an hour
Cut filter sheets to fit gel and polyvinylidene fluoride (PDVF) membrane to fit gel
Wet sponge and filter paper in transfer buffer and PDVF membrane in methanol
Remove gel from casting
Create transfer stack:
Sponge
3 filter papers
Gel PDVF
3 filter papers
Squeeze out extra liquid and air bubbles
Transfer for 90 mins
Block membrane with 5% skim milk in TBST 1 hr
Add streptavidin in 5% bovine serum albumin and incubate overnight at 4C on shaker
Wash membrane 5 with TBST
Add anti-Strep-HRP in 5% skim milk and incubate 1 hr
Wash with TBST
Incubate membrane in ECL mix and visualize in dark room

## Slide 10
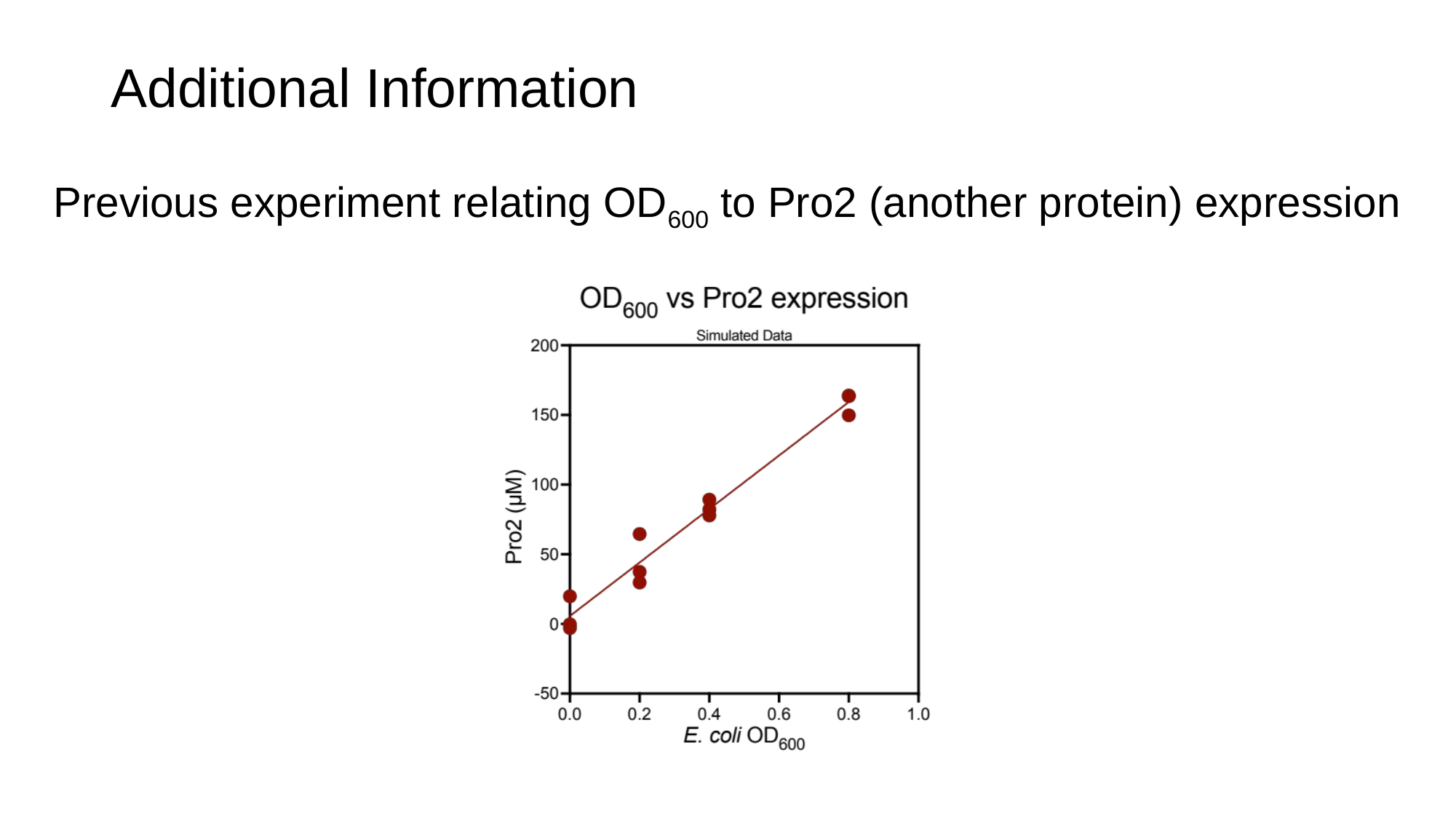

# Additional Information
Previous experiment relating OD600 to Pro2 (another protein) expression

## Slide 11
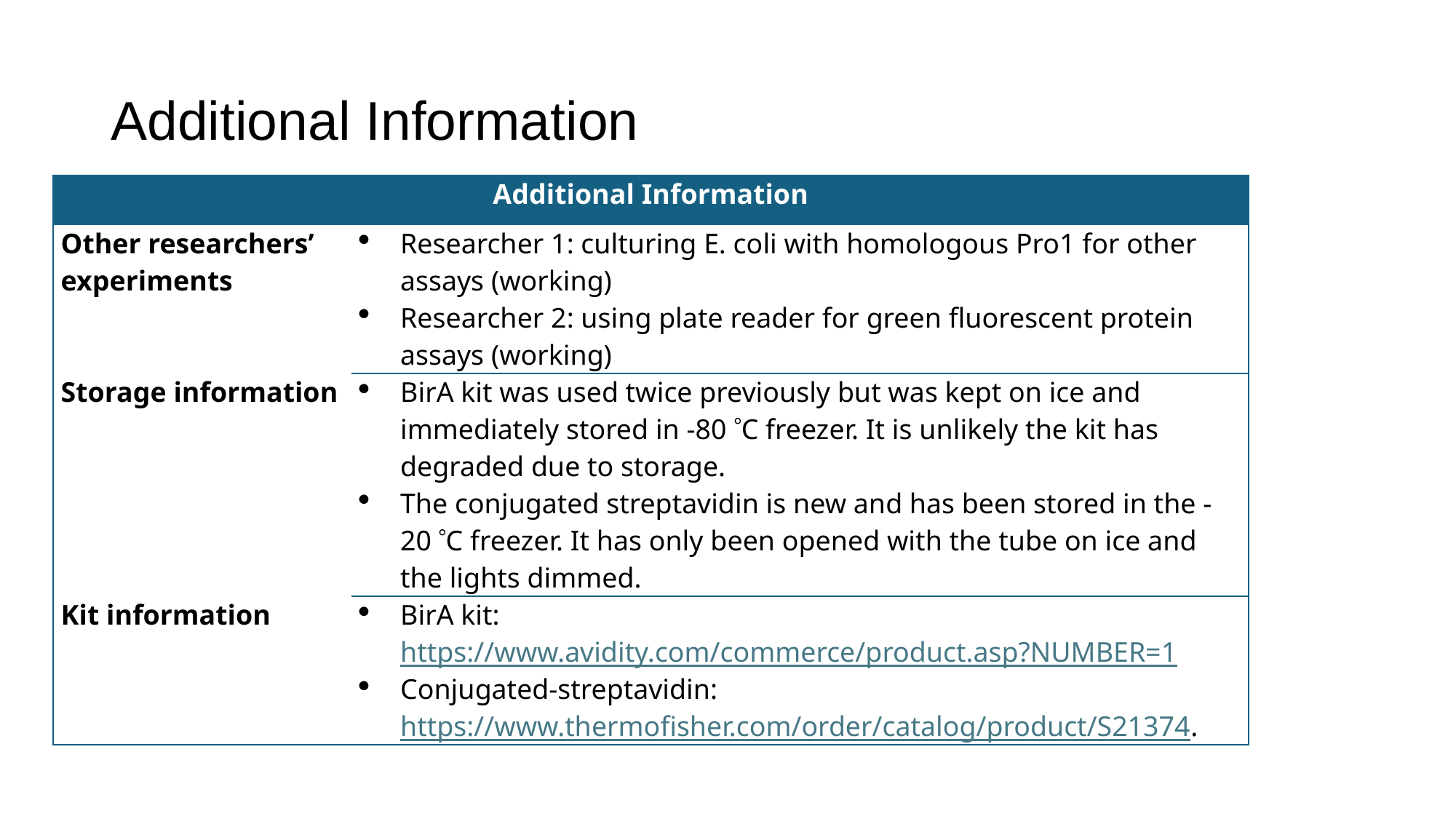

# Additional Information
| Additional Information | |
| --- | --- |
| Other researchers’ experiments | Researcher 1: culturing E. coli with homologous Pro1 for other assays (working) Researcher 2: using plate reader for green fluorescent protein assays (working) |
| Storage information | BirA kit was used twice previously but was kept on ice and immediately stored in -80 °C freezer. It is unlikely the kit has degraded due to storage. The conjugated streptavidin is new and has been stored in the -20 °C freezer. It has only been opened with the tube on ice and the lights dimmed. |
| Kit information | BirA kit: https://www.avidity.com/commerce/product.asp?NUMBER=1 Conjugated-streptavidin: https://www.thermofisher.com/order/catalog/product/S21374. |

## Slide 12
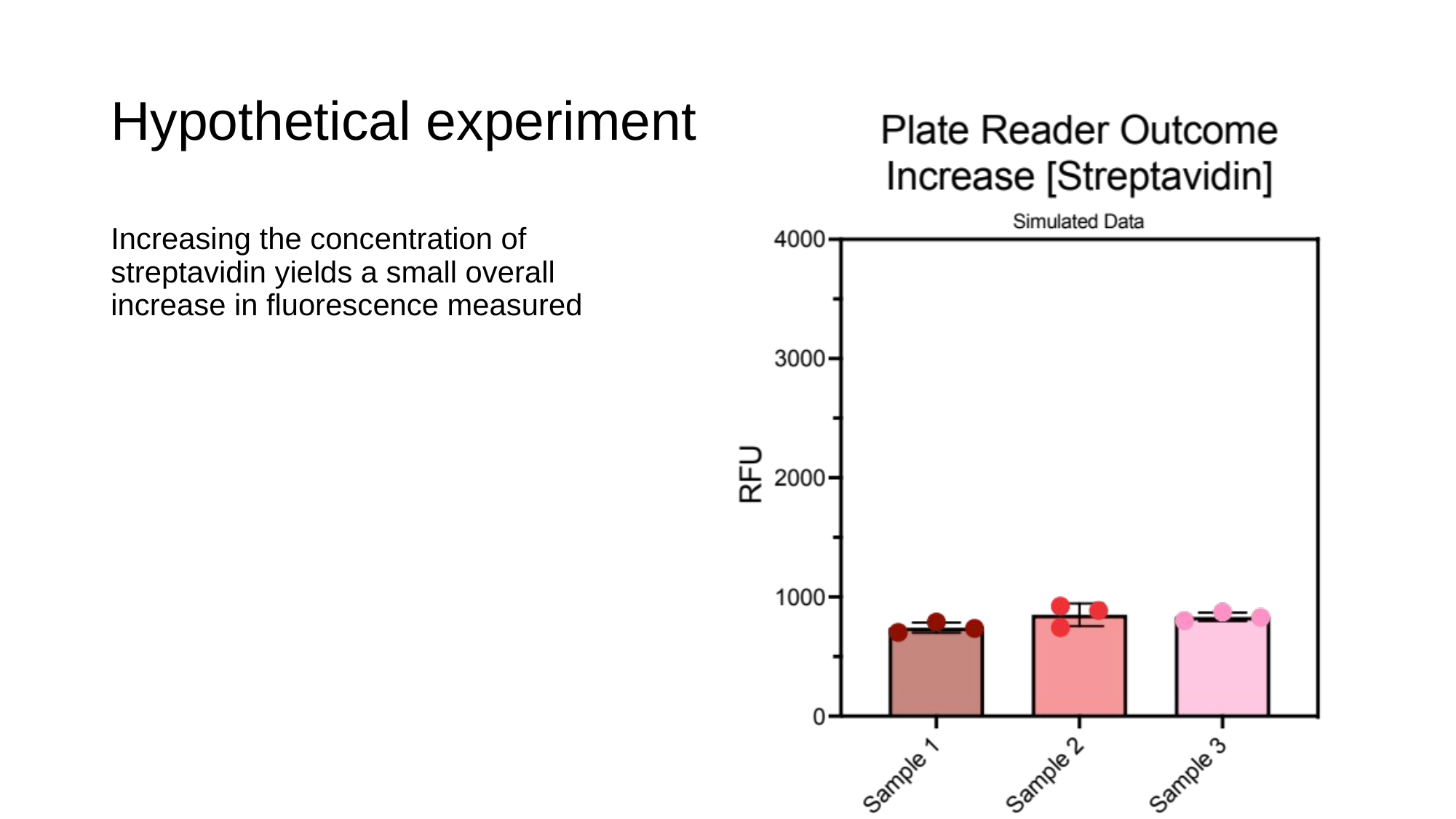

# Hypothetical experiment
Increasing the concentration of streptavidin yields a small overall increase in fluorescence measured

## Slide 13
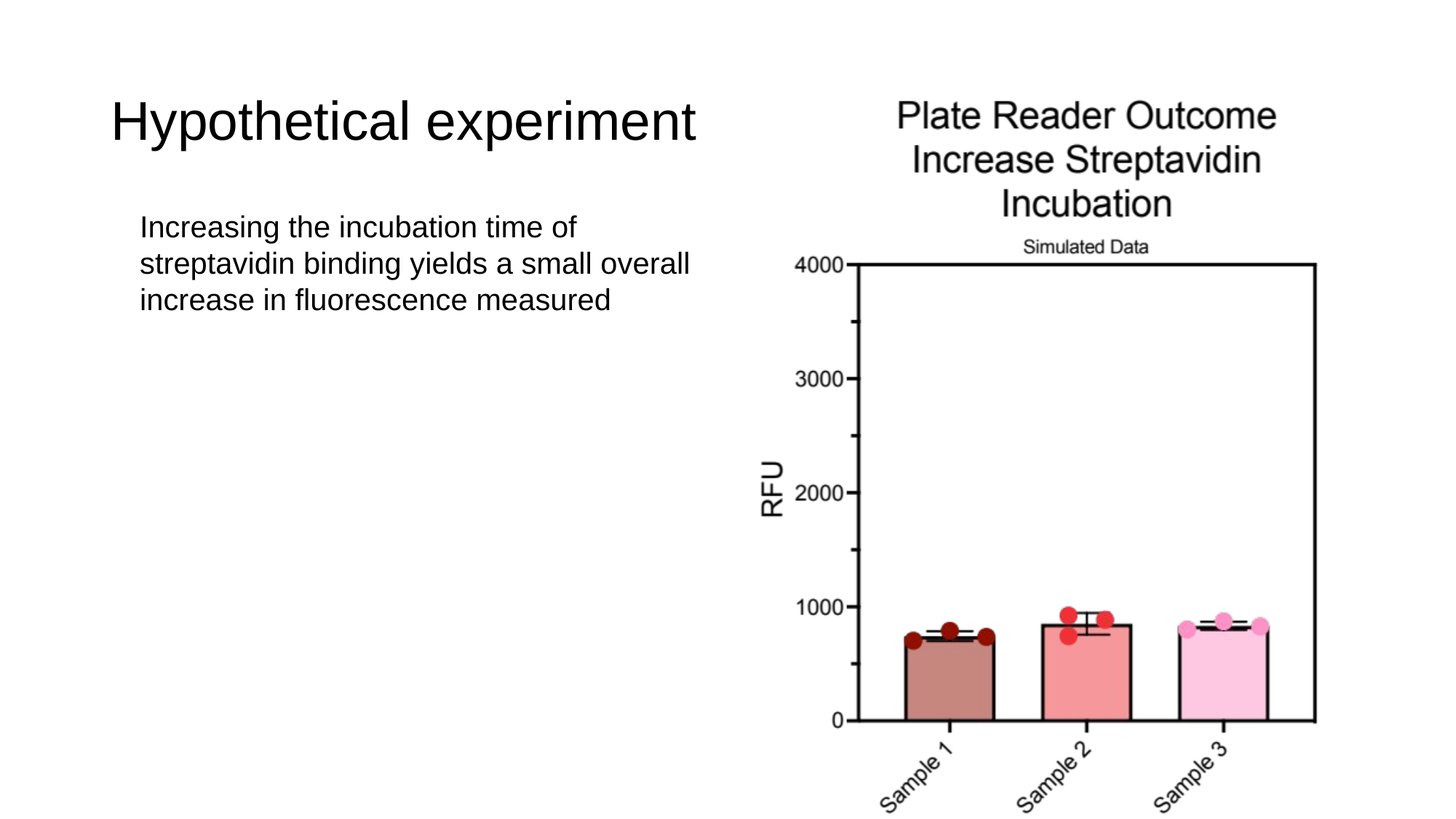

# Hypothetical experiment
Increasing the incubation time of streptavidin binding yields a small overall increase in fluorescence measured

## Slide 14
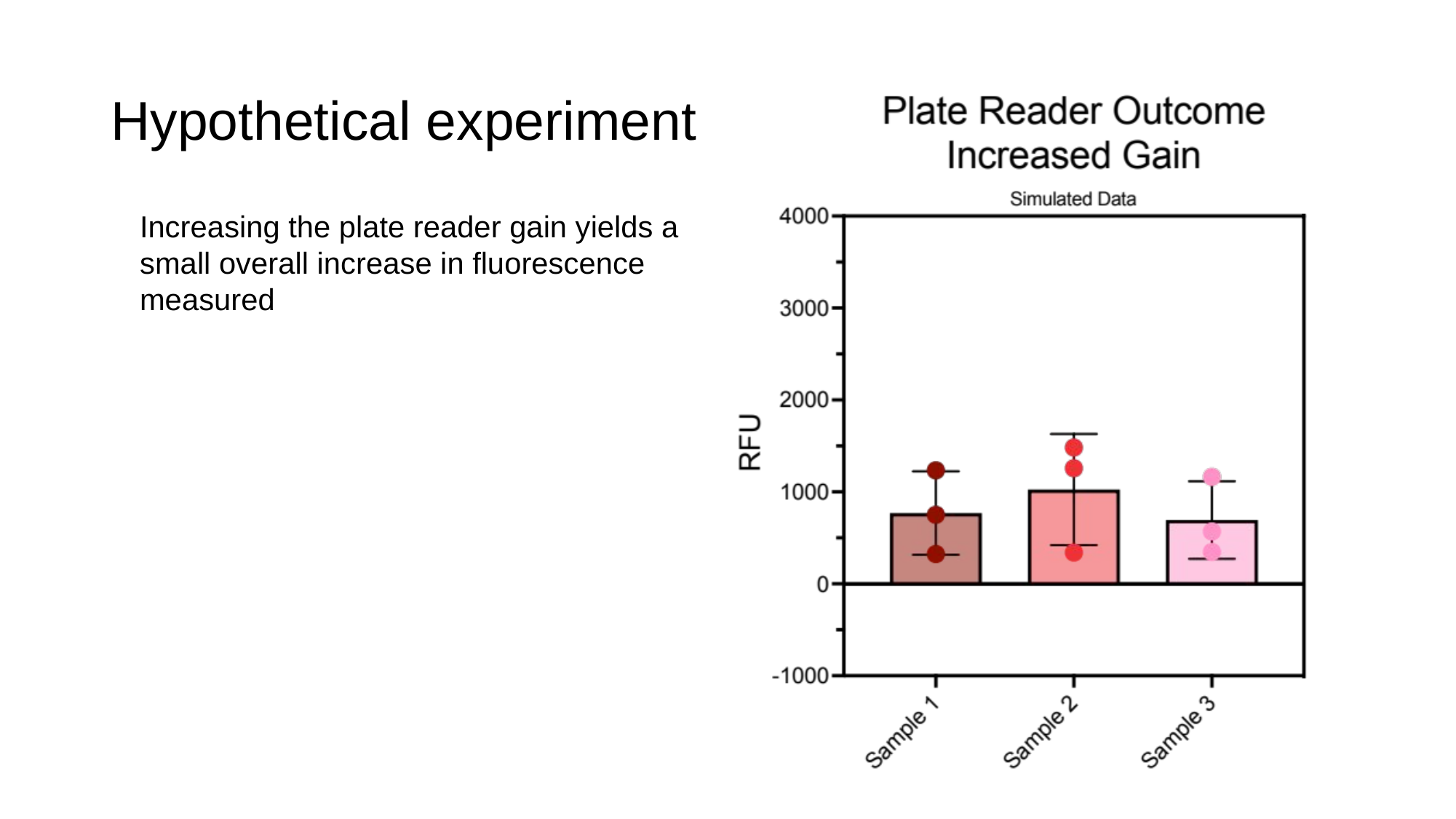

# Hypothetical experiment
Increasing the plate reader gain yields a small overall increase in fluorescence measured

## Slide 15
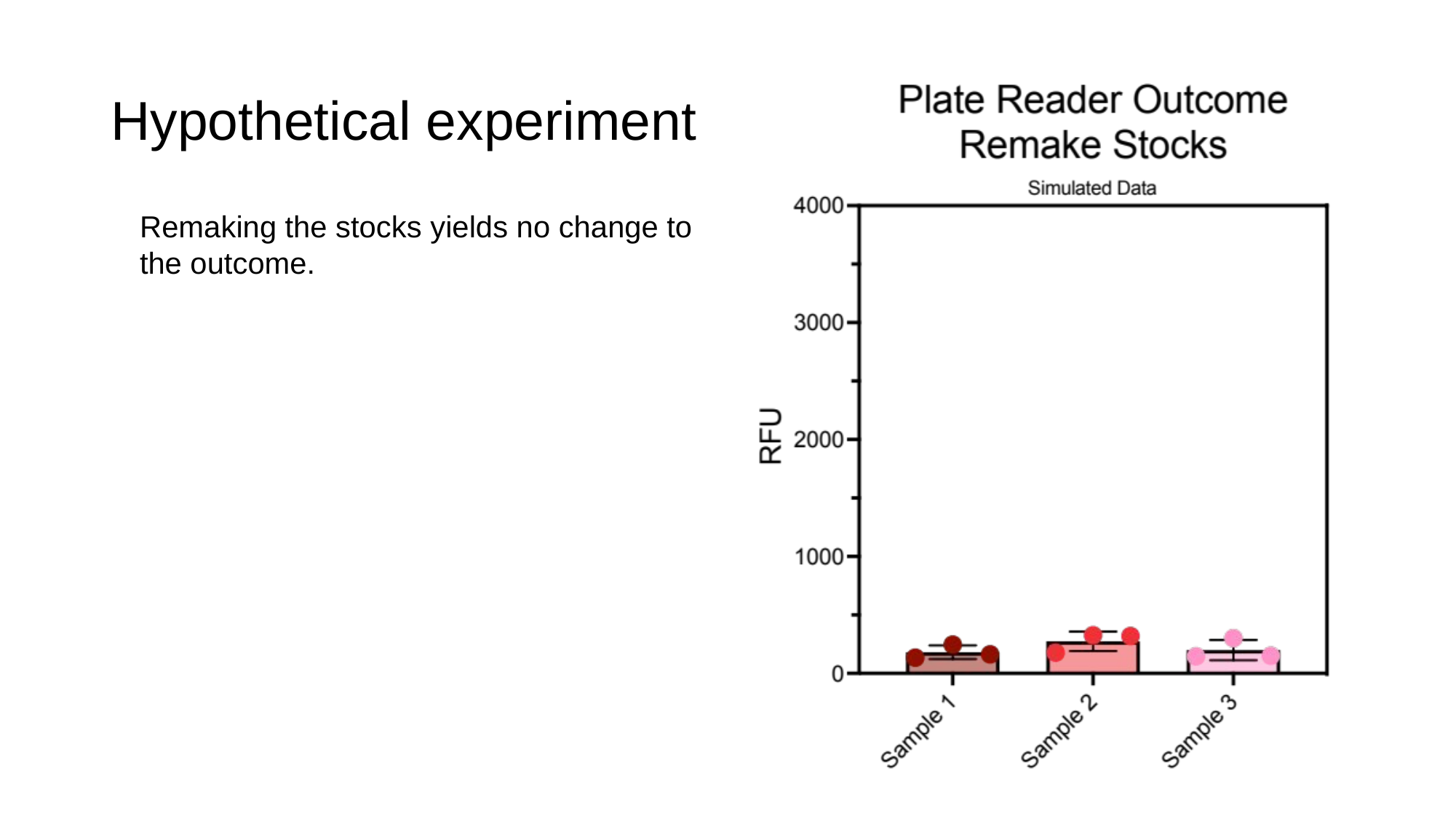

# Hypothetical experiment
Remaking the stocks yields no change to the outcome.

## Slide 16
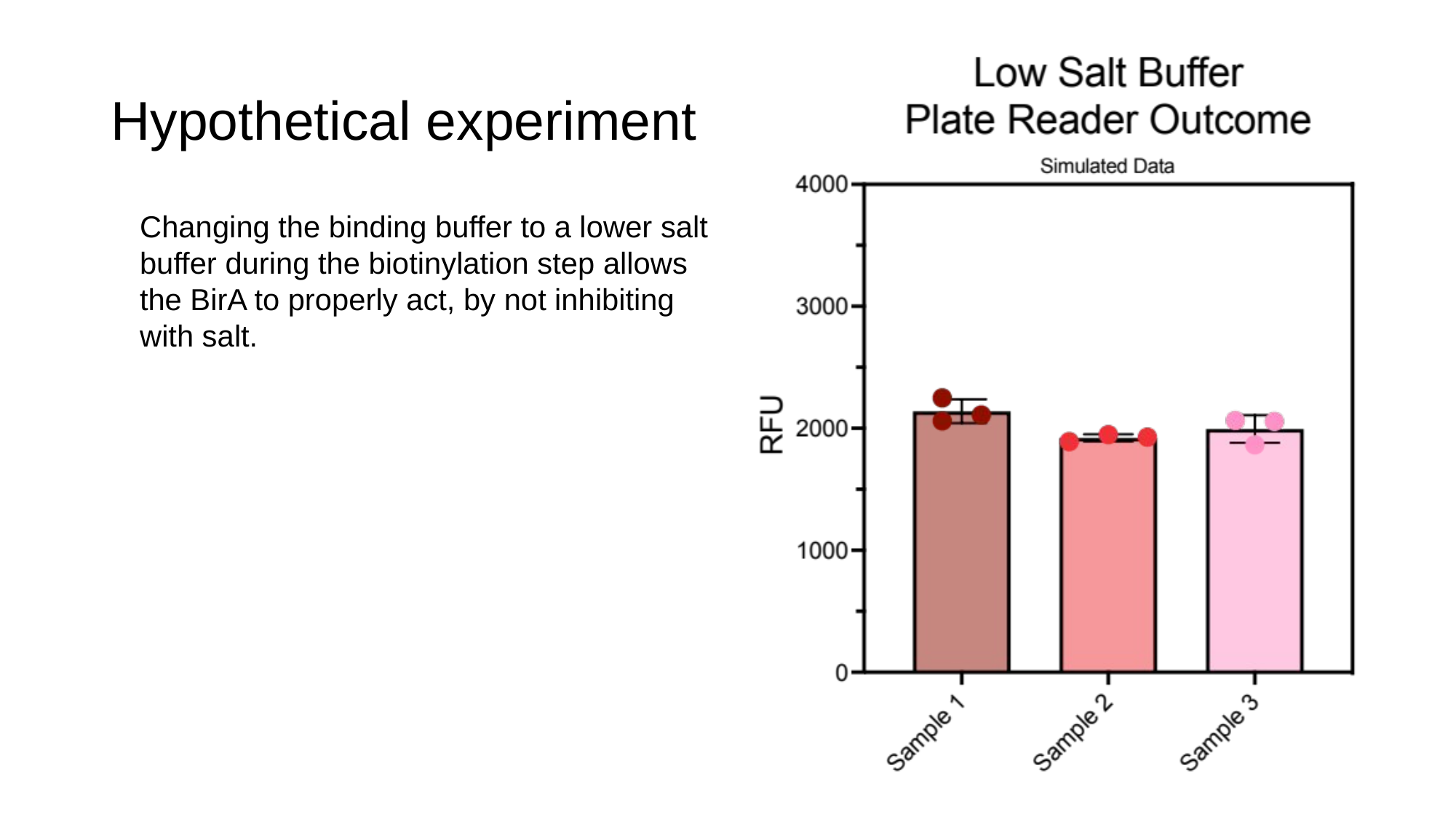

# Hypothetical experiment
Changing the binding buffer to a lower salt buffer during the biotinylation step allows the BirA to properly act, by not inhibiting with salt.

## Slide 17
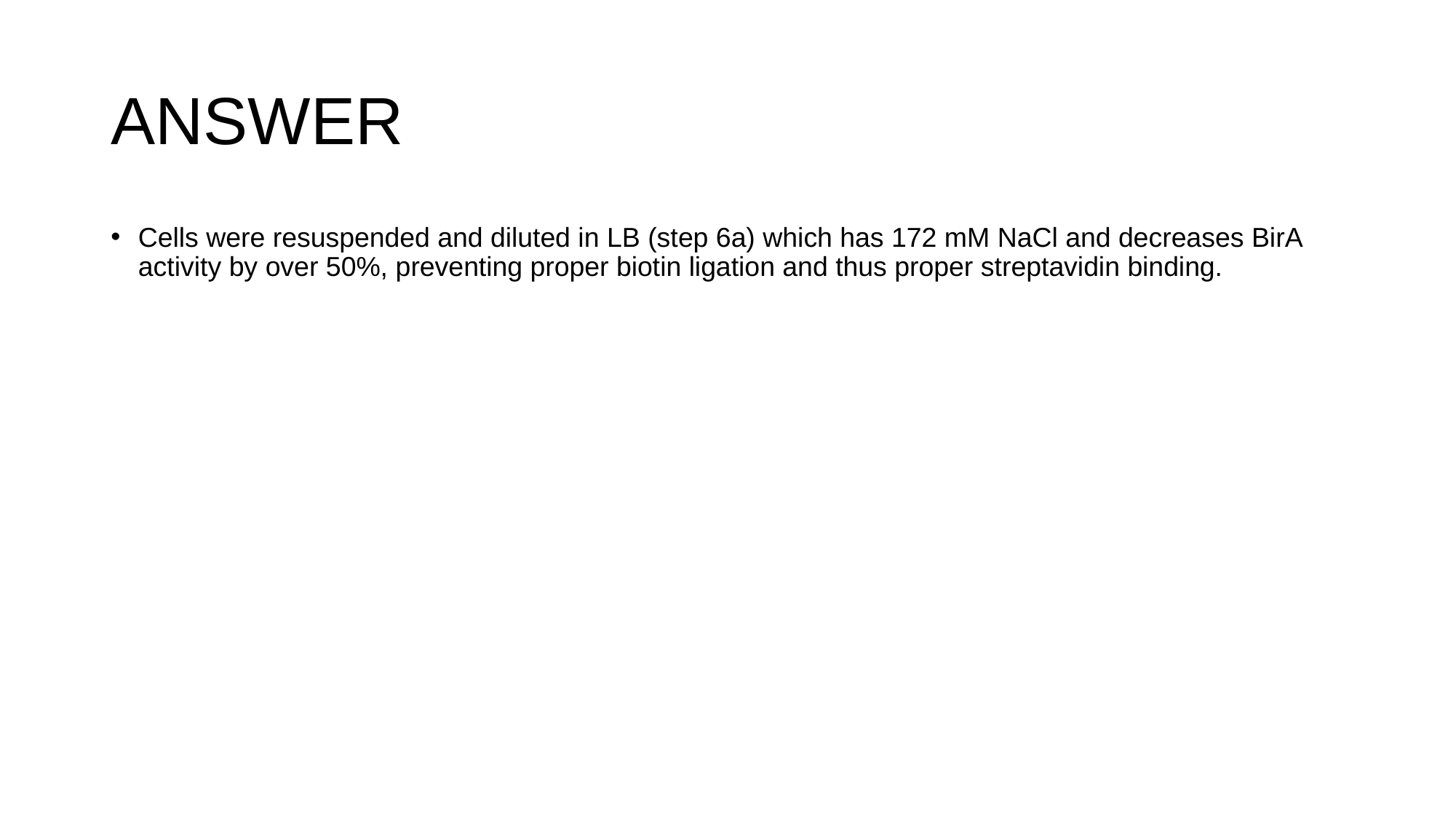

# ANSWER
Cells were resuspended and diluted in LB (step 6a) which has 172 mM NaCl and decreases BirA activity by over 50%, preventing proper biotin ligation and thus proper streptavidin binding.
