# Supplementary material for: Teaching troubleshooting skills to graduate students
Source: eLife. 2024 Sep 17;13:e100761. doi: 10.7554/eLife.100761 (PMC11407763; doi:10.7554/eLife.100761)
Supplement: Supplementary file 1. — For each scenario there is a Word file that contains the following: background information; a description of the scenario; the protocol for the experiment that produced the unexpected result; the results of the experiment; information on the source of the error; background information that can be used to answer questions; and references. There is also a PowerPoint file for each scenario that contains example slides that can be used in real meetings. There are also templates for the Word and PowerPoint files. [file elife-100761-supp1.zip › Final Scenarios/Example5.docx]

**Recombinant Quorum Sensing Expression and Activity Verification**

Keywords: quorum sensing, synthetic biology, plasmid cloning, structural biology

**Background:**

In *Pseudomonas aeruginosa*, the LasIR quorum sensing system allows for collective gene expression in bacterial communities. The protein LasI synthesizes an autoinducer, a diffusible acyl-homoserine lactone (AHL), and LasR acts a transcriptional activator upon binding to the AHL. At low cell density, the AHL signal is present at basal levels in the environment, but as population size grows, AHL becomes more abundant and reaches a threshold concentration leading to gene expression driven by a LuxR/AHL complex^5^.

The goal of this assay is to confirm the successful recombinant plasmid-based expression of the LasIR system in *E. coli* DH5⍺, which does not express this system or any orthogonal quorum sensing^3^. The assay involves concentrating a population of “sender” cells containing a plasmid with an inducible copy of LasI onto a lawn of “receiver” cells containing a plasmid with constitutively expressed LasR driving the expression of gfp in the presence of AHL. If the assay is successful, a “halo” of fluorescence should be observable around the “sender” cell population, confirming that LasI has been expressed and functions correctly in *E. coli* as an AHL synthase.

**Scenario:**

You are a researcher interested in assaying the activity of the LasIR system driven by newly constructed and transformed recombinant plasmids in *E. coli* using the method described above. You have successfully performed this assay multiple times with a homologous quorum sensing system, so you expect similar results. You are aware that this system is not as robust. After performing the assay three times, you see no fluorescence under any conditions, induced or uninduced.


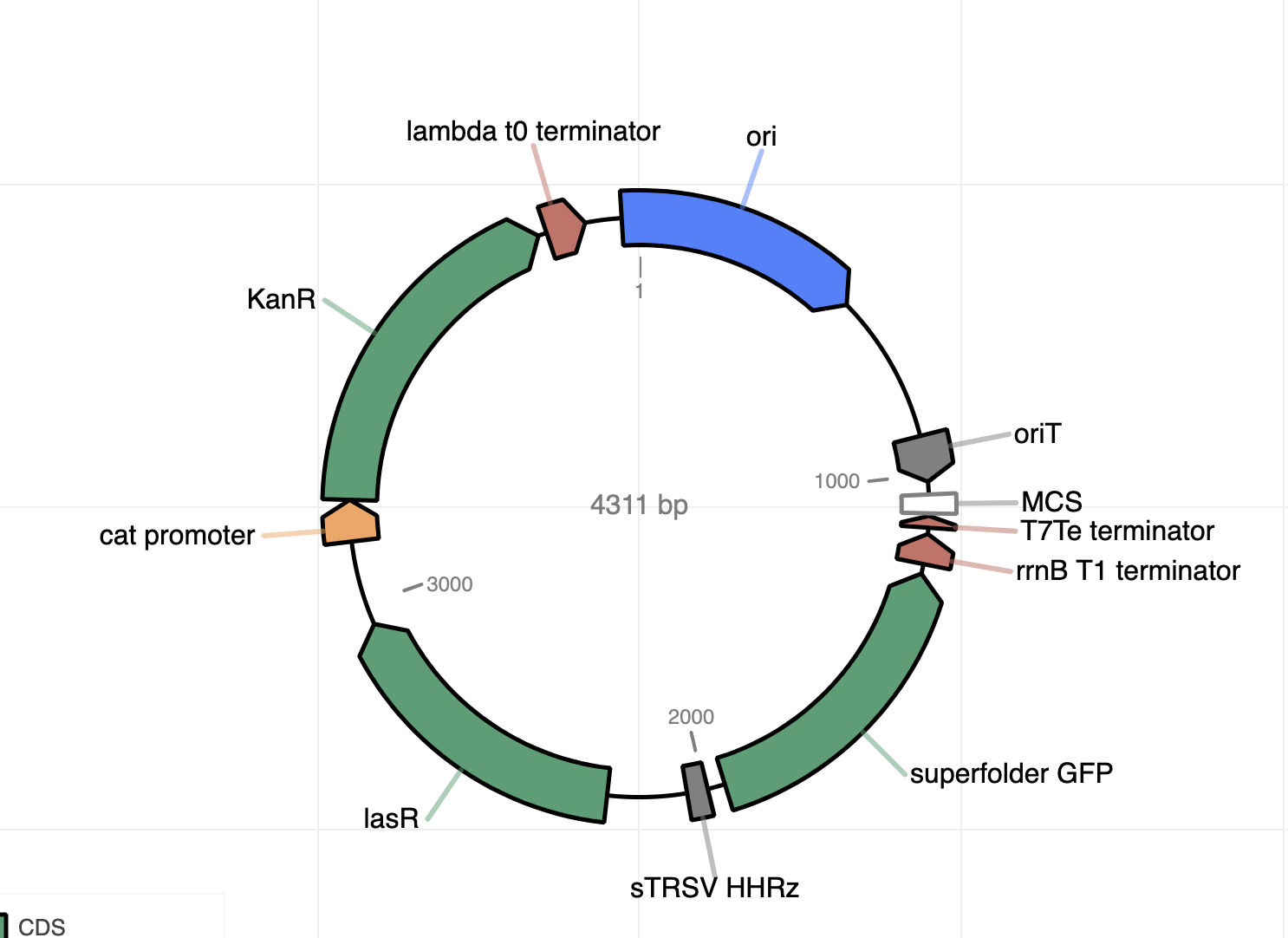
**
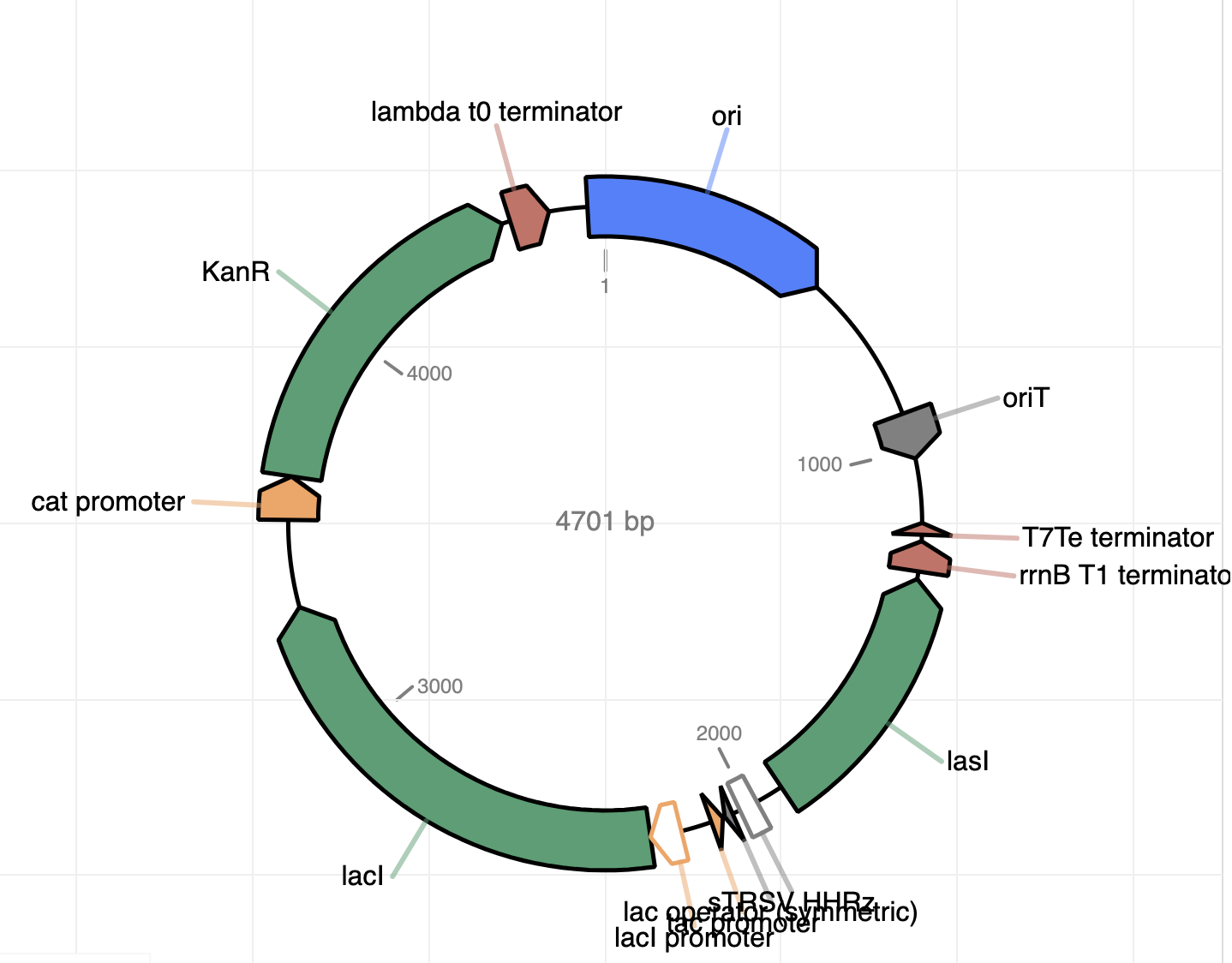
**

**Sender plasmid**

Addition of IPTG leads to expression of LasI which synthesizes OC12.

**Receiver plasmid**

Addition of OC12 leads to expression of gfp.

**Protocol:**

1. From a single colony, start two separate 5 mL cultures of receiver and sender cells in minimal media with the appropriate antibiotic for plasmid selection and maintenance. Allow to grow overnight at 30 ℃ with shaking at 250 rpm
2. Make 1.5% agarose plates containing the appropriate antibiotic for plasmid selection and maintenance
3. The next day, take an absorbance measurement at a wavelength of 600 nm to confirm healthy growth and to normalize cell density across samples.
4. Wash 1 mL aliquots of the receiver by pelleting at 6000 rcf for 5 minutes, discarding the supernatant, and resuspending the pellet with fresh minimal media. Repeat for a total of three washes.
5. Dilute the sender cells to an OD_600_ of 0.15 in 3 mL of 0.7% agarose and plate evenly onto the agarose plate.
6. For each experimental condition, (in this case uninduced and induced sender cells), wash 1 mL aliquots of the sender cells at an OD_600_ of 0.3 by pelleting at 6000 rcf for 5 minutes, discarding the supernatant, and resuspending the pellet with fresh minimal media. Repeat for a total of three washes.
7. After the final wash, resuspend the sender cell pellet in 20 µL of minimal media containing the appropriate antibiotic and variable concentration of IPTG (inducer) at either 0 mM or 1 mM.
8. Pipette the concentrated cells onto a 5 mm disk of filter paper and place the disc onto the receiver cell lawn on the agarose plate.
9. Incubate overnight at 30 ℃.
10. To visualize fluorescence, place the agarose plates onto a UV transilluminator and image.

**Materials used in protocol:**

1. Shewanella Basal Media + 0.05% cas amino acids (pH 7.2)
2. 60% lactate (filter sterilized)
3. 1 M fumarate (filter sterilized)
4. 25 mg/mL kanamycin in sterile milliQ water
5. Agarose
6. 100 mL beaker
7. Hot plate
8. Stir bar
9. 1 M IPTG in sterile water
10. Filter paper
11. Hole punch
12. Plate reader for OD600 and fluorescence measurements

**Available materials for further experiments:**

1. Purified OC12 in DMF (OC12 is the autoinducer produced by the sender cells, it should induce fluorescence in the receiver cells)
2. Nickel resin column and buffers for HisTag purification
3. Sequence alignment tools and plasmid maps


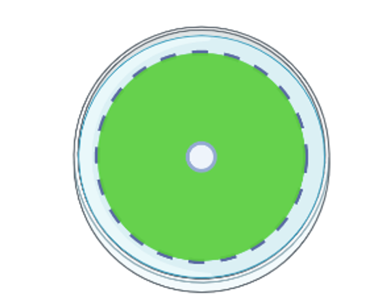
**Results:**


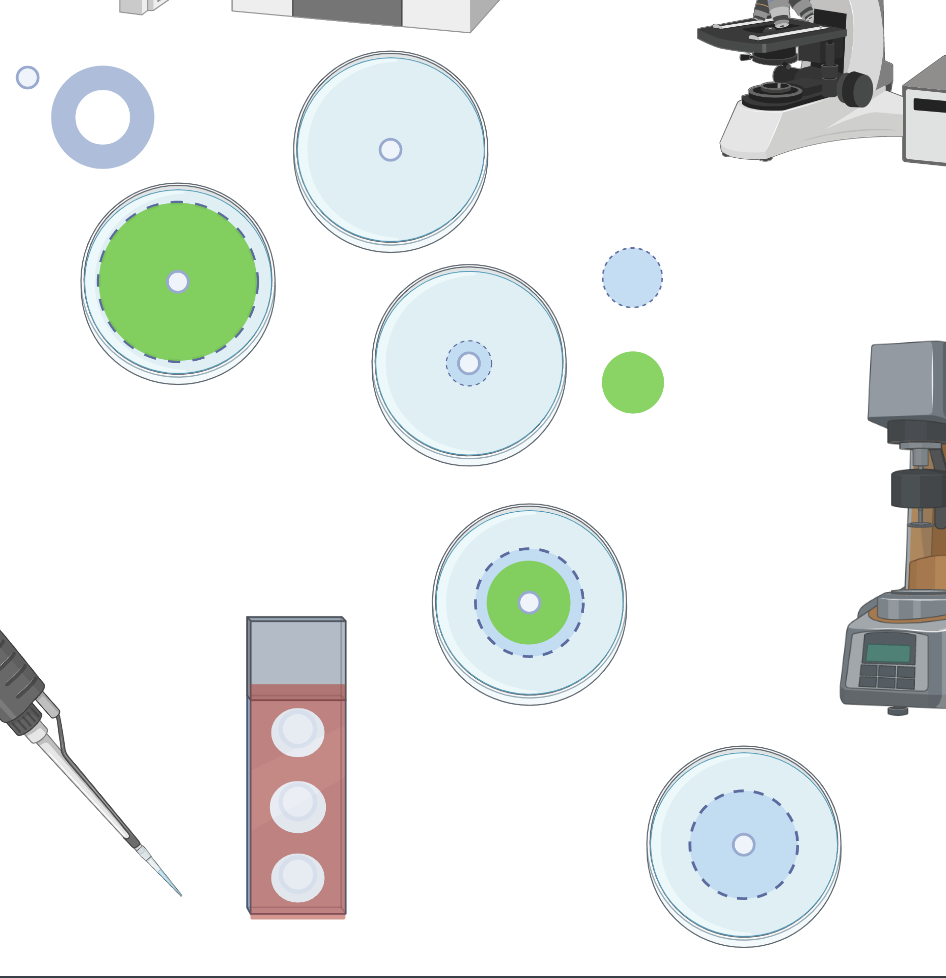

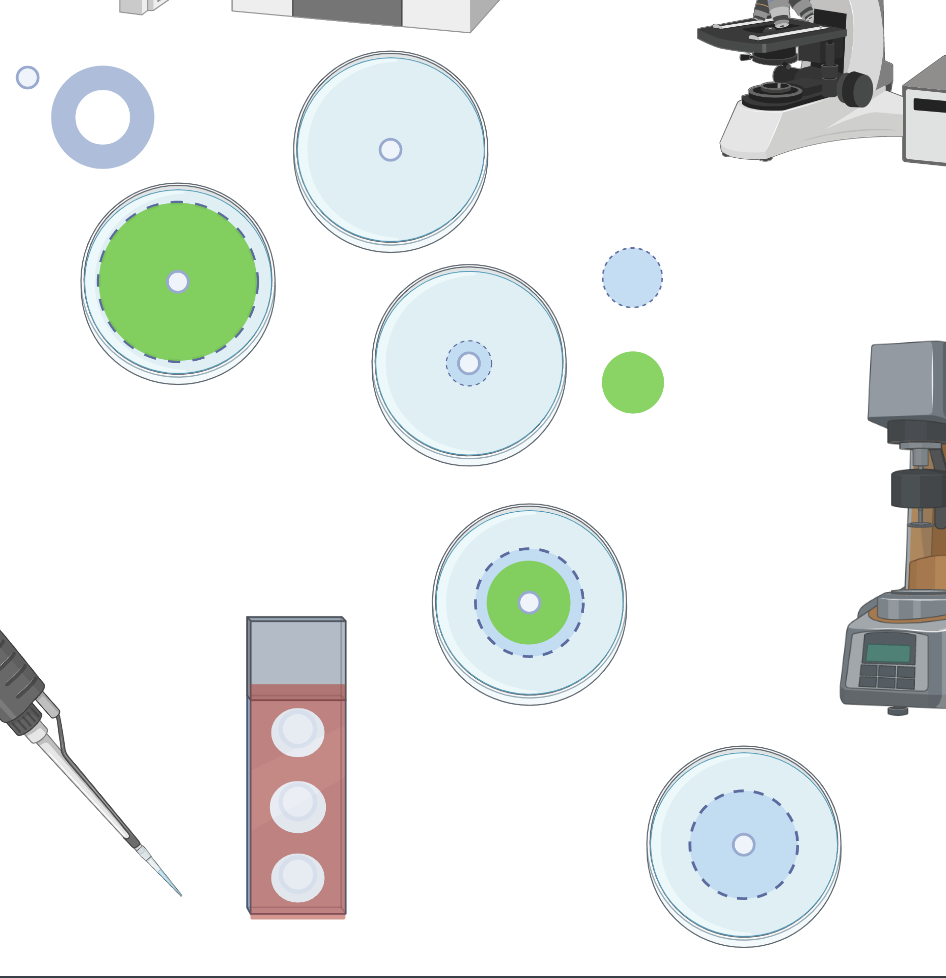


**Expected “receiver” fluorescence expression pattern** from induced “sender” cells using a homologous quorum sensing system

**LasIR experiment results.** Left plate has uninduced “sender” cells and right plate has fully induced “sender” cells

**Details:**

After sequence verification, the LasI sequence was amplified from Addgene plasmid pLux76-LasI^2^. A C-terminal 10x HisTag was added and the modified LasI was cloned into an IPTG-inducible vector.

After sequence verification, the LasR protein and promoter sequences were amplified from the Addgene plasmid Bsrs078-LasR^6^ and cloned into a *gfp*-containing vector.

Sequence verification was performed using whole-plasmid nanopore sequencing and the pLannotate tool to confirm plasmid size and annotate expected components.

pLannotate showed plasmids of the expected size. LasI had 99% sequence identity to sequence databases. LasR has 100% sequence identity to sequence databases.

**Source of error:**

A random mismatch introduced during PCR amplification results in the mutation L72F. This mutation creates a pi-cation interaction between the errant negatively charged phenylalanine and a nearby positively charged alanine that is critical in a coordination system required for AHL synthesis^1^. This was discovered by performing pairwise sequence alignment of the Addgene plasmid sequence and the final plasmid sequencing result. ChimeraX and Blender were used to visualize the mutation. A simple point mutation fixing the phenylalanine back to a neutral leucine recovers the function of the protein.

**Relevant Resources**

LasI UniProt Entry –

<https://www.uniprot.org/uniprotkb/P33883/entry>

ChimeraX Download–

<https://www.cgl.ucsf.edu/chimerax/>

Modeling mutations from existing structures in ChimeraX-

https://www.cgl.ucsf.edu/chimerax/docs/user/commands/swapaa.html

**Table 1.** Additional information known by the leader that can be provided upon request

| **Meeting Notes for the Leader**  Not to be shared with the group | |
| --- | --- |
| Other researcher’s experiments | - One researcher is working on plasmid construction (PCR, DNA extraction, agarose gels, Golden Gate assembly, etc.) - Another researcher is synthesizing methacrylated agarose in the fume hood across the lab - A third researcher is teaching their undergraduate mentee how to perform colony forming units counts |
| Storage information | - All stocks were freshly prepared under flame in autoclaved containers and stored at 4°C after filter sterilization - OC12 stock was stored at -20°C - Minimal media agarose plates were freshly poured before every experiment |
| Construct information | - The sender construct contains a ColE1 origin of replication, kanamycin resistance, constitutively expressed LacI, and the LasI (OC12 AHL synthase) gene under the inducible pTac promoter [**IPTG → OC12**] - The receiver construct contains a ColE1 origin of replication, kanamycin resistance, constitutively expressed LuxR, and the gfp (fluorescent protein) gene under the inducible pLux promoter [**OC12 → Fluorescence**] |
| Source of error | - Point mutation introduced during LasI gene amplification for sender construct assembly disrupts a system of electrostatic interactions necessary for OC12 AHL synthesis. In short, the sender plasmid is expressing the LasI protein, but the protein is inactive and unable to produce the signal to turn on fluorescence in the receiver cells. |
| Hints for group | - You have access to the expected sequence and sequencing results - You have chemically synthesized OC12 (receiver cell inducer) stocks available - LasI has a HisTag |

**Works Cited**

1. Gould, T. A., Schweizer, H. P., & Churchill, M. E. (2004). Structure of the *Pseudomonas aeruginosa* acyl‐homoserine lactone synthase LasI. *Molecular Microbiology*, *53*(4), 1135–1146.
2. Grant, P. K., Dalchau, N., Brown, J. R., Federici, F., Rudge, T. J., Yordanov, B., Patange, O., Phillips, A., & Haseloff, J. (2016). Orthogonal intercellular signaling for programmed spatial behavior. *Molecular Systems Biology*, *12*(1).
3. Surette, M. G., Miller, M. B., & Bassler, B. L. (1999). Quorum sensing in Escherichia coli, Salmonella typhimurium, and Vibrio harveyi: A new family of genes responsible for autoinducer production. *Proceedings of the National Academy of Sciences of the United States of America*, *96*(4), 1639-1644.
4. McGuffie,M.J. and Barrick,J.E. (2021) pLannotate: engineered plasmid annotation. *Nucleic Acids Research*
5. Pesci, E. C., Pearson, J. P., Seed, P. C., & Iglewski, B. H. (1997). Regulation of las and rhl quorum sensing in pseudomonas aeruginosa. *Journal of Bacteriology*, *179*(10), 3127–3132. https://doi.org/10.1128/jb.179.10.3127-3132.1997
6. Scott, S. R., & Hasty, J. (2016). Quorum Sensing Communication Modules for Microbial Consortia. *ACS Synthetic Biology*, *5*(9), 969–977. https://doi.org/10.1021/acssynbio.5b00286
