# Supplementary material for: Teaching troubleshooting skills to graduate students
Source: eLife. 2024 Sep 17;13:e100761. doi: 10.7554/eLife.100761 (PMC11407763; doi:10.7554/eLife.100761)
Supplement: Supplementary file 1. — For each scenario there is a Word file that contains the following: background information; a description of the scenario; the protocol for the experiment that produced the unexpected result; the results of the experiment; information on the source of the error; background information that can be used to answer questions; and references. There is also a PowerPoint file for each scenario that contains example slides that can be used in real meetings. There are also templates for the Word and PowerPoint files. [file elife-100761-supp1.zip › Final Scenarios/Example4.pptx]

## Slide 1
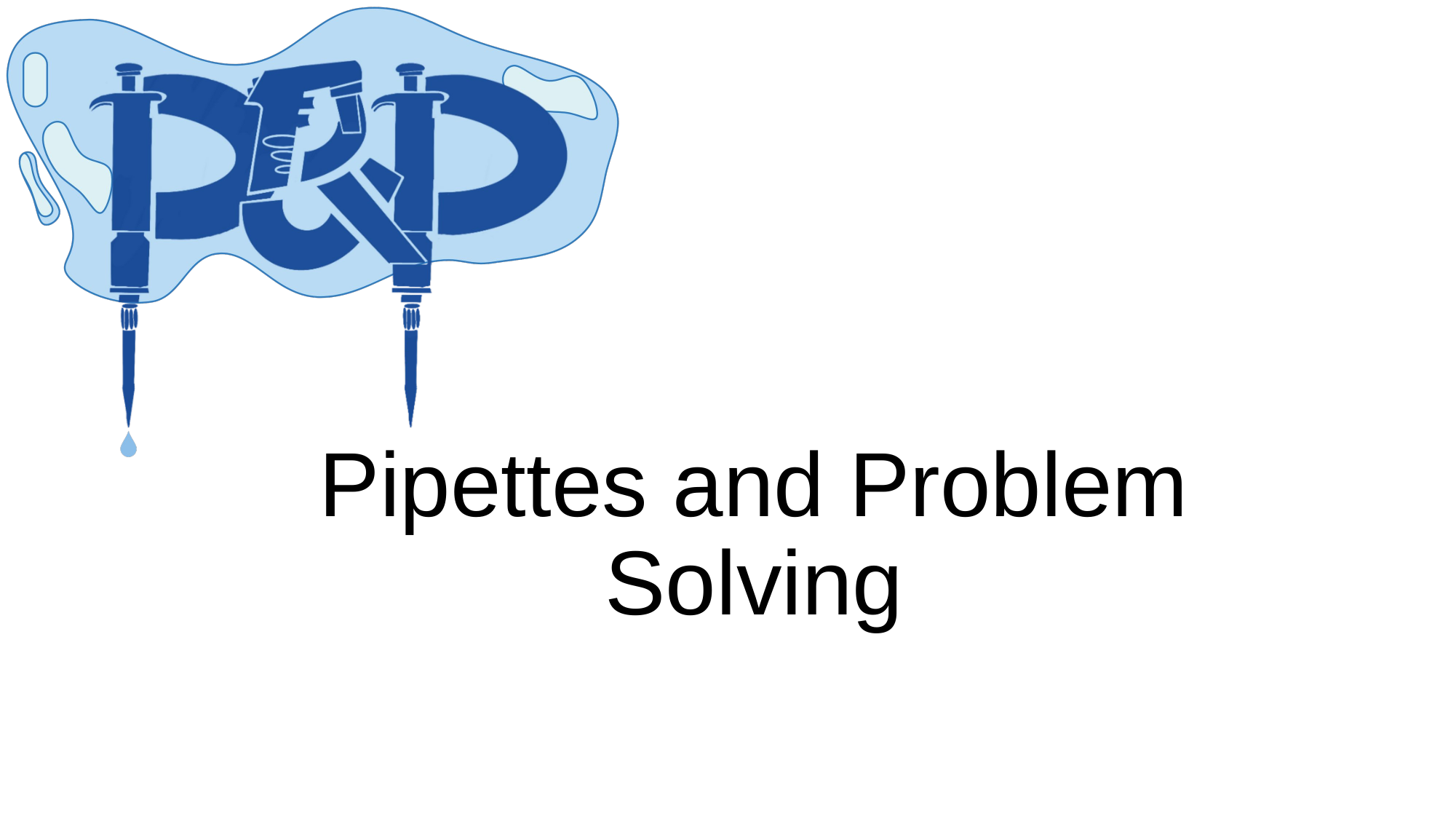

# Pipettes and Problem Solving

## Slide 2
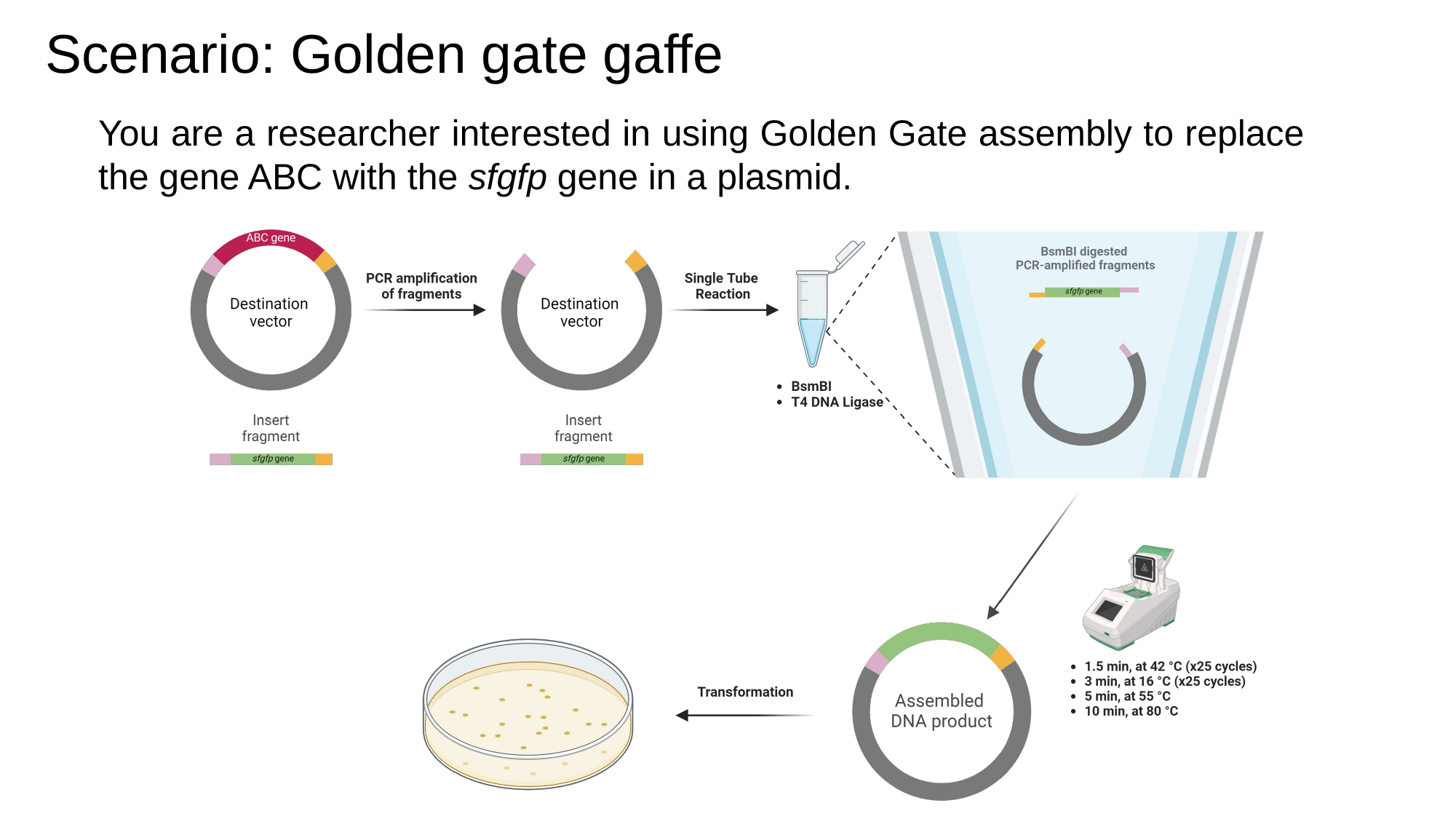

Scenario: Golden gate gaffe
You are a researcher interested in using Golden Gate assembly to replace the gene ABC with the sfgfp gene in a plasmid.

## Slide 3
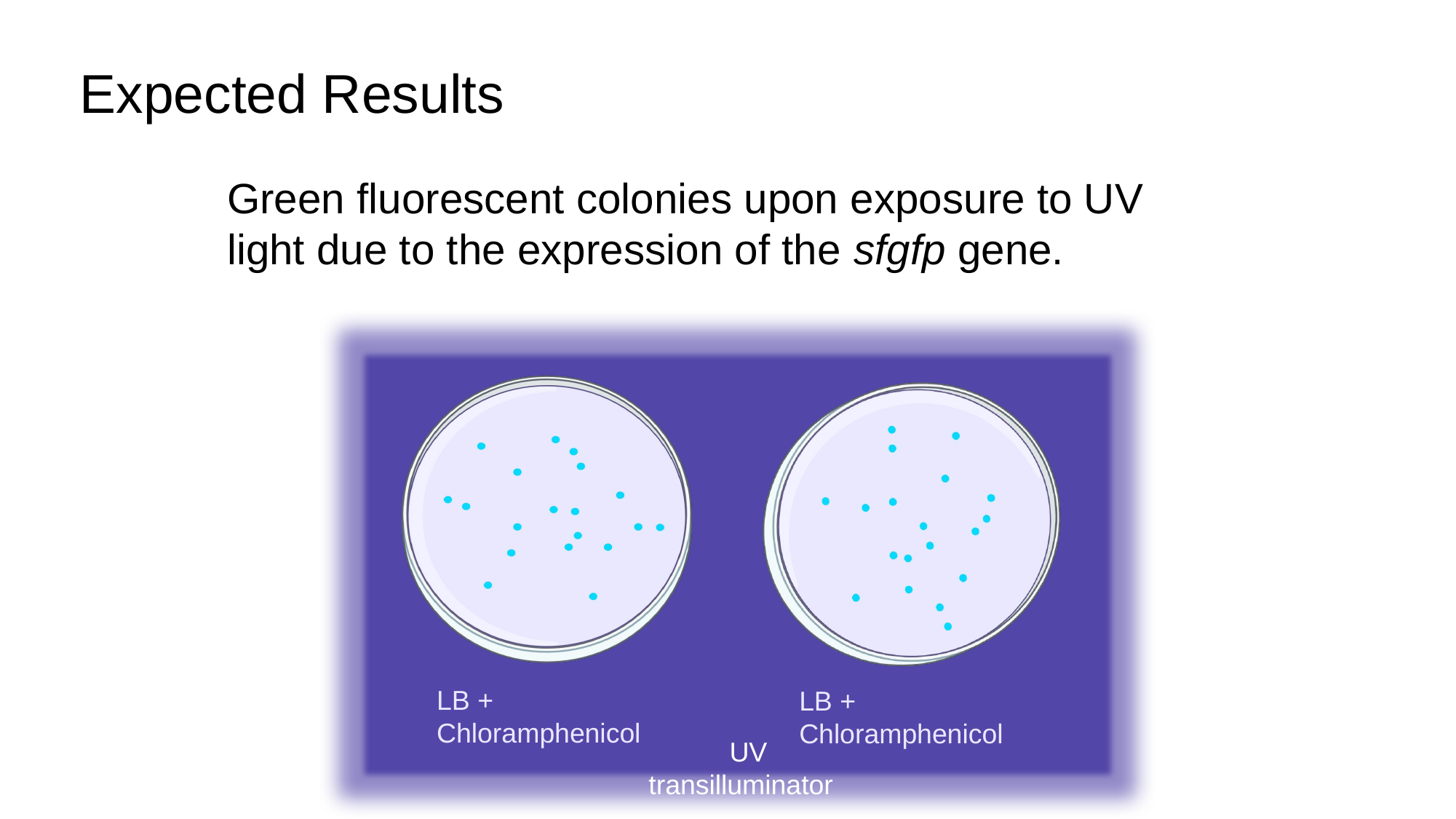

Expected Results
Green fluorescent colonies upon exposure to UV light due to the expression of the sfgfp gene.
LB + Chloramphenicol
LB + Chloramphenicol
UV transilluminator
UV transilluminator

## Slide 4
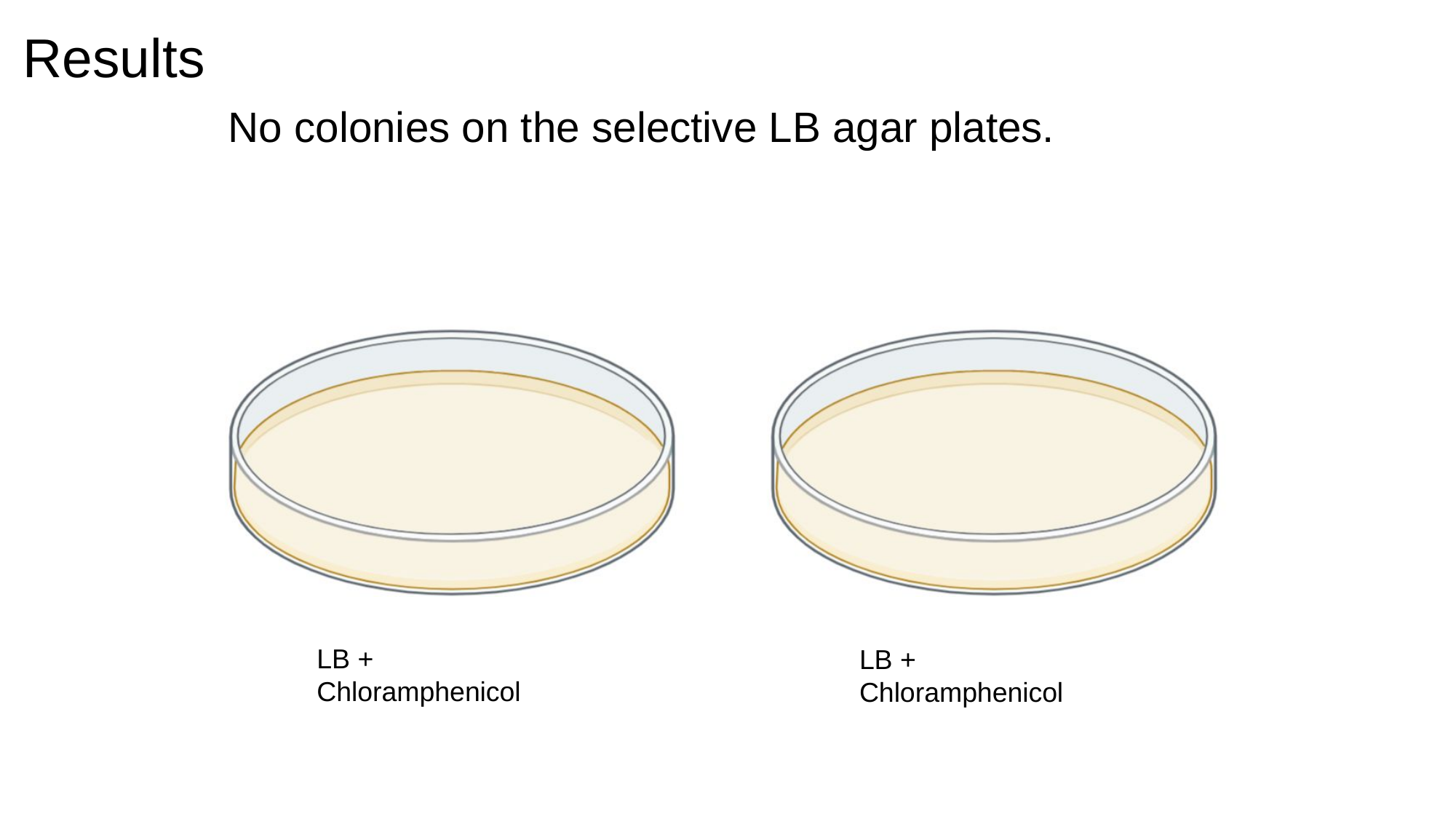

Results
No colonies on the selective LB agar plates.
LB + Chloramphenicol
LB + Chloramphenicol

## Slide 5
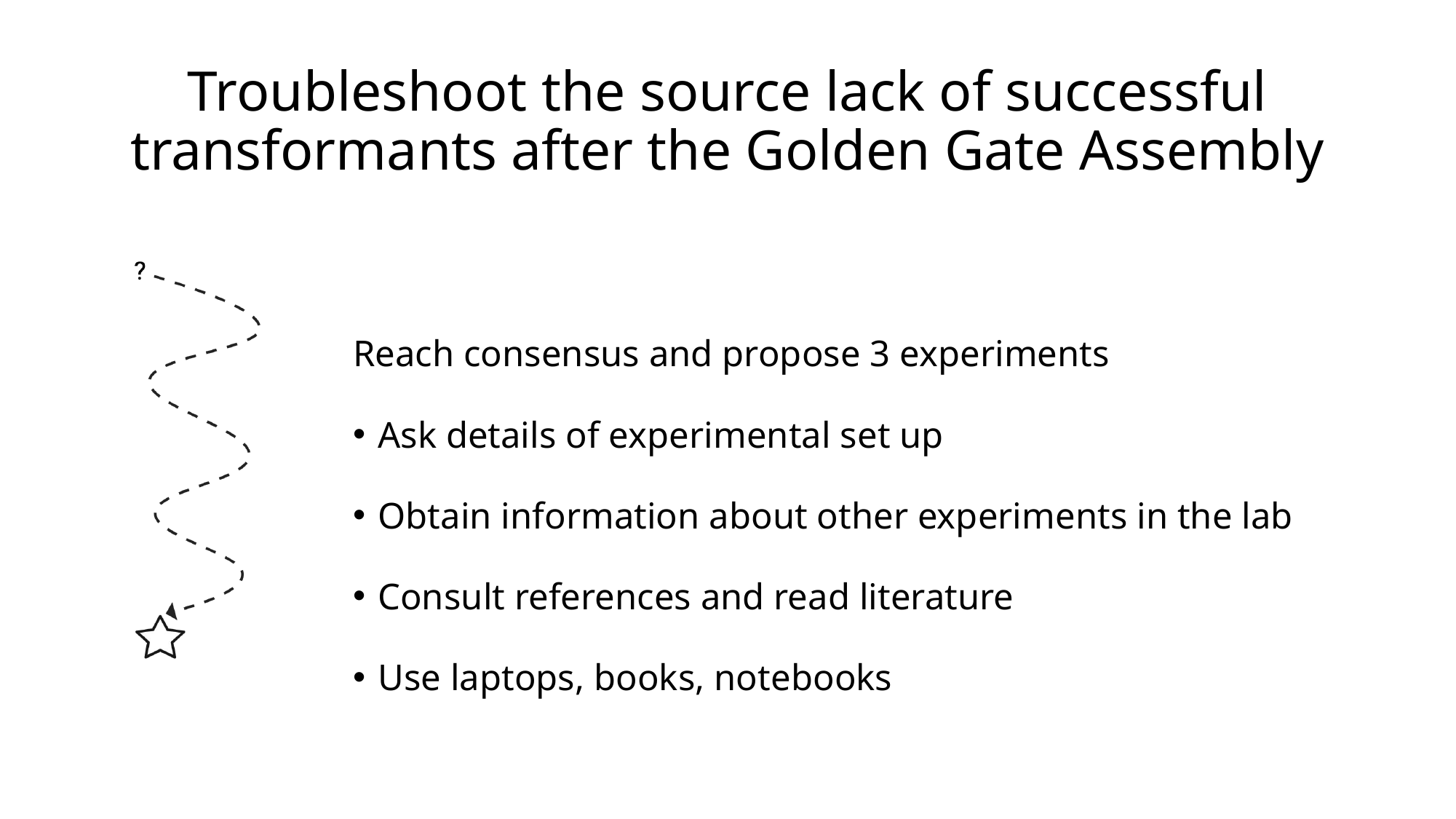

# Troubleshoot the source lack of successful transformants after the Golden Gate Assembly
Reach consensus and propose 3 experiments
Ask details of experimental set up
Obtain information about other experiments in the lab
Consult references and read literature
Use laptops, books, notebooks

## Slide 6
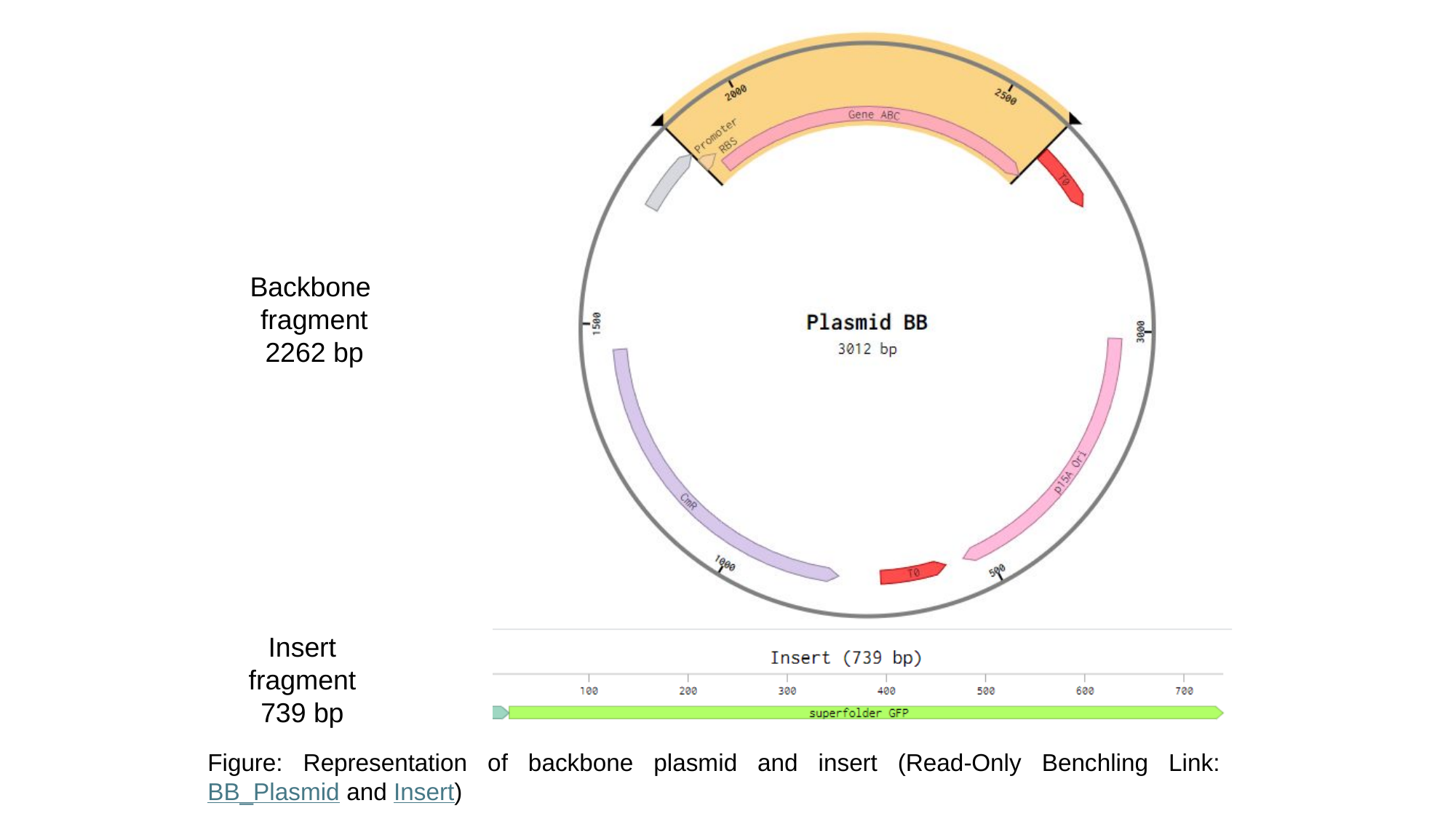

Backbone
fragment
2262 bp
Insert
fragment
739 bp
Figure: Representation of backbone plasmid and insert (Read-Only Benchling Link: BB_Plasmid and Insert)

## Slide 7
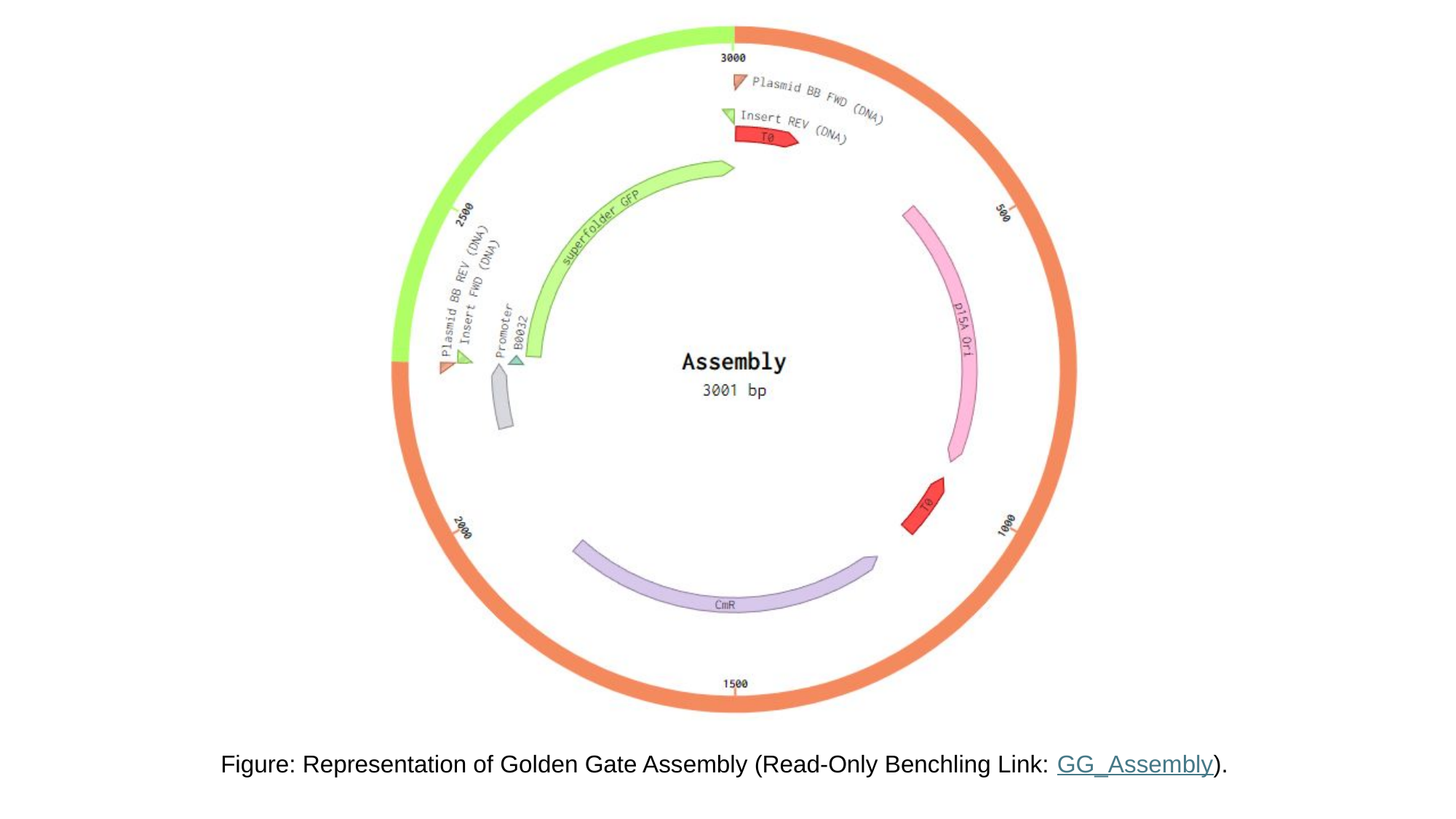

Figure: Representation of Golden Gate Assembly (Read-Only Benchling Link: GG_Assembly).

## Slide 8
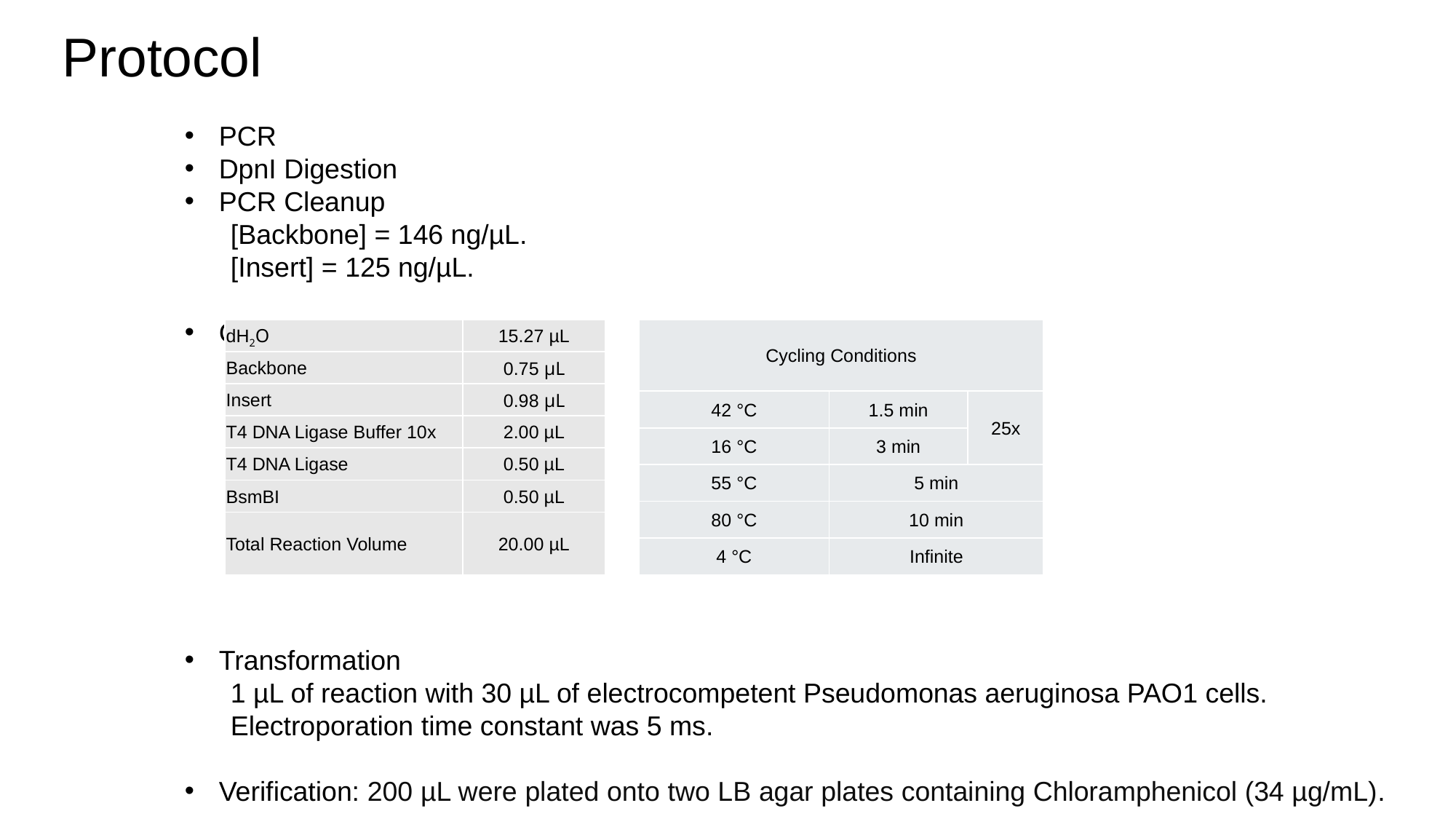

Protocol
PCR
DpnI Digestion
PCR Cleanup
 [Backbone] = 146 ng/µL.
 [Insert] = 125 ng/µL.
Golden Gate Assembly
Transformation
 1 µL of reaction with 30 µL of electrocompetent Pseudomonas aeruginosa PAO1 cells.
 Electroporation time constant was 5 ms.
Verification: 200 µL were plated onto two LB agar plates containing Chloramphenicol (34 µg/mL).
| Cycling Conditions | | |
| --- | --- | --- |
| 42 °C | 1.5 min | 25x |
| 16 °C | 3 min | |
| 55 °C | 5 min | |
| 80 °C | 10 min | |
| 4 °C | Infinite | |
| dH2O | 15.27 µL |
| --- | --- |
| Backbone | 0.75 µL |
| Insert | 0.98 µL |
| T4 DNA Ligase Buffer 10x | 2.00 µL |
| T4 DNA Ligase | 0.50 µL |
| BsmBI | 0.50 µL |
| Total Reaction Volume | 20.00 µL |

## Slide 9
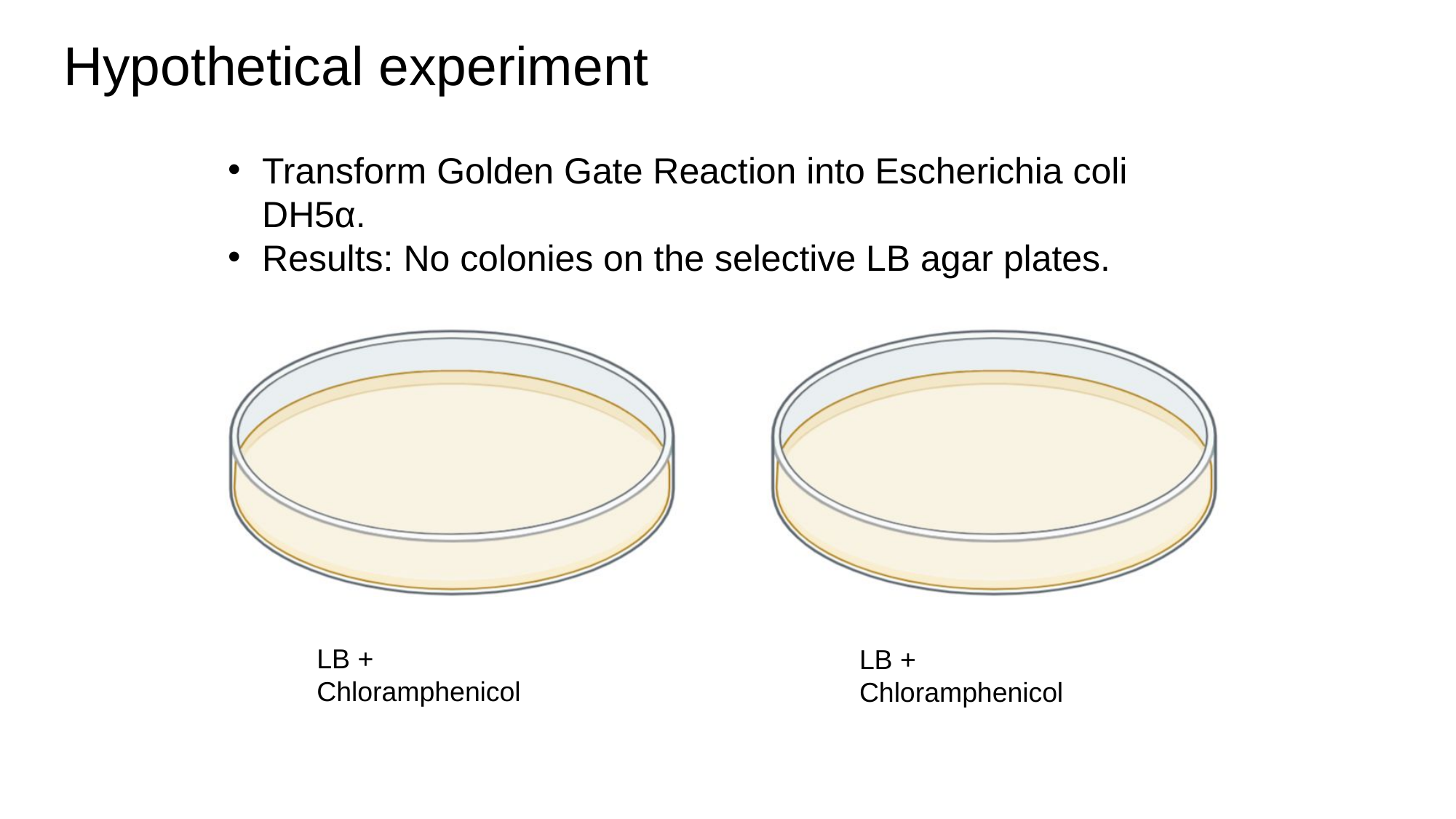

Hypothetical experiment
Transform Golden Gate Reaction into Escherichia coli DH5α.
Results: No colonies on the selective LB agar plates.
LB + Chloramphenicol
LB + Chloramphenicol

## Slide 10
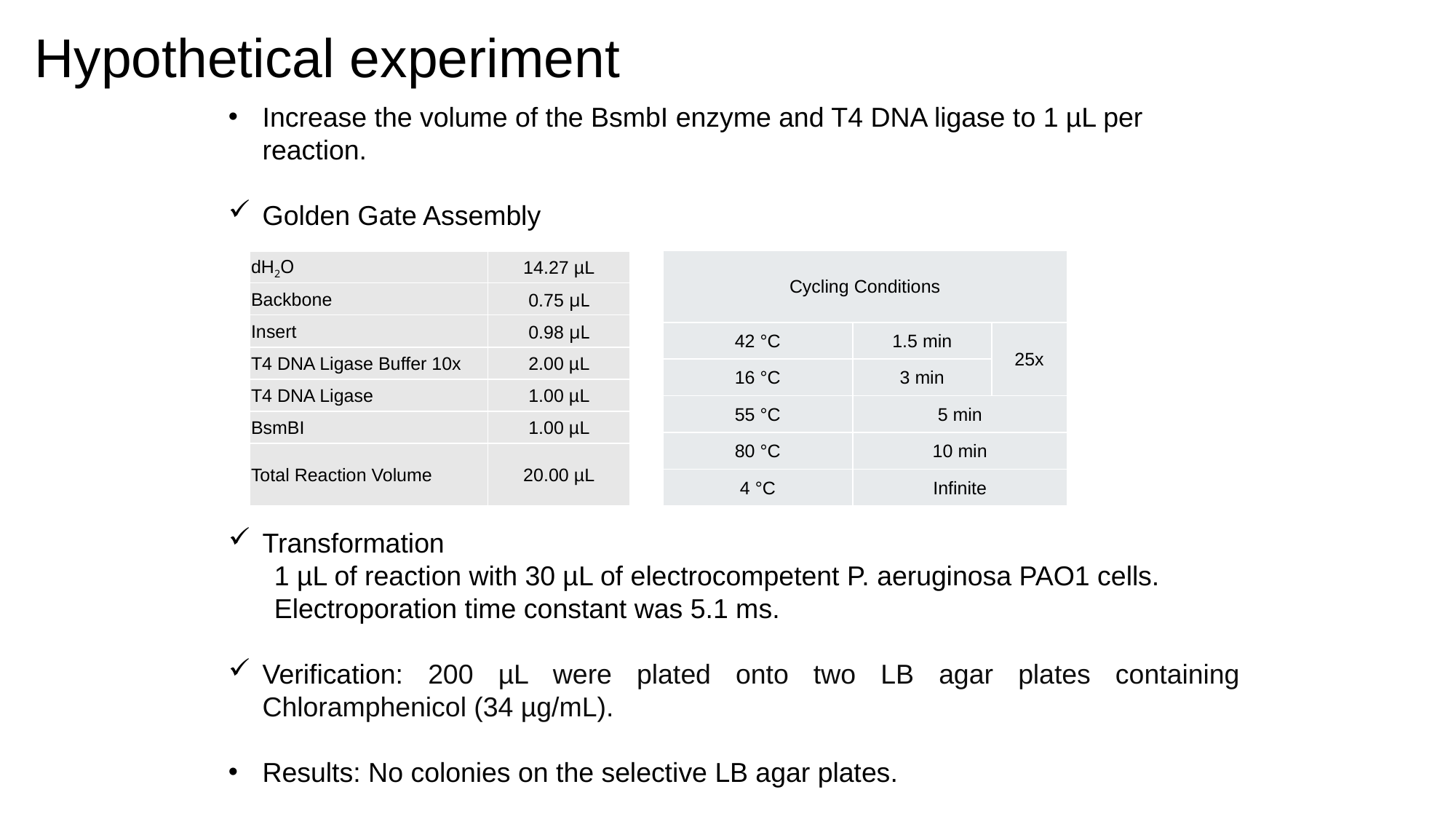

Hypothetical experiment
Increase the volume of the BsmbI enzyme and T4 DNA ligase to 1 µL per reaction.
Golden Gate Assembly
Transformation
 1 µL of reaction with 30 µL of electrocompetent P. aeruginosa PAO1 cells.
 Electroporation time constant was 5.1 ms.
Verification: 200 µL were plated onto two LB agar plates containing Chloramphenicol (34 µg/mL).
Results: No colonies on the selective LB agar plates.
| Cycling Conditions | | |
| --- | --- | --- |
| 42 °C | 1.5 min | 25x |
| 16 °C | 3 min | |
| 55 °C | 5 min | |
| 80 °C | 10 min | |
| 4 °C | Infinite | |
| dH2O | 14.27 µL |
| --- | --- |
| Backbone | 0.75 µL |
| Insert | 0.98 µL |
| T4 DNA Ligase Buffer 10x | 2.00 µL |
| T4 DNA Ligase | 1.00 µL |
| BsmBI | 1.00 µL |
| Total Reaction Volume | 20.00 µL |

## Slide 11
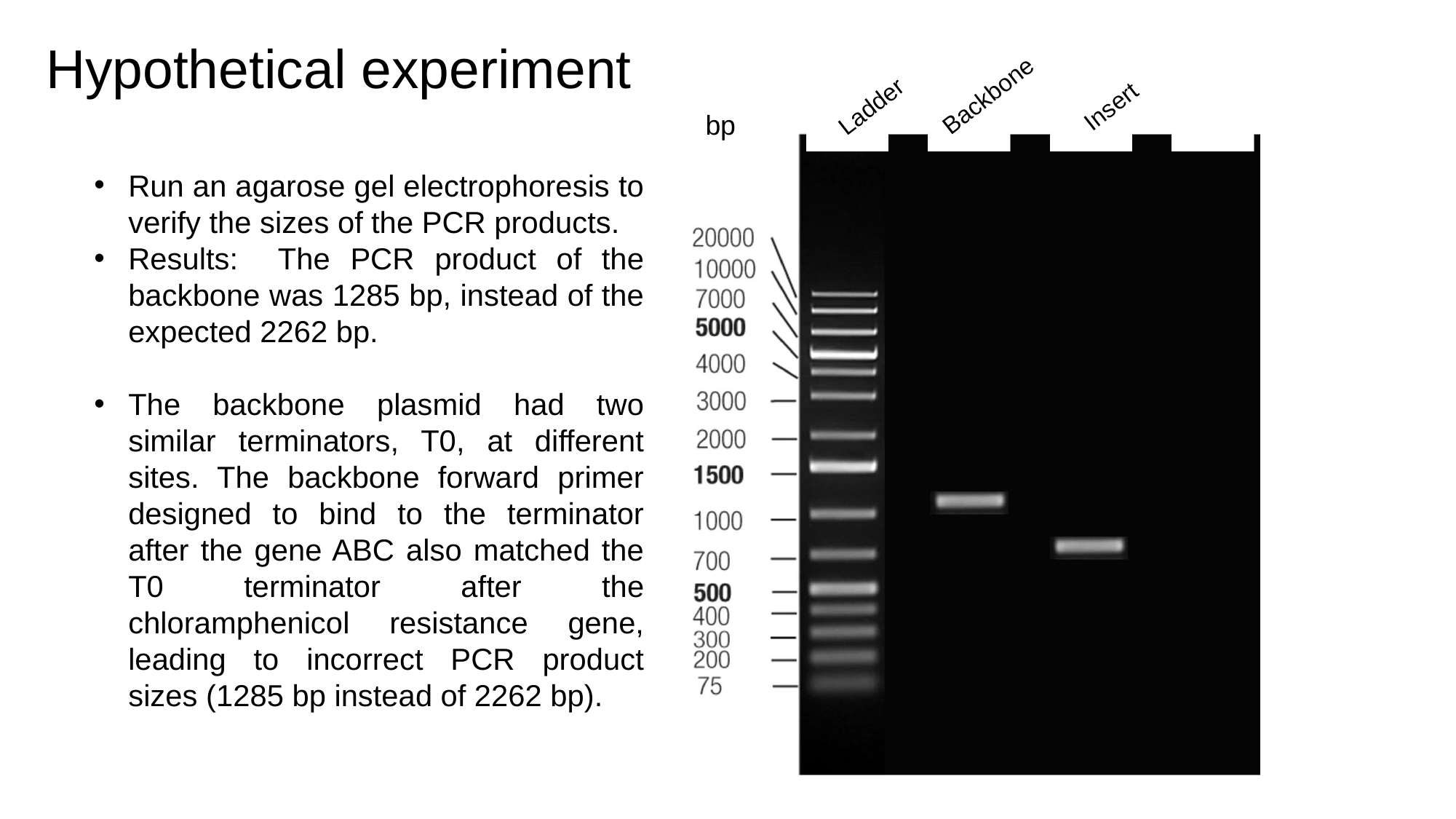

Hypothetical experiment
Backbone
Insert
Ladder
bp
Run an agarose gel electrophoresis to verify the sizes of the PCR products.
Results: The PCR product of the backbone was 1285 bp, instead of the expected 2262 bp.
The backbone plasmid had two similar terminators, T0, at different sites. The backbone forward primer designed to bind to the terminator after the gene ABC also matched the T0 terminator after the chloramphenicol resistance gene, leading to incorrect PCR product sizes (1285 bp instead of 2262 bp).

## Slide 12
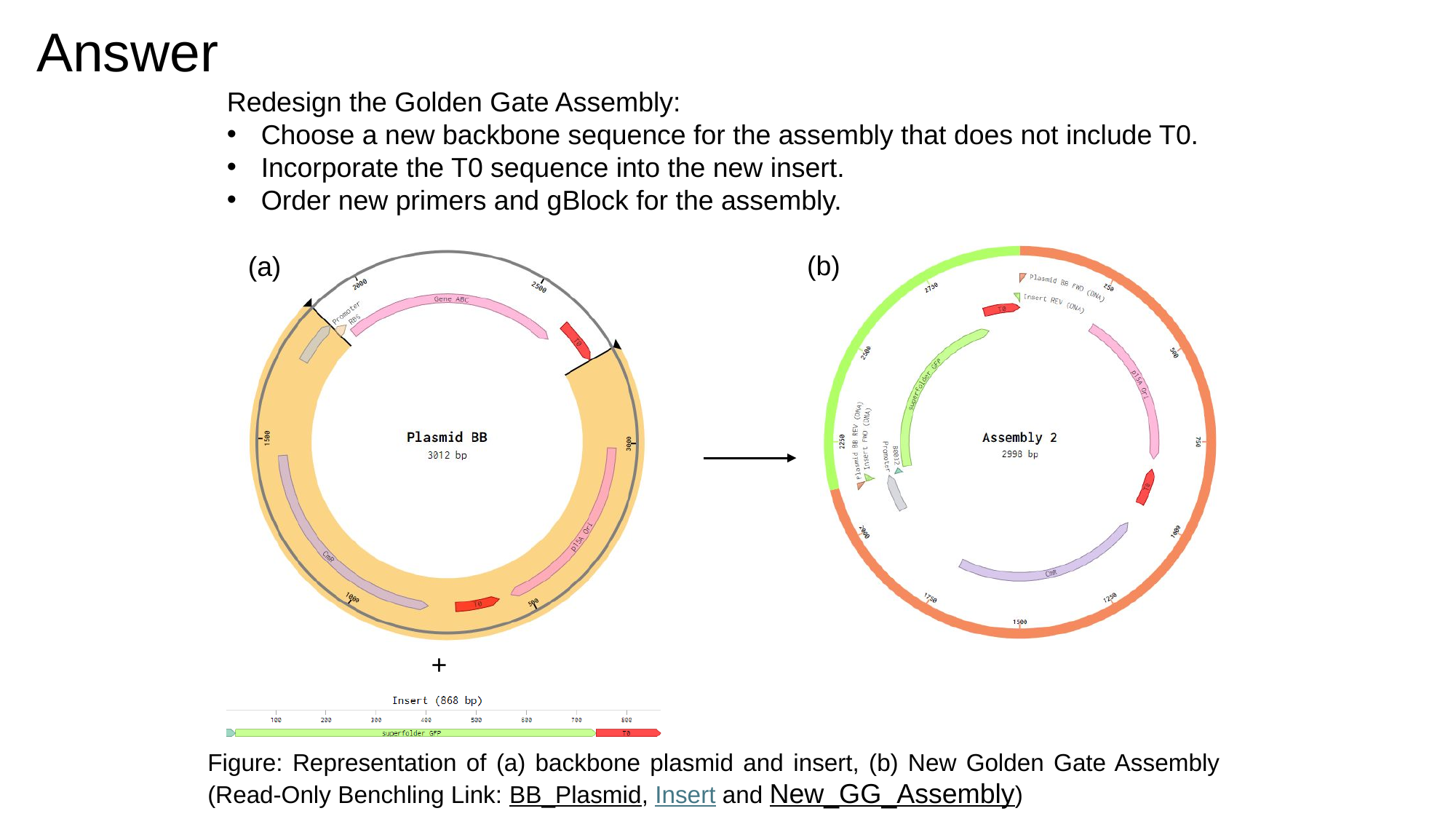

Answer
Redesign the Golden Gate Assembly:
Choose a new backbone sequence for the assembly that does not include T0.
Incorporate the T0 sequence into the new insert.
Order new primers and gBlock for the assembly.
(b)
(a)
+
Figure: Representation of (a) backbone plasmid and insert, (b) New Golden Gate Assembly (Read-Only Benchling Link: BB_Plasmid, Insert and New_GG_Assembly)
