# Supplementary material for: Teaching troubleshooting skills to graduate students
Source: eLife. 2024 Sep 17;13:e100761. doi: 10.7554/eLife.100761 (PMC11407763; doi:10.7554/eLife.100761)
Supplement: Supplementary file 1. — For each scenario there is a Word file that contains the following: background information; a description of the scenario; the protocol for the experiment that produced the unexpected result; the results of the experiment; information on the source of the error; background information that can be used to answer questions; and references. There is also a PowerPoint file for each scenario that contains example slides that can be used in real meetings. There are also templates for the Word and PowerPoint files. [file elife-100761-supp1.zip › Final Scenarios/Example2.docx]

**Membrane Surface Charge**

Keywords: synthetic biology, bioelectronics, imaging, electrochemical devices

**Background:**

Fluorescent membrane potential indicators provide a non-invasive *in vivo* single-cell level assay for cell membrane hyperpolarization. Thioflavin T (ThT) has been used as the membrane potential probe with bacteria cells^1^. The positively charged ThT molecules accumulate on the negatively charged (hyperpolarized) cell membrane. Thus, the fluorescence intensity is inversely correlated with the cell membrane potential.

Extracellular electron transfer (EET) is employed in anaerobic respirations of electroactive bacteria. The EET involves the transfer of electrons from the inner metabolic pathways of the cell to the outside electron acceptors such as metal oxides. *Shewanella oneidensis* MR-1 is a facultative anaerobic microorganism, which means it can use both oxygen (aerobic) and other electron acceptors (anaerobic) for respiration.

In one work, the ThT was used to examine the EET activity of S. oneidensis MR-1 in electrochemical cells^2^. They showed a positive correlation between cellular EET activity and fluorescence intensity. When the electrode was biased at positive potentials that energetically favor the electron transfer from cells to the electrode, increased fluorescence intensity from the cells was observed.

Resazurin (RZ) is commonly used to visualize oxygen leakage. Particularly, the *S. oneidensis* MR-1 would reduce the RZ to dihydroresorufin (colorless). And the dihydroresorufin would be oxidized to resorufin (pink) when contacted with oxygen. The RZ is widely used in cytotoxicity assay, thus, it should be compatible with *S. oneidensis* MR-1.

**Scenario:**

You would like to use the ThT with your electrochemical (EC) device to observe membrane potential under different electrode bias voltages. As shown in Figure 1, the EC device contains one Au working electrode (WE) and one counter electrode (CE), fabricated on the microscope slide. Since the reference electrodes (REs) for the EC device were not compatible with the fluorescent microscope setup, you can only control the voltage between electrodes, but cannot measure the electrode potentials accurately. Normally electrode voltages are kept < 0.5 V between the two electrodes. The initial step is to apply a bias voltage between two electrodes and verify that the ThT fluorescence intensity is higher on the positive electrode, compared to the negative electrode.


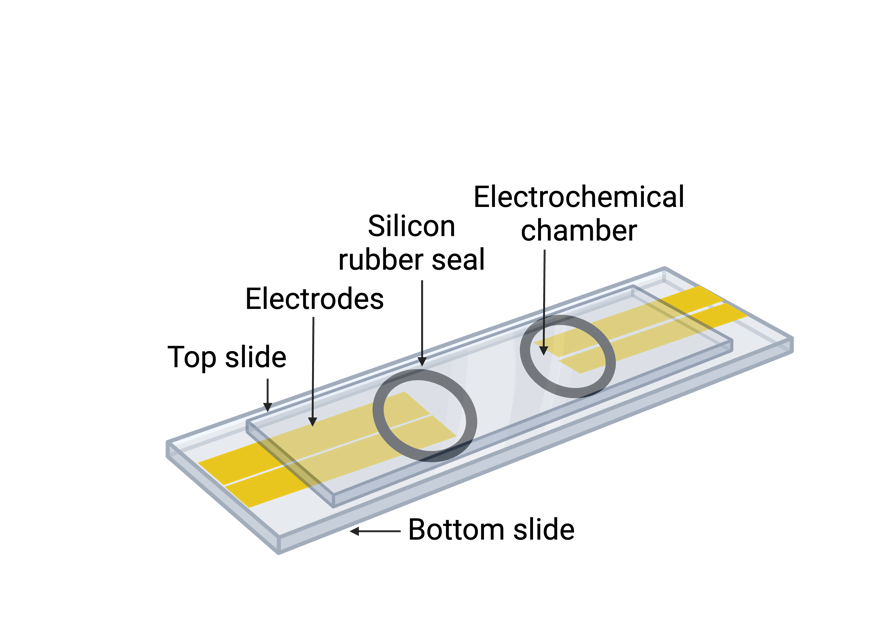


**Figure 1.** Proposed electrochemical cell device.

**Experiment design:**

You would like to use a fluorescence microscope to observe the EC device inoculated with *S. oneidensis* MR-1 and ThT. The EC device can be connected with a potentiostat (to change the electrode voltages) while on the microscope stage. Since there is no environment chamber for the microscope stage, you would need to seal the EC device to achieve an anaerobic environment using silicon rubber as the seal.

**Set up details:**

1. The EC devices with two Au electrodes are used.
2. No reference electrode is used.
3. Electrolyte is a common medium used to support cell growth in a minimal media supplemented with lactate.
4. ThT concentration is 10 μM.
5. *S. oneidensis* MR-1 is grown aerobically overnight to reach the stationary growth phase.
6. Grown cell cultures are 3X washed, and have the cell density measured with OD_600_ (usually around 2), before transferred into the anaerobic chamber.
7. EC devices are inoculated inside the anaerobic chamber with *S. oneidensis* MR-1 at OD_600_ of 0.1 to 0.5.
8. EC devices are sealed air-tight with silicon rubber gaskets, in the anaerobic chamber.
9. Then, without waiting, the EC devices are brought to the microscope room for examination (ambient environment). The examination could last up to 5 hours.
10. Negative controls are the abiotic electrolyte.
11. Brightness, contrast, exposure time, and laser aperture size are kept the same to ensure comparability of the results.

**Example graphs and experimental outcomes:**

The initial experiment showed expected fluorescence intensity change correlated with electrode bias. As shown in Figure 2a, when the gate was biased at 0.5 V against the source (V_GS_ =0.5 V), the fluorescence intensity differences were observed 6 minutes after applied voltages. Subsequently, 6 minutes after switching V_GS_ to -0.5 V, higher fluorescence intensity over the source was observed compared to the gate (Figure 2b).

However, in other separate experiments, the fluorescence intensity did not change regardless of the applied electrode voltage (Figure 3).

All negative controls (abiotic samples with ThT) did not show fluorescence.

**Graphs and supporting graphics:**


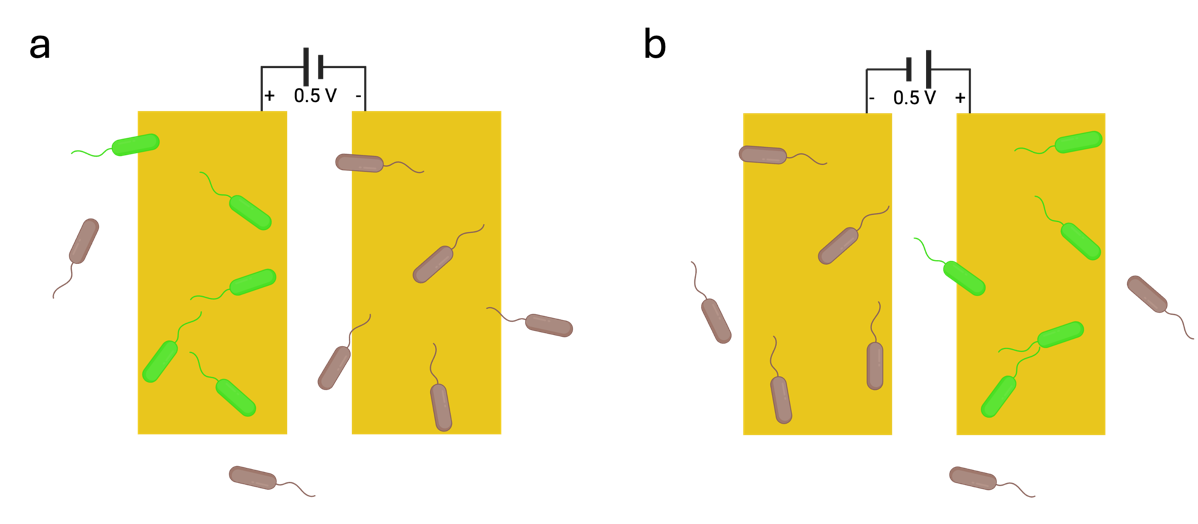


**Figure 2.** The expected fluorescence intensity showed changes with electrode voltages. (a) 6 minutes after biasing at 0.5V. (b) 6 minutes after biasing at -0.5 V.


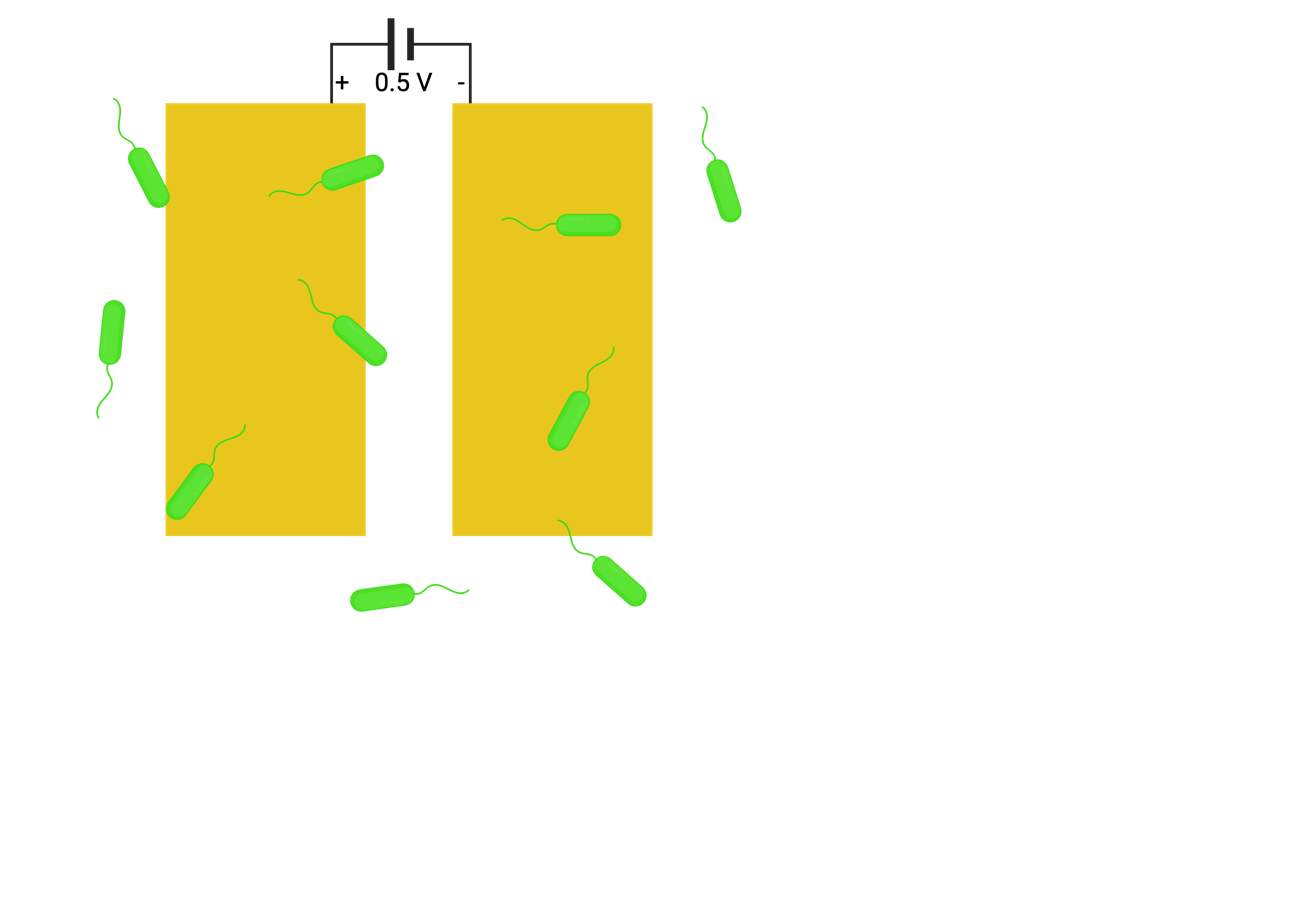


**Figure 3.** Uniform fluorescence intensity observed on devices when the electrode potential could not induce the intensity change.

**Source of error:**

Dissolved oxygen could cause cell membrane hyperpolarization, leading to isotropic fluorescence in the cell culture. Oxygen leakage could also explain the higher-than-expected cell number observed with the cell band, as cells grow faster during aerobic conditions through the hours-long experiment.

Using oxygen indicators such as resazurin (RZ), we could visualize the oxygen leakage from the seal. S. oneidensis MR-1 would reduce the RZ to dihydroresorufin (colorless). And the dihydroresorufin would be oxidized to resorufin (pink) when contacted with oxygen. As shown in Figure 4, when the EC chambers are supplemented with RZ and exposed to oxygen in the ambient environment, the oxygen diffusion could be visualized by the pink color. Since *Shewanella oneidensis* MR-1 is a facultative anaerobic microorganism, the cells near the seal would switch to aerobic respiration, consuming the permeated oxygen. Consequently, at higher cell density the core of the chamber could remain anaerobic, leading to inconsistent results.

**Table 1.** Additional information known by the leader that can be provided upon request

| **Meeting Notes for the Leader**  Not to be shared with the group | |
| --- | --- |
| Other researcher’s experiments | - Other lab members are performing cloning work. - One previous work used the same silicon rubber seal for electrochemical devices. Their results showed comparable results for sealed devices in ambient conditions and devices in the anaerobic chamber. However, the reductive activities from bacterial EET were slightly less for ambient sealed devices. This was attributed to differences in experiment conditions such as longer waiting time between device preparation and sampling and slight oxygen leakage from the seals. |
| Storage information | - The media stocks are purged with argon for 15 mins and stored in the anaerobic chamber. - Medium supplements such as lactate, antibiotics are not purged and stored in the anaerobic chamber. |
| Device information | - Devices are used for other electrochemical experiments in the anaerobic chamber without issues. |
| Source of error | - Dissolved oxygen could cause cell membrane hyperpolarization, leading to isotropic fluorescence in the cell culture. |
| Hints for group | - Since *S. oneidensis* is facultatively anaerobic, their respiratory pathway could switch between the aerobic and anaerobic ones. Thus, they may need time to switch from aerobic growth to anaerobic EET. - The *S. oneidensis* could ‘self-purge’ the medium by consuming the dissolved oxygen with aerobic respiration and then switching on their anaerobic pathways afterward. |

**References:**

1 Prindle, A. *et al.* Ion channels enable electrical communication in bacterial communities. *Nature* **527**, 59-63 (2015). <https://doi.org/10.1038/nature15709>

2 Pirbadian, S., Chavez, M. S. & El-Naggar, M. Y. Spatiotemporal mapping of bacterial membrane potential responses to extracellular electron transfer. *Proceedings of the National Academy of Sciences of the United States of America* **117**, 20171-20179 (2020). <https://doi.org/10.1073/pnas.2000802117>
