# Supplementary material for: Teaching troubleshooting skills to graduate students
Source: eLife. 2024 Sep 17;13:e100761. doi: 10.7554/eLife.100761 (PMC11407763; doi:10.7554/eLife.100761)
Supplement: Supplementary file 1. — For each scenario there is a Word file that contains the following: background information; a description of the scenario; the protocol for the experiment that produced the unexpected result; the results of the experiment; information on the source of the error; background information that can be used to answer questions; and references. There is also a PowerPoint file for each scenario that contains example slides that can be used in real meetings. There are also templates for the Word and PowerPoint files. [file elife-100761-supp1.zip › Final Scenarios/Example8.pptx]

## Slide 1
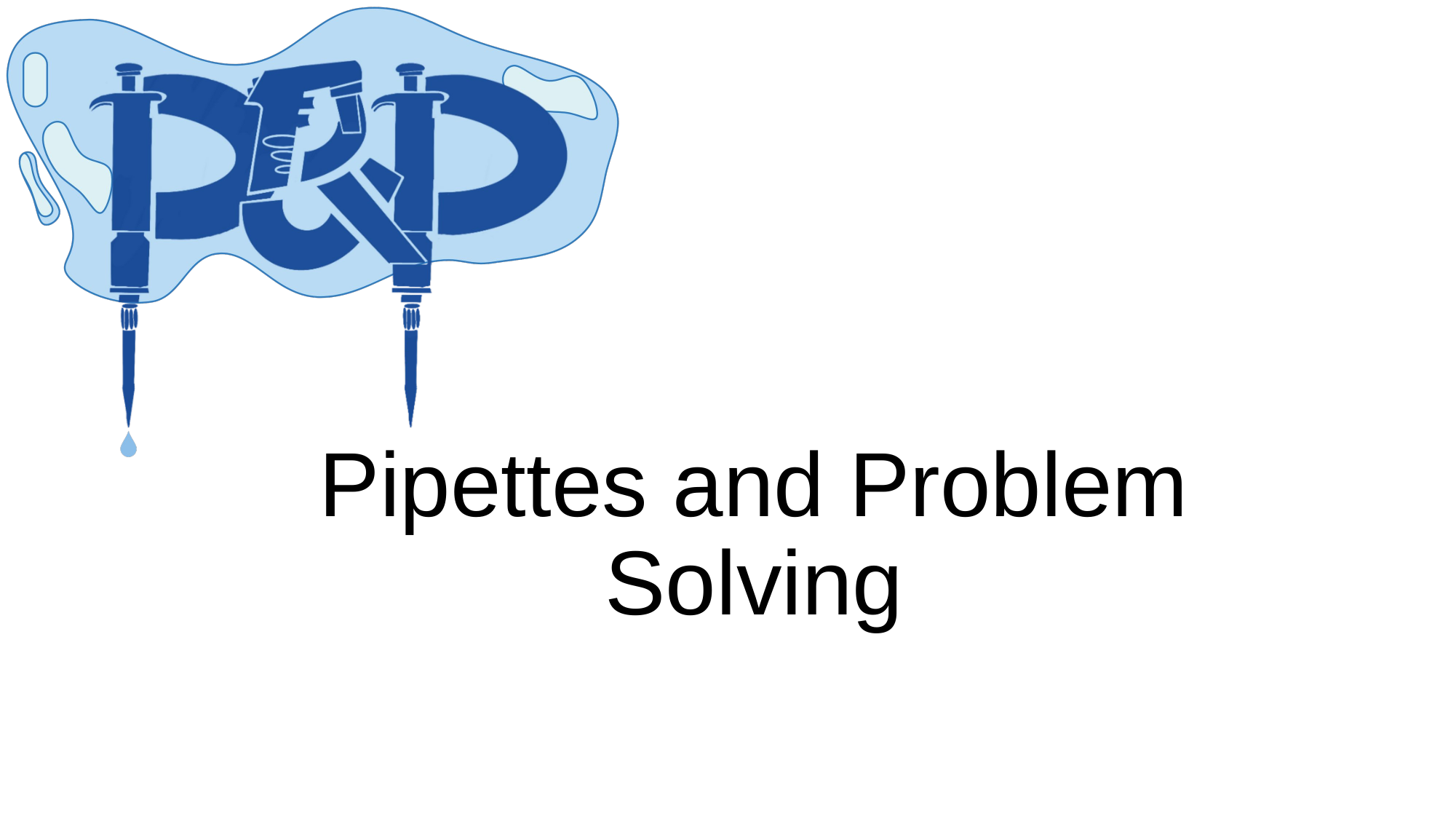

# Pipettes and Problem Solving

## Slide 2
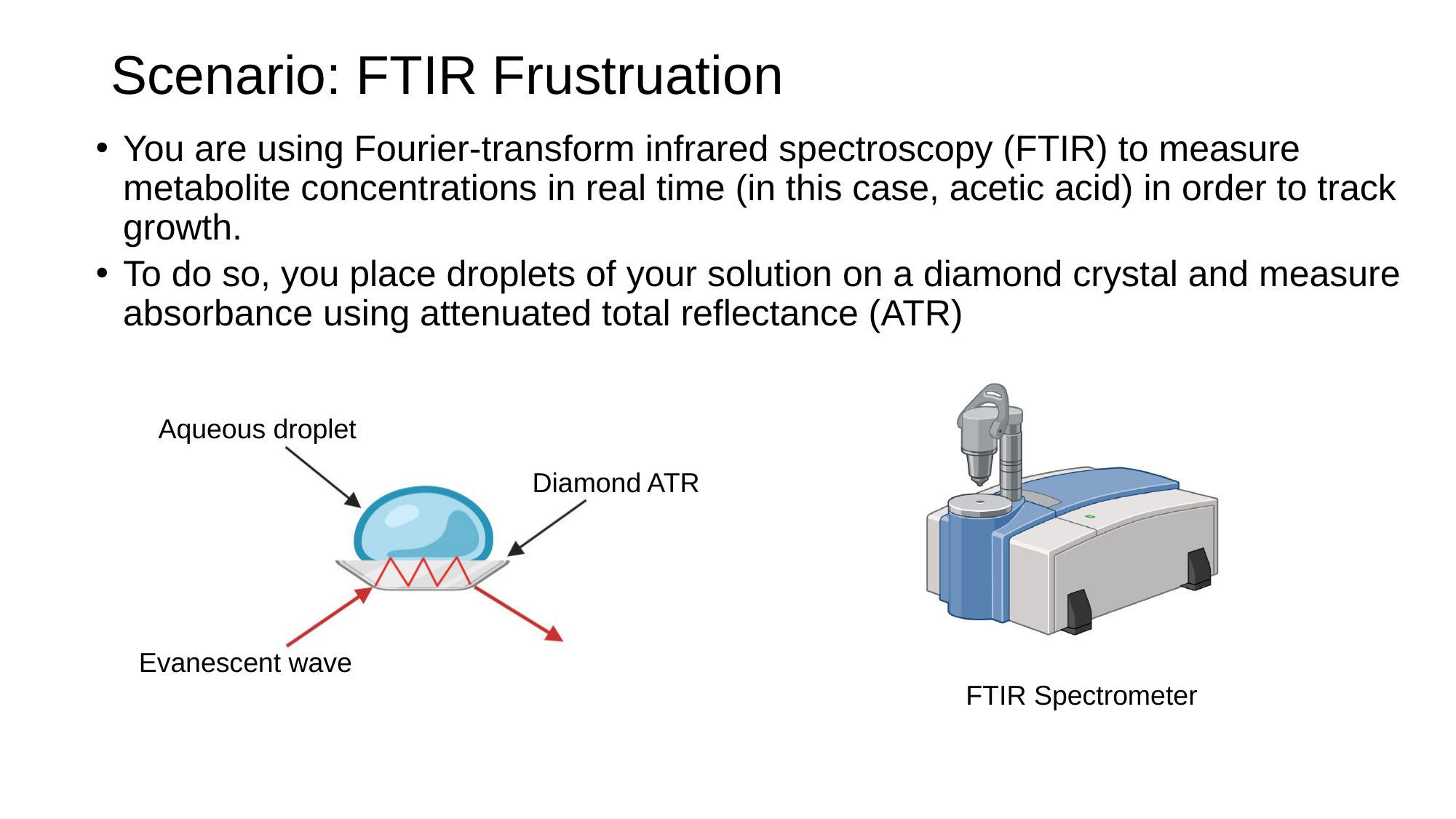

# Scenario: FTIR Frustruation
You are using Fourier-transform infrared spectroscopy (FTIR) to measure metabolite concentrations in real time (in this case, acetic acid) in order to track growth.
To do so, you place droplets of your solution on a diamond crystal and measure absorbance using attenuated total reflectance (ATR)
Aqueous droplet
Diamond ATR
Evanescent wave
FTIR Spectrometer

## Slide 3
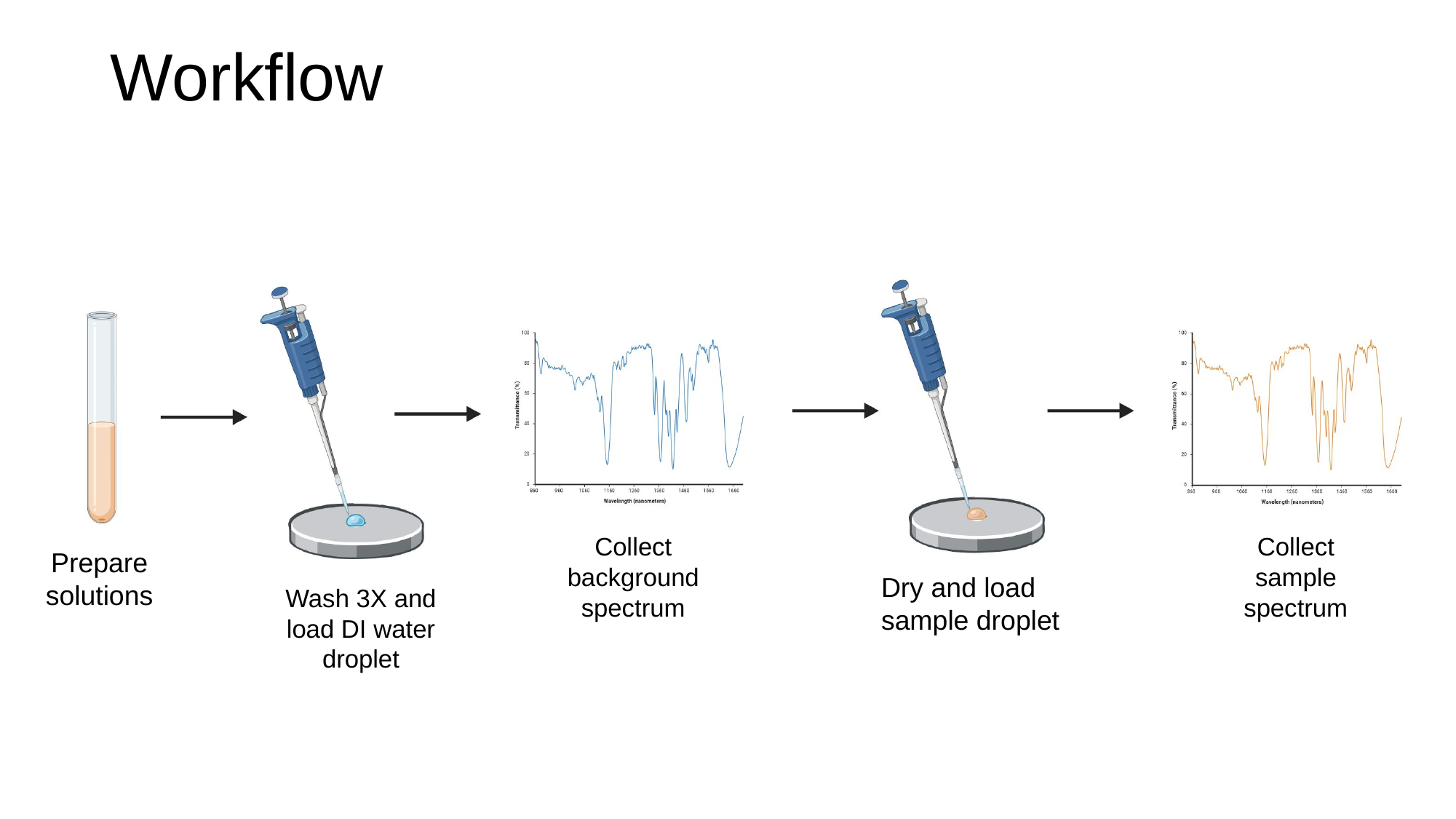

# Workflow
Collect sample spectrum
Collect background spectrum
Prepare solutions
Dry and load sample droplet
Wash 3X and load DI water droplet

## Slide 4
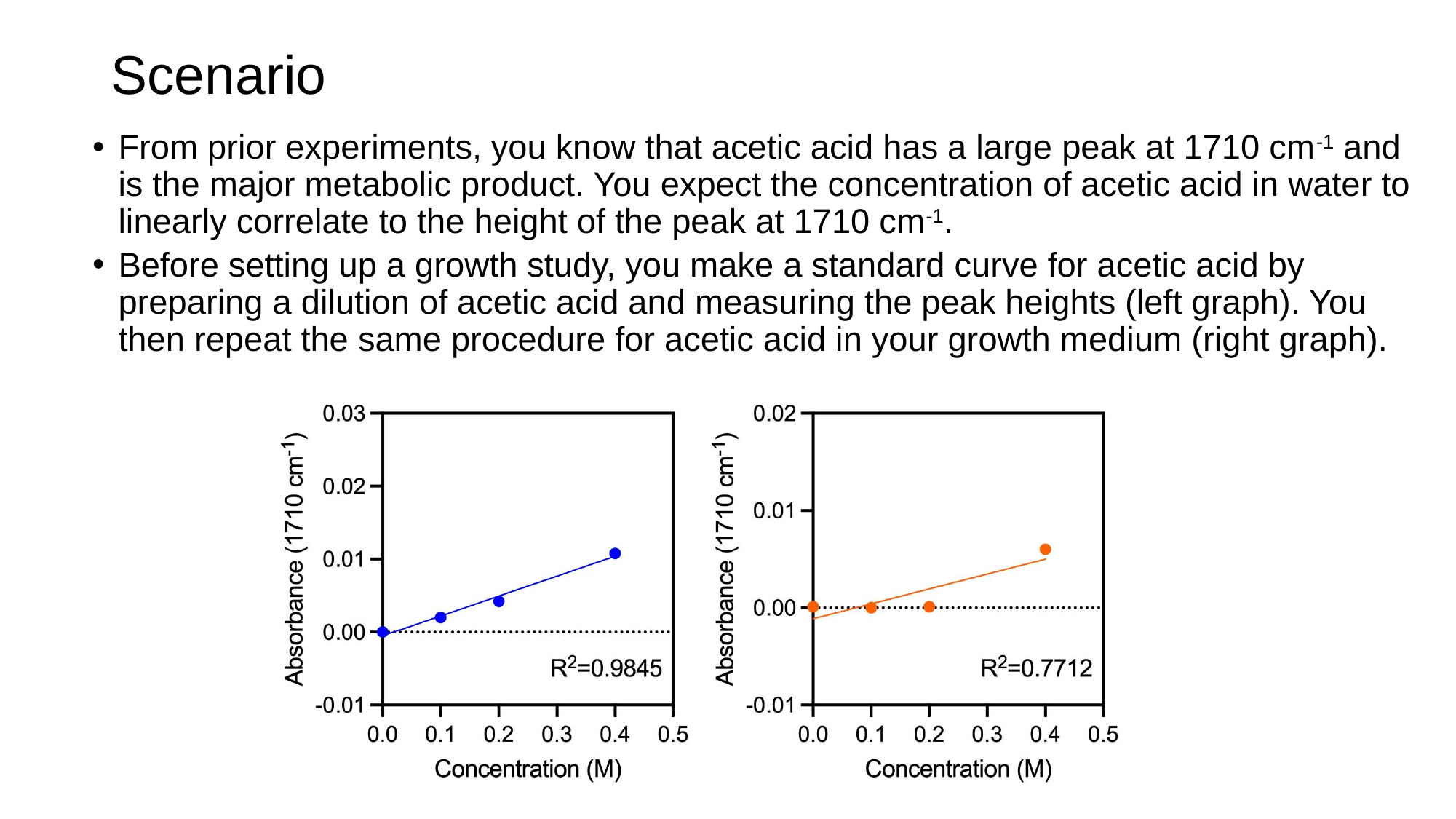

# Scenario
From prior experiments, you know that acetic acid has a large peak at 1710 cm-1 and is the major metabolic product. You expect the concentration of acetic acid in water to linearly correlate to the height of the peak at 1710 cm-1.
Before setting up a growth study, you make a standard curve for acetic acid by preparing a dilution of acetic acid and measuring the peak heights (left graph). You then repeat the same procedure for acetic acid in your growth medium (right graph).

## Slide 5
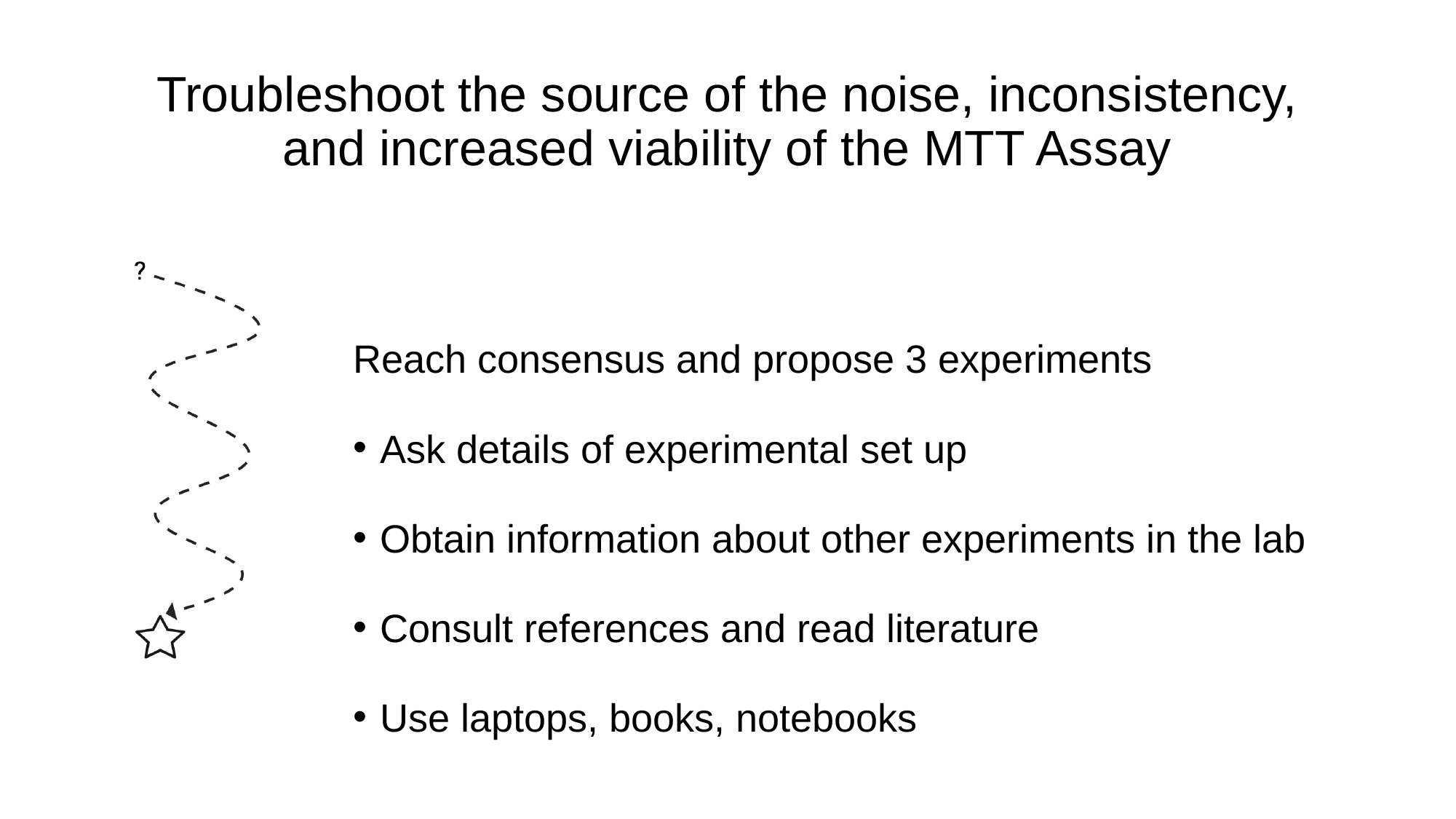

# Troubleshoot the source of the noise, inconsistency, and increased viability of the MTT Assay
Reach consensus and propose 3 experiments
Ask details of experimental set up
Obtain information about other experiments in the lab
Consult references and read literature
Use laptops, books, notebooks

## Slide 6
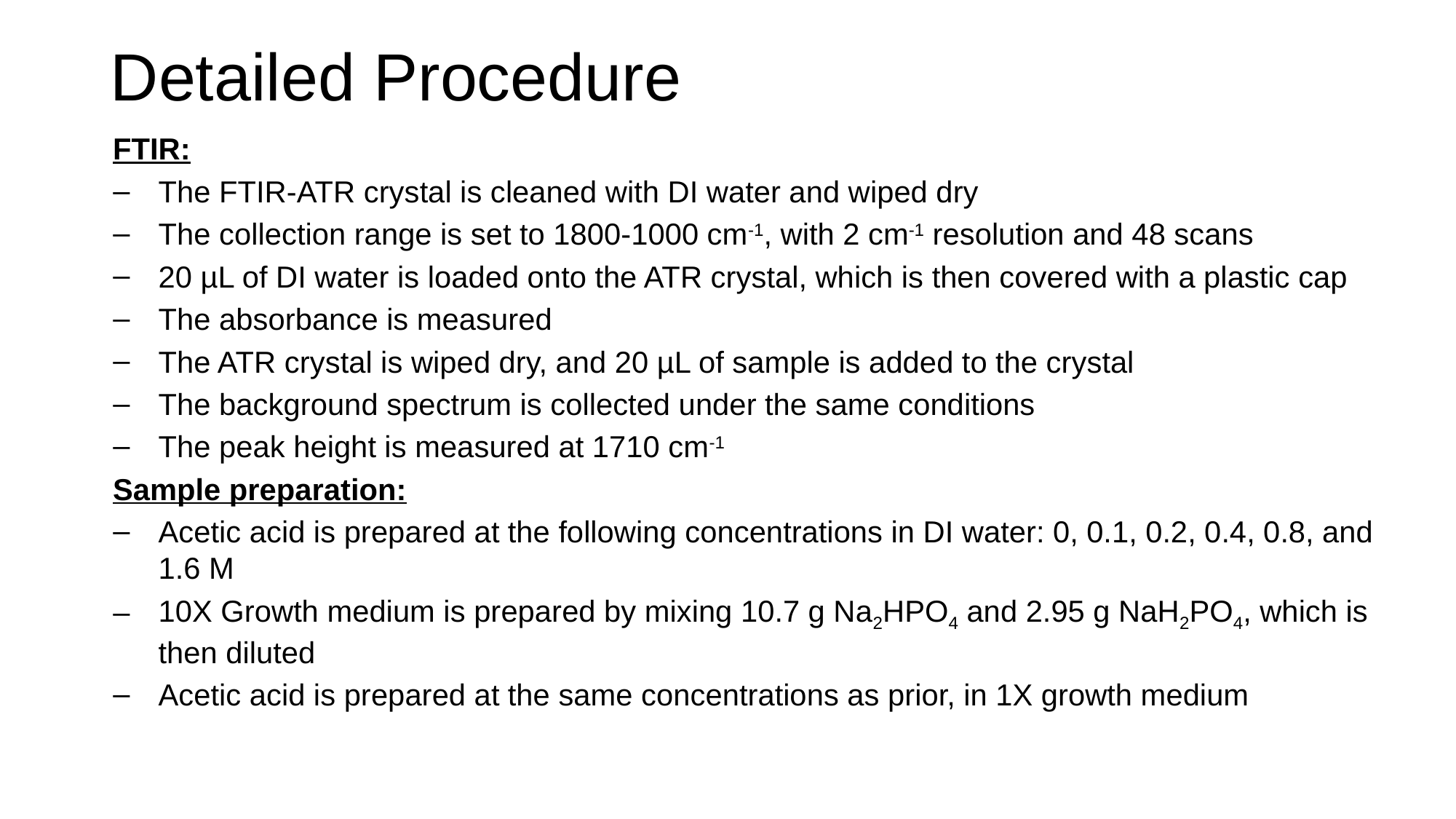

# Detailed Procedure
FTIR:
The FTIR-ATR crystal is cleaned with DI water and wiped dry
The collection range is set to 1800-1000 cm-1, with 2 cm-1 resolution and 48 scans
20 µL of DI water is loaded onto the ATR crystal, which is then covered with a plastic cap
The absorbance is measured
The ATR crystal is wiped dry, and 20 µL of sample is added to the crystal
The background spectrum is collected under the same conditions
The peak height is measured at 1710 cm-1
Sample preparation:
Acetic acid is prepared at the following concentrations in DI water: 0, 0.1, 0.2, 0.4, 0.8, and 1.6 M
10X Growth medium is prepared by mixing 10.7 g Na2HPO4 and 2.95 g NaH2PO4, which is then diluted
Acetic acid is prepared at the same concentrations as prior, in 1X growth medium

## Slide 7
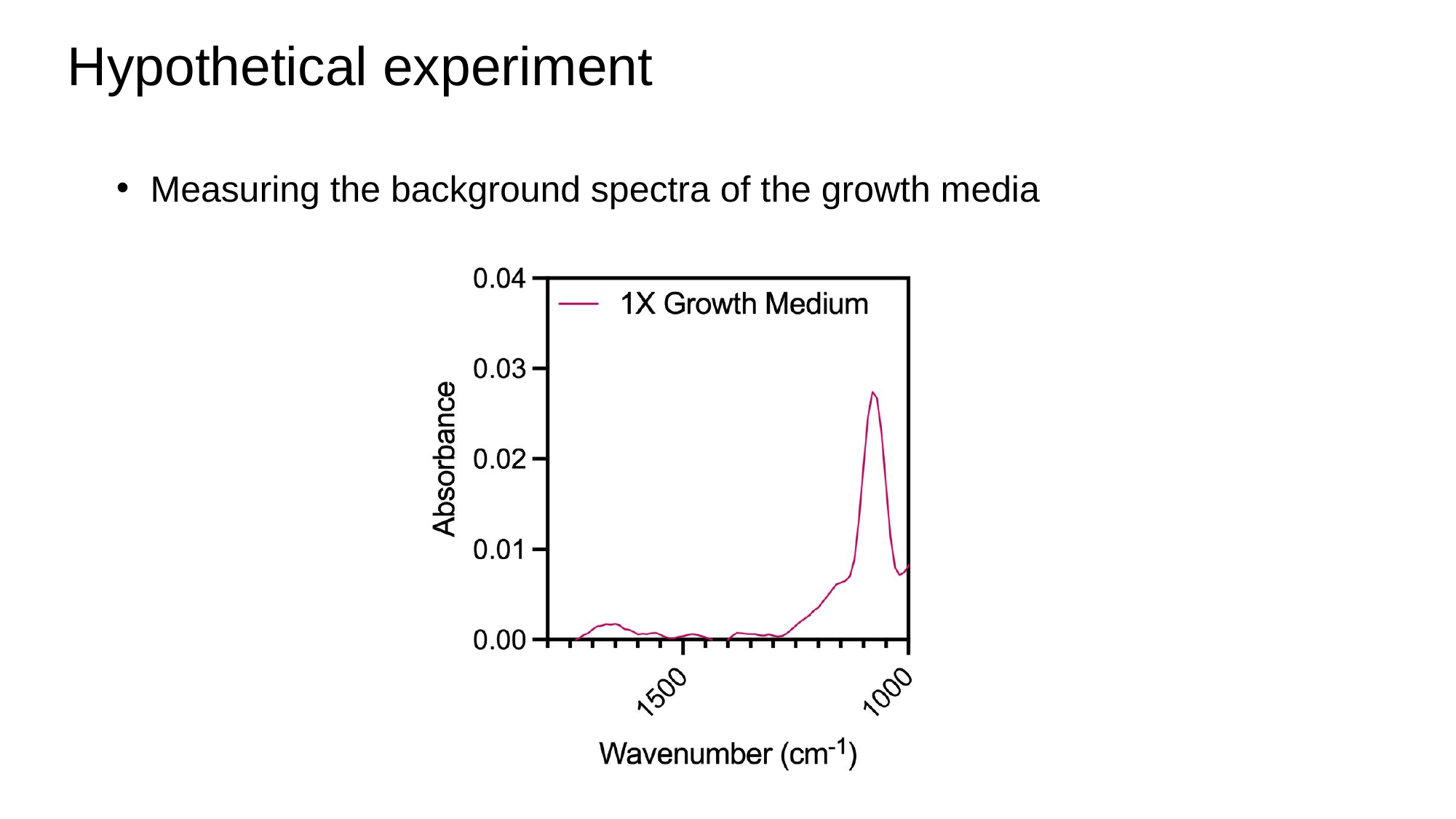

# Hypothetical experiment
Measuring the background spectra of the growth media

## Slide 8
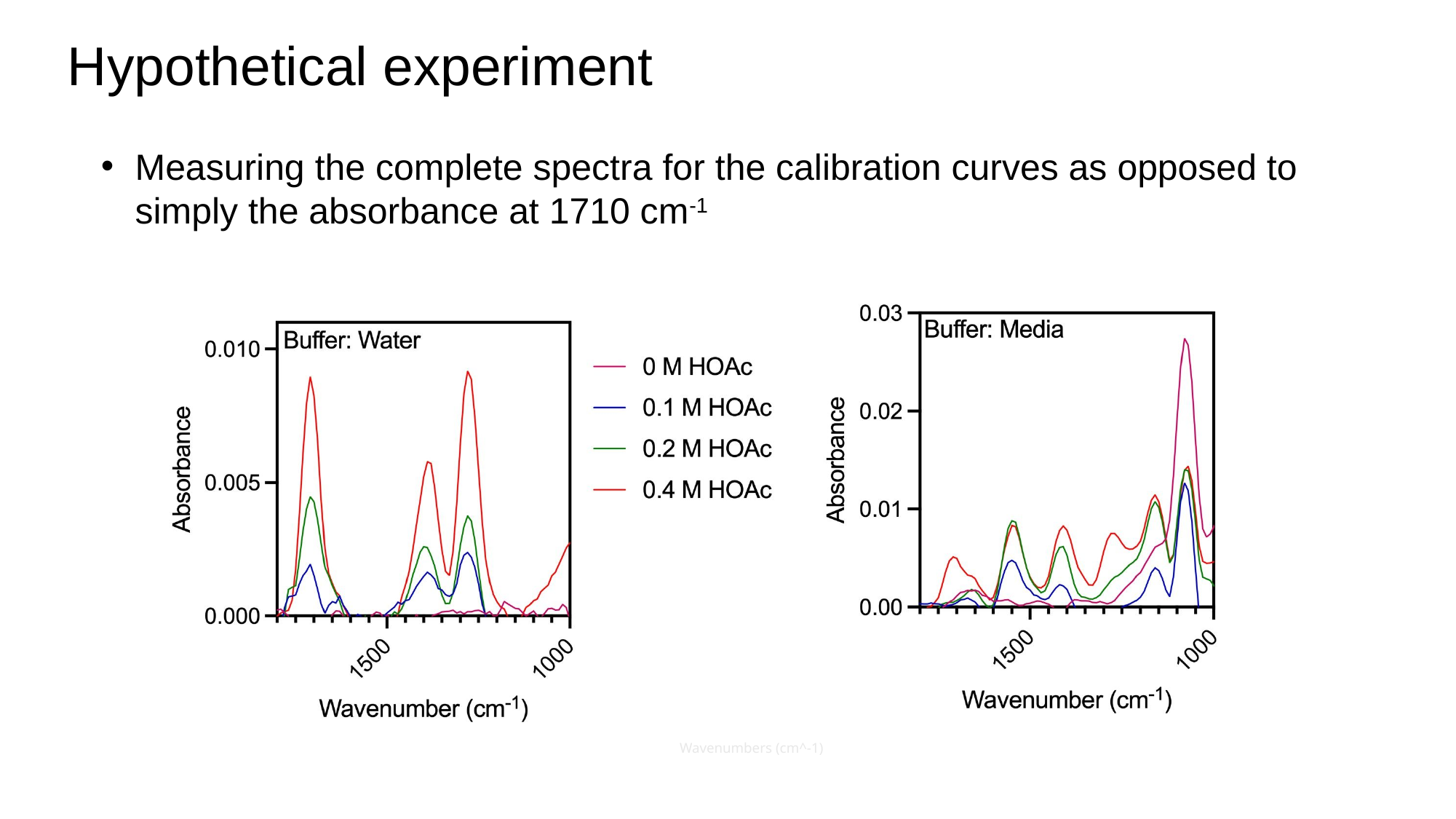

Hypothetical experiment
Measuring the complete spectra for the calibration curves as opposed to simply the absorbance at 1710 cm-1
Wavenumbers (cm^-1)

## Slide 9
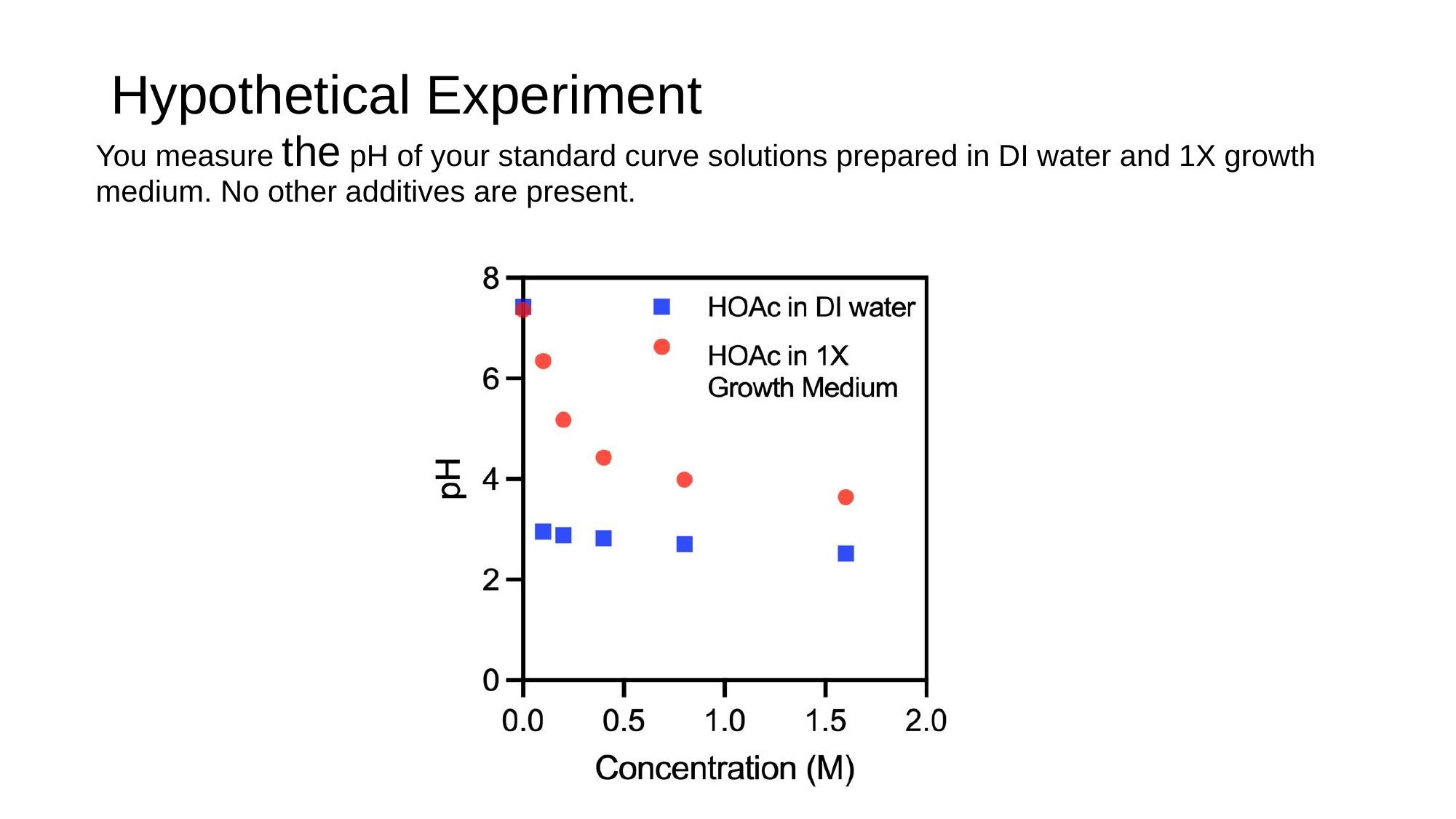

# Hypothetical Experiment
You measure the pH of your standard curve solutions prepared in DI water and 1X growth medium. No other additives are present.

## Slide 10
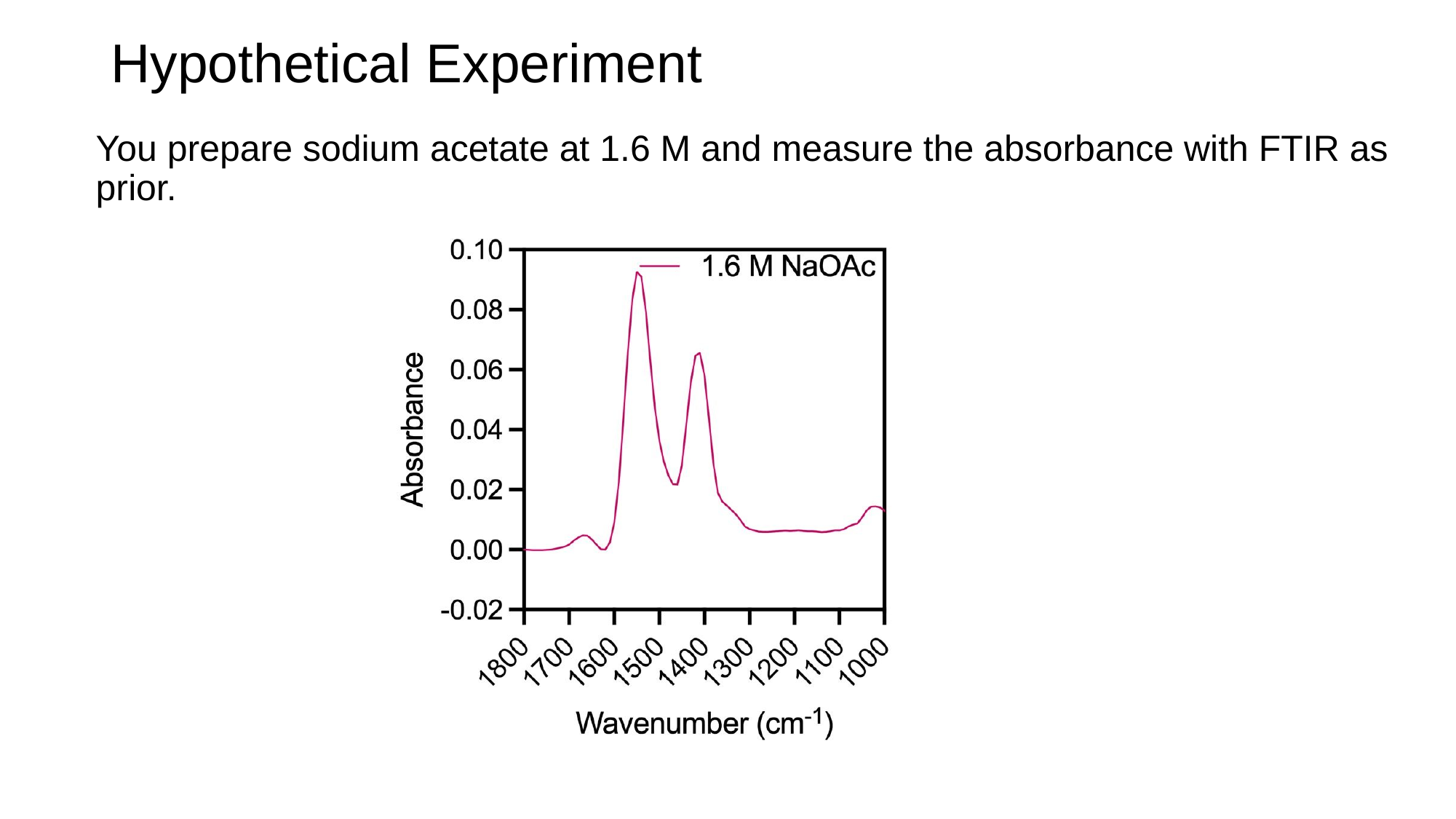

# Hypothetical Experiment
You prepare sodium acetate at 1.6 M and measure the absorbance with FTIR as prior.

## Slide 11
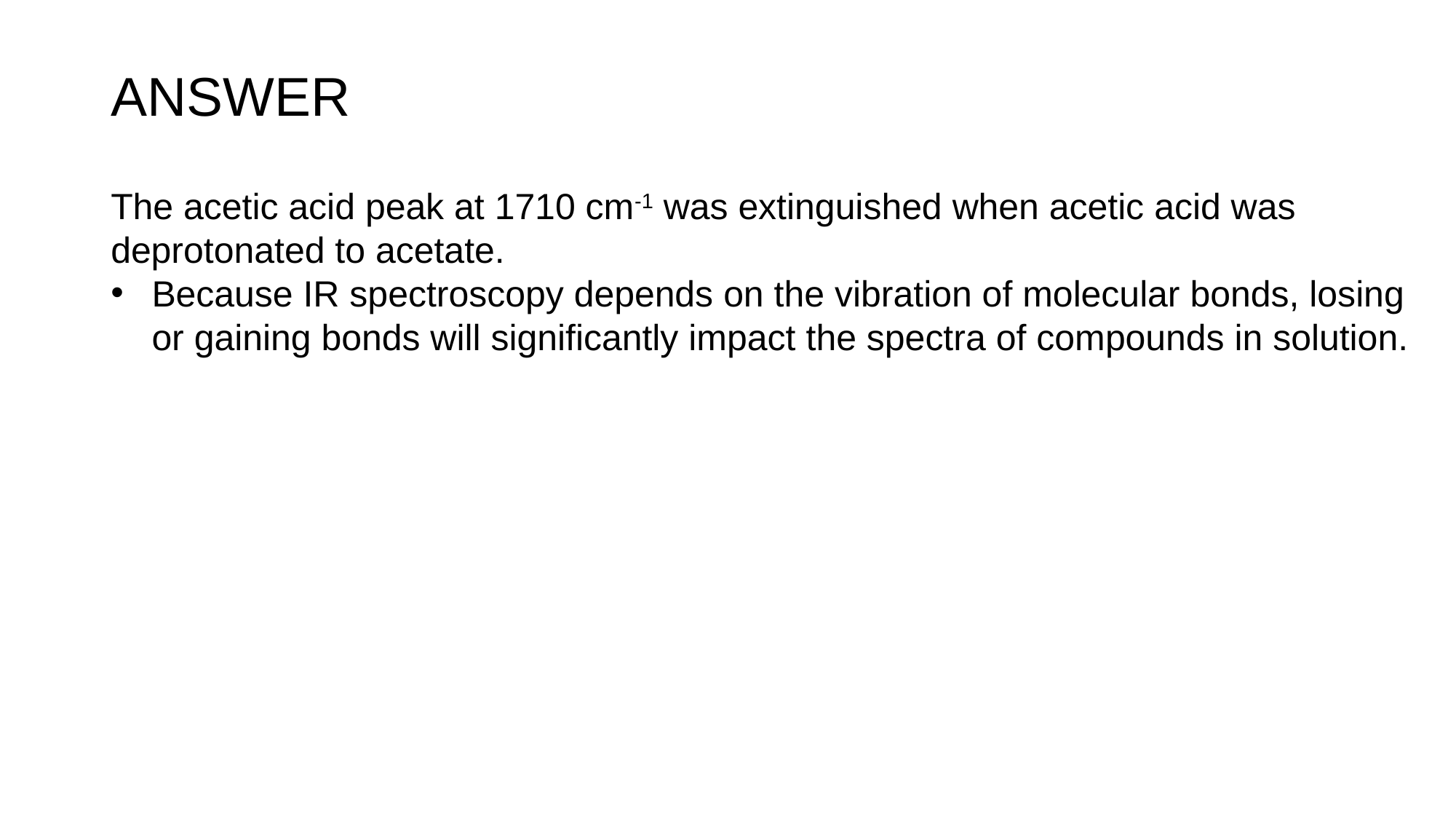

# ANSWER
The acetic acid peak at 1710 cm-1 was extinguished when acetic acid was deprotonated to acetate.
Because IR spectroscopy depends on the vibration of molecular bonds, losing or gaining bonds will significantly impact the spectra of compounds in solution.
