# Supplementary material for: Teaching troubleshooting skills to graduate students
Source: eLife. 2024 Sep 17;13:e100761. doi: 10.7554/eLife.100761 (PMC11407763; doi:10.7554/eLife.100761)
Supplement: Supplementary file 1. — For each scenario there is a Word file that contains the following: background information; a description of the scenario; the protocol for the experiment that produced the unexpected result; the results of the experiment; information on the source of the error; background information that can be used to answer questions; and references. There is also a PowerPoint file for each scenario that contains example slides that can be used in real meetings. There are also templates for the Word and PowerPoint files. [file elife-100761-supp1.zip › Final Scenarios/Example3.docx]

**Streptavidin-conjugation assay**

Keywords: Antibody, molecular biology, biochemistry, immunology

**Background:**

Biotin is a cofactor or coenzyme that can also be coopted for molecular biology (*1*). Expression of a biotin carboxy carrier protein (BCCP) fused to the protein of interest allows the BCCP to be biotinylated by a biotin ligase, BirA (*1*). BirA functions by recognizing a specific lysine residue within the BCCP sequence. One of the shorter BCCP sequences is the 15 amino acid long AviTag (*2*). BirA kits are available commercially for biotinylation purposes (Avidity) (*3*).

Biotin covalently binds streptavidin (*4*). Streptavidin can be conjugated to a fluorophore or enzyme to amplify detection of biotinylated molecules (*5*). Biotinylation for streptavidin binding is possible with both purified protein and live cells (*6*).

**Scenario:**

You are a researcher interested in quantifying protein expression using fluorescently conjugated streptavidin as measured on a plate reader. To do this, you have decided to recombinantly express a modified version of your protein of interest, Pro2, containing an AviTag via an inducible plasmid under control of a LacI promoter induced by isopropyl ß-D-1-thiogalactopyranoside (IPTG). The protocol described below has been passed to you from a previous student in the lab who was quantifying a homologous protein, Pro1 using an AviTag. The previous student was using purified protein, and you are using whole cells; however, the biotin-streptavidin system has been used for both. You are obtaining plate reader fluorescent values within the noise regime.

**Previous Student’s Notes** (can be shared with players)

1. Thaw ~0.5 mL Pro2 on ice (from 1 mg/mL solution)
   1. Pro2 = 150 kDa MW
   2. 1 nmol = 150 µg Pro2 = 33 nmol in 0.5 mL (66 µM)
2. Buffer exchange into 10 mM Tris, pH 8, 20 mM NaCl
3. Using Avidity BirA kit (*1*, *7*):
   1. 33 nmol Pro2 x (2.5 µg BirA per 10 nmol protein substrate) = 8.325 µg BirA
   2. Dilute Pro2 to 40 µM
      1. 0.83 mL final reaction volume
   3. Final reaction
      1. 500 µL Pro2 at 66 µM
      2. 83 µL BiomixA (bicine buffer)
      3. 83 µL BiomixB (ATP, biotin, MgOAc)
      4. 8.325 µL BirA (1 mg/mL)
      5. 155.68 µL water
   4. Incubate 40 mins at room temperature
4. Use centrifugal filter with 100000 MWCO to filter out unbound BirA
5. Prepare and run western blot (protocol from Mahmood and Yang, 2012 (*9*))
   1. Incubate 50 µg Pro2 in 15 µL dd H2O and sample buffer at 100C for 15 mins
   2. Prepare 10% stacking/6% separating SDS gel
   3. Once gel is solidified, place in electrophorator and cover with buffer
   4. Load ladder and samples
   5. Run samples for about an hour
   6. Cut filter sheets to fit gel and polyvinylidene fluoride (PDVF) membrane to fit gel
   7. Wet sponge and filter paper in transfer buffer and PDVF membrane in methanol
   8. Remove gel from casting
   9. Create transfer stack:
      1. Sponge
      2. 3 filter papers
      3. Gel PDVF
      4. 3 filter papers
      5. Squeeze out extra liquid and air bubbles
   10. Transfer for 90 mins
   11. Block membrane with 5% skim milk in TBST 1 hr
   12. Add streptavidin in 5% bovine serum albumin and incubate overnight at 4C on shaker
   13. Wash membrane 5 with TBST
   14. Add anti-Strep-HRP in 5% skim milk and incubate 1 hr
   15. Wash with TBST
   16. Incubate membrane in ECL mix and visualize in dark room

**Protocol:**

1. Inoculate 5 mL Luria Broth (LB) cultures with *E. coli* cells harboring Pro2 gene on inducible plasmid and incubate at 37 °C.
2. After 4 hours, induce cultures with IPTG and lower the incubator temperature.
3. Allow the cells to grow overnight.
4. The next day, harvest the cells by centrifuging the cultures for 10 minutes at 6000 xg.
5. Resuspend the cell pellet in 0.5 mL LB at OD = 0.8 (as measured on plate reader) in microcentrifuge tubes.
6. Using Avidity BirA kit (*1*, *7*):
   1. Dilute cells using LB to 40 µM Pro2 based on previous OD vs Pro2 expression calibration curve.
   2. Final reaction:

| **Component** | **µL per reaction** | **Amount per reaction** |
| --- | --- | --- |
| Pro2 | 500 | 40 µM (33 nmol) |
| BioMixA | 83 |  |
| BioMixB | 83 |  |
| BirA | 8.325 | 8.325 µg |
| Sterile MilliQ water | 155.68 |  |

Table 1: Final BirA reaction components.

- 1. Incubate the reaction mixture for 40 minutes at room temperature.

1. To conjugate the fluorophore (*10*), wash biotinylated cells from step 6c with PBS 3 times, centrifuging at 6000 xg for 3 minutes.
2. After the final wash, resuspend the cells in 500 µL of PBS and add 50 µL of streptavidin-AF647 (diluted to 20 nm).
3. Incubate the cells and streptavidin for 1 hour at room temperature while shaking.
4. Wash the cells and streptavidin again in PBS 3 times at 6000 xg for 3 minutes.
5. After the final wash, resuspend the cells in 50 µL of PBS.
6. Visualize on plate reader using 650 nm excitation:
   1. 200 µL total per well
   2. 10 µL cells, 190 µL PBS
   3. 3 technical replicates per biological replicate
   4. Three blanks (PBS)

**Relevant resources and protocols:**

Avidity BirA

Below is a condensed version of the Avidity BirA Ligase Kit reaction conditions (*7*). The full SOP can be found at <https://www.avidity.com/commerce/product.asp?NUMBER=1>.

Conjugated Streptavidin

Below is a condensed version of the ThermoFisher AlexaFluor647-Streptavidin conjugate information (*8*). The full SOP can be found at <https://www.thermofisher.com/order/catalog/product/S21374>.

**Figure 2.** Example relationship between OD and Pro2 expression from hypothetical previous experiment by another researcher.

**Figure 1**. Example fluorescence data from hypothetical experiment. Data represents relative expression units as Fluorescence_Sample_/OD_Sample_.

**Source of error:**

Cells were grown in LB which contains 172 mM NaCl. The researcher did not wash the cells or change the buffer before using the BirA ligase kit. During step 6a, cells were diluted in LB to reach the desired OD and concentration of Pro2. In an optimal protocol, the researcher would have washed the cells prior the the BirA ligation and resuspended in a low-sodium buffer, such as PBS, as described in the BirA Standard of Practice. NaCl decreases BirA activity, and over 100 mM NaCl decreases activity by over 50%. BirA could not ligate properly so streptavidin was then not able to label properly.

**Table 1.** Additional information known by the leader that can be provided upon request

| **Meeting Notes for the Leader**  Not to be shared with the group | |
| --- | --- |
| Other researchers’ experiments | - Researcher 1: culturing *E. coli* with homologous Pro1 for other assays (working) - Researcher 2: using plate reader for green fluorescent protein assays (working) |
| Storage information | - BirA kit was used twice previously but was kept on ice and immediately stored in -80 °C freezer. - The conjugated streptavidin is new and has been stored in the -20 °C freezer. It has only been opened with the tube on ice and the lights dimmed. |
| Protein information | - Your protein of interest, Pro1, and its homolog, Pro2, are both expressed extracellularly. You have previously determined that Pro1 is being secreted out of the cell and is not trapped within the cell membrane. |
| Source of error | - Cells were grown in LB which contains 172 mM NaCl. The researcher did not wash the cells or change the buffer before using the BirA ligase kit. BirA cannot function properly at high levels of NaCl. |
| Hints for group | - You are able to look up standards of practice and protocols for anything mentioned, especially for the kits and antibodies. - You can ask questions about what the previous student did or did not do in their protocol. |

Works Cited

1. B. K. Kay, S. Thai, V. V. Volgina, in *High Throughput Protein Expression and Purification: Methods and Protocols*, S. A. Doyle, Ed. (Humana Press, Totowa, NJ, 2009; https://doi.org/10.1007/978-1-59745-196-3_13), pp. 185–198.

2. D. Beckett, E. Kovaleva, P. J. Schatz, *Protein Sci.* **8**, 921–929 (1999).

3. M. Fairhead, M. Howarth, in *Site-Specific Protein Labeling: Methods and Protocols*, A. Gautier, M. J. Hinner, Eds. (Springer, New York, NY, 2015; https://doi.org/10.1007/978-1-4939-2272-7_12), pp. 171–184.

4. M. González *et al.*, *J. Biol. Chem.* **272**, 11288–11294 (1997).

5. Avidin and Streptavidin Conjugates—Section 7.6 - US, (available at https://www.thermofisher.com/us/en/home/references/molecular-probes-the-handbook/antibodies-avidins-lectins-and-related-products/avidin-streptavidin-neutravidin-and-captavidin-biotin-binding-proteins-and-affinity-matrices.html).

6. C. M. Dundas, D. Demonte, S. Park, *Appl. Microbiol. Biotechnol.* **97**, 9343–9353 (2013).

7. Avidity, Reaction conditions for BirA biotin ligase, (available at https://www.avidity.com/commerce/product.asp?NUMBER=1).

8. Streptavidin, Alexa Fluor^TM^ 647 conjugate, (available at https://www.thermofisher.com/order/catalog/product/S21374).

9. T. Mahmood, P.-C. Yang, *North Am. J. Med. Sci.* **4**, 429–434 (2012).

10. G. W. Chong *et al.*, *Proc. Natl. Acad. Sci.* **119**, e2119964119 (2022).
