# Supplementary material for: Teaching troubleshooting skills to graduate students
Source: eLife. 2024 Sep 17;13:e100761. doi: 10.7554/eLife.100761 (PMC11407763; doi:10.7554/eLife.100761)
Supplement: Supplementary file 1. — For each scenario there is a Word file that contains the following: background information; a description of the scenario; the protocol for the experiment that produced the unexpected result; the results of the experiment; information on the source of the error; background information that can be used to answer questions; and references. There is also a PowerPoint file for each scenario that contains example slides that can be used in real meetings. There are also templates for the Word and PowerPoint files. [file elife-100761-supp1.zip › Final Scenarios/Example5.pptx]

## Slide 1
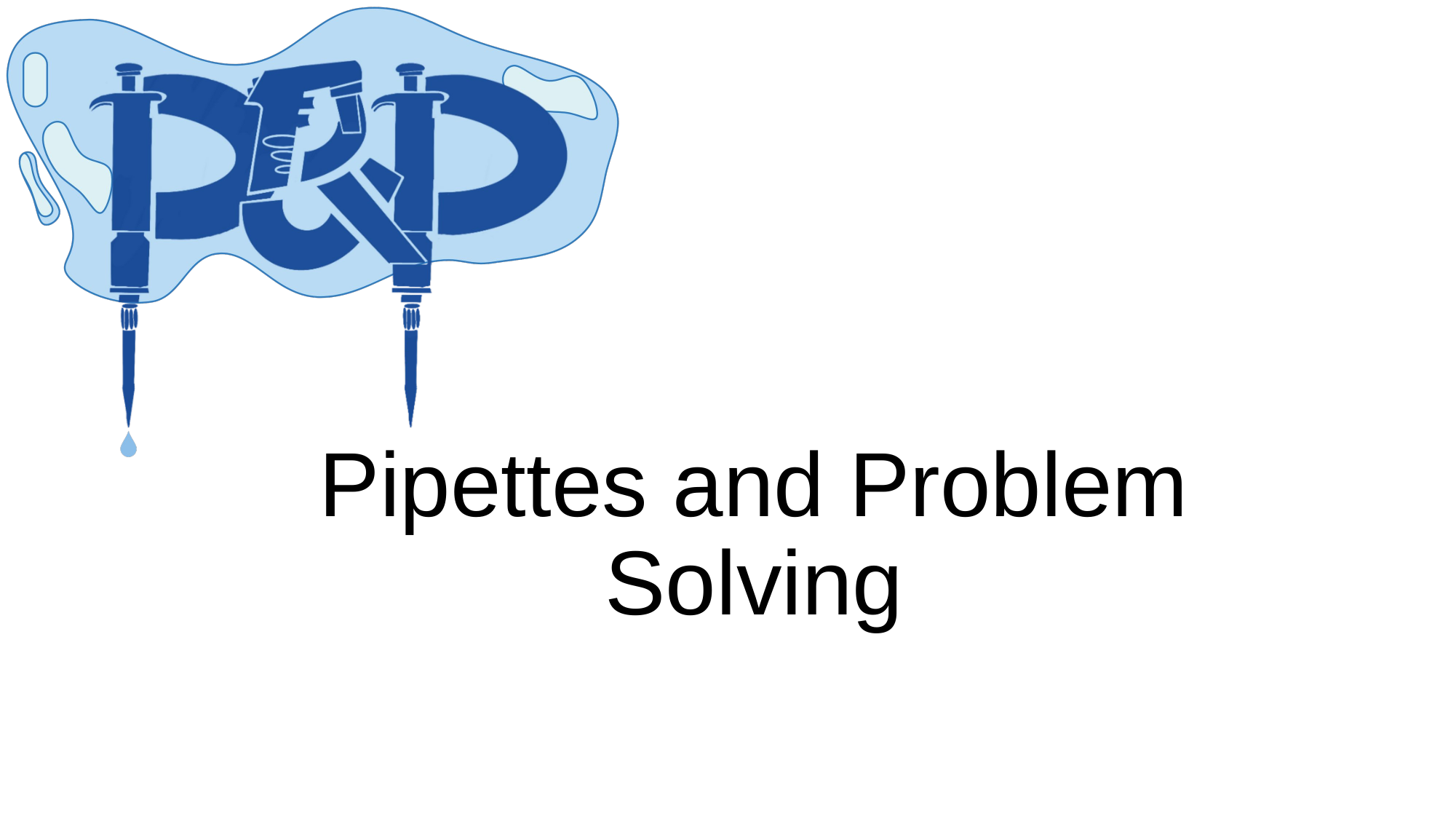

# Pipettes and Problem Solving

## Slide 2
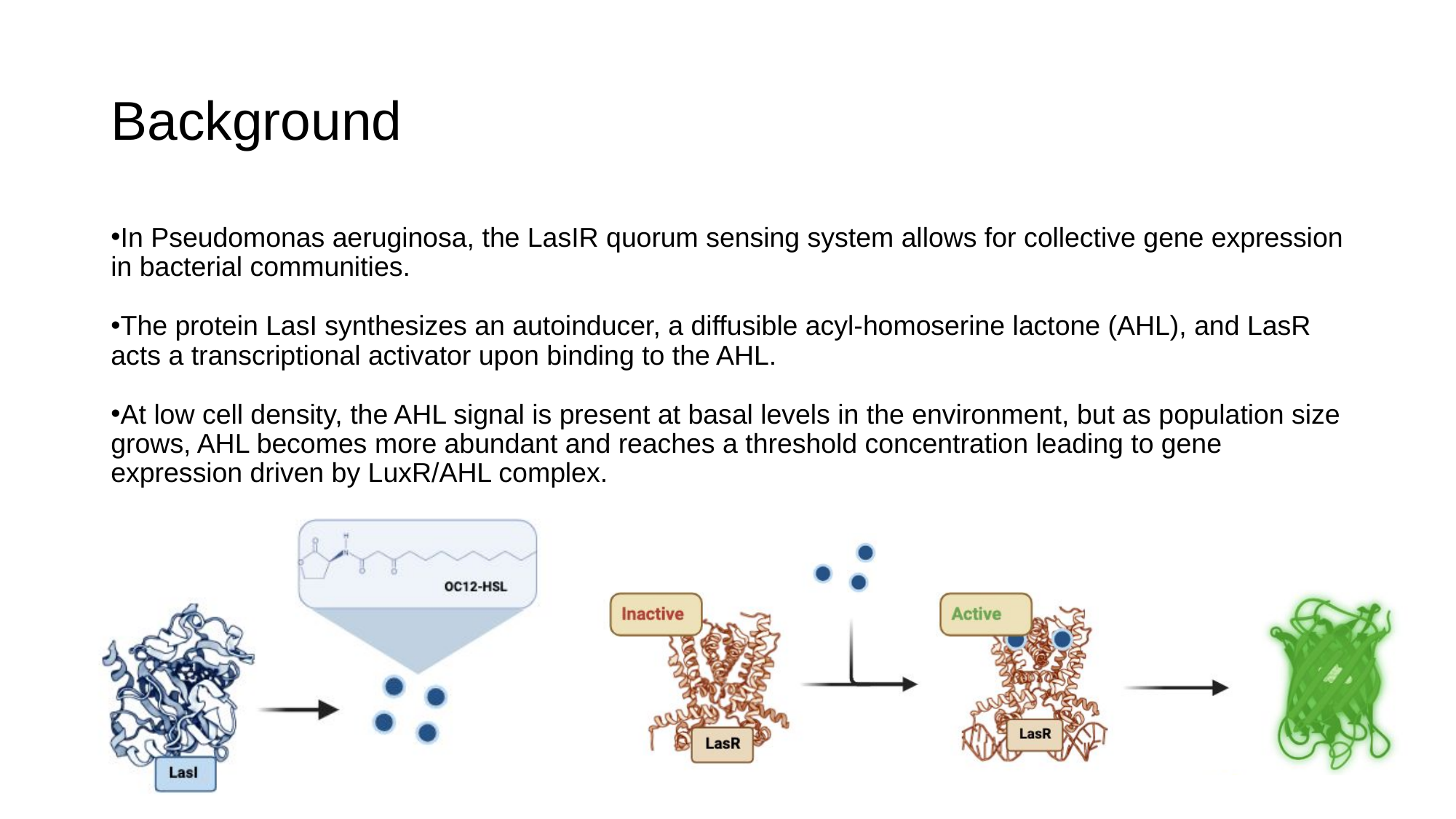

# Background
In Pseudomonas aeruginosa, the LasIR quorum sensing system allows for collective gene expression in bacterial communities.
The protein LasI synthesizes an autoinducer, a diffusible acyl-homoserine lactone (AHL), and LasR acts a transcriptional activator upon binding to the AHL.
At low cell density, the AHL signal is present at basal levels in the environment, but as population size grows, AHL becomes more abundant and reaches a threshold concentration leading to gene expression driven by LuxR/AHL complex.

## Slide 3
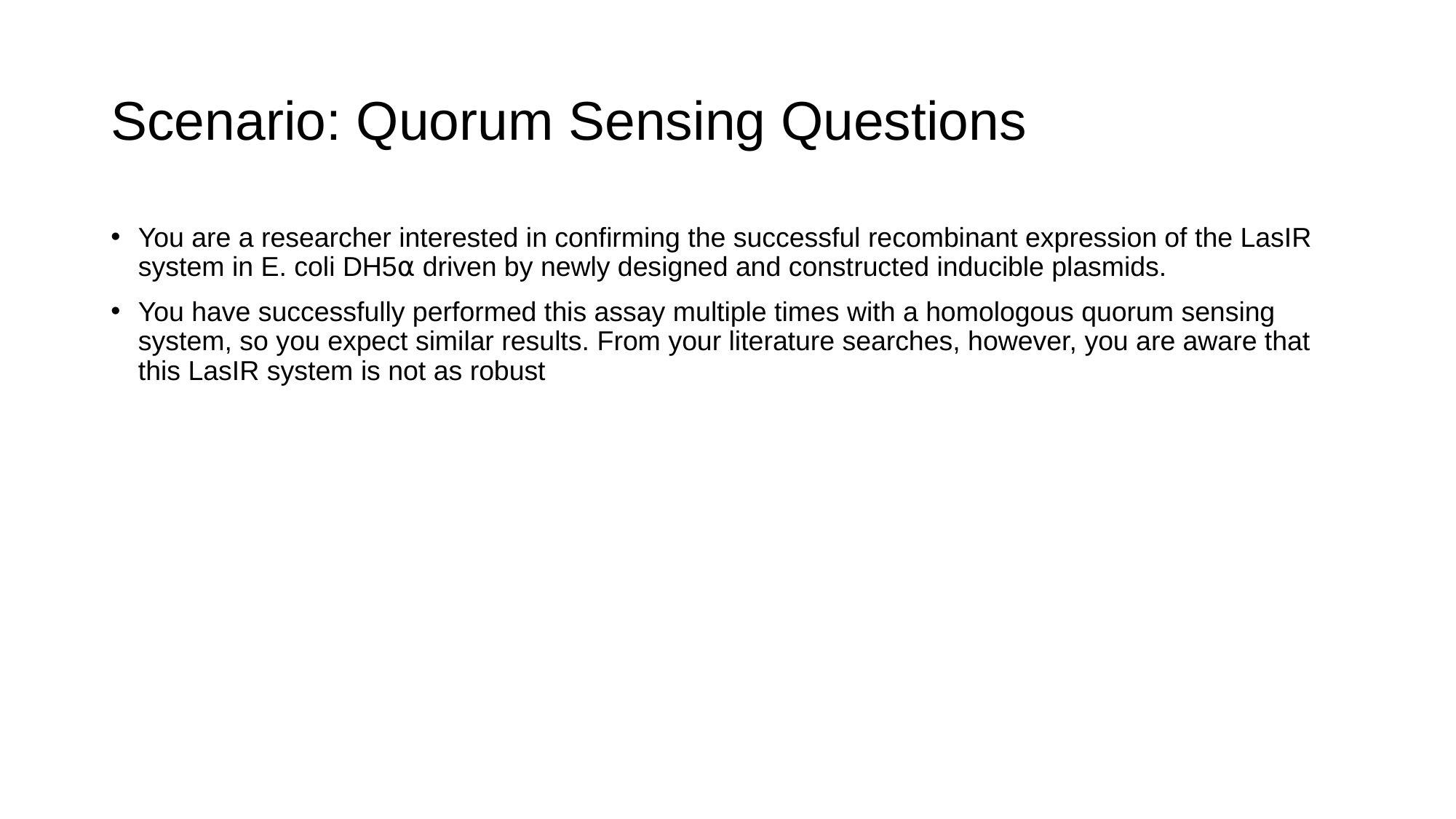

# Scenario: Quorum Sensing Questions
You are a researcher interested in confirming the successful recombinant expression of the LasIR system in E. coli DH5⍺ driven by newly designed and constructed inducible plasmids.
You have successfully performed this assay multiple times with a homologous quorum sensing system, so you expect similar results. From your literature searches, however, you are aware that this LasIR system is not as robust

## Slide 4
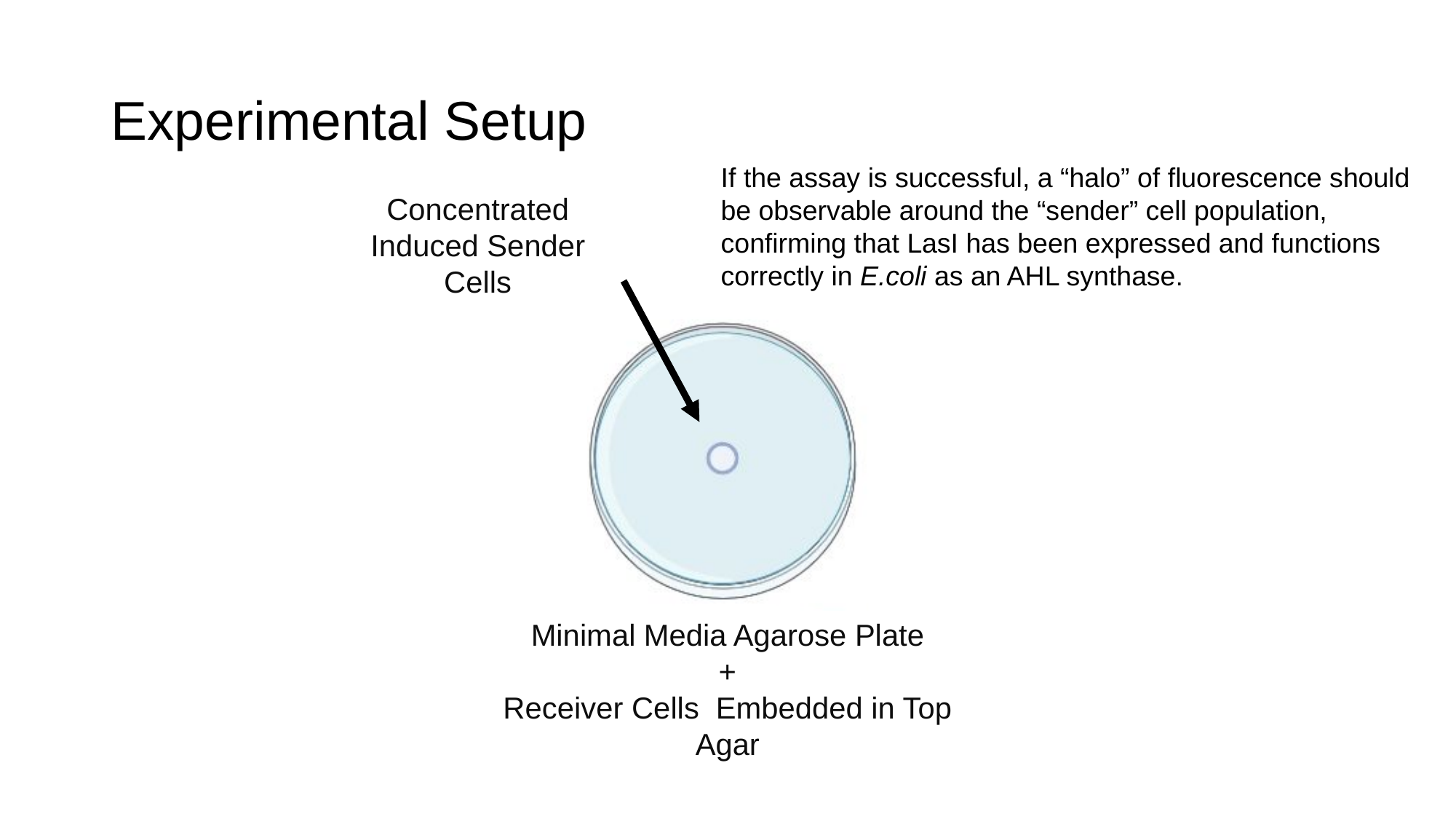

# Experimental Setup
If the assay is successful, a “halo” of fluorescence should be observable around the “sender” cell population, confirming that LasI has been expressed and functions correctly in E.coli as an AHL synthase.
Concentrated Induced Sender Cells
Minimal Media Agarose Plate
+
Receiver Cells Embedded in Top Agar

## Slide 5
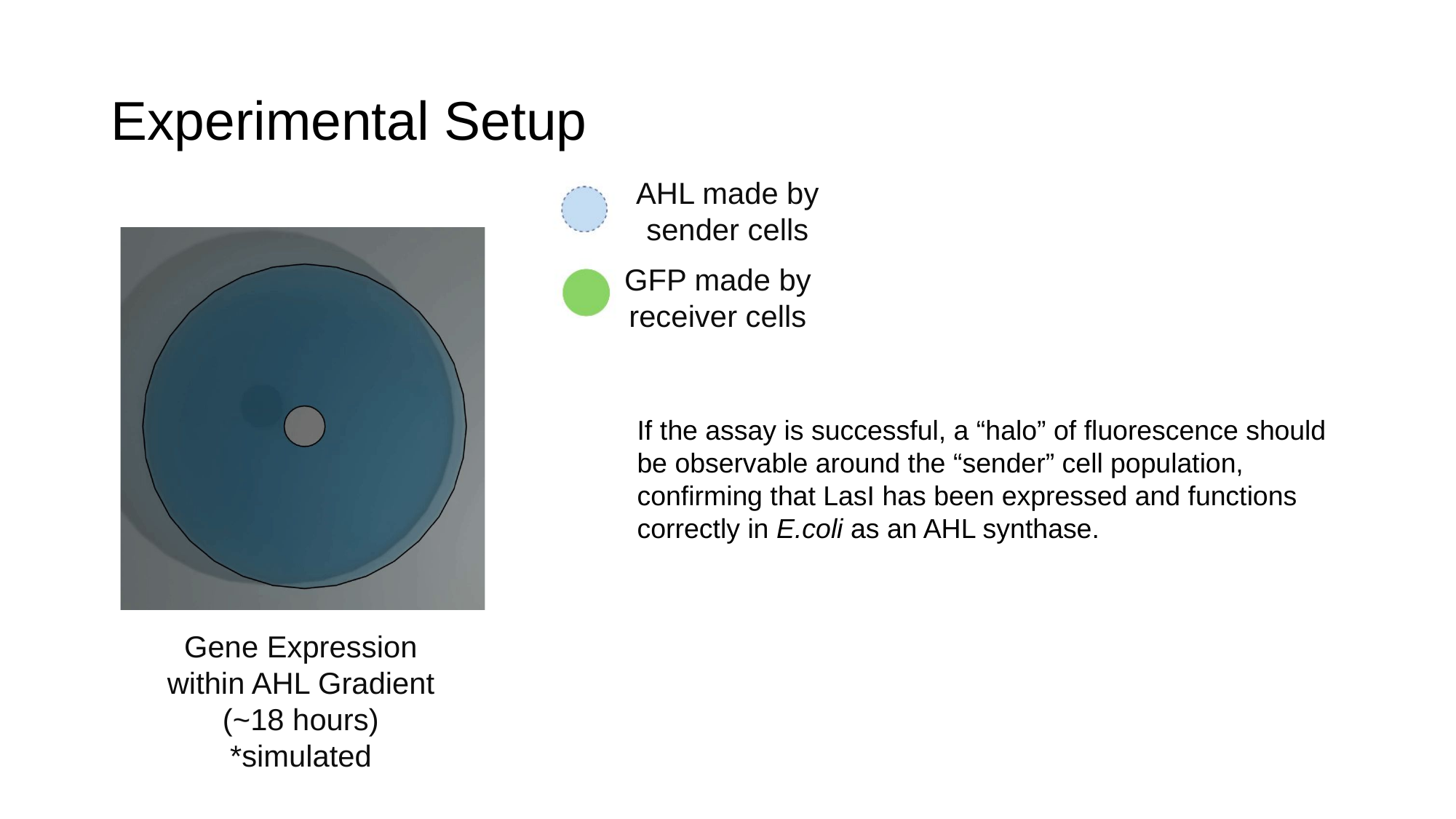

# Experimental Setup
AHL made by sender cells
GFP made by receiver cells
If the assay is successful, a “halo” of fluorescence should be observable around the “sender” cell population, confirming that LasI has been expressed and functions correctly in E.coli as an AHL synthase.
Gene Expression
within AHL Gradient
(~18 hours)
*simulated

## Slide 6
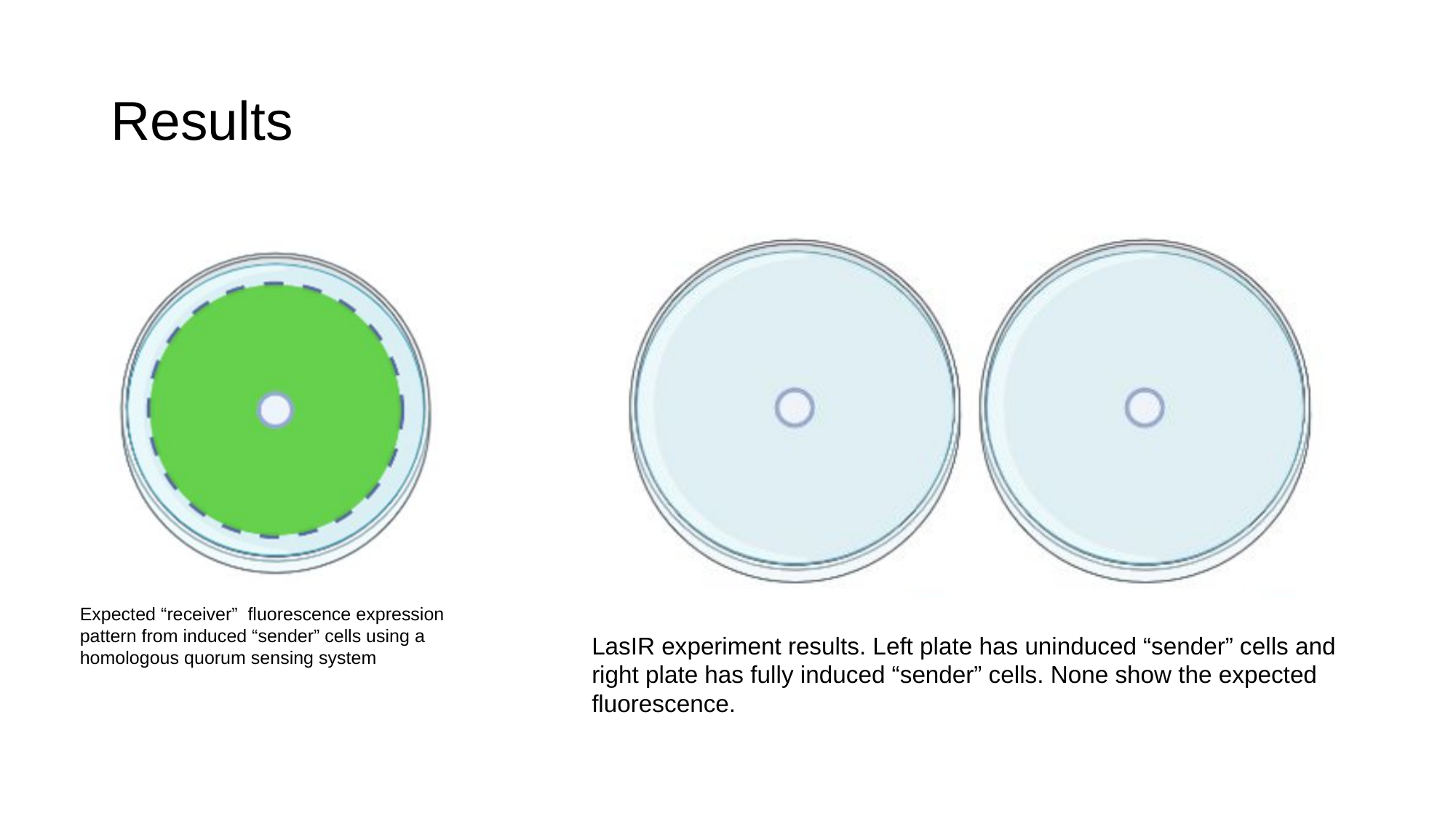

# Results
Expected “receiver” fluorescence expression pattern from induced “sender” cells using a homologous quorum sensing system
LasIR experiment results. Left plate has uninduced “sender” cells and right plate has fully induced “sender” cells. None show the expected fluorescence.

## Slide 7
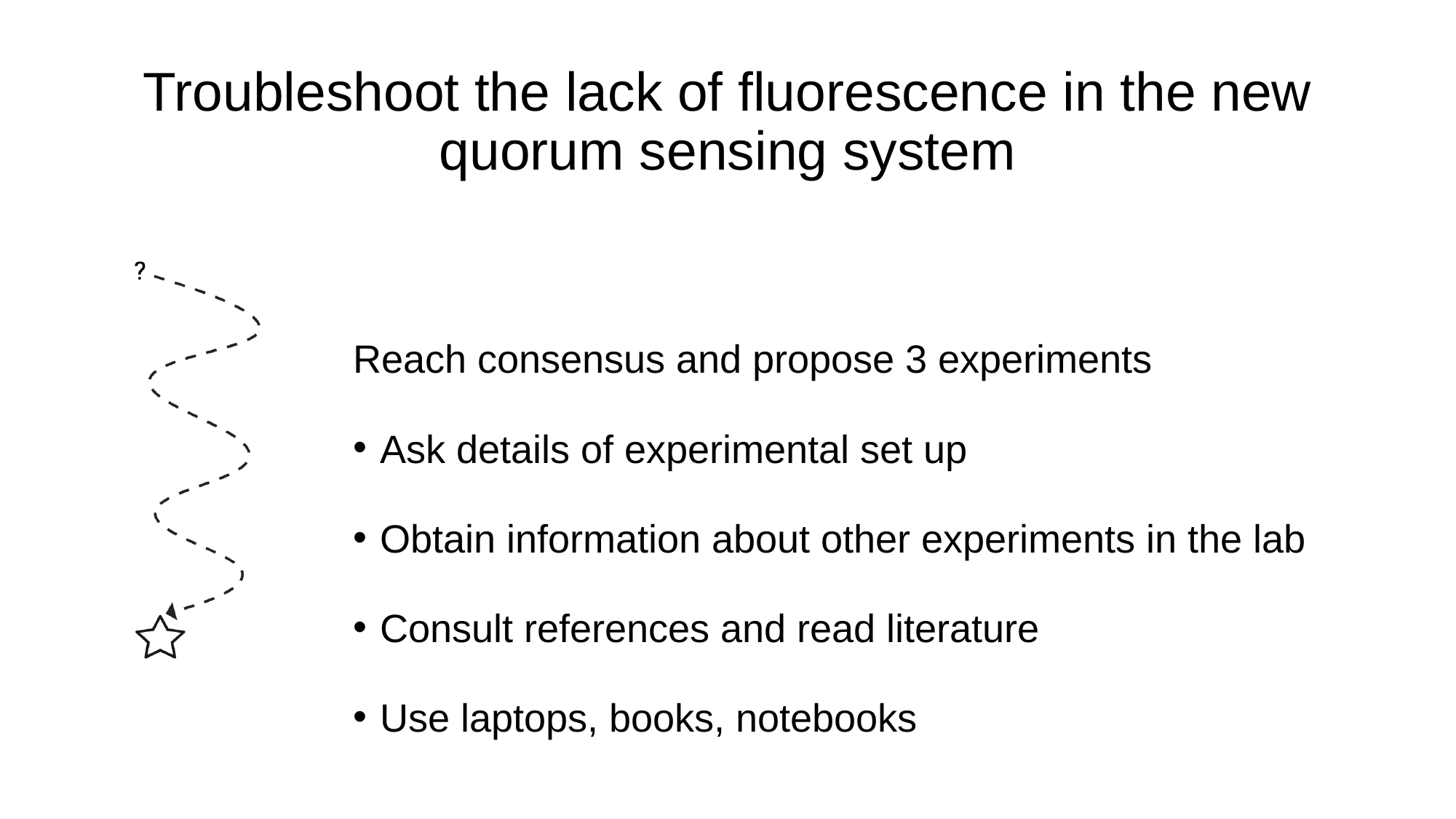

# Troubleshoot the lack of fluorescence in the new quorum sensing system
Reach consensus and propose 3 experiments
Ask details of experimental set up
Obtain information about other experiments in the lab
Consult references and read literature
Use laptops, books, notebooks

## Slide 8
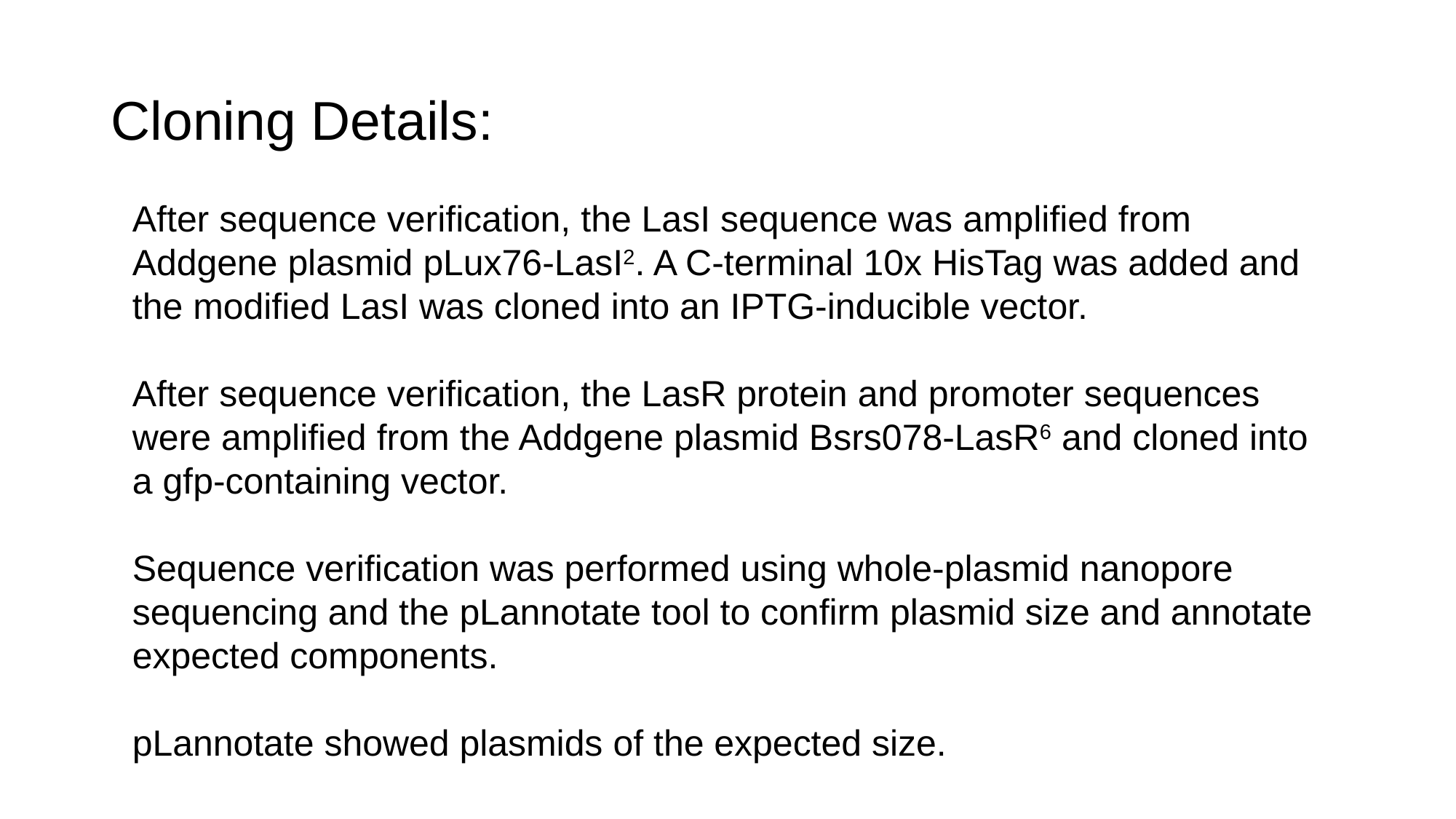

# Cloning Details:
After sequence verification, the LasI sequence was amplified from Addgene plasmid pLux76-LasI2. A C-terminal 10x HisTag was added and the modified LasI was cloned into an IPTG-inducible vector.
After sequence verification, the LasR protein and promoter sequences were amplified from the Addgene plasmid Bsrs078-LasR6 and cloned into a gfp-containing vector.
Sequence verification was performed using whole-plasmid nanopore sequencing and the pLannotate tool to confirm plasmid size and annotate expected components.
pLannotate showed plasmids of the expected size.

## Slide 9
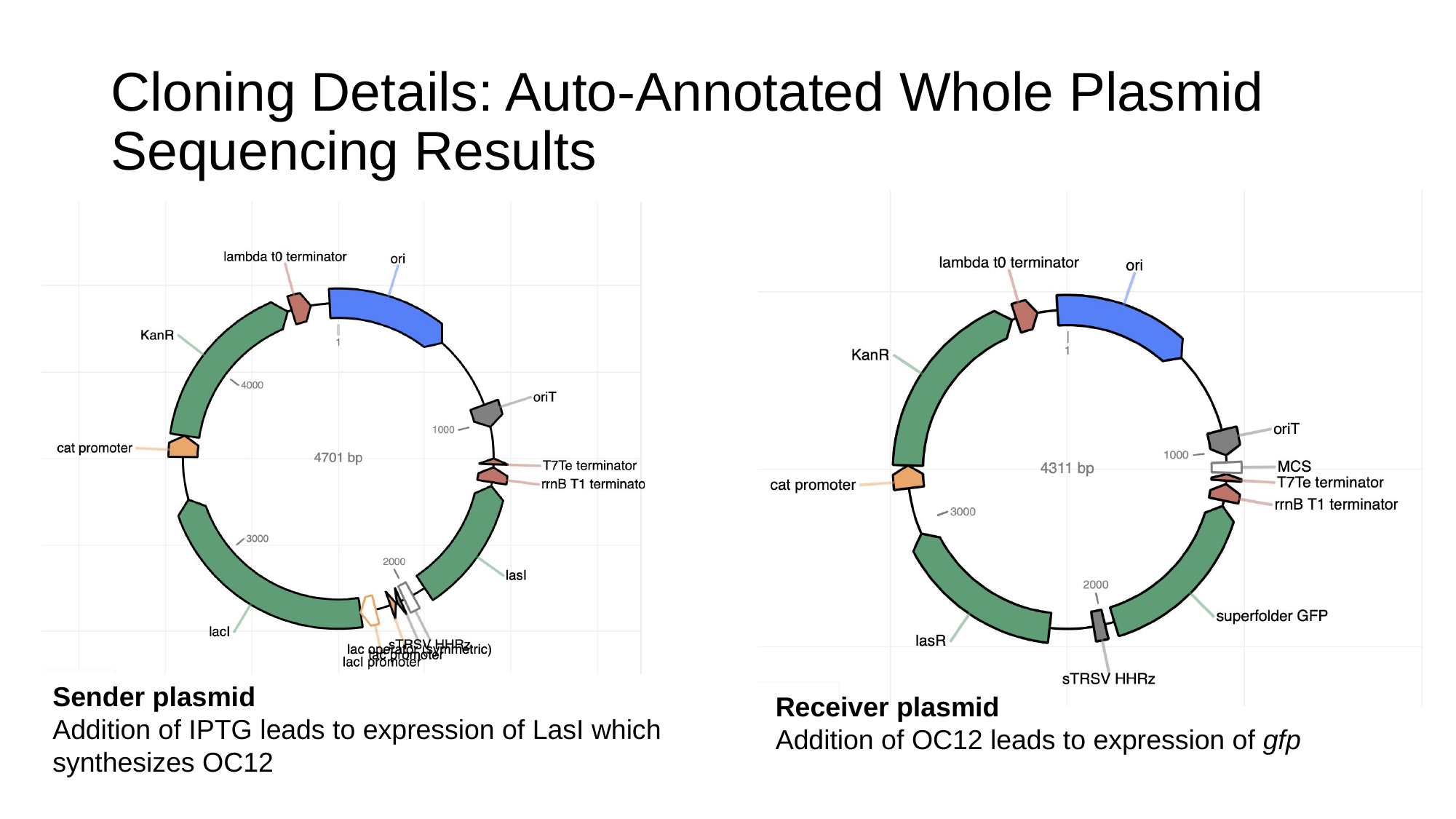

# Cloning Details: Auto-Annotated Whole Plasmid Sequencing Results
Receiver plasmid
Addition of OC12 leads to expression of gfp
Sender plasmid
Addition of IPTG leads to expression of LasI which synthesizes OC12

## Slide 10
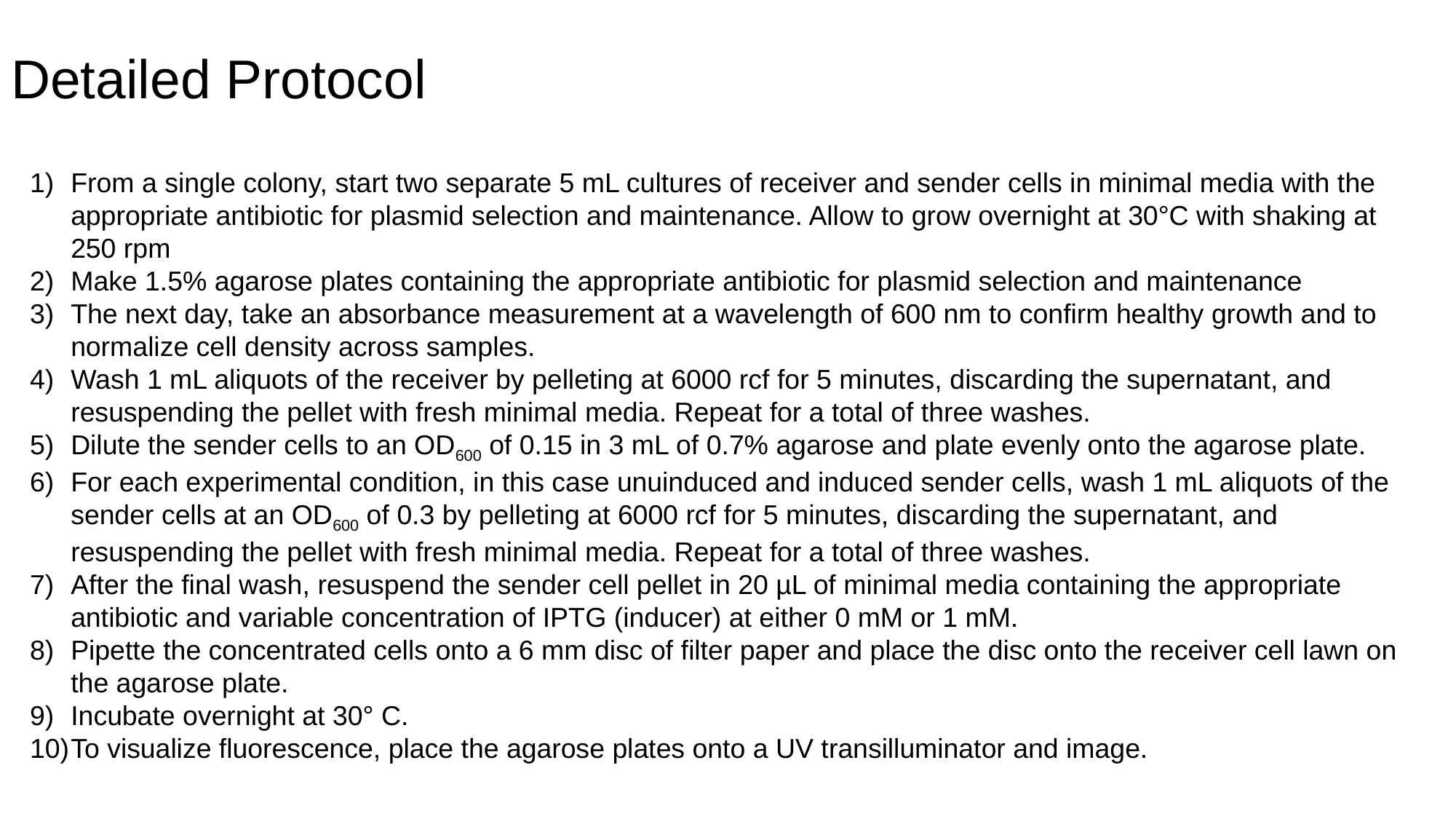

# Detailed Protocol
From a single colony, start two separate 5 mL cultures of receiver and sender cells in minimal media with the appropriate antibiotic for plasmid selection and maintenance. Allow to grow overnight at 30°C with shaking at 250 rpm
Make 1.5% agarose plates containing the appropriate antibiotic for plasmid selection and maintenance
The next day, take an absorbance measurement at a wavelength of 600 nm to confirm healthy growth and to normalize cell density across samples.
Wash 1 mL aliquots of the receiver by pelleting at 6000 rcf for 5 minutes, discarding the supernatant, and resuspending the pellet with fresh minimal media. Repeat for a total of three washes.
Dilute the sender cells to an OD600 of 0.15 in 3 mL of 0.7% agarose and plate evenly onto the agarose plate.
For each experimental condition, in this case unuinduced and induced sender cells, wash 1 mL aliquots of the sender cells at an OD600 of 0.3 by pelleting at 6000 rcf for 5 minutes, discarding the supernatant, and resuspending the pellet with fresh minimal media. Repeat for a total of three washes.
After the final wash, resuspend the sender cell pellet in 20 µL of minimal media containing the appropriate antibiotic and variable concentration of IPTG (inducer) at either 0 mM or 1 mM.
Pipette the concentrated cells onto a 6 mm disc of filter paper and place the disc onto the receiver cell lawn on the agarose plate.
Incubate overnight at 30° C.
To visualize fluorescence, place the agarose plates onto a UV transilluminator and image.

## Slide 11
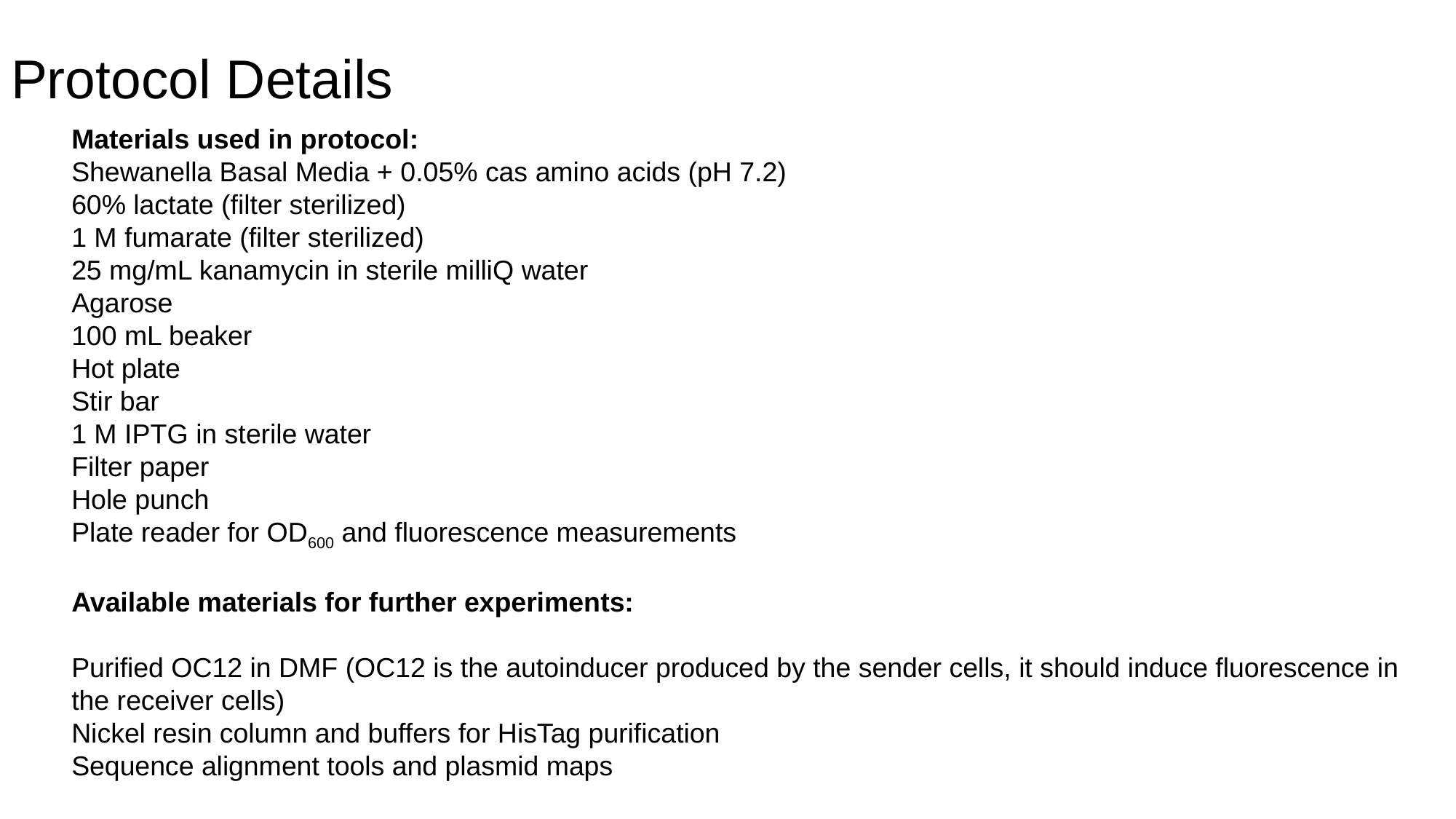

# Protocol Details
Materials used in protocol:
Shewanella Basal Media + 0.05% cas amino acids (pH 7.2)
60% lactate (filter sterilized)
1 M fumarate (filter sterilized)
25 mg/mL kanamycin in sterile milliQ water
Agarose
100 mL beaker
Hot plate
Stir bar
1 M IPTG in sterile water
Filter paper
Hole punch
Plate reader for OD600 and fluorescence measurements
Available materials for further experiments:
Purified OC12 in DMF (OC12 is the autoinducer produced by the sender cells, it should induce fluorescence in the receiver cells)
Nickel resin column and buffers for HisTag purification
Sequence alignment tools and plasmid maps

## Slide 12
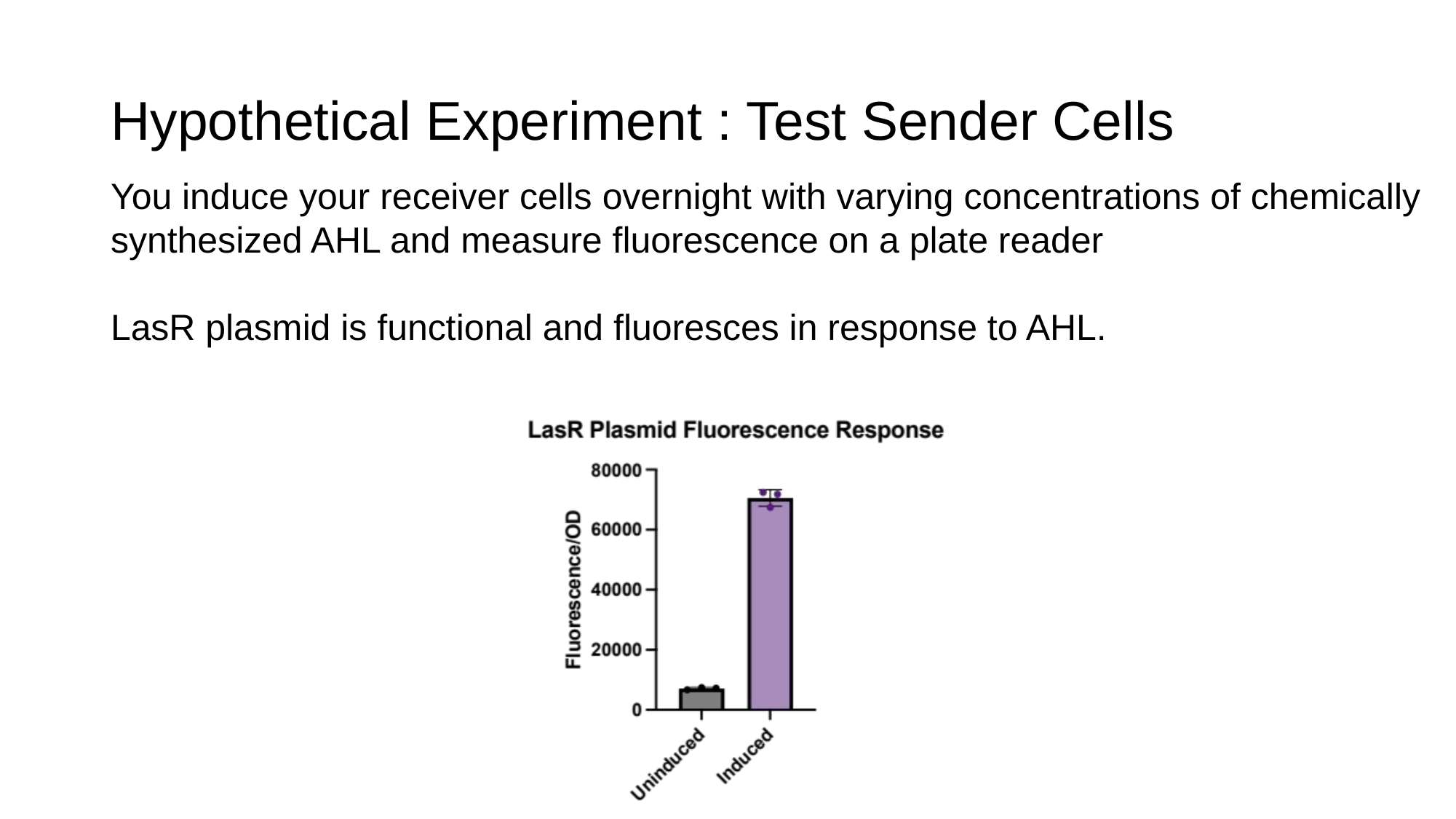

# Hypothetical Experiment : Test Sender Cells
You induce your receiver cells overnight with varying concentrations of chemically synthesized AHL and measure fluorescence on a plate reader
LasR plasmid is functional and fluoresces in response to AHL.

## Slide 13
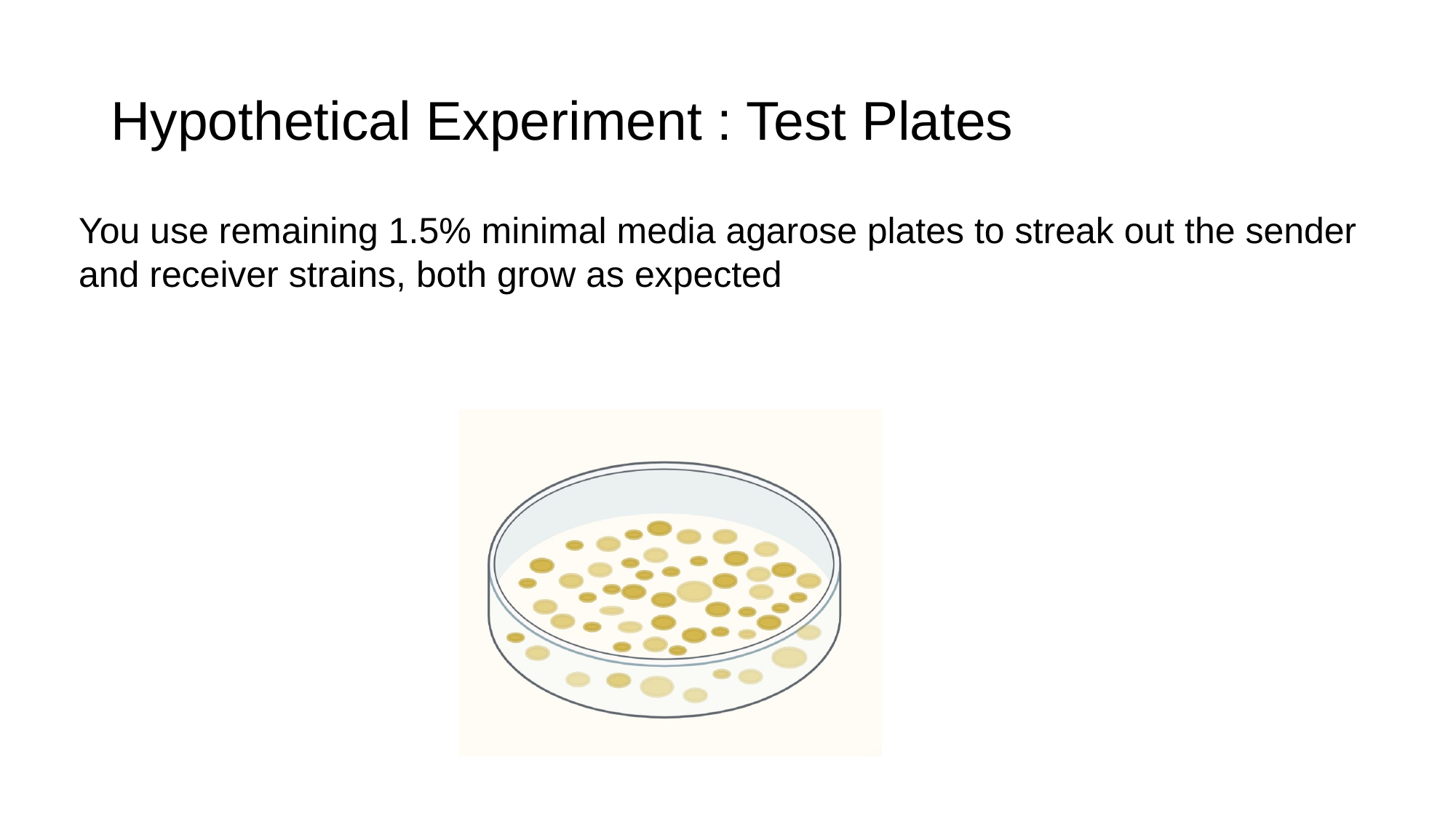

# Hypothetical Experiment : Test Plates
You use remaining 1.5% minimal media agarose plates to streak out the sender and receiver strains, both grow as expected

## Slide 14
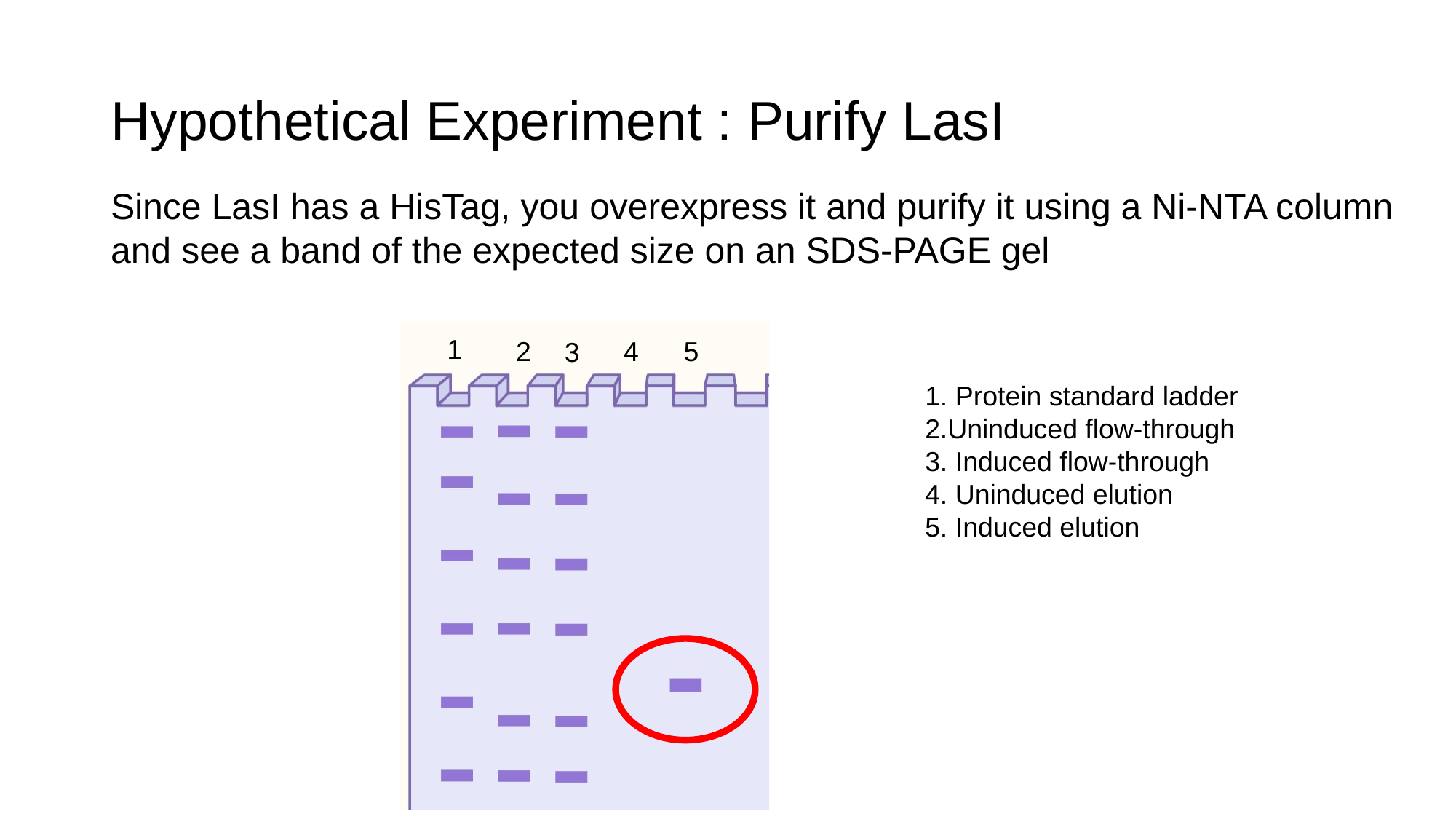

# Hypothetical Experiment : Purify LasI
Since LasI has a HisTag, you overexpress it and purify it using a Ni-NTA column and see a band of the expected size on an SDS-PAGE gel
1
2
4
5
3
1. Protein standard ladder
2.Uninduced flow-through
3. Induced flow-through
4. Uninduced elution
5. Induced elution

## Slide 15
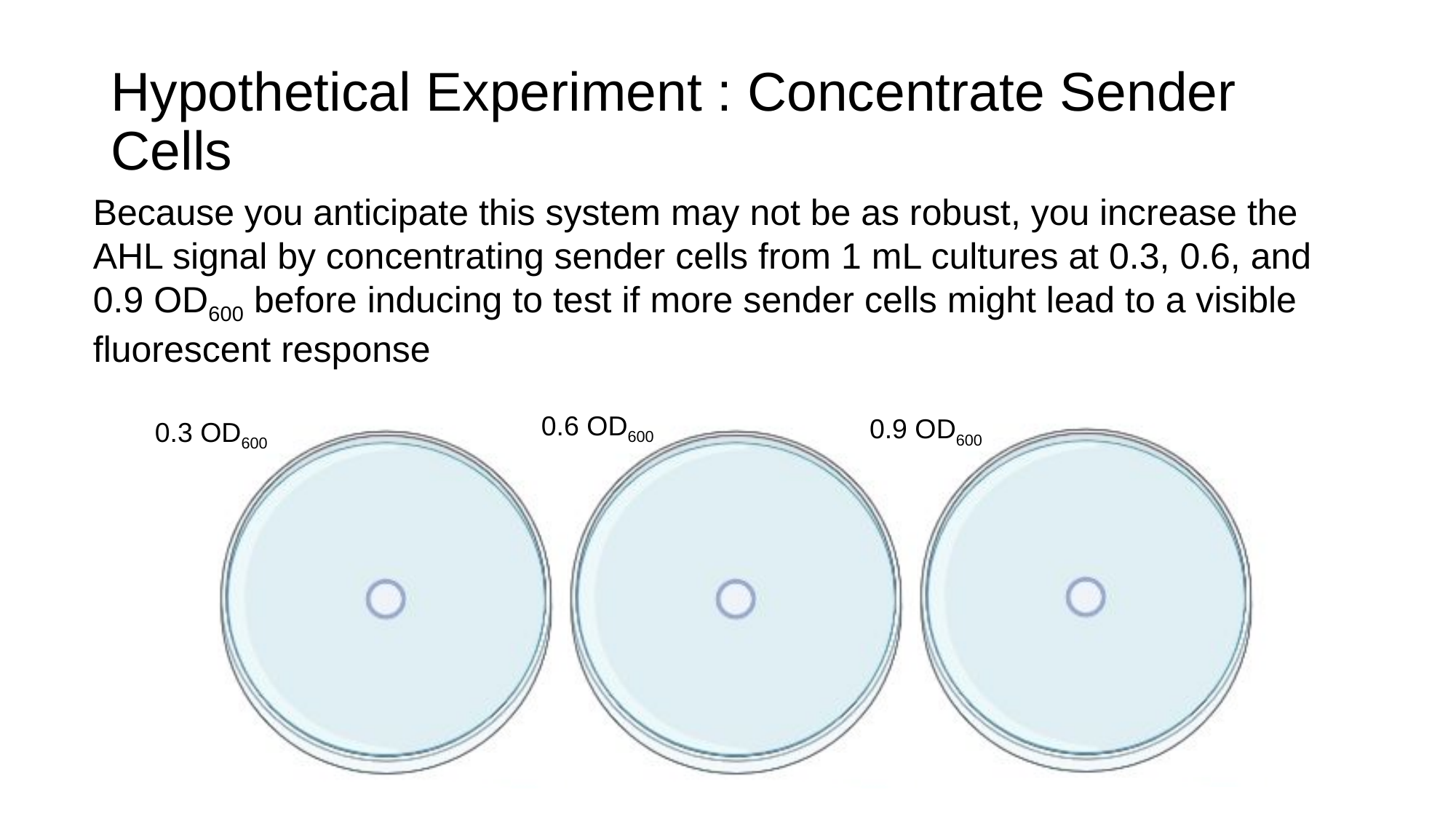

# Hypothetical Experiment : Concentrate Sender Cells
Because you anticipate this system may not be as robust, you increase the AHL signal by concentrating sender cells from 1 mL cultures at 0.3, 0.6, and 0.9 OD600 before inducing to test if more sender cells might lead to a visible fluorescent response
0.6 OD600
0.9 OD600
0.3 OD600

## Slide 16
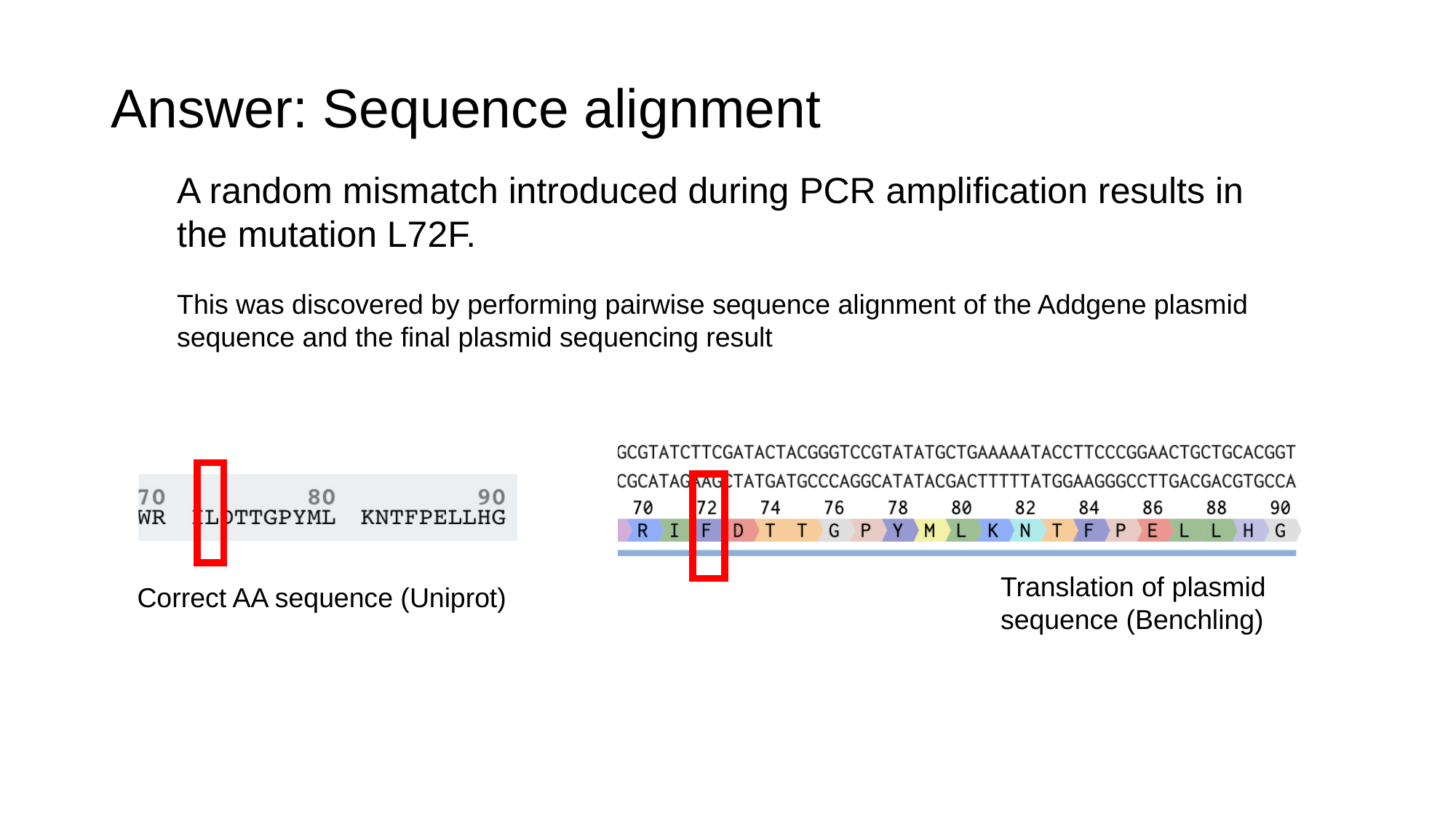

# Answer: Sequence alignment
A random mismatch introduced during PCR amplification results in the mutation L72F.
This was discovered by performing pairwise sequence alignment of the Addgene plasmid sequence and the final plasmid sequencing result
Translation of plasmid sequence (Benchling)
Correct AA sequence (Uniprot)

## Slide 17
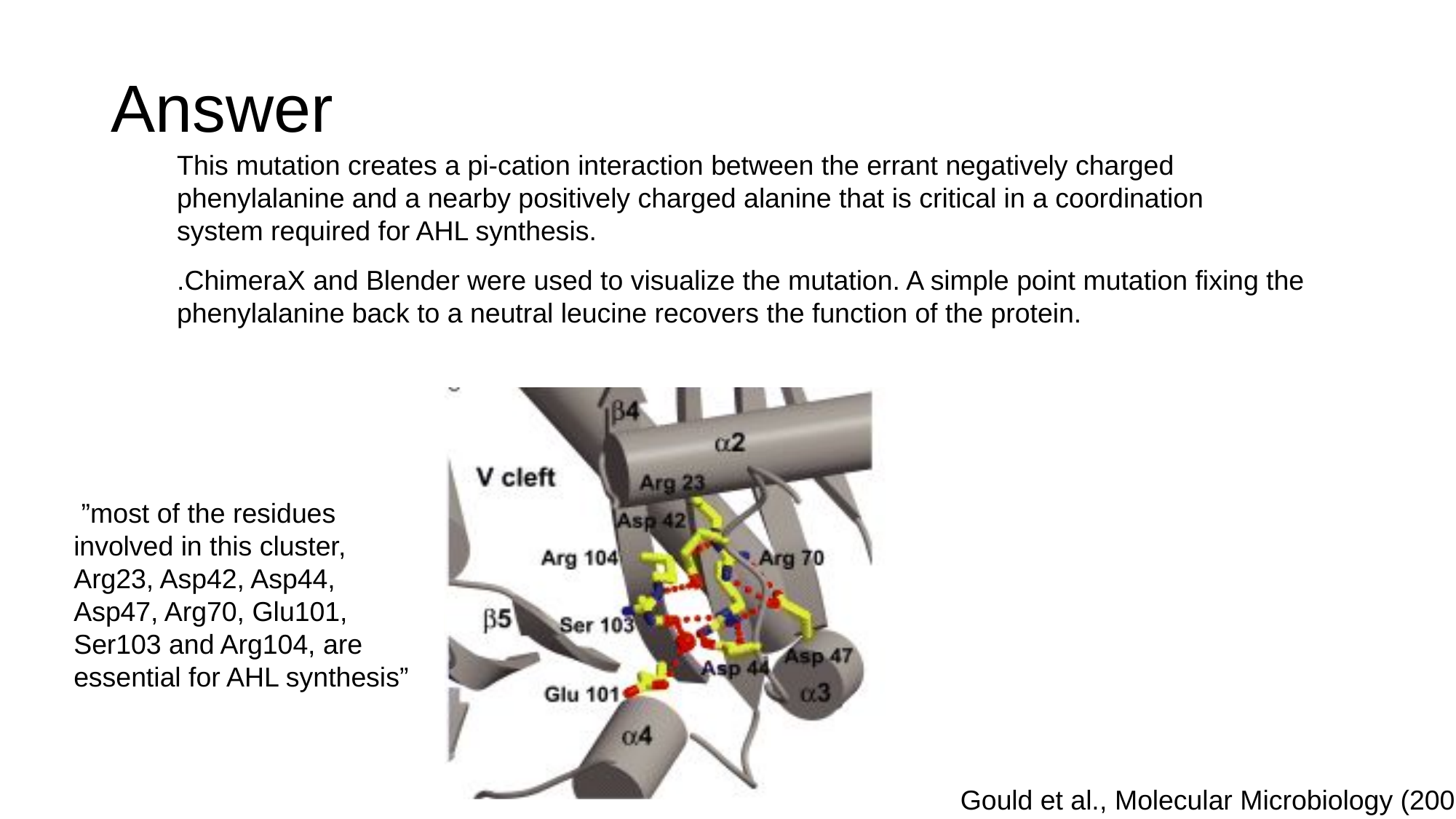

# Answer
This mutation creates a pi-cation interaction between the errant negatively charged phenylalanine and a nearby positively charged alanine that is critical in a coordination system required for AHL synthesis.
.ChimeraX and Blender were used to visualize the mutation. A simple point mutation fixing the phenylalanine back to a neutral leucine recovers the function of the protein.
 ”most of the residues involved in this cluster, Arg23, Asp42, Asp44, Asp47, Arg70, Glu101, Ser103 and Arg104, are essential for AHL synthesis”
Gould et al., Molecular Microbiology (2004)

## Slide 18
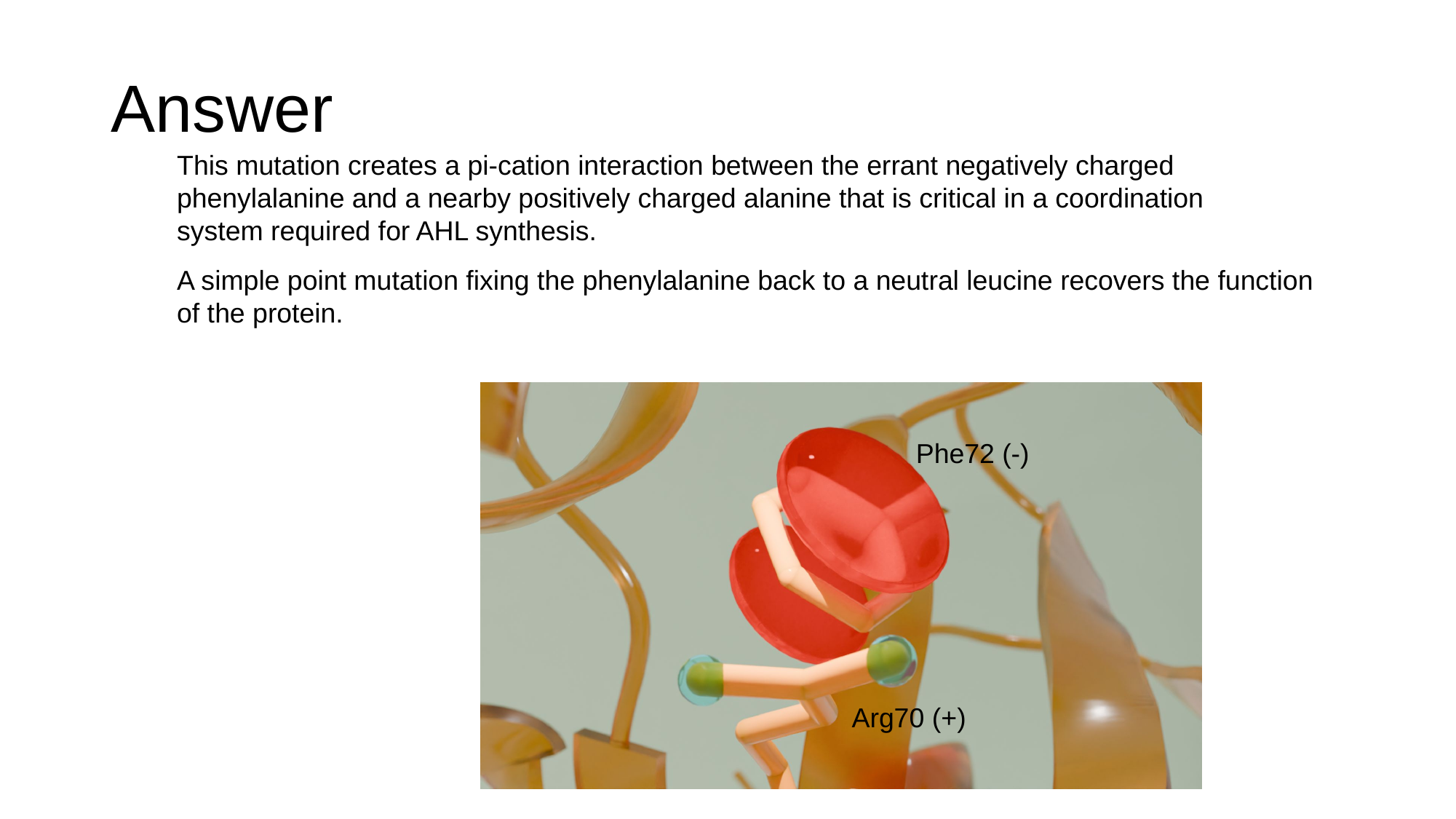

# Answer
This mutation creates a pi-cation interaction between the errant negatively charged phenylalanine and a nearby positively charged alanine that is critical in a coordination system required for AHL synthesis.
A simple point mutation fixing the phenylalanine back to a neutral leucine recovers the function of the protein.
Phe72 (-)
Arg70 (+)
